# Integrated transcriptome and metabolome analysis to investigate the mechanism of intranasal insulin treatment in a rat model of vascular dementia

**DOI:** 10.3389/fphar.2023.1182803

**Published:** 2023-05-15

**Authors:** Liang Tang, Yan Wang, Xujing Gong, Ju Xiang, Yan Zhang, Qin Xiang, Jianming Li

**Affiliations:** ^1^ Department of Basic Biology, Changsha Medical College, Changsha, China; ^2^ Center for Neuroscience and Behavior, Changsha Medical College, Changsha, China; ^3^ The Hunan Provincial University Key Laboratory of the Fundamental and Clinical Research on Functional Nucleic Acid, Changsha Medical College, Changsha, China; ^4^ School of Computer and Communication Engineering, Changsha University of Science and Technology, Changsha, China; ^5^ School of Computer Science and Engineering, Central South University, Changsha, China

**Keywords:** vascular dementia, insulin, multi-omics, hippocampus, integrated analysis

## Abstract

**Introduction:** Insulin has an effect on neurodegenerative diseases. However, the role and mechanism of insulin in vascular dementia (VD) and its underlying mechanism are unknown. In this study, we aimed to investigate the effects and mechanism of insulin on VD.

**Methods:** Experimental rats were randomly assigned to control (CK), Sham, VD, and insulin (INS) + VD groups. Insulin was administered by intranasal spray. Cognitive function was evaluated using the Morris's water maze. Nissl's staining and immunohistochemical staining were used to assess morphological alterations. Apoptosis was evaluated using TUNEL-staining. Transcriptome and metabolome analyses were performed to identify differentially expressed genes (DEGs) and differentially expressed metabolites (DEMs), respectively.

**Results:** Insulin significantly improved cognitive and memory functions in VD model rats (*p* < 0.05). Compared with the VD group, the insulin + VD group exhibited significantly reduced the number of Nissl's bodies numbers, apoptosis level, GFAP-positive cell numbers, apoptosis rates, and p-tau and tau levels in the hippocampal CA1 region (*p* < 0.05). Transcriptomic analysis found 1,257 and 938 DEGs in the VD vs. CK and insulin + VD vs. VD comparisons, respectively. The DEGs were mainly enriched in calcium signaling, cAMP signaling, axon guidance, and glutamatergic synapse signaling pathways. In addition, metabolomic analysis identified 1 and 14 DEMs between groups in negative and positive modes, respectively. KEGG pathway analysis indicated that DEGs and DEMs were mostly enriched in metabolic pathway.

**Conclusion:** Insulin could effectively improve cognitive function in VD model rats by downregulating tau and p-tau expression, inhibiting astrocyte inflammation and neuron apoptosis, and regulating genes involved in calcium signaling, cAMP signaling, axon guidance, and glutamatergic synapse pathways, as well as metabolites involved in metabolic pathway.

## Introduction

Vascular dementia (VD) is a chronic progressive disease that is characterized by mental and cognitive impairment resulting from various cerebrovascular diseases including hypertension, diabetes, hyperlipidemia, cerebral infarction, cerebral hemorrhage, and chronic cerebral ischemia (O'Brien and Thomas, 2015; Iadecola, 2013; Wolters and Ikram, 2019; Lyu et al., 2020; Rajeev et al., 2022). Among the many causes of dementia, VD is the second most common cause after Alzheimer’s disease (AD) (Chang and Chang, 2022). Although an impaired cholinergic system, toxic effects of excitatory amino acids (EAAs), altered synapses and synaptic plasticity, inflammatory responses, and genetic factors have been described to contribute to the development of VD, there is still a lack of understanding about the pathogenesis of VD (Tarkowski, 2002; Ikram et al., 2017; Zhang et al., 2018; Kozubski et al., 2021).

Impairment of brain energy metabolism is one of the main features of neurodegenerative diseases including VD, Parkinson’s disease (PD), and AD (Li D. P. et al., 2001; Costantini et al., 2008; Perneczky et al., 2008). Patients with VD showed decreased cerebral glucose metabolism in a wide range of cortical areas, and low metabolism was found in the corticohypothalamus and caudate nucleus. Kerrouche et al. (2006) reported that lower glucose metabolism was found mainly in the hippocampal region and orbitofrontal, posterior cingulate, and posterior parietal cortices of VD patients compared to AD patients. In addition, insulin resistance (IR) was reported to play an important role in the pathogenesis of VD, which could promote the metabolism of β-amyloid (Aβ) and tau proteins, and lead to metabolic disorders, and ultimately cognitive dysfunction (Leonard and Wegener, 2020). IR can dysregulate oxygen metabolism in brain tissue, leading to an increase in glycosylation product levels and impairment of cerebrovascular endothelial function, resulting in cerebrovascular atherosclerosis, narrowing of the cerebrovascular lumen and reduction in blood perfusion, which in turn leads to cognitive impairment (Pugazhenthi et al., 2017). Thus, it is possible that therapeutic options used for the treatment of diabetes may also be used for the prevention and treatment of VD.

Insulin is an important hormone in regulating blood glucose levels. Recent studies have demonstrated that insulin and its receptors are present in the human and mammalian central nervous systems (CNS) (Park, 2001; Qeva et al., 2022). There is a strong relationship between insulin and learning memory, anti-apoptosis, neurogenesis, and synaptic plasticity in the CNS (Xing et al., 2009; Bayram et al., 2022). Currently, studies on insulin use for dementia focus mostly on AD patients, while the treatment of VD is rarely reported (Chapman et al., 2018; Akhtar and Sah, 2020; Chung et al., 2022). Sain et al. (2011) revealed that insulin could protect the hippocampus against cerebral ischemic injury in VD rat models and cognitive function can be improved after administration of an insulin sensitizer. Studies on ischemic brain injury in animals have shown that insulin reduces ischemic brain injury independent of hypoglycemia, indicating a central direct protective effect (Voll and Auer, 1991; Lioutas et al., 2015). The research on polypeptide drug delivery routes focuses on mucosal delivery based on nasal mucosa, pulmonary mucosa, and rectal mucosa (Chono, 2009; Bernocchi et al., 2016; Bamberg et al., 2021). However, intranasal administration has the advantages of rapid absorption, rapid onset, avoidance of hepatic first-pass effects, high bioavailability, ease of use, and direct delivery of drugs to the brain by bypassing the blood-brain barrier. William Frey II has first proposed and patented intranasal administration for the direct delivery of therapeutics to the CNS (Frey, 1991; Frey, 1997; Frey, 2001). The effect of intranasal insulin administration on AD and PD has been widely reported (Hallschmid, 2021; Iravanpour et al., 2021). Jauch-Chara et al. (2012) revealed that intranasal insulin reduces food consumption via enhancement of the neuroenergetic level. However, little is known concerning the effect of intranasal insulin on VD and the underlying mechanism.

Multi-omics analysis, including genomics, transcriptomics, proteomics and metabolomics analysis, is an effective tool for finding new targets or mechanisms (Jiang et al., 2022; Park et al., 2022). Since the differentially expressed genes obtained by a single transcriptome analysis act on a variety of substances in metabolic pathways, the factor causing the accumulation of metabolites cannot be directly determined (Advani and Kumar, 2021). However, metabolomics can identify differences in specific metabolite types and ion abundance in related pathways, which is of great importance for the comprehensive analysis of metabolite biosynthesis regulation related to the occurrence and development of diseases (Wang et al., 2011). Therefore, it is possible to fully explore the molecular mechanism of insulin intervention in the occurrence and development of VD by combining transcriptomic and metabolomic analyze.

To identify new theoretical VD-related targets of insulin treatment, the present study was designed to investigate the effect of insulin on bilateral common carotid artery ligation (2-VO) induced VD model rats and to combine and analyze the deregulated transcriptome and metabolome profiles of VD model rats treated with intranasal insulin.

## Materials and methods

### Animal management

All animal experiments were conducted at the Neuroscience and Behavioral Research Center of Changsha Medical College. The experimental procedure followed ethical standards and the Declaration of Helsinki, as well as national and international standards. The research procedure was approved by the Ethics Committee of Changsha Medical University (CSMUE-20220182). Sixty Sprague-Dawley (SD) rats (male, 300 ± 25 g, 6 months old) were purchased from Hunan Slyke Jingda Biotechnology Co., Ltd. The rats were housed under controlled conditions (temperature: 23°C ± 0.6°C, relative humidity: 55% ± 8%, light and dark period = 12 h:12 h). After adaptive feeding for 7 days, rats were randomly assigned to 4 groups (n = 15): (1) control (CK) group, (2) sham group (3) VD group, (3) insulin (INS) + VD group. Rats in the CK group did not receive any treatment. Rats in the sham group underwent separation of the carotid artery without ligation. Rats in the other 2 groups underwent 2-VO. The 2-VO procedure was performed according to Xu et al. (2012). The corresponding drugs were administered by intranasal spray once daily for 8 weeks starting on the 1^st^ postinjury day. The limbs of mice were fixed in the horizontal position and the neck was kept straight to ensure smooth breathing. A total of 1U/10 μL insulin was dripped alternately through both nostrils using a microdoser (10 μL). After the infusion, the mice remained in position for 5–10 s to ensure that the fluid was fully absorbed. The dose and duration of insulin treatment followed the studies conducted by Zeng et al. (2013) and Wu et al. (2019).

(1) CK group: normal saline (0.9%), 0.5 mL/d;
(2) Sham group: normal saline (0.9%), 0.5 mL/d;
(3) VD group: normal saline (0.9%), 0.5 mL/d;
(4) Insulin (INS) + VD group: 1 U/kg/d;

### Morris water maze

The Morris water maze test, which tests spatial navigation, was performed on the 50th −56th postinjury days. The procedure of the Morris water maze test followed the study conducted by Vorhees and Williams (2006). The ethological data collection procedure followed our previous studies (Li D. P. et al., 2001; Li X. G. et al., 2001; Tang et al., 2019).

### Tissue collection

Rats were peritoneally anesthetized with pentobarbital sodium (40 mg/kg). Six whole brain samples were placed in 4% paraformaldehyde for morphological analysis. The other 6 whole brains and 3 hippocampal tissues were frozen in liquid nitrogen and then placed in a −80°C refrigerator separately for further metabolomic and transcriptomic analysis.

### Nissl’s staining

Nissl staining was used to determine the distribution of Nissl’s body in the CA1 region of the hippocampus. Tissue sections were placed in the following solutions in order: xylene (15 min), xylene (15 min), anhydrous ethanol (5 min), anhydrous ethanol (5 min), 90% alcohol, 75% alcohol (5 min), and tap water. Nissl’s stain (tolonium chloride) was added and incubated for 5 min. Tissue sections were then differentiated with 1% glacial acetic acid for 2–3 s. Finally, the sections were cleared with xylene for 5 min and examined under a microscope.

### Immunohistochemistry

Postfixed brain tissue blocks (0.5 cm^3^) were immersed in 20%–40% sucrose with 0.1 M phosphate buffer solution (PBS), precipitated sugar in a gradient at 4°C, and then stored in a −80°C refrigerator. 20 μm sections from each sample were collected and placed in culture plates filled with PBS (0.01 M, pH = 7.2), and then transferred to antifreeze cache solution for storage at −20°C. Warmed brain tissue sections were placed in a reaction tube containing 5% normal horse serum and incubated at room temperature for 2 h. Primary antibodies (Anti-p-tau (181) (1:500) (Abcam, Massachusetts, United States); anti-tau (1:500) (Abcam, Massachusetts, United States); anti-Aβ_1-42_ (1:800) (Abcam, Massachusetts, United States); anti-GFAP (1:1,000) (Abcam, Massachusetts, United States) were added and incubated at room temperature for 2 h and at 4°C overnight. Tissues were selected in biotinylated broad-spectrum IgG (PAN:5% horse serum = 1:200) and incubated at room temperature for 2 h. Biotin-vitalin-horseradish peroxidase (ABC) complex was added to 0.01 M PBST buffer and incubated at room temperature for 2 h. The tissues were placed in 0.05% diaminobenzidine (DAB) dye solution, and then 0.03% H_2_O_2_ was added sequentially after preincubation. The tissue patch was immersed in the hematoxylin dye solution for color observation. Sections were then dehydrated through a series of 80%, 90%, 100%, and 100% graded alcohols, cleared twice through xylene, and then embedded in neutral resin.

### TUNEL staining

The tissue sections were sequentially washed in xylene Ⅰ (20 min), xylene Ⅱ (20 min), anhydrous ethanol Ⅰ (10 min), anhydrous ethanol Ⅱ (10 min), 95% alcohol (5 min), 90% alcohol (5 min), 80% alcohol (5 min), 70% alcohol (5 min), and distilled water. Then the cells were covered with protease K and incubated at 37°C for 15 min. The tissues were washed in PBS (pH = 7.4), covered with a film-breaking working solution, incubated at room temperature, and then washed in PBS (pH = 7.4). TUNEL staining was performed using a one-step TUNEL cell apoptosis detection kit (#C1088, Beyotime, Beijing, China). The cells were observed using a fluorescence microscope (Olympus, BX53, Japan.). Morphological changes in the hippocampal CA1 region were measured using Image-Pro Plus 5.1 software (Media Cybernetics, Inc., Bethesda, United States) (https://imagej.en.softonic.com/). The integrated optical density (IOD) of immunoreactivity was analyzed using OptiQuant software (Parkard Instruments, Meriden, CT).

### Transcriptome profiling analysis

Total RNA was extracted from the hippocampus using TRIzol reagent (#R0016, Beyotime, Beijing, China). RNA purity and concentration were determined by ultraviolet spectrophotometry (NanoDrop, Thermo Fisher Technology Company, United States). RNA was selected with an optical density (OD) between 1.8 and 2.0.

The mRNA was enriched with oligo (dT) using magnetic beads, and the mRNA was cleaved into short fragments by adding the cleavage reagent. The fragmented mRNA was used as a template to synthesize one cDNA strand with six-base random primers, and then a two-strand synthesis reaction system was prepared to synthesize double-stranded cDNA, and the double-stranded cDNA was purified using a kit (#A63987, Beckman, America). The purified double-stranded cDNA was then terminally repaired, a tail was added, and a sequencing linker was connected. Then, fragment size selection was performed, and PCR amplification was performed. After the constructed library was qualified by an Agilent 2,100 Bioanalyzer (Agilent, United States). An Illumina HiSeqTM 2,500 sequencer (Hiscan, United States) was used for sequencing. Illumina sequencing was performed at CapitalBio Technology Co., Ltd. (Beijing, China).

Clean sequences were compared to reference genome sequences using HISAT2 software (https://daehwankimlab.github.io/hisat2/). Gene expression levels were calculated using the fragments per kilobase of transcript per million fragments mapped (FPKM) method. Differential expression analyses between the two comparisons (VD vs. CK, insulin + VD vs. VD, with three biological replicates per treatment) were performed using the limma package (1.10.1). Gene with a |log_2_ fold change (FC)|≥1 and an adjusted *p*-value < = 0.05 was considered differentially expressed gene (DEG). Cluster analysis of differential gene expression patterns was performed using the R package. Gene Ontology (GO) annotation (cell components, biological process, and molecular function) (http://www.ebi.ac.uk/QuickGO/) and Kyoto Encyclopedia of Genes and Genomes (KEGG) pathway enrichment (http://www.genome.jp/kegg/) analyses of DEGs were applied.

### Validation of functional gene expression

Three hippocampal RNA samples from the CK, VD, and INS + VD groups were tested. RNA was reverse transcribed into cDNA using Prime-Script™ RT Master Mix (#RR036A, Takara, Japan). qPCR was performed using SYBR^®^Premix^Ex^ Taq™ (#DRR081A, Takara, Japan) on an ABI 7,500 system (ABI, United States). β-Actin expression levels were used as standardized internal control. RNA expression levels were calculated using the 2^−ΔΔCt^ method. Primer sequences are listed in Supplementary Table S1.

### Metabolome profiling analysis

Six hippocampal samples from 3 groups were homogenized using a grinder (30 HZ). A 400 μL solution (methanol: water = 7:3, v/v) containing an internal standard was added. A 300 μL supernatant was collected and stored at −20°C for 30 min. The samples were then centrifuged at 12,000 rpm for 3 min (4°C). A 200 μL aliquot of the supernatant was subjected to LC-MS analysis with the following analytical conditions: UPLC: column, Waters ACQUITY UPLC HSS T3 C18 (1.8 µm, 2.1 mm*100 mm); column temperature, 40°C; flow rate, 0.4 mL/min; injection volume, 2 μL; solvent system, water (0.1% formic acid): acetonitrile (0.1% formic acid); gradient program, 95:5 V/V at 0 min, 10:90 V/V at 11.0 min, 10:90 V/V at 12.0 min, 95:5 V/V at 12.1 min, 95:5 V/V at 14.0 min. The metabolome profiling analysis was performed at CapitalBio Technology Co., Ltd. (Beijing, China).

Unsupervised principal component analysis (PCA) and hierarchical cluster analysis (HCA) were performed using PRCOMP and Complex Heatmap in the R package (www.r-project.org), respectively. DEMs were selected by variable importance in projection (VIP) (VIP ≥ 1), *p*-value (*p*-value ≤ 0.05, Student’s t-test) and absolute Log_2_FC (|Log_2_FC| ≥ 1.0) in two-group analysis. The orthogonal partial least-squares-discriminant analysis (OPLS-DA) results were generated from the R package (Metabo analyst R). Cluster analysis of differentially expressed metabolite (DEM) expression patterns was performed by using the R package. DEMs were annotated using the KEGG compound database (http://www.kegg.jp/kegg/compound/).

### Combined analysis of the transcriptome and metabolome

The KEGG pathways in which DEMs and DEGs were both involved were analyzed based on the results of the transcriptome and metabolome. To detect the upstream and downstream relationships between DEGs and DEMs in regulatory networks, a metabolite regulatory network containing compounds, reactions, enzymes, and genes based on KEGG interaction data was constructed by using Cytoscape (MetScape plugin).

### Statistical analysis

SPSS 25.0 was used for statistical analysis (IBM, United States). For continuous variables, the normal distribution test was performed first. Measurement data conforming to a normal distribution were expressed as the mean ± standard deviation (±s), and one-way ANOVA was used to compare groups. If the variances were homogeneous, the least Significant Difference (LSD) test was used to compare between groups. When variances were not homogeneous, the Welch approximate *F* test was used to compare components. *p* < 0.05 indicated a statistically significant difference.

## Results

### Insulin improves the cognitive function of VD rat

**FIGURE 1:**
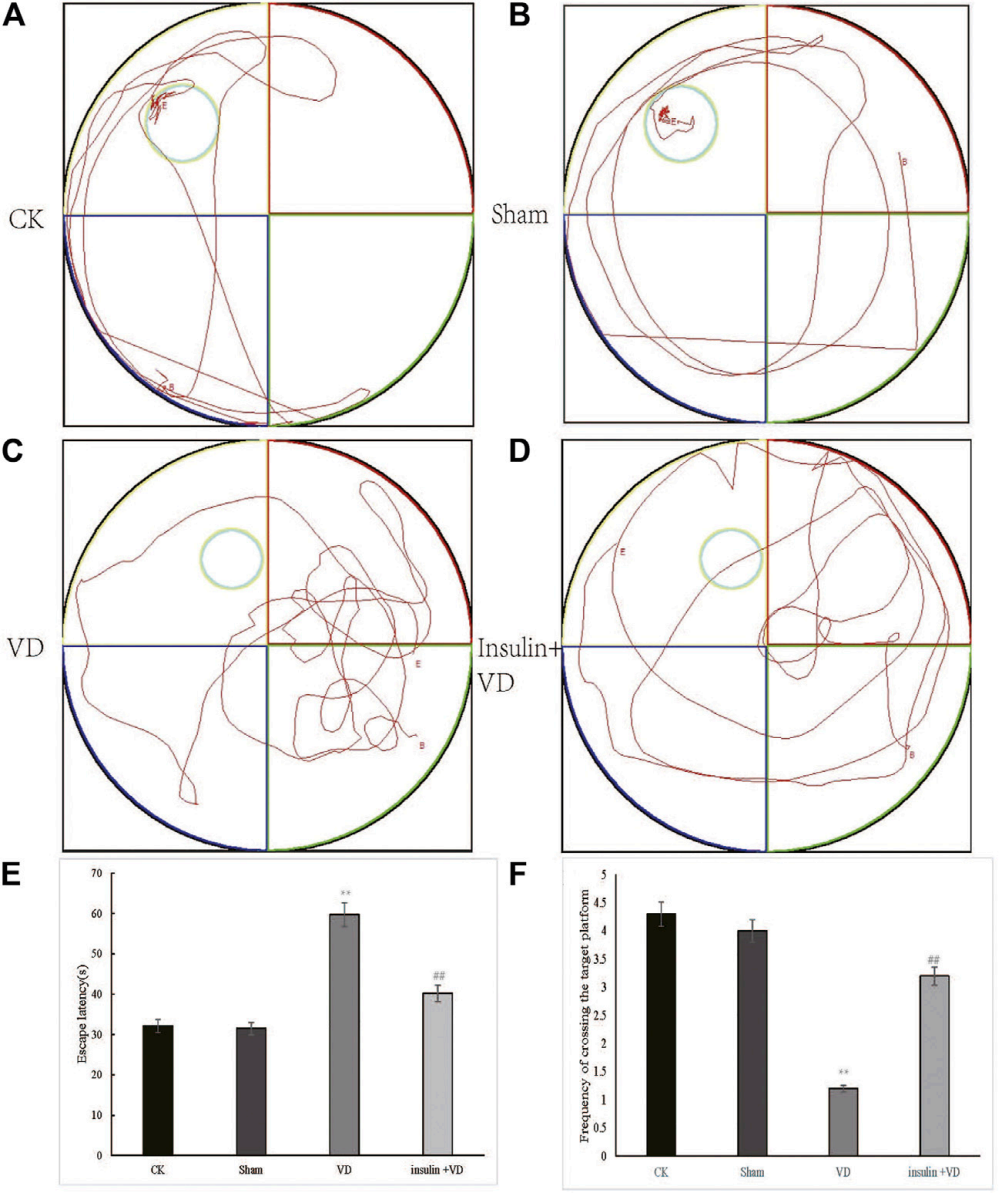
Testing of spatial learning and memory in CK, Sham, VD and Insulin + VD groups by Morris’s water maze **(A–D)**. Swimming trajectory of rats **(A)** CK; **(B)** Sham; **(C)** VD; **(D)** Insulin + VD **(E)**. Significant difference of escape latency between VD vs. CK(**p < 0.05) and Insulin + VD vs. VD (^##^ *p* < 0.05) were observed (*n* = 15/group) **(F)**. Significant difference of the frequency of crossing the target platform between VD vs. CK(**p < 0.05) and Insulin + VD vs. VD (^##^ *p* < 0.05) were detected. Data are expressed as the mean ± standard error of the mean (SEM) (*n* = 15/group). “**” indicating significant difference between VD and CK groups (*p* < 0.05). “^##^” indicating significant difference between Insulin + VD and VD groups (*p* < 0.05).

The escape latencies of the CK, Sham, VD, and INS + VD groups were 32.1 ± 3.81, 31.5 ± 2.34, 59.7 ± 4.53, and 40.2 ± 4.09, respectively. The mean escape latency in the Sham group not significantly changed compared with that of the CK group (*p >* 0.05). The mean escape latency in the VD group significantly increased compared to the CK group (*p* < 0.05). After treated with insulin, the mean escape latency in the INS + VD group significantly decreased compared with the VD group (*p* < 0.05). The number of hidden platform crossings in the CK, Sham, VD, and INS + VD groups were 4.3 ± 0.52, 4.0 ± 0.31, 1.2 ± 0.36, and 3.2 ± 0.37, respectively. The number of crossing the hidden platform crossings in the Sham group not significantly changed compared with that of the CK group (*p >* 0.05). The number of hidden platform crossings in the VD group significantly reduced compared to the CK group (*p* < 0.05). After treated with insulin, the number of hidden platform crossings in the INS + VD group significantly increased compared with that of the VD group (*p* < 0.05). (Figure 1). The results suggest that insulin treatment can significantly improve the learning and memory abilities of VD model rats.

### Insulin protects against VD-induced neural apoptosis and neuroinflammation

**FIGURE 2:**
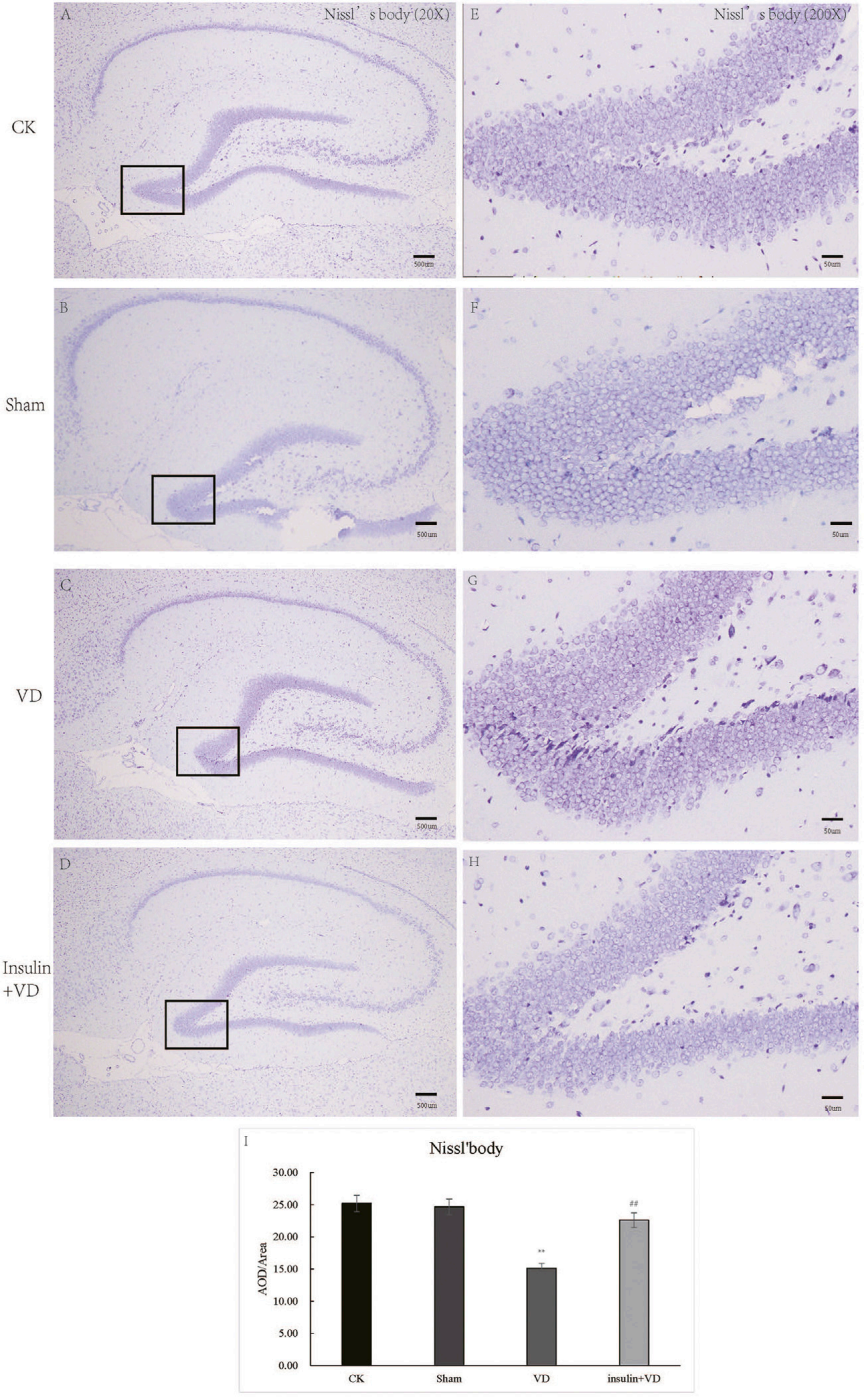
The Nissl staining of hippocampus in CK, Sham, VD and Insulin + VD groups **(A–H)**. The results of Nissl staining **(A–D)**. Nissl staining in the CK, Sham, VD and Insulin + VD groups (20X) **(E–H)**. Nissl staining in the CK, Sham, VD and Insulin + VD groups (200X). **(I)**. The comparison of the number of pyramidal neurons in the CK, Sham, VD and Insulin + VD groups. Data was expressed as the mean ± standard error of the mean (SEM). (*n* = 15/group in each group). “**” indicating significant difference between VD and CK groups (*p* < 0.05). “^##^” indicating significant difference between Insulin + VD and VD groups (*p* < 0.05).

As shown in Figures 2A–H, the neurons in the CA1 region of the hippocampus of rats in the CK, Sham, and INS + VD groups were regularly arranged, slightly nuclear stained, and clearly cytoplasmic stained. The arrangement of the neurons in the hippocampal CA1 region of rats in the VD group was disordered. The distance between the cells increased. The IOD of Nissl’s bodies in the Sham group (24.67 ± 5.2) was similar with that in the CK group (25.18 ± 5.1) (*p* > 0.05). The IOD of Nissl’s body in the VD group (15.12 ± 3.2) was significantly reduced compared to the CK group (25.18 ± 5.1) (*p* < 0.05). After treated with insulin, the IOD of Nissl’s body in the INS + VD group (22.59 ± 2.7) significantly increased compared to the VD group (15.12 ± 3.2) (*p* < 0.05) (Figure 2I).

**FIGURE 3:**
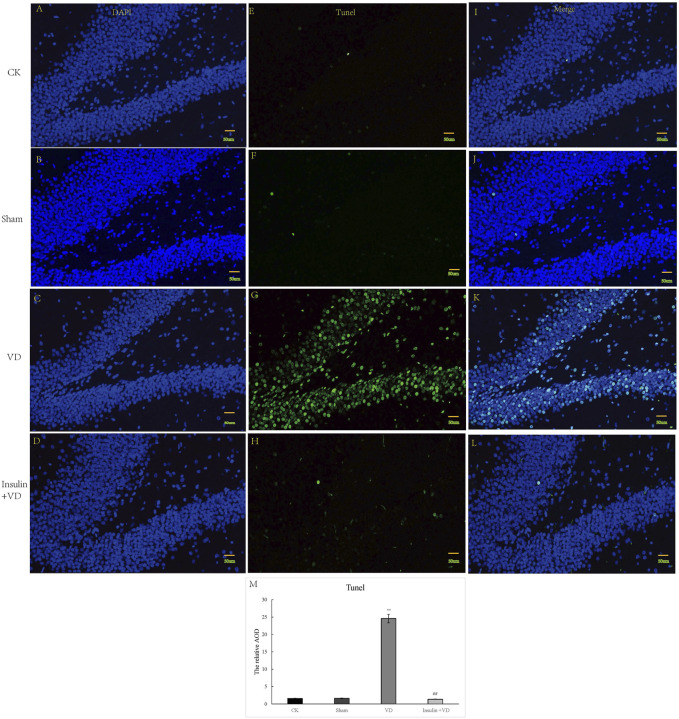
The tunnel staining of hippocampus in CK, Sham, VD and Insulin + VD groups **(A–M)**. The results of DAPI fluorescence in the CK, Sham, VD and Insulin + VD groups (200X) **(A–D)**. The tunnel fluorescence in the CK, Sham, VD and Insulin + VD groups (200X) **(E–H)**. The merged fluorescence in the CK, Sham, VD and Insulin + VD groups (200X) **(I-L)**. The comparison of tunneling staining in the CK, Sham, VD and Insulin + VD groups **(M)**. Data was expressed as the mean ± standard error of the mean (SEM). (*n* = 15/group in each group). “**” indicating significant difference between VD and CK groups (*p* < 0.05). “^##^” indicating significant difference between Insulin + VD and VD groups (*p* < 0.05).

The nuclei of tunnel-stained apoptotic neurons were green in color. Compared with the CK group, large number of apoptotic neurons were seen in clusters in the hippocampal CA1 region in the VD group. The proportion of TUNEL-staining positive cells in the hippocampal CA1 region was significantly increased in the VD group compared with that in the CK group (*p* < 0.05), indicating that ischemia might induce neuron apoptosis. And the proportion of TUNEL-staining positive cells in the hippocampal CA1 region exhibited significantly reduced in the INS + VD group compared to the VD group (*p* < 0.05), indicating that insulin might inhibit apoptosis (Figure 3).

**FIGURE 4:**
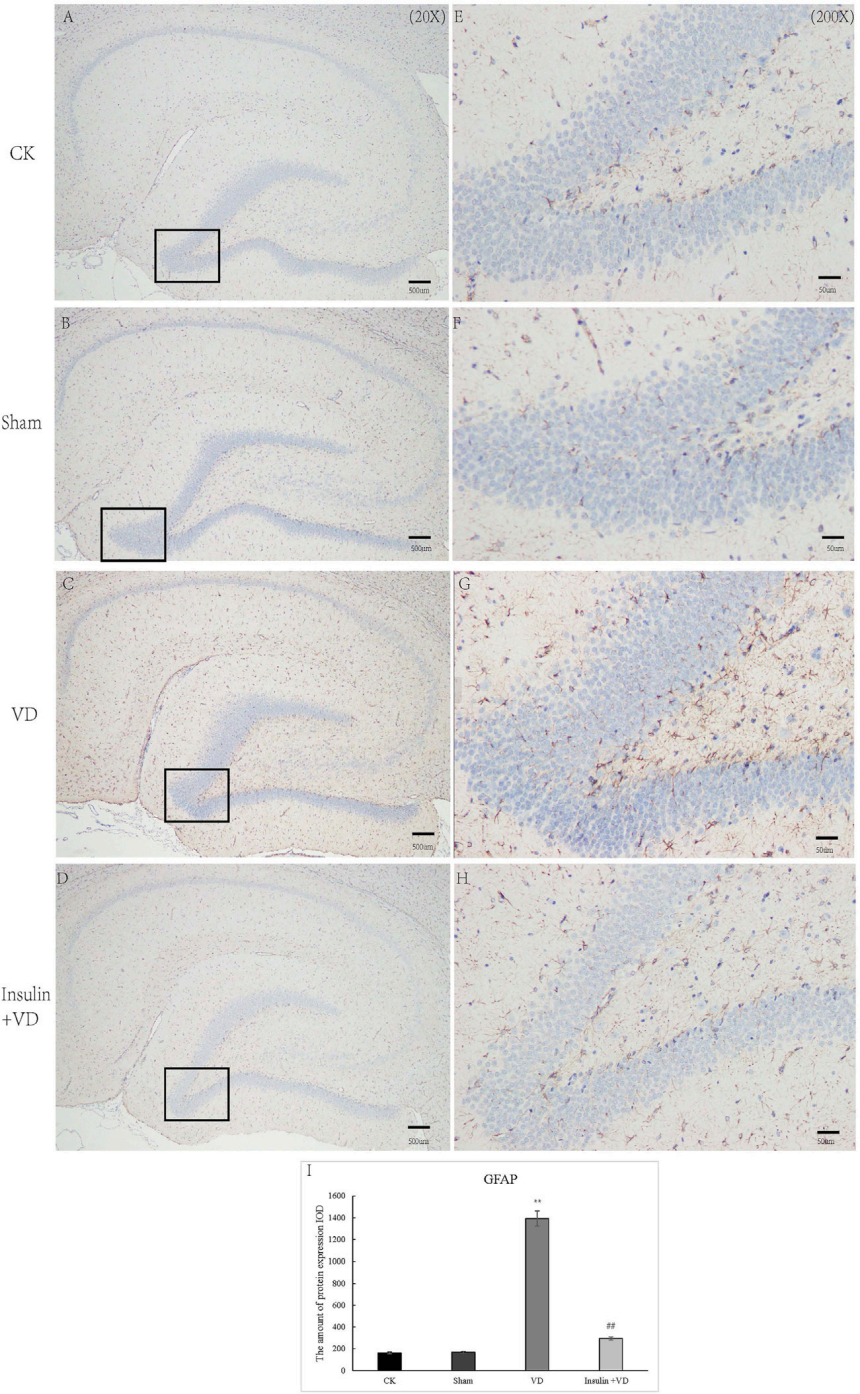
The distribution of GFAP immunolabeling across the brains in the CK, Sham, VD and Insulin + VD groups **(A–D)**. The distribution of GFAP immunolabeling in the CK, Sham, VD and Insulin + VD groups (20X) **(E–H)**. The distribution of GFAP immunolabeling in the CK, Sham, VD and Insulin + VD groups (200X) **(I)**. The comparison of GFAP immunolabeling in the CK, Sham, VD and Insulin + VD groups. Data was expressed as the mean ± standard error of the mean (SEM). (*n* = 15/group). “**” indicating significant difference between VD and CK groups (*p* < 0.05). “^##^” indicating significant difference between Insulin + VD and VD groups (*p* < 0.05).

As shown in Figure 4, the IODs of GFAP in CA1 region of hippocampus were similar in CK (161.9 ± 29.64) and Sham (168.2 ± 27.37) groups (*p* > 0.05). Compared to the CK group, GFAP IOD were significantly increased in VD group (1,391.9 ± 220.4) (*p* < 0.05). While, GFAP IOD was significantly decreased in INS + VD (293.9 ± 36.6) groups, when compared to the VD group (*p* < 0.05) (Figure 4).

### The effect of insulin on tau, p-tau, and Aβ_1-42_ activity

**FIGURE 5:**
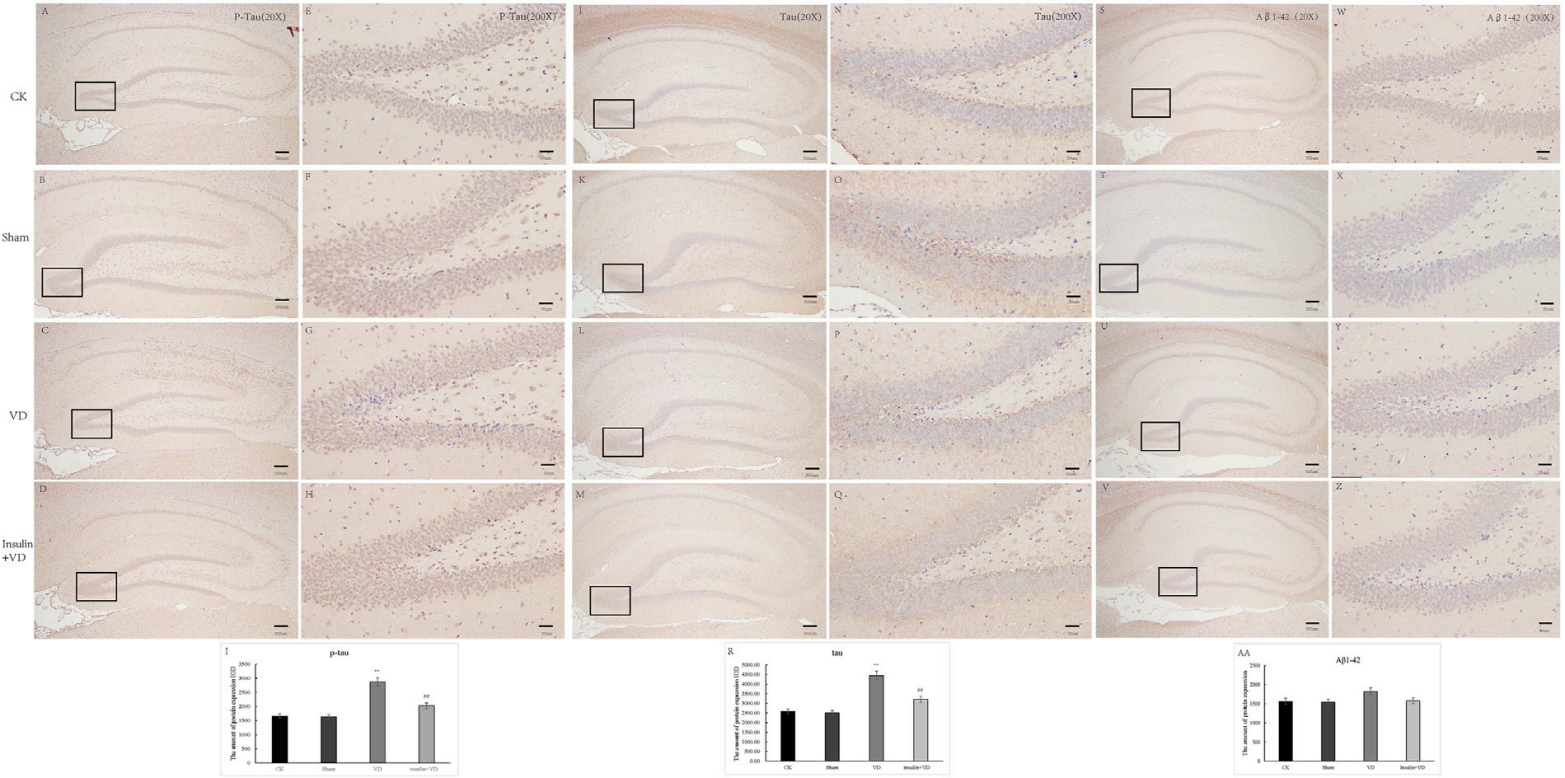
The distribution of p-Tau, Tau and Aβ_1-42_ immunolabeling across the brains in the CK, Sham, VD and Insulin + VD groups **(A–D)**: The distribution of p-Tau immunolabeling in the CK, VD and Insulin + VD groups (20X) **(E–H)**. The distribution of p-Tau immunolabeling in the CK, Sham, VD and Insulin + VD groups (200X) **(I)**. The comparison of p-Tau immunolabeling in the CK, Sham, VD and Insulin + VD groups **(J–M)**. The distribution of Tau immunolabeling in the CK, Sham, VD and Insulin + VD groups (20X) **(N–Q)**. The distribution of Tau immunolabeling in the CK, Sham, VD and Insulin + VD groups (200X) **(R)**. The comparison of Tau immunolabeling in the CK, Sham, VD and Insulin + VD groups **(S–V)**. The distribution of Tau immunolabeling in the CK, Sham, VD and Insulin + VD groups (20X) **(W–Z)**. The distribution of Tau immunolabeling in the CK, Sham, VD and Insulin + VD groups (200X) **(AA)**. The comparison of Tau immunolabeling in the CK, VD and Insulin + VD groups. Data was expressed as the mean ± standard error of the mean (SEM). (*n* = 15/group). “**” indicating significant difference between VD and CK groups (*p* < 0.05). “^##^” indicating significant difference between Insulin + VD and VD groups (*p* < 0.05).

There was no significant difference for IODs of p-tau and tau between CK and Sham groups (*p* > 0.05). The IODs of p-tau and tau were significantly increased in the VD group compared with that in the VD group (*p* < 0.05). And, compared to the VD group, a significantly lower IODs of p-tau and tau were detected in the INS + VD group (*p* < 0.05). In addition, there was no significant difference for IOD of Aβ_1-42_ between CK, Sham, VD, and INS + VD groups (*p* > 0.05) (Figure 5), indicating that insulin might reduce the level of p-tau and tau, but not Aβ_1-42_.

### Different expression genes (DEGs) screened

**FIGURE 6:**
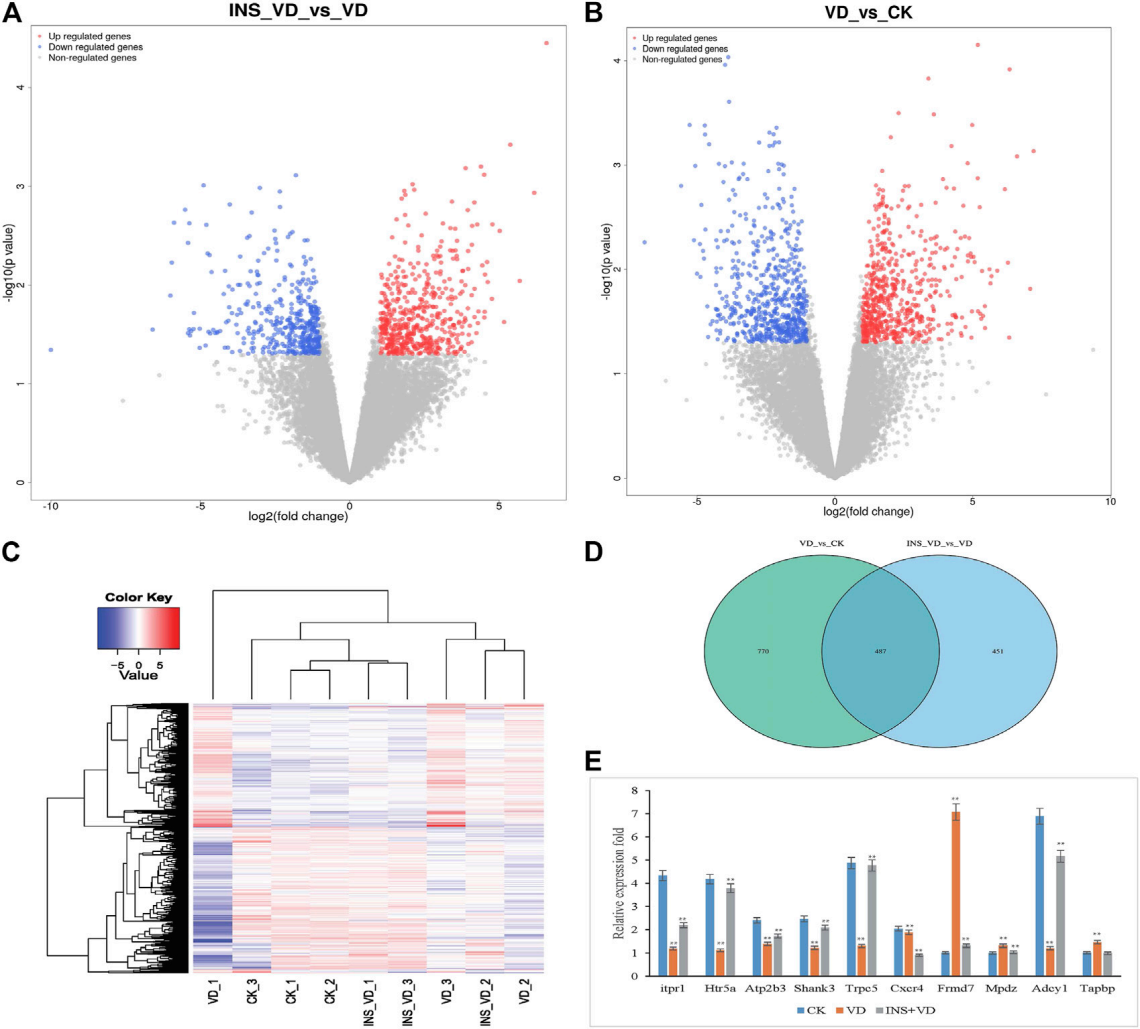
The differentially expressed genes (DEGs) among three comparison groups **(A)**. Volcano plot of DEGs (INS-VD vs. VD) **(B)**. Volcano plot of DEGs (VD vs. CK) **(C)**. Hierarchical clustering of DEGs **(D)**. Venn diagram of DEGs **(E)**. The gene expression was detected by qRT-PCR and normalized by GAPDH expression; **p < 0.05.

In total, 43.17 Gb of data were obtained from the transcriptome analysis. The effective data of each sample were distributed between 6.67 and 6.94G. Q30 bases were found in 92.46–93.38% of the data, and the average GC content was 50.55%. DEGs from the three-treatment comparison (CK vs. VD vs. INS + VD) with a |fold change| ≥ 1 and FDR < 0.05 (Figures 6A, B) were obtained. Hierarchical clustering of the DEGs based on the FPKM values showed the expression pattern of genes in the three-treatment comparison (CK vs. VD vs. INS + VD) (Figure 6C). There were 1,257 (VD vs. CK) and 938 (INS + VD vs. VD) DEGs in the two comparison groups, respectively (Figure 6D). The two comparisons (VD vs. CK and INS + VD vs. VD) shared 487 DEGs (Figure 6D). A total of 618 upregulated and 639 downregulated genes were identified between the VD and CK groups (Supplementary Table S2). A total of 521 upregulated and 417 downregulated genes were identified between the INS + VD and VD groups (Supplementary Table S3). The *Frmd7* and *Mpdz* genes were the most downregulated and upregulated mRNAs, with FCs of −6.59 and 1.03 respectively between the INS + VD and VD groups. The *Adcy1* and *Tapbp* genes were the most downregulated and upregulated mRNAs, with FCs of −6.89 and 1.00, respectively between the VD and CK groups.

### Validation of functional gene expression

To validate the reliability of the RNA-Seq gene expression data, the transcriptome levels of 10 DEGs (*Itpr1, Htr5a, Atp2b3, Shank3, Trpc, Cxcr4, Frmd7, Mpdz, Adcy1,* and *Tapbp*) were determined by qRT-PCR with three replicates (Figure 6E). Of these, the expression trends were consistent with those obtained in the RNA-Seq analysis. In addition, the RNA-Seq and qRT-PCR results demonstrated that the data could be used to assess the upregulation and downregulation of gene expression.

### Functional analysis of DEGs

**FIGURE 7:**
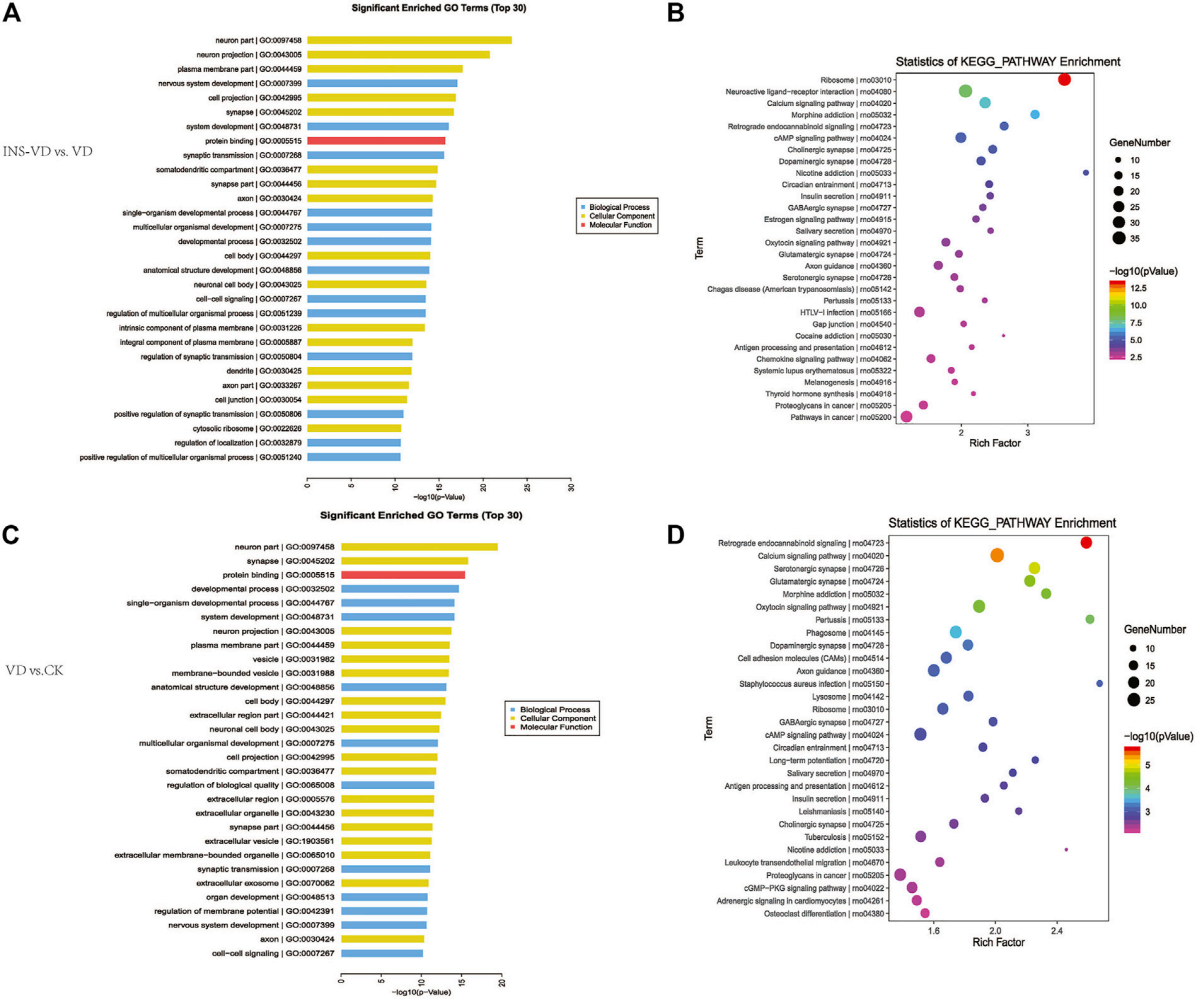
KEGG pathway and GO enrichment analysis of DEGs **(A)**. The top 30 most enriched GO categories and pathways were calculated and plotted (INS-VD vs. VD) **(B)**. The top 30 most enriched GO categories and pathways were calculated and plotted (VD vs. CK) **(C)**.Top30 KEGG pathways (INS-VD vs. VD) **(D)**. Top30 KEGG pathways (VD vs. CK).

Functional classification of DEGs was performed by GO analysis. In total, 61 GO terms were significantly enriched between the INS + VD and VD groups, most of which were enriched in neuron part (GO:0097458), neuron projection (GO:0043005), plasma membrane part (GO:0044459), and other functional categories (Figure 7A) (Supplementary Table S4). And 61 GO terms were significantly enriched between the VD and CK groups, most of which were enriched in neuron part (GO:0097458), synapse (GO:0045202), protein binding (GO:0005515), and other functional categories (Figure 7B) (Supplementary Table S5).

KEGG enrichment pathway analysis showed that ribosome (mo03010, 34 genes, adjusted *p* = 5.321E-11), neuroactive ligand-receptor interaction (rno04080, 36 genes, adjusted *p* = 8.33E-07), and calcium signaling pathway (rno04020, 26 genes, adjusted *p* = 7.563E-6) were the top 3 significantly altered pathways between the INS + VD and VD groups (Figure 7C). In addition, cAMP signaling pathway (rno04024, 24 genes, adjusted *p* = 0.0002), glutamatergic synapse (rno04724, 14 genes, adjusted *p* = 0.012), and axon guidance (rno04360, 18 genes, adjusted *p* = 0.013) were also found to be significantly altered pathways between the INS + VD and VD groups (Supplementary Table S6). Furthermore, calcium signaling pathway (rno04020, 28 genes, adjusted *p* = 0.002), oxytocin signaling pathway (rno04921, 23 genes, adjusted *p* = 0.01), cAMP signaling pathway (rno04024, 23 genes, adjusted *p* = 0.04) were the top 3 significantly altered pathways between the VD and CK groups (Figure 7D). And axon guidance (rno04360, 22 genes, adjusted *p* = 0.04), serotonergic synapse (rno04726, 21 genes, adjusted *p* = 0.004), and glutamatergic synapse (rno04724, 20 genes, adjusted *p* = 0.006) were found to be significantly altered pathways between the VD and CK groups (Supplementary Table S7). Clearly, several pathways including calcium signaling pathway (rno04020), cAMP signaling pathway (rno04024), axon guidance (rno04360), and glutamatergic synapse (rno04724) were significantly altered in the INS + VD vs. VD and VD vs. CK comparisons. These pathways may be involved in the pathogenesis of VD and insulin treatment.

### Metabolomic profiling

**FIGURE 8:**
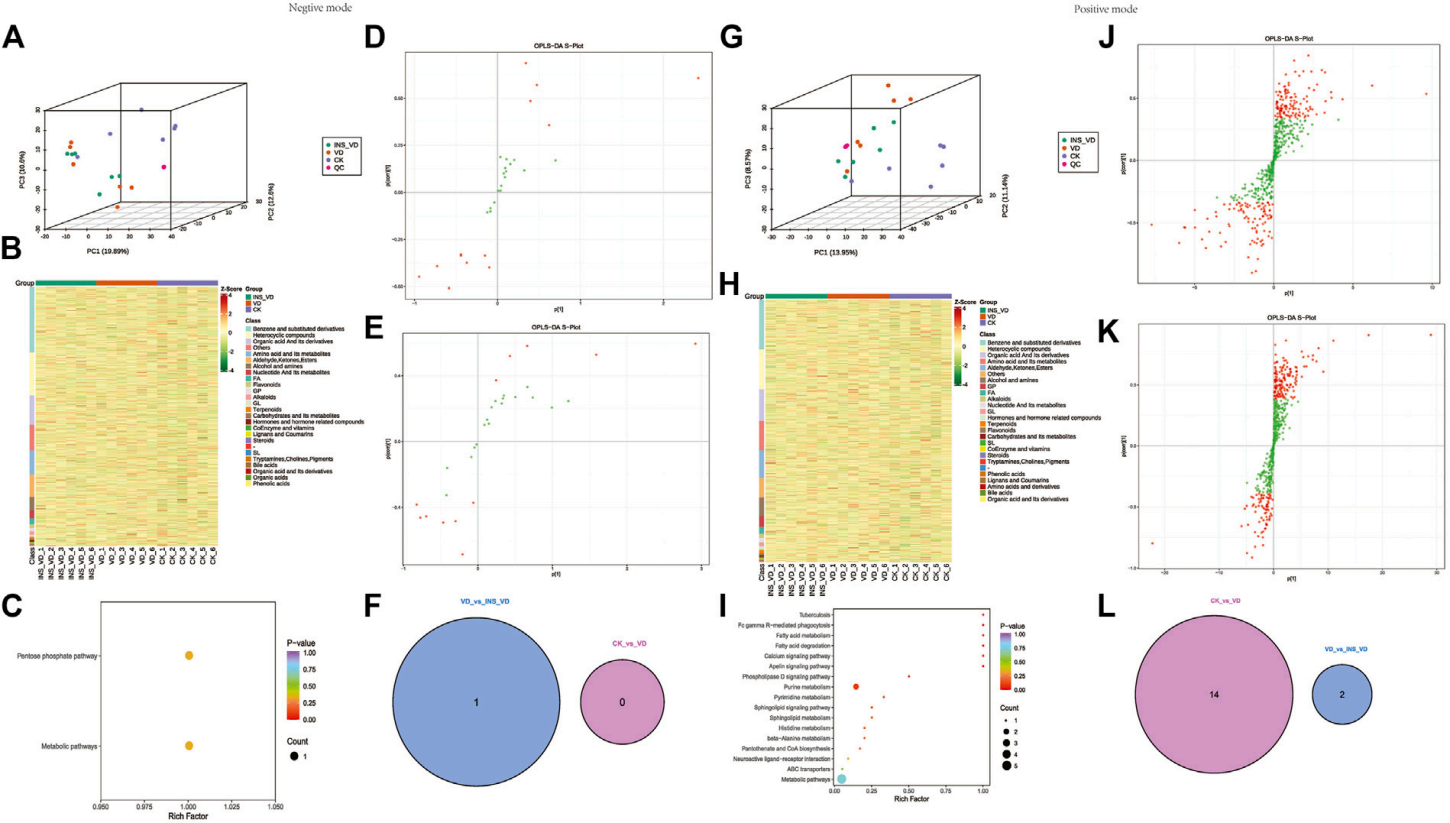
Hippocampus metabolic profiles between groups **(A,G)**. All samples’ principal component analysis (PCA) (at the metabolites level) **(A)**. Negative ion mode, **(G)**. Positive ion mode **(B,H)**. HCA-heatmap analysis comparison metabolites between CK, VD and Insulin + VD groups **(B)**. Negative ion mode, **(H)**. Positive ion mode **(D,E)**. OPLS-DA score plot between groups (Negative ion mode), **(D)**. INS-VD vs. VD, **(E)**. VD vs. CK **(J,K)**. OPLS-DA score plot between groups (Positive ion mode), **(J)**. INS-VD vs. VD, **(K)**. VD vs. CK **(C,I)**. Kyoto Encyclopedia of Genes and Genomes (KEGG) pathway terms enriched by metabolites of hippocampus between groups, **(C)**. Negative ion mode **(I)**. Positive ion mode **(F,L)**. Venn diagram of DEMs.

The PCA results showed that the metabolites were clearly separated among the three groups (Figures 8A, G). The OPLS-DA model score plots showed no outliers in our study and revealed a significant difference in metabolomics among the groups (Figures 8D, E, J, K). Hierarchical clustering was performed on all significantly different metabolites (*p* < 0.05) based on VIP values (Figures 8B, H). A total of 3,223 metabolites (1912 in positive mode and 1,311 in negative mode) were detected. Among them, 1 downregulated metabolite (2-keto-D-gluconic acid, VIP = 1.92, *p* = 0.046) was found between the INS + VD and VD groups in negative mode (Figure 8F). And 14 deregulated metabolites (2 downregulated (3-methoxybenzyl alcohol, VIP = 1.96, *p* = 0.018 and 13Z-docosenamide, VIP = 2.09, *p* = 0.013) and 12 upregulated) (Table 1) were detected in the INS + VD vs. VD, as well as VD vs. CK comparisons in positive mode (Figure 8L). In addition, KEGG pathway enrichment results showed that DEMs were mainly involved in pentose phosphate (ko00030) and metabolic pathways (ko01100) between the INS + VD and VD groups in negative mode (Figure 8C), and involved in purine metabolism (ko00230), metabolic (ko01100) and ABC transporters pathways (ko02010) between the VD and CK groups in positive mode (Figure 8I; Supplementary Table S8).

**TABLE 1:** DEMs between VD and CK groups in positive mode.

Compounds	Score	CAS	VIP	*p*-value	Fold_Change	Log_2_FC	Type
Tranexamic acid	0.7992	1197-18-8	2.065064	2.15E-05	0.114689	−3.1242	down
N-Methyl-alpha-aminoisobutyric acid	0.722	2566-34-9	2.273094	0.000398	0.498935	−1.00308	down
1-Pentadecanoyl-sn-glycero-3-phosphocholine	0.7115	108273-89-8	2.073048	0.00078	2.765342	1.467458	up
Glu-Glu	0.9049	3929-61-1	1.884533	0.001311	2.06077	1.043183	up
[(2R,3S,4R,5S)-5-acetamido-2,3,4-trihydroxy-6-oxohexyl] dihydrogen phosphate	0.9209	—	1.999141	0.002603	2.050003	1.035626	up
Uracil	0.6739	66-22-8	1.89839	0.004515	2.235804	1.160794	up
Allantoic Acid	0.8436	99-16-1	1.916647	0.010903	2.534159	1.341507	up
2′-Deoxyguanosine	0.7509	961-07-9	2.233093	0.024677	5.868143	2.552904	up
3-Methylhistidine	0.7142	368-16-1	1.794705	0.032918	2.68368	1.424213	up
gamma-Glutamylglutamate	0.508	1116-22-9	1.688524	0.037426	2.040798	1.029134	up
Carnitine C18:1	0.8862	—	1.829554	0.040743	2.736038	1.452088	up
L-Palmitoylcarnitine	0.7402	1935-18-8	1.684958	0.044127	2.030595	1.021902	up
Sphinganine 1-phosphate	0.6433	19794-97-9	2.351424	0.045046	80.37447	6.328665	up
Thioisonicotinamide	0.7162	2196-13-6	1.465142	0.047353	2.044889	1.032022	up

Abbreviations: DEMs: differentially expressed metabolites; VD: vascular dementia; CK: normal saline control; CAS: chemical abstracts service; VIP: variable importance in projection; FC: fold change.

### Integrative analysis of the transcriptome and metabolome

**FIGURE 9:**
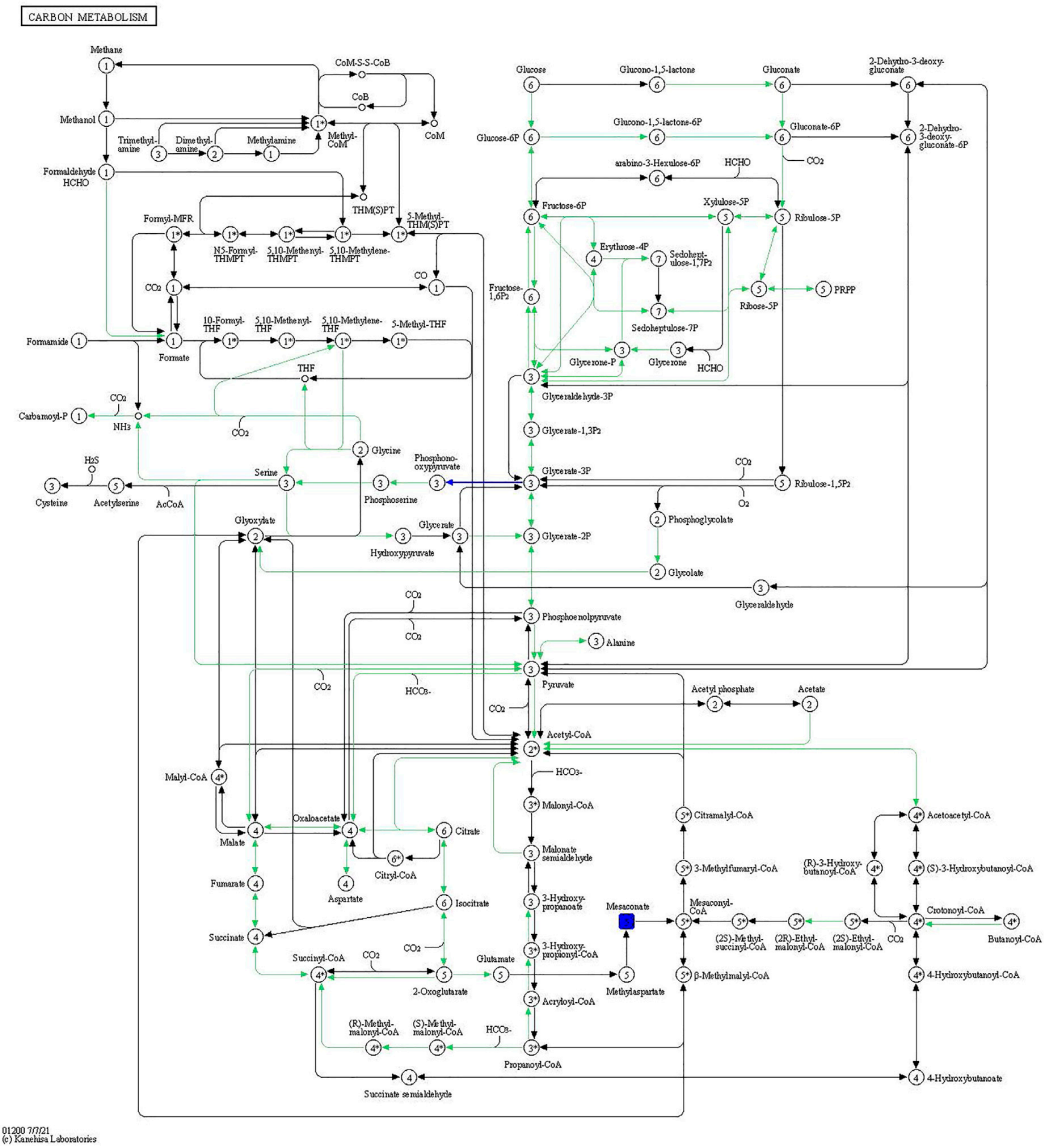
Carbon metabolism pathway. The blue rectangles are downregulated differentially expressed genes/proteins.

The compound-reaction-enzyme-gene networks in VD vs. CK and INS + VD vs. VD comparisons are shown in Supplementary Figures S1, S2. A total of 798 DEGs and 7 DEMs, as well as 1,097 DEGs and 18 DEMs were included in the individual INS + VD vs. VD and VD vs. CK comparisons. Pathway-level integration analysis showed that 27 pathways (Supplementary Table S9) and 1 pathway (carbon metabolism: rno01200) (Figure 9) were enriched upon integration of transcriptomics and metabolomics data in the VD vs. CK and INS + VD vs. VD comparisons respectively.

In addition, the DEGs related to the integrated pathways including Spp1, Frmd7, Asb16 were significantly upregulated, and Adcy1, Mfsd4a, and Mettl11b were significantly downregulated in the VD group compared to the CK group. The differentially accumulated metabolites: sphinganine 1-phosphate, 2′-Deoxyguanosine and 3-Methylhistidine were significantly upregulated, and L-Valine, Thiosulfate, and Tranexamic acid were significantly downregulated in VD group compared with the CK group.

Furthermore, the DEGs related to the integration pathways including Nkx2-1, Asb15, and Mettl11b were significantly upregulated and Ppm1j, Sfrp5, and Prss56 were significantly downregulated in the INS + VD group compared with the VD group. The differentially accumulated metabolites 3-Methoxybenzyl alcohol and 2-keto-D-gluconic acid were significantly upregulated and downregulated, respectively, in the INS + VD group, compared with the VD group.

## Discussion

Insulin and its receptors exist widely in the central nervous system, among which important signal transduction components are selectively distributed in the cognition-related hippocampus and other regions, with special biological effects, and play an important role in the growth and development of neurons, regulation of brain neurotransmitter transmission, utilization and uptake of glucose in the brain, learning and memory (D Ünal et al., 2012; Emra et al., 2019; Begg, 2019). The pharmacological mechanism of insulin administration via the nasal route may be that the hormone enters the brain directly via the olfactory and trigeminal nerves and exerts cognitive improvement by activating known or yet unidentified insulin receptors (Thorne et al., 2004; Renner et al., 2012; Lochhead et al., 2015; Lochhead et al., 2019). Since insulin plays an important role in cellular energy metabolism, proliferation, and growth activities, it is possible that the transnasal pathway of insulin may protect olfactory receptor cells in the nasal mucosa, improve olfactory mucosa regeneration, and slow neurodegenerative lesions of the olfactory pathway. The transnasal route of insulin administration used in the present study can effectively improve the cognitive function of VD rats, indicating that the transnasal route of insulin may be feasible for the treatment of VD.

Neuroinflammation is one of the pathogenic mechanisms of VD (Zha et al., 2021). The process of cerebral ischemic injury involves the inflammatory response, which is also involved in neurological impairment due to vascular diseases (Wang et al., 2018). Numerous experiments have shown that astrocytes can be damaged by ischemia and play an important role in ischemic injury of the CNS (Sun et al., 2006; Sugawara et al., 2009; Meeuwsen et al., 2010). The survival of astrocytes after brain ischemia affects neuronal survival and synaptic remodeling and is involved in learning memory processes and cognitive impairment (Oh et al., 2019; Woodburn et al., 2021). After brain ischemia, astrocytes are stimulated and induce the production of many inflammatory mediators, including the transcription factor NF-KB, tumor necrosis factor (TNF), interleukins (IL), and interferons (IFN) (Sun et al., 2017; Hyunlee et al., 2021). The various bioactive factors, in turn, exacerbate the activation of glial cells through various feedback pathways, triggering an inflammatory response and causing brain damage and ultimately cognitive dysfunction (Pamies et al., 2021). In addition, we previously reported that Taohong Siwu decoction (TSD) could protect against VD through mediating the inflammatory response, oxidative stress, and apoptosis pathways (He et al., 2023). We also found that triptolide suppressed the activation and proliferation of microglial cells and astrocytes in the hippocampus of APP/PS1 double transgenic AD model mice (Li et al., 2016). Furthermore, studies of insulin treatment in animals and clinical trials of intranasal insulin administration in individuals with AD have indicated that intranasal insulin not only improves memory but also reduces inflammation and white matter hyperintensities (Reger et al., 2006; Craft et al., 2012; Chang et al., 2021; Kellar et al., 2021). In the present study, the number of GFAP immunopositive cells in the CA1 hippocampal region of VD model rats was found to be significantly higher than that in the CK group. The number of GFAP immunopositive cells in the INS + VD group was significantly lower than that in the VD group. This result suggests that intranasal insulin may inhibit the inflammatory response of astrocytes in VD model rats.

Tau protein is a relatively sensitive marker of neuronal ischemic damage (Sun et al., 2007). Tau protein levels in CSF may reflect neuronal degeneration and damage (Wang et al., 2016). A multicenter clinical study found histologic changes such as neurogenic fiber tangles and senile plaque formation in the brain tissue of VD patients (Victorof, 1995). Some studies have confirmed that upregulated expression of tau and p-tau is associated with VD pathogenesis and may be involved in postischemic hippocampal neuronal damage as one of the mechanisms constituting VD (Liang et al., 2011). In the present study, we found that tau and p-tau protein expression levels were significantly lower in the CK and INS + VD groups than those in the VD group, which further confirmed the important role of tau and p-tau in the pathogenesis of VD. In addition to phosphorylation modifications, Tau has O-GlcNAc glycosylation modifications. Since they modify the same or neighboring serine and threonine hydroxyl groups of the same protein, there may be competitive inhibitors (Deng et al., 2008). O-GlcNAc glycosylation of proteins is regulated by glucose metabolism, and glucose uptake and metabolism in the brain are mainly molecularly regulated through the insulin signaling pathway, which indicates that phosphorylation of Tau is affected by abnormal insulin signaling pathway leading to impaired glucose metabolism in the brain (Liu et al., 2011; Stanciu et al., 2020). In addition, the insulin signaling pathway is a collection of autophosphorylation processes catalyzed by tyrosine kinases that are widely present in mammals (Takeda et al., 2011). The impairment of the sinsulin signaling pathway caused by abnormal insulin levels in the brain may alter the activity of important kinases that regulate Tau protein phosphorylation such as phosphatidylinositol-3 protein kinase (P13K) and mitogen-activated protein kinase (MAPK), and thus may affect Tau protein phosphorylation and its function (Stern et al., 2014), which in turn may lead to neurodegeneration.

Furthermore, it has been suggested that chronic cerebral perfusion deficits can cause increased Aβ expression in the brain, and accelerated Aβ deposition affects brain energy metabolism and subsequently cognitive function (Mukaetova-Ladinska et al., 2015). In the present study, we found no statistically significant differences in Aβ_1-42_ expression levels between the VD, CK, and INS + VD groups. This finding is inconsistent with the results of several previous studies (Jia et al., 2005; Paradowski et al., 2007). Notably, Xu et al. (2010) found that there was a trend of increased, but not significant, Aβ_1-40_ and Aβ_1-42_ levels in VD patients. This result suggested that Aβ_1-40_ and Aβ_1-42_ were involved in the formation and development of VD, but are not the primarily mechanism. The above evidence supports our findings to some extent. However, the role of Aβ, tau and p-tau in the pathogenesis of VD needs to be further investigated.

Another pathogenic link of cognitive dysfunction in VD is the apoptosis of hippocampal cells after ischemia (Gao et al., 2003). After the onset of cerebral ischemia, protein synthesis in the brain is severely impaired, while new specific proteins are synthesized to regulate apoptosis (Yan et al., 2022). These active regulatory genes include the *Bcl-2* and *P53* gene families (Khaksari et al., 2015). The nucleus and mitochondria are key determinants of apoptosis, and mitochondrial damage generates large amounts of ROS, which promotes a specific translocation mechanism of Bcl-2 and Bax that inhibits the production of oxygen radicals in mitochondria, and the released cytochrome C binds to apoptosis enzyme activator and then to aspartate-containing cysteine-specific proteases-9 (caspase-9), the bound complex of which activates caspase-3 and exacerbates apoptosis and necrosis by disrupting intranuclear DNA repair enzymes (Qiu et al., 2007; Hussar, 2022). Several studies have shown that intracerebroventricular injection of insulin after whole brain ischemia upregulates Bcl-2 and Bcl-x protein expression in the hippocampal CA1 region and reduces neuronal apoptosis (Li et al., 2003; Lin et al., 2007; Huang et al., 2011). In the present study, we found that the neuronal arrangement in the hippocampal CA1 region of rats in the VD group was disorganized, the number of Nissl’s bodies significantly reduced, and the rate of neuronal apoptosis significantly increased. After treatment with insulin, the rate of neuronal apoptosis was significantly reduced. The above results suggested that insulin could reduce neuronal apoptosis in the hippocampal CA1 region and improve cognitive and memory functions in VD model rats.

Apoptosis of neuronal cells caused by activation of EAA receptors is also an important cause of VD (Cao et al., 2016). When brain injury occurs, such as ischemia and hypoxia, glutamate-based excitatory neurotransmitters are released in excess and EAA receptors are activated. Large amounts of glutamate accumulate in the synaptic cleft and act on N-methyl-D-aspartate (NMDA) receptors in the postsynaptic membrane. Postsynaptic neurons are in a sustained depolarized state, with large Ca^2+^ inward currents and elevated intracellular free Ca^2+^, leading to Ca^2+^ overload, which mediates a series of intracellular Ca^2+^-dependent biochemical reactions, that cause dyfunction of intersynaptic information transmission and ultimately lead to neuronal damage and impaired learning and memory functions (Tanović and Alfaro, 2006). Li X. G. et al. (2001) found that glutamate induces neuronal Ca^2+^ influx and cell apoptosis, and that Ca^2+^ influx plays an important role in glutamate toxicity leading to apoptosis. *In vitro* studies have shown that glutamate affects calcium signaling in rat hippocampal neurons through multiple pathways, and activation of NMDA and α-amino-3-hydroxy-5-methyl-4-isoxazolepropionicacid (AMPA) receptors is one of the important mechanisms (Tan et al., 2006). In the present study, transcriptome analysis revealed that most of the differentially expressed genes between the CK, VD, and VD + INS groups were enriched in calcium signaling pathway (rno04020), cAMP signaling pathway (rno04024), axon guidance (rno04360), and Ca^2+^ signaling pathway (rno04360). Genes related to Ca^2+^ signaling (Itpr1), glutamatergic synaptic (Shank3), and axon guidance (Trpc5 and Cxcr4) were significantly differentially expressed among the three groups. These results suggested that insulin may affect glutamate release and Ca^2+^ overload by altering the expression of Ca^2+^ signaling pathway, glutamatergic synaptic pathway, and axon guidance pathway-related genes, modulating hippocampal synaptic plasticity, reducing brain damage, and improving cognitive function in VD model rats.

Moreover, we detected 1 and 16 DEMs in the INS + VD vs. VD and VD vs. CK comparisons in negative and positive modes, enriched in 2 and 17 KEGG pathways. Although, no common metabolite was detected between VD vs. CK and INS + VD vs. VD comparisons, we found that the DEMs between the INS + VD and VD groups in negative mode and the VD and CK groups in positive mode were enriched in the metabolic pathway (ko01100). The metabolic pathway included metabolites of 2-keto-D-gluconic acid, 3-methylhistidine, sphinganine 1-phosphate, 2′-deoxyguanosine, allantoic acid, and uracil, which indicate that these metabolites may act as epigenetically, play an important role and are involved in the pathogenesis of VD and can be used as novel therapeutic target compounds for VD therapy.

In addition, the DEGs related to the integrated pathways suggested that the Spp1, Frmd7, Asb16, Adcy1, Mfsd4a, and Mettl11b genes may regulate the synthesis and function of metabolites such as sphinganine 1-phosphate, 2′-deoxyguanosine and 3-methylhistidine, L-valine, thiosulfate, and tranexamic acid. The Nkx2-1, Asb15, Mettl11b, Ppm1j, Sfrp5, and Prss56 genes may regulate the synthesis and function of metabolites such as 3-methoxybenzyl alcohol and 2-keto-D-gluconic acid. However, little is known about the function of these genes and the regulation of these metabolites. It is necessary to identify them with further experiments.

## Conclusion

In summary, our study demonstrated that intranasal spray insulin could effectively reduce tau and p-tau levels, inhibit astrocyte inflammation, reduce neuron apoptosis, and improve cognitive function in VD model rats. The combined transcriptome and metabolome analyses suggested that the potential mechanism of insulin against VD involved genes were enriched in the calcium signaling pathway, cAMP signaling pathway, axon guidance, and glutamatergic synapse. Moreover, metabolites involved in metabolic pathway might participate in the protective effects of insulin against VD.

## Data Availability

The datasets presented in this study can be obtained in online GEO (accession number: GSE228174, https://www.ncbi.nlm.nih.gov/geo/query/acc.cgi?acc=GSE228174).

## Supplementary material

SUPPLEMENTARY FIGURE S1 Compound-Reaction-Enzyme-Gene network (INS-VD vs. VD). Notes: The hexagonal nodes are metabolites (the annotated differential metabolites are shown in dark red), the rhombic nodes represent the reaction, and the squares represent the enzymes in the reaction, the circular nodes represent the enzyme’s coding genes (the annotated differential genes are shown in dark blue).

SUPPLEMENTARY FIGURE S2 Compound-Reaction-Enzyme-Gene network (VD vs. CK). Notes: The hexagonal nodes are metabolites (the annotated differential metabolites are shown in dark red), the rhombic nodes represent the reaction, and the squares represent the enzymes in the reaction, the circular nodes represent the enzyme’s coding genes (the annotated differential genes are shown in dark blue).
