# Supplementary material for: Integrated transcriptome and metabolome analysis to investigate the mechanism of intranasal insulin treatment in a rat model of vascular dementia
Source: Front Pharmacol. 2023 May 15;14:1182803. doi: 10.3389/fphar.2023.1182803 (PMC10225696; doi:10.3389/fphar.2023.1182803)
Supplement: Supplementary file 1 [file Table1.docx]

Table S1. The primers used for qRT-PCR detection of selected genes.

| Gene name | Forward 5’-3’ | Reverse 3’-5’ |
| --- | --- | --- |
| Itpr1 | TGTCCTCGAC TCTAGTGACC GG | GGAGCTCGTG CCCAATGAGG AC |
| Agtr1a | GAGA ATGTTCTGTT AAGCTGGG | CTCCCTAAA GTGCAGTCTT AGA |
| Atp2b3 | CTTCGGTCCT CAGCCAGCTC CATG | TTCCAGGCC TGGGAGCCCA CTC |
| VAV1 | GCACTGCT CATCTGTAAG CGCCG | GCCATTTCTG CGGCTCAACC CT |
| Grin3b | ATCCGGGGAG GCCCTAACCG CAG | CCTGGGTTCG TCCCCAGCTC CG |
| Shank3 | CCTACTCCCT GTAGACCTGA CTC | TCTCTGTTAC CTCGGGACTG GC |
| Trpc5 | GTTCATGGGA TAAGTGGGAG ATG | CTATTTCAGT AATACCTACA TG |
| Cxcr4 | TCGATCTTGG AGTAACGCGC CTG | GATGGTGGTG TTCCAGTTCC AGC |
| Frmd7 | GCAGGCAAGG AAAGCATTGT TAC | GTCCCAATCC CCACCTCGTG CTG |
| Mpdz | GGAAAGGTAT CATTGTCAGG CTGG | GG AGTTTATCAT GGTAGCGAAT C |
| Adcy1 | CAAGGGCCAA GGCTCAGAGC AAG | GT AGCCCAGCTC TCTTACACTT G |
| Tapbp | GCT TTACCTCCTC CTGCCAGTTA TC | CTC CTGCCTTGGT TTTTTGAGAC |
