# Supplementary material for: Integrated transcriptome and metabolome analysis to investigate the mechanism of intranasal insulin treatment in a rat model of vascular dementia
Source: Front Pharmacol. 2023 May 15;14:1182803. doi: 10.3389/fphar.2023.1182803 (PMC10225696; doi:10.3389/fphar.2023.1182803)
Supplement: Supplementary file 2 [file Table8.docx]

Table S8 KEGG pathways of DEMs between VD and CK groups.

| Kegg_pathway | ko_ID | Sig_compound | compound | Sig_compound_all | compound_all | IndexList | CIDList |
| --- | --- | --- | --- | --- | --- | --- | --- |
| Purine metabolism | ko00230 | 2 | 14 | 6 | 149 | MW0105582;MW0103343 | C00499+C00330 |
| Metabolic pathways | ko01100 | 5 | 124 | 6 | 149 | MEDP0034;MW0063621;MW0103343;MW0105582;MEDP0178 | C01152+C06124+C00330+C00499+C00106 |
| ABC transporters | ko02010 | 1 | 19 | 6 | 149 | MW0103343 | C00330 |
| Pyrimidine metabolism | ko00240 | 1 | 3 | 6 | 149 | MEDP0178 | C00106 |
| beta-Alanine metabolism | ko00410 | 1 | 5 | 6 | 149 | MEDP0178 | C00106 |
| Pantothenate and CoA biosynthesis | ko00770 | 1 | 6 | 6 | 149 | MEDP0178 | C00106 |
| Histidine metabolism | ko00340 | 1 | 5 | 6 | 149 | MEDP0034 | C01152 |
| Sphingolipid metabolism | ko00600 | 1 | 4 | 6 | 149 | MW0063621 | C06124 |
| Calcium signaling pathway | ko04020 | 1 | 1 | 6 | 149 | MW0063621 | C06124 |
| Sphingolipid signaling pathway | ko04071 | 1 | 4 | 6 | 149 | MW0063621 | C06124 |
| Phospholipase D signaling pathway | ko04072 | 1 | 2 | 6 | 149 | MW0063621 | C06124 |
| Neuroactive ligand-receptor interaction | ko04080 | 1 | 11 | 6 | 149 | MW0063621 | C06124 |
| Apelin signaling pathway | ko04371 | 1 | 1 | 6 | 149 | MW0063621 | C06124 |
| Fc gamma R-mediated phagocytosis | ko04666 | 1 | 1 | 6 | 149 | MW0063621 | C06124 |
| Tuberculosis | ko05152 | 1 | 1 | 6 | 149 | MW0063621 | C06124 |
| Fatty acid degradation | ko00071 | 1 | 1 | 6 | 149 | MW0054414 | C02990 |
| Fatty acid metabolism | ko01212 | 1 | 1 | 6 | 149 | MW0054414 | C02990 |

Abbreviations: KEGG; Kyoto Encyclopedia of Genes and Genomes; DEMs: differentially expressed metabolites; VD: vascular dementia; CK: normal saline control
