# Supplementary material for: Integrated transcriptome and metabolome analysis to investigate the mechanism of intranasal insulin treatment in a rat model of vascular dementia
Source: Front Pharmacol. 2023 May 15;14:1182803. doi: 10.3389/fphar.2023.1182803 (PMC10225696; doi:10.3389/fphar.2023.1182803)
Supplement: Supplementary file 3 [file Table7.docx]

Table S7 Top30 KEGG pathways of DEGs between VD and CK groups

| #Term | ID | Input number | P-Value | Corrected P-Value |
| --- | --- | --- | --- | --- |
| Retrograde endocannabinoid signaling | rno04723 | 21 | 2.06E-06 | 0.002164 |
| Calcium signaling pathway | rno04020 | 28 | 3.92E-06 | 0.002164 |
| Serotonergic synapse | rno04726 | 21 | 1.37E-05 | 0.003785 |
| Glutamatergic synapse | rno04724 | 20 | 2.58E-05 | 0.005705 |
| Morphine addiction | rno05032 | 17 | 6.11E-05 | 0.010019 |
| Oxytocin signaling pathway | rno04921 | 23 | 6.35E-05 | 0.010019 |
| Pertussis | rno05133 | 14 | 9.21E-05 | 0.011308 |
| Phagosome | rno04145 | 22 | 0.000264 | 0.020854 |
| Dopaminergic synapse | rno04728 | 18 | 0.000576 | 0.036523 |
| Cell adhesion molecules (CAMs) | rno04514 | 20 | 0.000739 | 0.036523 |
| Axon guidance | rno04360 | 22 | 0.000755 | 0.036523 |
| Staphylococcus aureus infection | rno05150 | 10 | 0.000769 | 0.036523 |
| Lysosome | rno04142 | 17 | 0.000794 | 0.036523 |
| Ribosome | rno03010 | 20 | 0.000859 | 0.036523 |
| GABAergic synapse | rno04727 | 14 | 0.00109 | 0.039927 |
| cAMP signaling pathway | rno04024 | 23 | 0.001152 | 0.039927 |
| Circadian entrainment | rno04713 | 14 | 0.001454 | 0.042371 |
| Long-term potentiation | rno04720 | 11 | 0.001457 | 0.042371 |
| Salivary secretion | rno04970 | 12 | 0.001504 | 0.04261 |
| Antigen processing and presentation | rno04612 | 12 | 0.001858 | 0.047737 |
| Insulin secretion | rno04911 | 13 | 0.002006 | 0.05037 |
| Leishmaniasis | rno05140 | 11 | 0.002052 | 0.050391 |
| Cholinergic synapse | rno04725 | 15 | 0.002504 | 0.058865 |
| Tuberculosis | rno05152 | 19 | 0.002912 | 0.063086 |
| Nicotine addiction | rno05033 | 8 | 0.003974 | 0.080236 |
| Leukocyte transendothelial migration | rno04670 | 15 | 0.00398 | 0.080236 |
| Proteoglycans in cancer | rno05205 | 22 | 0.004028 | 0.080236 |
| cGMP-PKG signaling pathway | rno04022 | 19 | 0.004238 | 0.080745 |
| Adrenergic signaling in cardiomyocytes | rno04261 | 17 | 0.005459 | 0.098892 |
| Osteoclast differentiation | rno04380 | 15 | 0.00653 | 0.112739 |

Abbreviations: KEGG; Kyoto Encyclopedia of Genes and Genomes; DEGs: differentially expressed genes; VD: vascular dementia; CK: normal saline control
