# Supplementary material for: Integrated transcriptome and metabolome analysis to investigate the mechanism of intranasal insulin treatment in a rat model of vascular dementia
Source: Front Pharmacol. 2023 May 15;14:1182803. doi: 10.3389/fphar.2023.1182803 (PMC10225696; doi:10.3389/fphar.2023.1182803)
Supplement: Supplementary file 4 [file Table9.docx]

Table S9 KEGG pathways enriched upon integration of both transcriptomics and metabolomics data in VD vs. CK comparison

| KEGG_CID | Name | FC | Up/Down | log2FC | VIP | P | Formula |
| --- | --- | --- | --- | --- | --- | --- | --- |
| C00330 | 2'-Deoxyguanosine | 5.868143 | up | 2.552904 | 2.233093 | 0.024677 | C10H13N5O4 |
| C05282 | gamma-Glutamylglutamate | 2.040798 | up | 1.029134 | 1.688524 | 0.037426 | C10H16N2O7 |
| C02814 | 1,2,4-Benzenetriol | 0.609207 | down | -0.715 | 2.137685 | 0.004946 | C6H6O3 |
| C00183 | L-Valine | 0.563701 | down | -0.827 | 2.367349 | 0.002526 | C5H11NO2 |
| C12535 | Tranexamic acid | 0.114689 | down | -3.1242 | 2.065064 | 2.15E-05 | C8H15NO2 |
| C00106 | Uracil | 2.235804 | up | 1.160794 | 1.89839 | 0.004515 | C4H4N2O2 |
| C05689 | Se-methylselenocysteine | 1.724088 | up | 0.785834 | 1.505165 | 0.044175 | C4H9NO2Se |
| C00499 | Allantoic Acid | 2.534159 | up | 1.341507 | 1.916647 | 0.010903 | C4H8N4O4 |
| C01425 | Glu-Glu | 2.06077 | up | 1.043183 | 1.884533 | 0.001311 | C10H16N2O7 |
| C01152 | 3-Methylhistidine | 2.68368 | up | 1.424213 | 1.794705 | 0.032918 | C7H13N3O3 |
| C00979 | O-Acetyl-L-serine | 1.672322 | up | 0.741852 | 1.658038 | 0.017886 | C5H9NO4 |
| C02710 | N-Acetyl-L-Leucine | 1.613732 | up | 0.690401 | 1.559408 | 0.042136 | C8H15NO3 |
| C05529 | Thiosulfate | 0.50508 | down | -0.98542 | 1.699958 | 0.025563 | H2O3S2 |
| C01996 | Acetylcholine | 0.654253 | down | -0.61208 | 1.912325 | 0.007532 | C7H16NO2+ |
| C00864 | Pantothenic acid | 1.584835 | up | 0.664332 | 1.631961 | 0.034317 | C9H17NO5 |
| C06124 | Sphinganine 1-phosphate | 80.37447 | up | 6.328665 | 2.351424 | 0.045046 | C18H40NO5P |
| C12151 | Mycolactone | 0.647559 | down | -0.62692 | 1.828504 | 0.026395 | C44H70O9 |
| C02990 | L-Palmitoylcarnitine | 2.030595 | up | 1.021902 | 1.684958 | 0.044127 | C23H45NO4 |

Abbreviations: KEGG; Kyoto Encyclopedia of Genes and Genomes; DEMs: differentially expressed metabolites; VD: vascular dementia; CK: normal saline control
