# Supplementary material for: Integrated transcriptome and metabolome analysis to investigate the mechanism of intranasal insulin treatment in a rat model of vascular dementia
Source: Front Pharmacol. 2023 May 15;14:1182803. doi: 10.3389/fphar.2023.1182803 (PMC10225696; doi:10.3389/fphar.2023.1182803)
Supplement: Supplementary file 5 [file Table2.docx]

Table S2 DEGs between VD and CK groups

| ID | logFC | P value | regulation | pos_info | ref_pos_info | symbol |
| --- | --- | --- | --- | --- | --- | --- |
| ENSRNOG00000001492 | -3.86483492390165 | 9.24E-05 | down | 1:+:78025995-78060111 | 1:+:78025995-78060111 | Slc8a2 |
| ENSRNOG00000028417 | -3.97978368251711 | 0.00011 | down | 10:-:86280892-86282693 | 10:-:86280892-86282693 | Neurod2 |
| ENSRNOG00000024923 | 2.310654 | 0.000318 | up | 3:+:154043873-154046330 | 3:+:154043873-154046330 | Nnat |
| ENSRNOG00000032328 | -3.835253943481 | 0.000247 | down | 17:+:12762752-12763952 | 17:+:12762752-12763952 | Diras2 |
| ENSRNOG00000046834 | 3.393088 | 0.000148 | up | 9:+:9721105-9747167 | 9:+:9721105-9747167 | C3 |
| ENSRNOG00000022162 | 3.590396 | 0.000327 | up | 3:-:13182024-13525983 | 3:-:13182024-13525983 | Pbx3 |
| ENSRNOG00000005018 | -2.37094228671975 | 0.000488 | down | 3:+:51687809-51822250 | 3:+:51687809-51822250 | Scn2a |
| ENSRNOG00000057044 | -2.1148084828915 | 0.000438 | down | 3:-:12034413-12155098 | 3:-:12034413-12155098 | Garnl3 |
| ENSRNOG00000008431 | -2.22155872421273 | 0.000506 | down | 5:-:62276100-62621737 | 5:-:62276100-62621737 | Gabbr2 |
| ENSRNOG00000018087 | 2.029379 | 0.000541 | up | 17:+:80882666-80891212 | 17:+:80882666-80891212 | Vim |
| ENSRNOG00000011160 | -2.0220170223687 | 0.000604 | down | 1:-:136664204-136842247 | 1:-:136664204-136842247 | AC126641.1 |
| ENSRNOG00000003741 | -2.7463716863436 | 0.000608 | down | 10:-:108682638-108691367 | 10:-:108682638-108691367 | Nptx1 |
| ENSRNOG00000021231 | -2.17017037540595 | 0.000606 | down | 3:-:123175860-123179644 | 3:-:123175860-123179644 | Lzts3 |
| ENSRNOG00000005985 | -2.24509470283894 | 0.000648 | down | 15:+:344360-1047956 | 15:+:344360-1047956 | Kcnma1 |
| ENSRNOG00000002626 | -2.37334426636014 | 0.000653 | down | 10:+:80790168-81296363 | 10:+:80790168-81296363 | Car10 |
| ENSRNOG00000012061 | -1.85043523582745 | 0.001011 | down | 1:+:192233910-192574831 | 1:+:192233910-192574831 | Prkcb |
| ENSRNOG00000049361 | -3.73796223186171 | 0.000942 | down | 10:+:54126486-54236476 | 10:+:54126486-54236476 | Gas7 |
| ENSRNOG00000004560 | -1.92567038337807 | 0.000984 | down | 3:-:1740024-1924827 | 3:-:1740024-1924827 | Cacna1b |
| ENSRNOG00000052296 | -2.02096904529497 | 0.001094 | down | 7:+:130474279-130534679 | 7:+:130474279-130534679 | Shank3 |
| ENSRNOG00000056643 | -2.05097202593508 | 0.000969 | down | 19:+:6046665-6426216 | 19:+:6046665-6426216 | Cdh8 |
| ENSRNOG00000019328 | 1.718864 | 0.001136 | up | 2:-:200484246-200513564 | 2:-:200484246-200513564 | Phgdh |
| ENSRNOG00000002549 | 6.336452 | 0.000121 | up | 13:-:37479758-37492680 | 13:-:37479758-37492680 | Htr5b |
| ENSRNOG00000005726 | -3.3258260378397 | 0.001216 | down | 4:-:16454904-17058921 | 4:-:16454904-17058921 | Pclo |
| ENSRNOG00000001647 | -1.85661635587152 | 0.001222 | down | 11:+:36075709-36092495 | 11:+:36075709-36092495 | Ets2 |
| ENSRNOG00000024435 | -3.30735335439431 | 0.000967 | down | 12:+:28381982-29268073 | 12:+:28381982-29268073 | Galnt17 |
| ENSRNOG00000011000 | -3.46112064610952 | 0.001321 | down | 9:-:28442229-28973246 | 9:-:28442229-28973246 | Rims1 |
| ENSRNOG00000018305 | -2.41280398462443 | 0.001142 | down | 18:+:59748444-59755226 | 18:+:59748444-59755226 | St8sia3 |
| ENSRNOG00000017123 | 1.491127 | 0.001566 | up | 3:+:114087287-114093309 | 3:+:114087287-114093309 | B2m |
| ENSRNOG00000016957 | 2.539851 | 0.001585 | up | 9:+:80118029-80144789 | 9:+:80118029-80144789 | Igfbp2 |
| ENSRNOG00000058842 | -2.01596162302537 | 0.001683 | down | 1:+:219964429-220005068 | 1:+:219964429-220005068 | Sptbn2 |
| ENSRNOG00000000814 | 2.678101 | 0.001582 | up | 20:+:40769586-40773349 | 20:+:40769586-40773349 | Fabp7 |
| ENSRNOG00000015550 | 2.473463 | 0.001756 | up | 3:-:2686123-2689084 | 3:-:2686123-2689084 | Ptgds |
| ENSRNOG00000011132 | -3.48787170134429 | 0.001361 | down | 3:-:94064234-94182714 | 3:-:94064234-94182714 | LOC100362814 |
| ENSRNOG00000026466 | -1.46395598826099 | 0.001687 | down | 20:+:25990304-26163656 | 20:+:25990304-26163656 | Lrrtm3 |
| ENSRNOG00000012062 | 1.747409 | 0.001742 | up | 6:-:108467410-108488330 | 6:-:108467410-108488330 | Npc2 |
| ENSRNOG00000019374 | 1.618796 | 0.001712 | up | 8:-:62405715-62424303 | 8:-:62405715-62424303 | Csk |
| ENSRNOG00000030714 | -2.5344806863124 | 0.001901 | down | 8:-:116873721-116965396 | 8:-:116873721-116965396 | Bsn |
| ENSRNOG00000007104 | -3.61600556116093 | 0.001919 | down | 4:+:140247313-140580748 | 4:+:140247313-140580748 | Itpr1 |
| ENSRNOG00000043103 | -3.17162489678664 | 0.001363 | down | 5:-:74000227-74029238 | 5:-:74000227-74029238 | Frrs1l |
| ENSRNOG00000020060 | 1.569919 | 0.001888 | up | 1:-:100808241-100810522 | 1:-:100808241-100810522 | Atf5 |
| ENSRNOG00000000768 | 5.184851 | 7.09E-05 | up | 20:-:1876173-1897814 | 20:-:1876173-1897814 | Ubd |
| ENSRNOG00000027264 | -1.57265878881021 | 0.002008 | down | 1:-:226297016-226353611 | 1:-:226297016-226353611 | Dagla |
| ENSRNOG00000014064 | 1.748012 | 0.002004 | up | 8:+:97439161-97458287 | 8:+:97439161-97458287 | Ctsh |
| ENSRNOG00000011425 | -1.88348319447814 | 0.002107 | down | 5:-:74248996-74368186 | 5:-:74248996-74368186 | Ptpn3 |
| ENSRNOG00000020843 | 1.743954 | 0.0023 | up | 1:-:101448346-101449829 | 1:-:101448346-101449829 | LOC100360087 |
| ENSRNOG00000014684 | 1.731353 | 0.002126 | up | 2:-:189840403-189856090 | 2:-:189840403-189856090 | Npr1 |
| ENSRNOG00000028580 | -1.8474210643865 | 0.002252 | down | 19:-:58583632-58735173 | 19:-:58583632-58735173 | Pcnx2 |
| ENSRNOG00000001309 | -2.0726126769623 | 0.002363 | down | 12:+:39253409-39302601 | 12:+:39253409-39302601 | Camkk2 |
| ENSRNOG00000002919 | 1.597821 | 0.002495 | up | 10:-:90990762-90999506 | 10:-:90990762-90999506 | Gfap |
| ENSRNOG00000010031 | 1.671575 | 0.002327 | up | 10:+:65767053-65771038 | 10:+:65767053-65771038 | Vtn |
| ENSRNOG00000017445 | 1.460394 | 0.002454 | up | 17:+:31441630-31482759 | 17:+:31441630-31482759 | Tubb2b |
| ENSRNOG00000009325 | 1.609953 | 0.002374 | up | 5:+:154269118-154286544 | 5:+:154269118-154286544 | Fuca1 |
| ENSRNOG00000043451 | 7.206301 | 0.000734 | up | 14:-:6673686-6679901 | 14:-:6673686-6679901 | Spp1 |
| ENSRNOG00000002210 | 1.701413 | 0.00241 | up | 14:+:7073445-7104566 | 14:+:7073445-7104566 | Hsd17b11 |
| ENSRNOG00000008223 | -2.25435536408474 | 0.002555 | down | 5:+:49311030-49333056 | 5:+:49311030-49333056 | Cnr1 |
| ENSRNOG00000023708 | 1.871454 | 0.002468 | up | 4:+:78458625-78462423 | 4:+:78458625-78462423 | Tmem176a |
| ENSRNOG00000014152 | -1.55916776549361 | 0.002425 | down | 3:-:120023917-120087136 | 3:-:120023917-120087136 | Kcnip3 |
| ENSRNOG00000016897 | 1.87855 | 0.00247 | up | 1:-:141097695-141111400 | 1:-:141097695-141111400 | Rlbp1 |
| ENSRNOG00000014723 | -2.44939502657853 | 0.00207 | down | 19:-:55441572-55510460 | 19:-:55441572-55510460 | Cbfa2t3 |
| ENSRNOG00000003463 | 1.496384 | 0.002669 | up | 10:-:46570996-46593009 | 10:-:46570996-46593009 | Srebf1 |
| ENSRNOG00000016398 | -1.51453806613949 | 0.00279 | down | 5:+:166533181-166601684 | 5:+:166533181-166601684 | Clstn1 |
| ENSRNOG00000006472 | 2.050043 | 0.002599 | up | 6:+:99433550-99436289 | 6:+:99433550-99436289 | Hspa2 |
| ENSRNOG00000025415 | -5.26476082258524 | 0.000413 | down | 13:-:81988926-82006005 | 13:-:81988926-82006005 | Mettl11b |
| ENSRNOG00000010362 | 2.299295 | 0.002467 | up | 8:+:75687100-75723594 | 8:+:75687100-75723594 | Anxa2 |
| ENSRNOG00000047211 | -1.98613270344223 | 0.002576 | down | 15:-:48601266-48670257 | 15:-:48601266-48670257 | Fzd3 |
| ENSRNOG00000014819 | 1.801003 | 0.002978 | up | 10:-:88257976-88266210 | 10:-:88257976-88266210 | Hap1 |
| ENSRNOG00000038980 | -2.02600154137379 | 0.00242 | down | 3:+:35679750-35707410 | 3:+:35679750-35707410 | Lypd6 |
| ENSRNOG00000009263 | 2.966708 | 0.00307 | up | 6:+:127327959-127334428 | 6:+:127327959-127334428 | Ifi27 |
| ENSRNOG00000036794 | 6.606627 | 0.000823 | up | 10:+:90217924-90226537 | 10:+:90217924-90226537 | Asb16 |
| ENSRNOG00000023588 | -4.71553648224526 | 0.000417 | down | 2:+:23289374-23370477 | 2:+:23289374-23370477 | Dmgdh |
| ENSRNOG00000002863 | -3.43657809342348 | 0.003028 | down | 13:-:71906702-72367980 | 13:-:71906702-72367980 | Cacna1e |
| ENSRNOG00000020684 | 1.593829 | 0.00327 | up | 10:-:89366899-89374516 | 10:-:89366899-89374516 | Vat1 |
| ENSRNOG00000051456 | -4.70711879969172 | 0.000508 | down | 2:+:254022079-254039326 | 2:+:254022079-254039326 | AABR07013729.1 |
| ENSRNOG00000005770 | 4.980895 | 0.000413 | up | 6:+:55812747-55817066 | 6:+:55812747-55817066 | Sostdc1 |
| ENSRNOG00000059776 | -3.98344184878747 | 0.001022 | down | AABR07024382.1:+:99089-150196 | AABR07024382.1:+:99089-150196 | Tnks2 |
| ENSRNOG00000004283 | -1.20099956281502 | 0.003456 | down | 10:-:57291192-57309298 | 10:-:57291192-57309298 | Camta2 |
| ENSRNOG00000002217 | 4.230167 | 0.000655 | up | 14:+:10692764-10714524 | 14:+:10692764-10714524 | Plac8 |
| ENSRNOG00000053583 | 1.239488 | 0.003481 | up | 1:+:198192773-198198975 | 1:+:198192773-198198975 | Mapk3 |
| ENSRNOG00000002753 | -1.48054657586318 | 0.003496 | down | 10:+:90731148-90749217 | 10:+:90731148-90749217 | Adam11 |
| ENSRNOG00000007003 | -4.55943791290309 | 0.00063 | down | 7:+:123308041-123361391 | 7:+:123308041-123361391 | Mei1 |
| ENSRNOG00000017072 | -2.75514782176482 | 0.002564 | down | 9:-:92503597-92530938 | 9:-:92503597-92530938 | Slc16a14 |
| ENSRNOG00000005450 | -2.51966245659795 | 0.002509 | down | 10:-:31028697-31041626 | 10:-:31028697-31041626 | Lsm11 |
| ENSRNOG00000017610 | -1.76657778310181 | 0.00355 | down | 18:+:60392376-60719720 | 18:+:60392376-60719720 | Nedd4l |
| ENSRNOG00000049071 | -1.89816371973036 | 0.003341 | down | 15:+:52148379-52160294 | 15:+:52148379-52160294 | Phyhip |
| ENSRNOG00000061230 | -1.32339393633062 | 0.003679 | down | X:+:156909913-156928057 | X:+:156909913-156928057 | L1cam |
| ENSRNOG00000031090 | 2.437185 | 0.002152 | up | 20:-:4694472-4896970 | 20:-:4694472-4896970 | RT1-CE7 |
| ENSRNOG00000000842 | 1.778211 | 0.003583 | up | 20:+:5049496-5052585 | 20:+:5049496-5052585 | Ddah2 |
| ENSRNOG00000050206 | -3.2338912989071 | 0.002933 | down | 1:+:217151166-217592763 | 1:+:217151166-217592763 | Shank2 |
| ENSRNOG00000029682 | 1.810503 | 0.003138 | up | 20:+:5040337-5049166 | 20:+:5040337-5049166 | Clic1 |
| ENSRNOG00000007645 | -1.61280901749283 | 0.003759 | down | 13:-:90703036-90710287 | 13:-:90703036-90710287 | Kcnj9 |
| ENSRNOG00000045771 | -2.93963754582093 | 0.003742 | down | 4:+:70252366-70330803 | 4:+:70252366-70330803 | Chl1 |
| ENSRNOG00000004423 | -3.72251321747723 | 0.0038 | down | 13:+:95589668-95591236 | 13:+:95589668-95591236 | Zbtb18 |
| ENSRNOG00000012881 | 2.699194 | 0.00249 | up | 4:-:10323607-10329241 | 4:-:10323607-10329241 | Fgl2 |
| ENSRNOG00000059746 | -5.06197295299351 | 0.001015 | down | X:-:129223138-129336004 | X:-:129223138-129336004 | AABR07041411.1 |
| ENSRNOG00000013190 | 1.945106 | 0.003626 | up | 1:+:53174879-53192048 | 1:+:53174879-53192048 | Rnaset2 |
| ENSRNOG00000020277 | -1.64556476747058 | 0.00404 | down | 10:+:89089646-89103614 | 10:+:89089646-89103614 | Cntnap1 |
| ENSRNOG00000007636 | -2.53585059150755 | 0.003995 | down | 4:-:50326442-50860756 | 4:-:50326442-50860756 | Cadps2 |
| ENSRNOG00000000777 | 1.586496 | 0.003834 | up | 20:+:3176107-3179818 | 20:+:3176107-3179818 | RT1-S3 |
| ENSRNOG00000008943 | 1.408899 | 0.004053 | up | 5:-:17056419-17061837 | 5:-:17056419-17061837 | Penk |
| ENSRNOG00000012876 | 1.954818 | 0.003545 | up | 4:+:153874852-153912155 | 4:+:153874852-153912155 | Slc6a13 |
| ENSRNOG00000013920 | -3.21832384080788 | 0.003271 | down | 18:-:31812109-32207801 | 18:-:31812109-32207801 | Arhgap26 |
| ENSRNOG00000001851 | -1.7287673397448 | 0.004054 | down | 4:+:182483194-182565436 | 4:+:182483194-182565436 | Far2 |
| ENSRNOG00000028404 | -1.78160433654165 | 0.004182 | down | 10:+:86303727-86312762 | 10:+:86303727-86312762 | Ppp1r1b |
| ENSRNOG00000024657 | -5.57382101827651 | 0.001578 | down | 13:-:48807720-48848997 | 13:-:48807720-48848997 | Mfsd4a |
| ENSRNOG00000037476 | -1.79862830109719 | 0.004266 | down | 12:-:52106506-52187164 | 12:-:52106506-52187164 | Galnt9 |
| ENSRNOG00000012053 | 1.793671 | 0.004276 | up | 2:+:189922996-189928848 | 2:+:189922996-189928848 | S100a16 |
| ENSRNOG00000026136 | 1.949891 | 0.003314 | up | 18:+:44716226-44779914 | 18:+:44716226-44779914 | Tnfaip8 |
| ENSRNOG00000051204 | -1.33239310060405 | 0.00431 | down | 11:+:33925498-34027435 | 11:+:33925498-34027435 | Dop1b |
| ENSRNOG00000000443 | 2.655155 | 0.002582 | up | 20:-:4302347-4508214 | 20:-:4302347-4508214 | LOC103689965 |
| ENSRNOG00000004496 | -1.2885761940556 | 0.004499 | down | 6:+:42180894-42289908 | 6:+:42180894-42289908 | Rock2 |
| ENSRNOG00000016225 | 1.878583 | 0.004176 | up | 17:+:15749978-15800401 | 17:+:15749978-15800401 | Fgd3 |
| ENSRNOG00000018237 | 1.99957 | 0.004486 | up | 1:-:219291679-219294147 | 1:-:219291679-219294147 | Gstp1 |
| ENSRNOG00000032618 | -4.20405603315118 | 0.00097 | down | 8:+:116686601-116700132 | 8:+:116686601-116700132 | Mst1r |
| ENSRNOG00000046021 | 1.793367 | 0.004396 | up | X:-:15425078-15428518 | X:-:15425078-15428518 | LOC108348172 |
| ENSRNOG00000018960 | -2.22533974144523 | 0.00459 | down | 1:-:41608418-42077535 | 1:-:41608418-42077535 | AABR07001382.1 |
| ENSRNOG00000014940 | 6.164748 | 0.0017 | up | 1:-:261442108-261446570 | 1:-:261442108-261446570 | Sfrp5 |
| ENSRNOG00000024899 | 4.81611 | 0.00096 | up | 14:-:15253125-15258207 | 14:-:15253125-15258207 | Cxcl13 |
| ENSRNOG00000032660 | -1.69878170948306 | 0.004528 | down | 2:-:258792839-258997145 | 2:-:258792839-258997145 | Adgrl2 |
| ENSRNOG00000002004 | -2.91095351533325 | 0.003827 | down | 14:-:13054771-13058172 | 14:-:13054771-13058172 | Prdm8 |
| ENSRNOG00000014961 | 1.72307 | 0.004411 | up | 5:-:161947137-161981441 | 5:-:161947137-161981441 | Pdpn |
| ENSRNOG00000007957 | -1.93525613503788 | 0.004915 | down | 10:+:75055020-75076434 | 10:+:75055020-75076434 | Tspoap1 |
| ENSRNOG00000011475 | -2.44371339534591 | 0.004996 | down | 10:-:85460059-85517683 | 10:-:85460059-85517683 | Srcin1 |
| ENSRNOG00000008061 | -1.37136470227542 | 0.005042 | down | 7:+:25039335-25113664 | 7:+:25039335-25113664 | Nuak1 |
| ENSRNOG00000003209 | 3.611898 | 0.004981 | up | 13:-:89542378-89565813 | 13:-:89542378-89565813 | Pcp4l1 |
| ENSRNOG00000000815 | 1.54731 | 0.00473 | up | 20:+:40778927-40800868 | 20:+:40778927-40800868 | Smpdl3a |
| ENSRNOG00000031700 | -2.31938488218059 | 0.004886 | down | 1:+:15412603-15613746 | 1:+:15412603-15613746 | Map3k5 |
| ENSRNOG00000013729 | -3.04898337950239 | 0.00446 | down | 4:-:65834035-65962539 | 4:-:65834035-65962539 | RGD1306271 |
| ENSRNOG00000060687 | -2.28596534362078 | 0.005133 | down | 3:+:139695028-139833967 | 3:+:139695028-139833967 | Slc24a3 |
| ENSRNOG00000013000 | 1.276626 | 0.005368 | up | 4:-:176701983-176720012 | 4:-:176701983-176720012 | Ldhb |
| ENSRNOG00000021962 | 2.367736 | 0.004788 | up | 10:+:90550147-90552057 | 10:+:90550147-90552057 | Fzd2 |
| ENSRNOG00000008475 | -2.32934978481766 | 0.004317 | down | 5:-:40032855-40237591 | 5:-:40032855-40237591 | Fut9 |
| ENSRNOG00000016244 | -2.54539036841338 | 0.005407 | down | 1:+:177048655-177173995 | 1:+:177048655-177173995 | Mical2 |
| ENSRNOG00000017093 | 1.774023 | 0.004336 | up | 17:+:30965942-30994410 | 17:+:30965942-30994410 | Pxdc1 |
| ENSRNOG00000004737 | 1.550891 | 0.005207 | up | 13:+:90116843-90140371 | 13:+:90116843-90140371 | Cd48 |
| ENSRNOG00000051261 | -4.04107387690634 | 0.001449 | down | 13:+:77264239-77485250 | 13:+:77264239-77485250 | AABR07021536.1 |
| ENSRNOG00000002468 | -2.95579846360767 | 0.005469 | down | 13:+:77485113-77678437 | 13:+:77485113-77678437 | Tnr |
| ENSRNOG00000008465 | 1.709623 | 0.005603 | up | 4:-:78450724-78458179 | 4:-:78450724-78458179 | Tmem176b |
| ENSRNOG00000023603 | -2.64029964041101 | 0.004788 | down | 9:+:41096835-41099189 | 9:+:41096835-41099189 | Amer3 |
| ENSRNOG00000006663 | -1.70935580771625 | 0.0055 | down | 8:+:48406260-48430885 | 8:+:48406260-48430885 | Usp2 |
| ENSRNOG00000001959 | 3.131557 | 0.002399 | up | 11:-:37891156-37914983 | 11:-:37891156-37914983 | Mx1 |
| ENSRNOG00000025287 | 5.18087 | 0.001333 | up | 10:+:64174931-64178591 | 10:+:64174931-64178591 | RGD1565611 |
| ENSRNOG00000013720 | 2.077097 | 0.005659 | up | 14:+:86101277-86111306 | 14:+:86101277-86111306 | Aebp1 |
| ENSRNOG00000018735 | 3.48272 | 0.005068 | up | 18:+:56071478-56080849 | 18:+:56071478-56080849 | Cd74 |
| ENSRNOG00000028630 | -2.93056605113841 | 0.003393 | down | 12:-:44536293-44911147 | 12:-:44536293-44911147 | Ksr2 |
| ENSRNOG00000005309 | -2.85646448521002 | 0.005802 | down | 7:+:142575672-142684114 | 7:+:142575672-142684114 | Scn8a |
| ENSRNOG00000002045 | 1.559059 | 0.00584 | up | 14:-:14364008-14426437 | 14:-:14364008-14426437 | Anxa3 |
| ENSRNOG00000009882 | -1.40435799658243 | 0.005882 | down | 2:+:241909832-242184854 | 2:+:241909832-242184854 | Ppp3ca |
| ENSRNOG00000004554 | 1.811605 | 0.005623 | up | 7:+:38742051-38782323 | 7:+:38742051-38782323 | Dcn |
| ENSRNOG00000028566 | 1.643088 | 0.0057 | up | 6:+:137323713-137331231 | 6:+:137323713-137331231 | Pld4 |
| ENSRNOG00000003512 | -1.65170250905356 | 0.005984 | down | 10:-:27310725-27366665 | 10:-:27310725-27366665 | Gabra1 |
| ENSRNOG00000009872 | 1.334914 | 0.006019 | up | 4:+:7355574-7387253 | 4:+:7355574-7387253 | Kcnh2 |
| ENSRNOG00000010065 | -4.83887831192504 | 0.002402 | down | 15:-:60803098-60959769 | 15:-:60803098-60959769 | Dgkh |
| ENSRNOG00000004201 | -2.78725528498357 | 0.005948 | down | 7:+:78092037-78594138 | 7:+:78092037-78594138 | Rims2 |
| ENSRNOG00000047393 | 4.319526 | 0.001673 | up | 7:+:143629455-143633131 | 7:+:143629455-143633131 | Krt18 |
| ENSRNOG00000054277 | 3.921607 | 0.001363 | up | 2:+:58724855-58747843 | 2:+:58724855-58747843 | Capsl |
| ENSRNOG00000015068 | 1.754546 | 0.006125 | up | 5:+:58151985-58159072 | 5:+:58151985-58159072 | Il11ra1 |
| ENSRNOG00000013884 | -2.20985864151708 | 0.0063 | down | 16:+:23447366-23781604 | 16:+:23447366-23781604 | Psd3 |
| ENSRNOG00000012749 | 1.849006 | 0.006286 | up | 5:-:155246447-155252003 | 5:-:155246447-155252003 | C1qb |
| ENSRNOG00000033688 | -1.21334605547638 | 0.006421 | down | 6:+:137288810-137311932 | 6:+:137288810-137311932 | Cep170b |
| ENSRNOG00000012807 | 1.749629 | 0.006408 | up | 5:-:155261250-155264143 | 5:-:155261250-155264143 | C1qa |
| ENSRNOG00000017003 | -1.44806157669779 | 0.006458 | down | 4:-:121217629-121257885 | 4:-:121217629-121257885 | Plxna1 |
| ENSRNOG00000002963 | 1.701328 | 0.00645 | up | 10:-:91040005-91047177 | 10:-:91040005-91047177 | C1ql1 |
| ENSRNOG00000019645 | -1.43602000743164 | 0.006515 | down | 14:-:83943977-84106997 | 14:-:83943977-84106997 | Osbp2 |
| ENSRNOG00000010731 | -1.31587251966591 | 0.006503 | down | 16:-:39367692-39719187 | 16:-:39367692-39719187 | Gpm6a |
| ENSRNOG00000001658 | -2.78322040038181 | 0.005365 | down | 11:-:35024196-35099383 | 11:-:35024196-35099383 | Kcnj6 |
| ENSRNOG00000043085 | -2.26128458421431 | 0.00648 | down | 8:-:105323837-105462168 | 8:-:105323837-105462168 | Clstn2 |
| ENSRNOG00000021098 | 1.849558 | 0.006203 | up | 1:+:221773254-221788765 | 1:+:221773254-221788765 | Rasgrp2 |
| ENSRNOG00000004280 | 1.355762 | 0.006531 | up | 14:-:84174138-84189266 | 14:-:84174138-84189266 | Tcn2 |
| ENSRNOG00000021823 | -3.69202434782649 | 0.001729 | down | 15:-:51174989-51176858 | 15:-:51174989-51176858 | AABR07018318.1 |
| ENSRNOG00000025278 | -2.30232765229179 | 0.004464 | down | 3:-:23362707-23474170 | 3:-:23362707-23474170 | Scai |
| ENSRNOG00000059479 | -6.89791073296983 | 0.005467 | down | 14:+:87312203-87421659 | 14:+:87312203-87421659 | Adcy1 |
| ENSRNOG00000026989 | 4.050954 | 0.001644 | up | 2:-:41869556-41871858 | 2:-:41869556-41871858 | Gapt |
| ENSRNOG00000012460 | 1.971522 | 0.005068 | up | 1:-:229599009-229601032 | 1:-:229599009-229601032 | Cntf |
| ENSRNOG00000007939 | 1.74627 | 0.006185 | up | 7:-:116922944-116926555 | 7:-:116922944-116926555 | Naprt |
| ENSRNOG00000001368 | -2.950452085602 | 0.006961 | down | 12:+:41073824-41148490 | 12:+:41073824-41148490 | Rph3a |
| ENSRNOG00000002736 | -1.53495795672909 | 0.006352 | down | 13:-:78011047-78609045 | 13:-:78011047-78609045 | Rabgap1l |
| ENSRNOG00000009884 | 2.503455 | 0.006569 | up | 7:+:120153184-120156289 | 7:+:120153184-120156289 | Lgals1 |
| ENSRNOG00000016046 | -2.78248345247996 | 0.006674 | down | 17:+:53229785-53773657 | 17:+:53229785-53773657 | Hecw1 |
| ENSRNOG00000011559 | 1.377151 | 0.007039 | up | 2:+:225005019-225036188 | 2:+:225005019-225036188 | Cnn3 |
| ENSRNOG00000020698 | 1.55493 | 0.007065 | up | 10:+:89376530-89380101 | 10:+:89376530-89380101 | Rnd2 |
| ENSRNOG00000057404 | 4.658397 | 0.002446 | up | 10:-:47632192-47666921 | 10:-:47632192-47666921 | Slc47a1 |
| ENSRNOG00000011334 | -1.57068035237344 | 0.007084 | down | 6:+:111049559-111120799 | 6:+:111049559-111120799 | Tmem63c |
| ENSRNOG00000013389 | -2.40563150281137 | 0.006565 | down | 2:-:35077504-35104963 | 2:-:35077504-35104963 | Rgs7bp |
| ENSRNOG00000025612 | -2.093190840737 | 0.007262 | down | 12:+:50090464-50241995 | 12:+:50090464-50241995 | Sez6l |
| ENSRNOG00000016980 | 2.839403 | 0.004396 | up | 1:-:198544262-198559568 | 1:-:198544262-198559568 | Qprt |
| ENSRNOG00000003217 | 2.000551 | 0.006761 | up | 10:-:107415326-107424710 | 10:-:107415326-107424710 | Lgals3bp |
| ENSRNOG00000000886 | -3.4676382460112 | 0.005424 | down | 12:-:29308341-29743705 | 12:-:29308341-29743705 | Caln1 |
| ENSRNOG00000057840 | -3.83420651504532 | 0.002144 | down | 16:+:77995246-78016003 | 16:+:77995246-78016003 | AABR07026473.1 |
| ENSRNOG00000062181 | 1.318311 | 0.007304 | up | 11:-:61994181-61995549 | 11:-:61994181-61995549 | AABR07072236.1 |
| ENSRNOG00000007457 | 2.84517 | 0.006897 | up | 3:-:72161189-72171078 | 3:-:72161189-72171078 | Serping1 |
| ENSRNOG00000012568 | -1.39769158753751 | 0.00755 | down | 3:-:79960301-80003032 | 3:-:79960301-80003032 | Madd |
| ENSRNOG00000024277 | 2.673755 | 0.003898 | up | 12:+:15700825-15844512 | 12:+:15700825-15844512 | AABR07035470.1 |
| ENSRNOG00000005509 | -1.50744753256925 | 0.007672 | down | 3:-:129042742-129357491 | 3:-:129042742-129357491 | Pak7 |
| ENSRNOG00000015599 | 3.654577 | 0.002289 | up | 3:-:120272583-120306551 | 3:-:120272583-120306551 | Mall |
| ENSRNOG00000057344 | 5.254788 | 0.002529 | up | 3:-:20479688-20479999 | 3:-:20479688-20479999 | AABR07051733.2 |
| ENSRNOG00000010081 | 1.866485 | 0.006742 | up | 2:-:178585592-178616719 | 2:-:178585592-178616719 | Tmem144 |
| ENSRNOG00000013907 | 2.070501 | 0.007835 | up | 19:+:23389375-23405039 | 19:+:23389375-23405039 | Sall1 |
| ENSRNOG00000019183 | -4.69309832941441 | 0.004138 | down | 10:-:56953691-56962161 | 10:-:56953691-56962161 | Alox15 |
| ENSRNOG00000011913 | 1.784289 | 0.007806 | up | 2:+:104744461-104799853 | 2:+:104744461-104799853 | Cp |
| ENSRNOG00000023951 | -1.63333920396648 | 0.007984 | down | 7:-:63361696-63407241 | 7:-:63361696-63407241 | Tbc1d30 |
| ENSRNOG00000059840 | -1.87279193311226 | 0.008127 | down | 17:-:10153994-10208360 | 17:-:10153994-10208360 | Unc5a |
| ENSRNOG00000031743 | 2.120376 | 0.005823 | up | 2:+:248276709-248293784 | 2:+:248276709-248293784 | Gbp2 |
| ENSRNOG00000008423 | -1.96653908771154 | 0.008159 | down | 6:-:51011426-51019407 | 6:-:51011426-51019407 | Gpr22 |
| ENSRNOG00000018927 | -2.93531299441185 | 0.007184 | down | 1:-:212622094-212622537 | 1:-:212622094-212622537 | Sprn |
| ENSRNOG00000055382 | -1.87488499707723 | 0.008193 | down | 2:+:50099576-50499799 | 2:+:50099576-50499799 | Hcn1 |
| ENSRNOG00000028708 | 3.138834 | 0.004289 | up | 3:+:175885894-176046345 | 3:+:175885894-176046345 | Ntsr1 |
| ENSRNOG00000026791 | -1.74369984394175 | 0.008349 | down | 19:-:57127054-57192095 | 19:-:57127054-57192095 | Pgbd5 |
| ENSRNOG00000013555 | 1.980281 | 0.006697 | up | 2:+:44096061-44196605 | 2:+:44096061-44196605 | Ankrd55 |
| ENSRNOG00000015318 | 1.594899 | 0.008414 | up | 5:+:140923914-140940905 | 5:+:140923914-140940905 | Heyl |
| ENSRNOG00000014722 | -2.2194901961522 | 0.007869 | down | 9:-:67293309-67341341 | 9:-:67293309-67341341 | Raph1 |
| ENSRNOG00000009112 | 1.413964 | 0.008573 | up | 4:-:30344709-30380119 | 4:-:30344709-30380119 | Pon2 |
| ENSRNOG00000038999 | 2.239734 | 0.008204 | up | 20:+:5351605-5421098 | 20:+:5351605-5421098 | RT1-A1 |
| ENSRNOG00000030269 | -1.4600217596399 | 0.008547 | down | 4:-:145703046-146016325 | 4:-:145703046-146016325 | Atp2b2 |
| ENSRNOG00000009863 | -1.28198133982466 | 0.008788 | down | 4:-:145450861-145454834 | 4:-:145450861-145454834 | Prrt3 |
| ENSRNOG00000052564 | 1.983529 | 0.008371 | up | 10:+:40247436-40255422 | 10:+:40247436-40255422 | Gpx3 |
| ENSRNOG00000017060 | -2.3651265484907 | 0.008919 | down | 17:-:65535403-65955606 | 17:-:65535403-65955606 | Ryr2 |
| ENSRNOG00000014297 | 1.513106 | 0.008996 | up | 3:-:160872503-160891190 | 3:-:160872503-160891190 | Sdc4 |
| ENSRNOG00000052955 | -3.64722392724671 | 0.002812 | down | 7:-:70452367-70452675 | 7:-:70452367-70452675 | AC114111.1 |
| ENSRNOG00000004932 | -2.39861005315107 | 0.009027 | down | 14:+:60123169-60229734 | 14:+:60123169-60229734 | Sel1l3 |
| ENSRNOG00000011054 | 1.722335 | 0.009044 | up | 5:+:149047681-149069719 | 5:+:149047681-149069719 | Laptm5 |
| ENSRNOG00000047085 | -1.67417041657512 | 0.009123 | down | 18:-:28012877-28017925 | 18:-:28012877-28017925 | Lrrtm2 |
| ENSRNOG00000050697 | 1.930152 | 0.008712 | up | 3:-:172527107-172537877 | 3:-:172527107-172537877 | Ctsz |
| ENSRNOG00000025115 | -3.32416096217399 | 0.008786 | down | 8:+:57886168-57962708 | 8:+:57886168-57962708 | Exph5 |
| ENSRNOG00000004292 | 1.206829 | 0.00917 | up | 6:-:75540174-75551625 | 6:-:75540174-75551625 | Sptssa |
| ENSRNOG00000001344 | 1.16694 | 0.009212 | up | 12:+:40466495-40498752 | 12:+:40466495-40498752 | Aldh2 |
| ENSRNOG00000012759 | -4.13515711334646 | 0.004284 | down | 9:-:30515089-30844199 | 9:-:30515089-30844199 | Col19a1 |
| ENSRNOG00000016255 | 1.555051 | 0.009176 | up | 4:-:120785495-120817377 | 4:-:120785495-120817377 | Podxl2 |
| ENSRNOG00000017108 | -1.99820552478259 | 0.009252 | down | 7:+:121311024-121334437 | 7:+:121311024-121334437 | Syngr1 |
| ENSRNOG00000023375 | 1.388911 | 0.009221 | up | 10:+:65448950-65452177 | 10:+:65448950-65452177 | Rab34 |
| ENSRNOG00000006557 | -1.29430711112709 | 0.009131 | down | 10:-:31278746-31419235 | 10:-:31278746-31419235 | Cyfip2 |
| ENSRNOG00000010079 | 4.716126 | 0.004816 | up | 2:-:88126667-88135410 | 2:-:88126667-88135410 | Ca3 |
| ENSRNOG00000009253 | -4.89363341771961 | 0.005689 | down | 8:+:28352772-28387383 | 8:+:28352772-28387383 | Igsf9b |
| ENSRNOG00000003841 | -2.14891268795464 | 0.009457 | down | 13:+:110920737-111232269 | 13:+:110920737-111232269 | Kcnh1 |
| ENSRNOG00000059538 | 1.385238 | 0.009443 | up | 4:+:162934195-162943981 | 4:+:162934195-162943981 | Clec2g |
| ENSRNOG00000054809 | -5.1099077049387 | 0.005227 | down | 16:-:60008769-60018283 | 16:-:60008769-60018283 | AABR07026032.1 |
| ENSRNOG00000003680 | -2.91196272904127 | 0.009529 | down | 10:+:27973681-28187565 | 10:+:27973681-28187565 | Gabrb2 |
| ENSRNOG00000012216 | 2.215773 | 0.00705 | up | 17:-:8400146-8429338 | 17:-:8400146-8429338 | Tgfbi |
| ENSRNOG00000016221 | -2.84938294398093 | 0.009643 | down | 8:+:49418965-49427689 | 8:+:49418965-49427689 | Scn2b |
| ENSRNOG00000010479 | -1.12073044832208 | 0.009582 | down | 15:-:87506959-93868301 | 15:-:87506959-93868301 | Mycbp2 |
| ENSRNOG00000020014 | 1.407198 | 0.00968 | up | 1:-:100608966-100671074 | 1:-:100608966-100671074 | Myh14 |
| ENSRNOG00000033261 | -2.15476031125111 | 0.009632 | down | 15:+:18399515-18415965 | 15:+:18399515-18415965 | Fam107a |
| ENSRNOG00000012960 | 1.679253 | 0.00955 | up | 3:-:2579258-2584523 | 3:-:2579258-2584523 | Uap1l1 |
| ENSRNOG00000042519 | -1.90203863527043 | 0.00957 | down | 8:-:60867819-61079526 | 8:-:60867819-61079526 | Peak1 |
| ENSRNOG00000048782 | 1.78024 | 0.009892 | up | 4:-:122994425-123040609 | 4:-:122994425-123040609 | Wnt7a |
| ENSRNOG00000020021 | -2.41163764644998 | 0.009923 | down | 1:+:214927172-214947624 | 1:+:214927172-214947624 | Brsk2 |
| ENSRNOG00000059456 | -1.78248503492618 | 0.009642 | down | 12:+:49626871-49746272 | 12:+:49626871-49746272 | Grk3 |
| ENSRNOG00000018505 | 4.366882 | 0.004319 | up | 18:+:63098144-63108051 | 18:+:63098144-63108051 | Cidea |
| ENSRNOG00000002256 | 2.188758 | 0.00892 | up | 14:-:17143040-17225389 | 14:-:17143040-17225389 | Art3 |
| ENSRNOG00000022248 | -2.14422434745029 | 0.009994 | down | 10:+:15183803-15186978 | 10:+:15183803-15186978 | Fbxl16 |
| ENSRNOG00000058308 | -1.38784385454923 | 0.010149 | down | 10:-:68248655-68517564 | 10:-:68248655-68517564 | Asic2 |
| ENSRNOG00000000186 | 1.532152 | 0.010134 | up | 7:-:119616324-119623072 | 7:-:119616324-119623072 | Tst |
| ENSRNOG00000013484 | 2.939353 | 0.010162 | up | 9:-:27368272-27452902 | 9:-:27368272-27452902 | Gsta1 |
| ENSRNOG00000003365 | -1.56425937749838 | 0.01004 | down | 13:-:91842333-91873096 | 13:-:91842333-91873096 | Cadm3 |
| ENSRNOG00000017079 | -1.3240621055971 | 0.010162 | down | 1:+:165237847-165286980 | 1:+:165237847-165286980 | Pgm2l1 |
| ENSRNOG00000010747 | 1.654686 | 0.01005 | up | 2:+:84275884-84328998 | 2:+:84275884-84328998 | Dap |
| ENSRNOG00000002831 | 3.101456 | 0.004744 | up | 10:-:81936444-81942188 | 10:-:81936444-81942188 | Wfikkn2 |
| ENSRNOG00000020951 | -4.29737980423896 | 0.005225 | down | 10:-:90296141-90312386 | 10:-:90296141-90312386 | Slc4a1 |
| ENSRNOG00000002496 | -2.4856328046108 | 0.01038 | down | 11:+:66316606-66566331 | 11:+:66316606-66566331 | Stxbp5l |
| ENSRNOG00000002092 | -4.24551422259325 | 0.006089 | down | 11:+:30550141-30663465 | 11:+:30550141-30663465 | Hunk |
| ENSRNOG00000003296 | -2.04966367451025 | 0.010291 | down | 14:-:20935369-20953095 | 14:-:20935369-20953095 | Dck |
| ENSRNOG00000007271 | -1.46438988524947 | 0.010669 | down | 6:-:105459706-105518748 | 6:-:105459706-105518748 | Map3k9 |
| ENSRNOG00000016257 | 1.421349 | 0.010731 | up | 19:-:52465087-52499433 | 19:-:52465087-52499433 | Cotl1 |
| ENSRNOG00000022421 | -1.48504296856822 | 0.01097 | down | 16:+:20740826-20794039 | 16:+:20740826-20794039 | Crtc1 |
| ENSRNOG00000062291 | -3.87664534371153 | 0.004272 | down | 2:-:184459622-184460674 | 2:-:184459622-184460674 | AABR07012058.2 |
| ENSRNOG00000001548 | 1.253045 | 0.011002 | up | 3:-:62497571-62524996 | 3:-:62497571-62524996 | Nfe2l2 |
| ENSRNOG00000017194 | 1.422773 | 0.010935 | up | 5:+:135536413-135551990 | 5:+:135536413-135551990 | Prdx1 |
| ENSRNOG00000018454 | 2.042273 | 0.010709 | up | 1:-:80612895-80617057 | 1:-:80612895-80617057 | Apoe |
| ENSRNOG00000012720 | 4.564942 | 0.00498 | up | 1:-:32634796-32643771 | 1:-:32634796-32643771 | Irx4 |
| ENSRNOG00000009264 | -1.84436397867993 | 0.011114 | down | 4:-:152087379-152380184 | 4:-:152087379-152380184 | Erc1 |
| ENSRNOG00000001152 | 4.870893 | 0.004987 | up | 20:+:9586075-9626228 | 20:+:9586075-9626228 | Glp1r |
| ENSRNOG00000000457 | 2.244704 | 0.009046 | up | 20:+:3979035-3989669 | 20:+:3979035-3989669 | Tap1 |
| ENSRNOG00000019751 | 2.668021 | 0.007128 | up | 1:-:171971701-171979933 | 1:-:171971701-171979933 | Cyb5r2 |
| ENSRNOG00000058217 | -3.91927151905188 | 0.005182 | down | X:-:129480906-129561668 | X:-:129480906-129561668 | AABR07041418.1 |
| ENSRNOG00000007057 | -1.80268497224897 | 0.011267 | down | 5:+:146383942-146739418 | 5:+:146383942-146739418 | Csmd2 |
| ENSRNOG00000020478 | -2.58267235982972 | 0.0113 | down | 18:+:25749098-25965957 | 18:+:25749098-25965957 | Camk4 |
| ENSRNOG00000046159 | -1.69502894781011 | 0.010415 | down | 7:+:65616177-65653052 | 7:+:65616177-65653052 | Gns |
| ENSRNOG00000013102 | 1.32837 | 0.011373 | up | 3:+:2617805-2623445 | 3:+:2617805-2623445 | Entpd2 |
| ENSRNOG00000031126 | 2.927618 | 0.008973 | up | 8:-:46603728-46675544 | 8:-:46603728-46675544 | Tecta |
| ENSRNOG00000049768 | -2.00307289842739 | 0.011432 | down | 10:+:11392625-11512600 | 10:+:11392625-11512600 | Adcy9 |
| ENSRNOG00000007014 | -2.67098906080218 | 0.011415 | down | X:+:39711201-39951847 | X:+:39711201-39951847 | Cnksr2 |
| ENSRNOG00000052319 | -2.06030510239611 | 0.01129 | down | 1:+:165284410-165284635 | 1:+:165284410-165284635 | AABR07004881.1 |
| ENSRNOG00000004137 | -1.22912363130901 | 0.011503 | down | 10:+:17327275-17392185 | 10:+:17327275-17392185 | Ubtd2 |
| ENSRNOG00000016484 | 1.361829 | 0.011444 | up | 4:+:71621729-71626107 | 4:+:71621729-71626107 | Gstk1 |
| ENSRNOG00000000167 | -2.44071457202736 | 0.009763 | down | X:-:23167696-23187341 | X:-:23167696-23187341 | Alas2 |
| ENSRNOG00000032569 | -3.69836467205661 | 0.009779 | down | 7:+:11737293-11746703 | 7:+:11737293-11746703 | Lingo3 |
| ENSRNOG00000059900 | 2.438622 | 0.010353 | up | 16:-:19938784-19942353 | 16:-:19938784-19942353 | Bst2 |
| ENSRNOG00000015991 | 1.139017 | 0.011635 | up | 5:+:59128315-59147255 | 5:+:59128315-59147255 | Npr2 |
| ENSRNOG00000017702 | 1.801397 | 0.011664 | up | 17:-:42085028-42127678 | 17:-:42085028-42127678 | Gpld1 |
| ENSRNOG00000059008 | -2.2100582639762 | 0.011665 | down | 10:+:85301875-85330397 | 10:+:85301875-85330397 | Socs7 |
| ENSRNOG00000019504 | -4.77964588766471 | 0.00661 | down | 16:+:81616642-81687655 | 16:+:81616642-81687655 | AABR07026536.1 |
| ENSRNOG00000053045 | -3.52156614045375 | 0.011038 | down | 12:-:48817320-48857724 | 12:-:48817320-48857724 | Wscd2 |
| ENSRNOG00000047714 | 3.285054 | 0.005302 | up | 13:-:36094520-36101411 | 13:-:36094520-36101411 | Tmem37 |
| ENSRNOG00000012865 | 1.979458 | 0.009012 | up | 8:-:115173453-115179191 | 8:-:115173453-115179191 | Parp3 |
| ENSRNOG00000061132 | -3.72499378096555 | 0.005465 | down | 18:-:44029927-44141865 | 18:-:44029927-44141865 | LOC103694210 |
| ENSRNOG00000035583 | 3.694462 | 0.004803 | up | 15:+:33613435-33613550 | 15:+:33613435-33613550 | Mir3546 |
| ENSRNOG00000048834 | 2.429625 | 0.007412 | up | 9:+:10773901-10781109 | 9:+:10773901-10781109 | Plin3 |
| ENSRNOG00000013938 | -2.31240857633442 | 0.011538 | down | 1:-:92703080-92774975 | 1:-:92703080-92774975 | Tshz3 |
| ENSRNOG00000055226 | -1.9072116905829 | 0.011961 | down | 3:+:14889510-15060286 | 3:+:14889510-15060286 | Dab2ip |
| ENSRNOG00000054984 | -4.01411444867161 | 0.005331 | down | 16:+:78236947-78404678 | 16:+:78236947-78404678 | AABR07026483.1 |
| ENSRNOG00000058975 | -1.36964142735813 | 0.011994 | down | X:+:22212132-22294402 | X:+:22212132-22294402 | Iqsec2 |
| ENSRNOG00000008182 | 1.27476 | 0.012112 | up | 14:+:80248140-80276534 | 14:+:80248140-80276534 | Htra3 |
| ENSRNOG00000012942 | -2.21108972276516 | 0.012262 | down | 9:+:2190915-2274629 | 9:+:2190915-2274629 | Satb1 |
| ENSRNOG00000014314 | 3.674949 | 0.005702 | up | 7:-:117675720-117680004 | 7:-:117675720-117680004 | Slc39a4 |
| ENSRNOG00000015461 | 1.496837 | 0.01204 | up | 9:-:85560863-85626094 | 9:-:85560863-85626094 | Serpine2 |
| ENSRNOG00000003171 | 6.276074 | 0.008571 | up | 13:+:89524329-89530068 | 13:+:89524329-89530068 | Mpz |
| ENSRNOG00000004109 | -2.09746474454344 | 0.01223 | down | 7:+:79638046-79964405 | 7:+:79638046-79964405 | Zfpm2 |
| ENSRNOG00000009433 | 5.028548 | 0.007532 | up | 2:-:230199343-230273709 | 2:-:230199343-230273709 | Mcub |
| ENSRNOG00000007230 | 1.607139 | 0.0125 | up | 7:+:18668692-18681939 | 7:+:18668692-18681939 | Kank3 |
| ENSRNOG00000020410 | 4.827755 | 0.008345 | up | 1:-:216073031-216080287 | 1:-:216073031-216080287 | Th |
| ENSRNOG00000000991 | 1.379802 | 0.0126 | up | 12:-:11252296-11265865 | 12:-:11252296-11265865 | Arpc1b |
| ENSRNOG00000011250 | 4.823826 | 0.007314 | up | 4:-:85381889-85386231 | 4:-:85381889-85386231 | Inmt |
| ENSRNOG00000005478 | 1.751127 | 0.012674 | up | 4:+:87167514-87234197 | 4:+:87167514-87234197 | Fkbp9 |
| ENSRNOG00000024990 | 3.991153 | 0.006438 | up | 3:+:66673071-66780075 | 3:+:66673071-66780075 | Ppp1r1c |
| ENSRNOG00000011971 | 2.29807 | 0.011273 | up | 4:-:157143592-157155609 | 4:-:157143592-157155609 | C1s |
| ENSRNOG00000059827 | 3.800498 | 0.012815 | up | 10:-:57669530-57671080 | 10:-:57669530-57671080 | LOC691995 |
| ENSRNOG00000017307 | 1.359149 | 0.012732 | up | 1:-:153732532-153752541 | 1:-:153732532-153752541 | Prss23 |
| ENSRNOG00000000825 | 4.07634 | 0.006214 | up | 20:-:27651312-27657983 | 20:-:27651312-27657983 | Calhm5 |
| ENSRNOG00000027960 | -3.65733596289991 | 0.005638 | down | 6:-:107572233-107578467 | 6:-:107572233-107578467 | AC094055.1 |
| ENSRNOG00000007743 | 3.263347 | 0.011079 | up | 4:+:172119331-172134607 | 4:+:172119331-172134607 | Mgst1 |
| ENSRNOG00000001285 | -1.40052025856539 | 0.012537 | down | 12:+:39553903-39603326 | 12:+:39553903-39603326 | Atp2a2 |
| ENSRNOG00000018158 | -1.70093227196391 | 0.013049 | down | 1:+:47162394-47202549 | 1:+:47162394-47202549 | Tmem181 |
| ENSRNOG00000052707 | -1.79792265313775 | 0.01278 | down | 19:+:25526751-25749550 | 19:+:25526751-25749550 | Cacna1a |
| ENSRNOG00000045604 | -3.56272335781327 | 0.006964 | down | 18:-:73563616-73564245 | 18:-:73563616-73564245 | AC139391.1 |
| ENSRNOG00000020151 | 5.068256 | 0.008473 | up | 19:+:38768467-38838395 | 19:+:38768467-38838395 | Cdh1 |
| ENSRNOG00000038686 | 1.6428 | 0.012997 | up | X:-:32329598-32355307 | X:-:32329598-32355307 | Ap1s2 |
| ENSRNOG00000006235 | -1.50471601425169 | 0.012766 | down | 7:-:136526496-136853957 | 7:-:136526496-136853957 | Nell2 |
| ENSRNOG00000004448 | 1.457861 | 0.012393 | up | 7:-:49047013-49250957 | 7:-:49047013-49250957 | Acss3 |
| ENSRNOG00000019728 | 1.885439 | 0.012325 | up | 1:+:199495298-199623960 | 1:+:199495298-199623960 | Itgam |
| ENSRNOG00000050057 | -1.0826895421709 | 0.013042 | down | 10:+:13723405-13779963 | 10:+:13723405-13779963 | Abca3 |
| ENSRNOG00000013014 | 2.2123 | 0.012546 | up | 19:-:55249616-55257876 | 19:-:55249616-55257876 | Cyba |
| ENSRNOG00000008901 | -4.30101430475999 | 0.009702 | down | 5:-:62156839-62187930 | 5:-:62156839-62187930 | Coro2a |
| ENSRNOG00000019147 | 1.381767 | 0.013081 | up | 3:-:14514868-14538241 | 3:-:14514868-14538241 | Stom |
| ENSRNOG00000049770 | 3.83048 | 0.006208 | up | 12:+:50407843-50414432 | 12:+:50407843-50414432 | Cryba4 |
| ENSRNOG00000005286 | 2.296612 | 0.013257 | up | 6:+:72359791-72373695 | 6:+:72359791-72373695 | Coch |
| ENSRNOG00000033942 | -4.99786536362756 | 0.011021 | down | 10:+:5930298-6119990 | 10:+:5930298-6119990 | Grin2a |
| ENSRNOG00000022256 | 4.353497 | 0.007599 | up | 14:+:17210733-17212930 | 14:+:17210733-17212930 | Cxcl10 |
| ENSRNOG00000017737 | -1.36853072926917 | 0.013091 | down | 3:-:80844005-80875817 | 3:-:80844005-80875817 | Dgkz |
| ENSRNOG00000046057 | 4.923628 | 0.007009 | up | 10:+:86340940-86342858 | 10:+:86340940-86342858 | Pnmt |
| ENSRNOG00000006553 | 3.273325 | 0.006458 | up | 5:-:102415847-102786331 | 5:-:102415847-102786331 | Bnc2 |
| ENSRNOG00000058678 | -2.83792150953772 | 0.006541 | down | 20:-:4964125-4966710 | 20:-:4964125-4966710 | AABR07072810.1 |
| ENSRNOG00000016717 | 1.764541 | 0.012043 | up | 1:+:107262659-107363788 | 1:+:107262659-107363788 | Gas2 |
| ENSRNOG00000060523 | 4.440782 | 0.006341 | up | 20:-:3134704-3135301 | 20:-:3134704-3135301 | AABR07044362.6 |
| ENSRNOG00000002734 | -2.22755138061135 | 0.013704 | down | X:-:147584268-147586777 | X:-:147584268-147586777 | AABR07042077.1 |
| ENSRNOG00000046254 | 1.741916 | 0.013028 | up | 9:-:9431860-9585865 | 9:-:9431860-9585865 | Adgre1 |
| ENSRNOG00000023601 | -1.2390481231851 | 0.013569 | down | 5:-:130001372-130085838 | 5:-:130001372-130085838 | Elavl4 |
| ENSRNOG00000004680 | -1.72644663974689 | 0.013336 | down | 3:+:35014538-35257407 | 3:+:35014538-35257407 | Kif5c |
| ENSRNOG00000000843 | 4.944701 | 0.007544 | up | 20:+:5057701-5059933 | 20:+:5057701-5059933 | Ly6g6c |
| ENSRNOG00000001953 | 1.591451 | 0.012826 | up | 11:+:37798370-37880821 | 11:+:37798370-37880821 | Bace2 |
| ENSRNOG00000024479 | -4.8736433413382 | 0.01181 | down | X:-:39954904-39956841 | X:-:39954904-39956841 | Klhl34 |
| ENSRNOG00000012966 | 1.492594 | 0.014018 | up | 9:-:73833388-73871888 | 9:-:73833388-73871888 | Acadl |
| ENSRNOG00000051875 | 3.533778 | 0.006912 | up | 1:-:62207548-62223307 | 1:-:62207548-62223307 | AABR07001926.2 |
| ENSRNOG00000015159 | -4.74425865023159 | 0.007833 | down | 1:-:31734327-31777070 | 1:-:31734327-31777070 | Slc9a3 |
| ENSRNOG00000029134 | -1.54789024402892 | 0.013696 | down | 19:+:25029037-25069142 | 19:+:25029037-25069142 | Adgrl1 |
| ENSRNOG00000003201 | 1.346911 | 0.01372 | up | X:-:72074108-72078551 | X:-:72074108-72078551 | Rps4x |
| ENSRNOG00000024631 | 1.320869 | 0.014188 | up | 7:-:122922022-122926336 | 7:-:122922022-122926336 | Chadl |
| ENSRNOG00000059662 | 3.450988 | 0.007873 | up | 1:-:131380879-131431948 | 1:-:131380879-131431948 | AABR07004228.1 |
| ENSRNOG00000005809 | 1.562277 | 0.014028 | up | 4:-:170913923-170932618 | 4:-:170913923-170932618 | Arhgdib |
| ENSRNOG00000014227 | 3.657464 | 0.006833 | up | 1:-:103298174-103323476 | 1:-:103298174-103323476 | Mrgprx3 |
| ENSRNOG00000000957 | 2.075519 | 0.014055 | up | 12:-:9996951-9998779 | 12:-:9996951-9998779 | Rpl21 |
| ENSRNOG00000059013 | 2.13543 | 0.013999 | up | 4:-:77413672-77446624 | 4:-:77413672-77446624 | AABR07060519.1 |
| ENSRNOG00000017712 | 5.890475 | 0.010309 | up | 2:-:30125249-30127269 | 2:-:30125249-30127269 | Cartpt |
| ENSRNOG00000012410 | 1.901475 | 0.014432 | up | 2:-:189900667-189903219 | 2:-:189900667-189903219 | S100a1 |
| ENSRNOG00000000105 | -2.29941308971939 | 0.014014 | down | 17:-:10756285-10818835 | 17:-:10756285-10818835 | Cplx2 |
| ENSRNOG00000008236 | 1.535624 | 0.014491 | up | 5:-:29573898-29601748 | 5:-:29573898-29601748 | Decr1 |
| ENSRNOG00000060728 | 1.154025 | 0.013872 | up | 7:-:140637287-140640953 | 7:-:140637287-140640953 | Tuba1a |
| ENSRNOG00000005705 | 2.1022 | 0.012275 | up | 6:+:1657331-1689559 | 6:+:1657331-1689559 | Qpct |
| ENSRNOG00000001963 | 1.724568 | 0.014653 | up | 11:+:38035450-38059950 | 11:+:38035450-38059950 | Mx2 |
| ENSRNOG00000048812 | 1.628395 | 0.014494 | up | 8:+:117117430-117118522 | 8:+:117117430-117118522 | Gpx1 |
| ENSRNOG00000043410 | 2.11841 | 0.010313 | up | 8:-:6218393-6235967 | 8:-:6218393-6235967 | Cfap300 |
| ENSRNOG00000011624 | -2.56201795630003 | 0.014619 | down | 3:-:176589906-176644951 | 3:-:176589906-176644951 | Kcnq2 |
| ENSRNOG00000053450 | -1.31053113754875 | 0.014681 | down | 1:-:64438472-64446818 | 1:-:64438472-64446818 | Myadm |
| ENSRNOG00000012827 | 5.607271 | 0.009974 | up | 2:+:164549455-164582643 | 2:+:164549455-164582643 | Mlf1 |
| ENSRNOG00000059485 | -3.69388883498379 | 0.008197 | down | 10:+:37458452-37493491 | 10:+:37458452-37493491 | Cdkl3 |
| ENSRNOG00000003620 | 2.711534 | 0.007201 | up | 13:-:80837420-80862963 | 13:-:80837420-80862963 | Fmo3 |
| ENSRNOG00000014859 | -2.49186807220397 | 0.014869 | down | 13:-:25186106-25262469 | 13:-:25186106-25262469 | Rnf152 |
| ENSRNOG00000059149 | -3.4925964541027 | 0.008263 | down | 10:-:90700111-90709932 | 10:-:90700111-90709932 | AABR07030498.1 |
| ENSRNOG00000008658 | 1.556632 | 0.014685 | up | 4:+:130172727-130425532 | 4:+:130172727-130425532 | Mitf |
| ENSRNOG00000031211 | 2.9676 | 0.008312 | up | 1:+:189233141-189272172 | 1:+:189233141-189272172 | Acsm5 |
| ENSRNOG00000050514 | 3.153636 | 0.008008 | up | 2:-:210115784-210116038 | 2:-:210115784-210116038 | LOC684509 |
| ENSRNOG00000010519 | 5.337506 | 0.010379 | up | 9:-:19804079-19880346 | 9:-:19804079-19880346 | Cyp39a1 |
| ENSRNOG00000016998 | -2.43541336989718 | 0.014885 | down | 17:+:19249952-19533814 | 17:+:19249952-19533814 | Atxn1 |
| ENSRNOG00000003553 | 2.48108 | 0.013997 | up | 14:+:113202419-113295014 | 14:+:113202419-113295014 | Efemp1 |
| ENSRNOG00000016684 | -1.29784726248293 | 0.014786 | down | 17:+:15924048-16032420 | 17:+:15924048-16032420 | Wnk2 |
| ENSRNOG00000017560 | 2.110583 | 0.015211 | up | 3:-:80841005-80842916 | 3:-:80841005-80842916 | Mdk |
| ENSRNOG00000049298 | -4.28650737494342 | 0.01065 | down | 4:-:155752447-155763500 | 4:-:155752447-155763500 | Slc2a3 |
| ENSRNOG00000013668 | 2.243252 | 0.0127 | up | 4:+:100407658-100419446 | 4:+:100407658-100419446 | Capg |
| ENSRNOG00000023226 | 2.188124 | 0.015264 | up | 2:+:193892589-193901236 | 2:+:193892589-193901236 | S100a10 |
| ENSRNOG00000046889 | 1.61122 | 0.015174 | up | 13:-:36117119-36174908 | 13:-:36117119-36174908 | Dbi |
| ENSRNOG00000018143 | 1.862394 | 0.013477 | up | 5:-:135675832-135677432 | 5:-:135675832-135677432 | Hpdl |
| ENSRNOG00000012156 | 1.27151 | 0.01563 | up | 1:+:234749568-234796739 | 1:+:234749568-234796739 | Ostf1 |
| ENSRNOG00000006144 | -1.6690791922808 | 0.015518 | down | 4:-:134784668-135069970 | 4:-:134784668-135069970 | Cntn3 |
| ENSRNOG00000009795 | -2.10340652938279 | 0.015741 | down | 5:-:100439082-100647727 | 5:-:100439082-100647727 | Nfib |
| ENSRNOG00000003386 | -2.21028729857943 | 0.015481 | down | 10:-:107516995-107539658 | 10:-:107516995-107539658 | Rbfox3 |
| ENSRNOG00000013526 | 2.195117 | 0.015445 | up | 4:-:148769096-148803988 | 4:-:148769096-148803988 | Rassf4 |
| ENSRNOG00000018416 | -1.1380280427178 | 0.015516 | down | 9:+:16862248-16902199 | 9:+:16862248-16902199 | Ttbk1 |
| ENSRNOG00000018291 | -4.12997000864249 | 0.009728 | down | 8:+:16287804-16333603 | 8:+:16287804-16333603 | LOC103693015 |
| ENSRNOG00000019692 | 1.337192 | 0.015524 | up | 10:-:15164439-15166457 | 10:-:15164439-15166457 | Metrn |
| ENSRNOG00000001224 | 1.758331 | 0.014496 | up | 20:-:11777783-11815647 | 20:-:11777783-11815647 | Itgb2 |
| ENSRNOG00000029841 | 2.468225 | 0.015276 | up | 13:-:32344966-32427177 | 13:-:32344966-32427177 | Cdh19 |
| ENSRNOG00000011501 | 1.078346 | 0.015509 | up | 8:-:104190032-104221342 | 8:-:104190032-104221342 | Atp1b3 |
| ENSRNOG00000019542 | 3.422706 | 0.009164 | up | 18:+:55576239-55584957 | 18:+:55576239-55584957 | MGC108823 |
| ENSRNOG00000011824 | 5.682322 | 0.011629 | up | 4:-:124110716-124113242 | 4:-:124110716-124113242 | Trh |
| ENSRNOG00000000456 | 2.955402 | 0.012039 | up | 20:+:3990613-3993769 | 20:+:3990613-3993769 | Psmb8 |
| ENSRNOG00000008843 | 1.357192 | 0.016319 | up | 10:+:13797562-13810913 | 10:+:13797562-13810913 | Eci1 |
| ENSRNOG00000024707 | 4.248618 | 0.009159 | up | 5:-:171355876-171415354 | 5:-:171355876-171415354 | Tp73 |
| ENSRNOG00000005580 | 4.4021 | 0.012417 | up | 10:+:104523996-104561078 | 10:+:104523996-104561078 | Itgb4 |
| ENSRNOG00000050090 | -1.38008611854731 | 0.015686 | down | 2:-:210249663-210299770 | 2:-:210249663-210299770 | Slc6a17 |
| ENSRNOG00000027756 | -1.80490407656444 | 0.016547 | down | 11:-:61432064-61499557 | 11:-:61432064-61499557 | Usf3 |
| ENSRNOG00000006096 | 2.798332 | 0.01054 | up | 5:-:27986663-28177340 | 5:-:27986663-28177340 | Slc26a7 |
| ENSRNOG00000017932 | -1.12094280727043 | 0.016182 | down | 19:-:43274493-43290363 | 19:-:43274493-43290363 | St3gal2 |
| ENSRNOG00000060195 | -3.61073143092386 | 0.009329 | down | 3:-:25356607-25414908 | 3:-:25356607-25414908 | AABR07051882.2 |
| ENSRNOG00000019244 | 1.463575 | 0.016661 | up | 5:+:173288447-173292929 | 5:+:173288447-173292929 | Mxra8 |
| ENSRNOG00000047446 | 2.514102 | 0.013092 | up | 19:+:53044379-53047081 | 19:+:53044379-53047081 | Foxc2 |
| ENSRNOG00000023463 | 1.414621 | 0.01621 | up | 11:-:67724076-67756799 | 11:-:67724076-67756799 | Parp9 |
| ENSRNOG00000010106 | -2.05863544611769 | 0.014918 | down | 5:+:36076565-36135884 | 5:+:36076565-36135884 | Faxc |
| ENSRNOG00000009881 | -2.92487658311638 | 0.00847 | down | 14:+:107785029-107798115 | 14:+:107785029-107798115 | Fam161a |
| ENSRNOG00000004361 | 2.216811 | 0.013849 | up | 7:-:117291877-117300662 | 7:-:117291877-117300662 | Parp10 |
| ENSRNOG00000025604 | -2.25265171513248 | 0.015085 | down | 7:+:97984862-98026052 | 7:+:97984862-98026052 | Atad2 |
| ENSRNOG00000015505 | 3.48295 | 0.008918 | up | 4:+:155313671-155336228 | 4:+:155313671-155336228 | Mfap5 |
| ENSRNOG00000053893 | 3.963242 | 0.009444 | up | 16:-:10943341-10952549 | 16:-:10943341-10952549 | Opn4 |
| ENSRNOG00000014532 | 3.988312 | 0.009832 | up | 3:+:154786215-154813464 | 3:+:154786215-154813464 | Lbp |
| ENSRNOG00000061508 | -4.30670172598316 | 0.014178 | down | X:+:33884499-34057399 | X:+:33884499-34057399 | Reps2 |
| ENSRNOG00000015903 | -1.73925169268096 | 0.016644 | down | 4:+:117743710-117882464 | 4:+:117743710-117882464 | Add2 |
| ENSRNOG00000010549 | 2.087528 | 0.013866 | up | 7:+:124460358-124470609 | 7:+:124460358-124470609 | Tspo |
| ENSRNOG00000007732 | -1.13196259147298 | 0.017116 | down | 3:-:81361056-81368442 | 3:-:81361056-81368442 | Slc35c1 |
| ENSRNOG00000007089 | 1.160678 | 0.016626 | up | 6:-:126282233-126320726 | 6:-:126282233-126320726 | Lgmn |
| ENSRNOG00000030467 | 3.114273 | 0.013 | up | 3:+:72191533-72206188 | 3:+:72191533-72206188 | Ube2l6 |
| ENSRNOG00000062209 | -3.08950295070973 | 0.009105 | down | 4:+:6313717-6314571 | 4:+:6313717-6314571 | AABR07059168.2 |
| ENSRNOG00000007637 | 1.333933 | 0.017219 | up | 5:+:105230580-105279731 | 5:+:105230580-105279731 | Acer2 |
| ENSRNOG00000030449 | 2.160593 | 0.01731 | up | 8:+:85497557-85518879 | 8:+:85497557-85518879 | Gsta4 |
| ENSRNOG00000021745 | -2.11156687706948 | 0.017243 | down | 2:+:102685513-102688624 | 2:+:102685513-102688624 | Bhlhe22 |
| ENSRNOG00000003745 | 1.850431 | 0.014119 | up | 13:-:109817728-109849632 | 13:-:109817728-109849632 | Atf3 |
| ENSRNOG00000014936 | 1.796805 | 0.016748 | up | 1:-:213750192-213751405 | 1:-:213750192-213751405 | Ifitm2 |
| ENSRNOG00000005888 | 3.590557 | 0.009654 | up | 3:-:109969152-110021149 | 3:-:109969152-110021149 | Fsip1 |
| ENSRNOG00000006110 | -2.48223811691583 | 0.016789 | down | 5:+:1417478-1511416 | 5:+:1417478-1511416 | Jph1 |
| ENSRNOG00000019357 | 1.322974 | 0.017433 | up | 10:+:59743544-59748062 | 10:+:59743544-59748062 | Tax1bp3 |
| ENSRNOG00000016091 | -1.27387506824534 | 0.01735 | down | 9:+:79630604-79654688 | 9:+:79630604-79654688 | Tmem169 |
| ENSRNOG00000012603 | -1.52082764194929 | 0.017576 | down | 3:-:64044112-64095120 | 3:-:64044112-64095120 | Sestd1 |
| ENSRNOG00000046950 | -2.57921869190036 | 0.016384 | down | 4:+:24612205-24640686 | 4:+:24612205-24640686 | LOC100912481 |
| ENSRNOG00000023400 | 1.75479 | 0.01549 | up | 11:+:67757928-67767622 | 11:+:67757928-67767622 | Dtx3l |
| ENSRNOG00000004823 | -2.14154240278612 | 0.017388 | down | 6:+:60566196-60782556 | 6:+:60566196-60782556 | Dock4 |
| ENSRNOG00000018680 | 2.117097 | 0.01708 | up | 18:+:70970596-70975494 | 18:+:70970596-70975494 | Rpl17 |
| ENSRNOG00000003183 | 2.092676 | 0.01782 | up | 13:+:50873605-50885563 | 13:+:50873605-50885563 | Fmod |
| ENSRNOG00000001104 | -1.52619994878777 | 0.017899 | down | 12:-:14180029-14244316 | 12:-:14180029-14244316 | Foxk1 |
| ENSRNOG00000030243 | -3.7267096210607 | 0.011875 | down | 3:-:25921427-26056818 | 3:-:25921427-26056818 | AABR07051892.1 |
| ENSRNOG00000049054 | 1.14888 | 0.017286 | up | 6:+:41917132-41923542 | 6:+:41917132-41923542 | Ntsr2 |
| ENSRNOG00000010841 | 3.003128 | 0.013582 | up | 5:+:144308611-144335172 | 5:+:144308611-144335172 | Col8a2 |
| ENSRNOG00000016182 | 1.68243 | 0.017766 | up | 4:+:117962319-118046344 | 4:+:117962319-118046344 | Tgfa |
| ENSRNOG00000062214 | -3.53459098450194 | 0.009967 | down | 2:-:184695861-184699901 | 2:-:184695861-184699901 | AABR07012065.2 |
| ENSRNOG00000025527 | -1.2766543793088 | 0.017658 | down | 9:-:114491419-114619711 | 9:-:114491419-114619711 | Mtcl1 |
| ENSRNOG00000019342 | 1.64467 | 0.017962 | up | 1:-:198100586-198104109 | 1:-:198100586-198104109 | Sult1a1 |
| ENSRNOG00000000894 | -1.78976032685245 | 0.017462 | down | 12:-:5573729-5822874 | 12:-:5573729-5822874 | Fry |
| ENSRNOG00000024762 | 7.082668 | 0.015392 | up | X:-:138098185-138148967 | X:-:138098185-138148967 | Frmd7 |
| ENSRNOG00000031138 | 1.805734 | 0.016033 | up | 10:-:34213936-34221928 | 10:-:34213936-34221928 | Irgm |
| ENSRNOG00000001215 | 1.065717 | 0.018144 | up | 20:-:11417429-11424324 | 20:-:11417429-11424324 | Cfap410 |
| ENSRNOG00000017469 | 2.15276 | 0.015922 | up | 1:-:237893966-237910012 | 1:-:237893966-237910012 | Anxa1 |
| ENSRNOG00000019387 | 1.698378 | 0.018136 | up | 16:+:20426566-20430752 | 16:+:20426566-20430752 | Ifi30 |
| ENSRNOG00000014199 | -1.2545884471596 | 0.018211 | down | 16:-:69237857-69242028 | 16:-:69237857-69242028 | Zfp703 |
| ENSRNOG00000002896 | 1.186718 | 0.017728 | up | 13:-:79077033-79088127 | 13:-:79077033-79088127 | Prdx6 |
| ENSRNOG00000003949 | -3.36359133882926 | 0.011214 | down | X:-:36857650-36884748 | X:-:36857650-36884748 | AABR07037995.1 |
| ENSRNOG00000005058 | -1.02488196826686 | 0.018081 | down | 3:+:103726238-103733354 | 3:+:103726238-103733354 | Lpcat4 |
| ENSRNOG00000061519 | -3.16667431790697 | 0.018177 | down | 6:+:43234526-43343551 | 6:+:43234526-43343551 | Asap2 |
| ENSRNOG00000047321 | -2.15590070318113 | 0.017917 | down | 10:-:15589364-15590220 | 10:-:15589364-15590220 | Hba-a2 |
| ENSRNOG00000016480 | 4.59813 | 0.011857 | up | 17:-:78904873-78910671 | 17:-:78904873-78910671 | Acbd7 |
| ENSRNOG00000060107 | -1.12868269879068 | 0.018702 | down | 2:+:243018975-243151933 | 2:+:243018975-243151933 | Dnajb14 |
| ENSRNOG00000015365 | -3.50970816374904 | 0.012097 | down | 9:+:88357556-88485830 | 9:+:88357556-88485830 | Col4a3 |
| ENSRNOG00000029886 | -1.85321471915385 | 0.017685 | down | 10:-:15602794-15603649 | 10:-:15602794-15603649 | Hba-a1 |
| ENSRNOG00000003269 | 1.047089 | 0.018688 | up | 10:-:16769666-16792909 | 10:-:16769666-16792909 | Atp6v0e1 |
| ENSRNOG00000007839 | -2.51066567169785 | 0.017319 | down | 7:-:68446193-68549763 | 7:-:68446193-68549763 | Slc16a7 |
| ENSRNOG00000009170 | -1.32429561597523 | 0.017884 | down | 8:-:58932580-59077690 | 8:-:58932580-59077690 | Dmxl2 |
| ENSRNOG00000004595 | -1.57000292448875 | 0.018803 | down | 7:-:142204036-142210738 | 7:-:142204036-142210738 | Pou6f1 |
| ENSRNOG00000056493 | -3.06118842541371 | 0.011443 | down | 7:-:29086156-29171783 | 7:-:29086156-29171783 | Mybpc1 |
| ENSRNOG00000024159 | 1.670581 | 0.018443 | up | 13:-:89601896-89606326 | 13:-:89601896-89606326 | Fcer1g |
| ENSRNOG00000020357 | -3.13369452100594 | 0.010931 | down | 2:-:188212211-188216482 | 2:-:188212211-188216482 | Msto1 |
| ENSRNOG00000010868 | -2.00112663720041 | 0.018817 | down | 3:-:160241619-160301552 | 3:-:160241619-160301552 | Rims4 |
| ENSRNOG00000021726 | 1.994912 | 0.017717 | up | 16:+:50016857-50031214 | 16:+:50016857-50031214 | Tlr3 |
| ENSRNOG00000019869 | -1.21374200419863 | 0.018627 | down | 1:-:85291462-85300825 | 1:-:85291462-85300825 | Lrfn1 |
| ENSRNOG00000017220 | 1.513851 | 0.018714 | up | 1:-:219127602-219139464 | 1:-:219127602-219139464 | Tcirg1 |
| ENSRNOG00000012858 | -3.62725116827386 | 0.011136 | down | 1:+:213597478-213600685 | 1:+:213597478-213600685 | Odf3 |
| ENSRNOG00000036911 | -1.87951192700587 | 0.019119 | down | 4:+:184019087-184165540 | 4:+:184019087-184165540 | Bicd1 |
| ENSRNOG00000001726 | -1.33073334676946 | 0.019145 | down | 11:+:73738433-73774190 | 11:+:73738433-73774190 | Tmem44 |
| ENSRNOG00000007650 | 1.493354 | 0.01846 | up | 7:+:3320103-3335582 | 7:+:3320103-3335582 | Cd63 |
| ENSRNOG00000050794 | 1.565338 | 0.019114 | up | 10:-:39390581-39405311 | 10:-:39390581-39405311 | Pdlim4 |
| ENSRNOG00000008463 | -1.33483666481038 | 0.019081 | down | 4:+:22445414-22586689 | 4:+:22445414-22586689 | Rundc3b |
| ENSRNOG00000009462 | 1.193751 | 0.019369 | up | 1:+:157403595-157417147 | 1:+:157403595-157417147 | Ccdc90b |
| ENSRNOG00000011006 | 5.642408 | 0.013624 | up | 2:+:142717281-142735410 | 2:+:142717281-142735410 | Stoml3 |
| ENSRNOG00000042729 | 2.180211 | 0.014612 | up | 9:+:27068443-27107709 | 9:+:27068443-27107709 | Efhc1 |
| ENSRNOG00000016828 | 1.295715 | 0.018889 | up | 15:+:33600102-33602834 | 15:+:33600102-33602834 | Cmtm5 |
| ENSRNOG00000002456 | -2.05195869489061 | 0.019479 | down | 10:-:77840568-77918191 | 10:-:77840568-77918191 | Hlf |
| ENSRNOG00000042478 | -2.91008842942347 | 0.019511 | down | 4:+:22859622-22996645 | 4:+:22859622-22996645 | Adam22 |
| ENSRNOG00000012804 | 1.434065 | 0.01938 | up | 5:-:155255005-155258392 | 5:-:155255005-155258392 | C1qc |
| ENSRNOG00000019937 | -1.43759064024136 | 0.018811 | down | 19:+:58823814-58862926 | 19:+:58823814-58862926 | Kcnk1 |
| ENSRNOG00000005048 | 3.100743 | 0.013912 | up | 7:+:83113672-83153519 | 7:+:83113672-83153519 | Trhr |
| ENSRNOG00000024536 | -3.53305718832994 | 0.011619 | down | 18:-:61759289-61788863 | 18:-:61759289-61788863 | AABR07032338.1 |
| ENSRNOG00000023991 | 3.18569 | 0.012584 | up | 16:+:83358116-83382326 | 16:+:83358116-83382326 | Rab20 |
| ENSRNOG00000007687 | -1.82236111500232 | 0.018838 | down | 8:+:62723788-62745868 | 8:+:62723788-62745868 | Sema7a |
| ENSRNOG00000001825 | -2.83213574623017 | 0.012326 | down | 11:-:88912599-88972176 | 11:-:88912599-88972176 | AABR07034767.1 |
| ENSRNOG00000020533 | 1.144155 | 0.018719 | up | 1:+:201499028-201548513 | 1:+:201499028-201548513 | Htra1 |
| ENSRNOG00000059500 | 1.39959 | 0.019855 | up | 1:-:216661080-216663721 | 1:-:216661080-216663721 | Cdkn1c |
| ENSRNOG00000019573 | 1.912255 | 0.020034 | up | 19:-:37913336-37916813 | 19:-:37913336-37916813 | Lcat |
| ENSRNOG00000016239 | 1.599089 | 0.019891 | up | 18:+:80939875-80949226 | 18:+:80939875-80949226 | Zadh2 |
| ENSRNOG00000029366 | -1.73985937269523 | 0.0193 | down | 1:-:198451324-198454914 | 1:-:198451324-198454914 | Prrt2 |
| ENSRNOG00000045743 | 1.739975 | 0.020226 | up | 2:+:235738416-235760352 | 2:+:235738416-235760352 | Etnppl |
| ENSRNOG00000033202 | -2.13643216288878 | 0.019888 | down | 4:-:117256770-117268178 | 4:-:117256770-117268178 | Fbxo41 |
| ENSRNOG00000004269 | -2.07829964632622 | 0.019652 | down | 6:+:48452369-48857936 | 6:+:48452369-48857936 | Myt1l |
| ENSRNOG00000006509 | -2.2779810424652 | 0.019586 | down | 4:-:144638335-144869919 | 4:-:144638335-144869919 | Srgap3 |
| ENSRNOG00000024506 | 2.076965 | 0.01765 | up | 5:+:78384444-78409050 | 5:+:78384444-78409050 | LOC500475 |
| ENSRNOG00000024728 | 1.928965 | 0.019812 | up | 16:+:9486832-9613528 | 16:+:9486832-9613528 | Arhgap22 |
| ENSRNOG00000019999 | 3.66013 | 0.012644 | up | 1:+:81499821-81504318 | 1:+:81499821-81504318 | Lypd3 |
| ENSRNOG00000039473 | 1.58933 | 0.020215 | up | 2:-:53116101-53140637 | 2:-:53116101-53140637 | Ccdc152 |
| ENSRNOG00000046242 | -1.48219534266421 | 0.020674 | down | 9:-:70740480-70787913 | 9:-:70740480-70787913 | Klf7 |
| ENSRNOG00000049918 | 2.426231 | 0.019769 | up | 9:+:10952374-10954681 | 9:+:10952374-10954681 | Lrg1 |
| ENSRNOG00000018317 | -1.11661960176382 | 0.019431 | down | 4:+:118655728-118795774 | 4:+:118655728-118795774 | Aak1 |
| ENSRNOG00000036475 | 2.156127 | 0.014673 | up | 5:+:115225263-115225366 | 5:+:115225263-115225366 | AABR07049302.1 |
| ENSRNOG00000048723 | 1.180094 | 0.020839 | up | 7:+:1206648-1288134 | 7:+:1206648-1288134 | Pros1 |
| ENSRNOG00000019453 | 1.387423 | 0.01968 | up | 1:-:65660470-65664767 | 1:-:65660470-65664767 | Rps5 |
| ENSRNOG00000019440 | 3.503267 | 0.014332 | up | 1:+:81230612-81245996 | 1:+:81230612-81245996 | Kcnn4 |
| ENSRNOG00000019902 | 3.526893 | 0.012586 | up | 1:-:166934460-166943592 | 1:-:166934460-166943592 | Folr1 |
| ENSRNOG00000017118 | -3.43772746598753 | 0.014653 | down | 1:+:165295847-165330322 | 1:+:165295847-165330322 | P4ha3 |
| ENSRNOG00000052157 | -2.95044082638211 | 0.020741 | down | 7:-:52165233-52404774 | 7:-:52165233-52404774 | Nav3 |
| ENSRNOG00000007666 | -2.76760431495529 | 0.020332 | down | 3:-:37955069-38090526 | 3:-:37955069-38090526 | Cacnb4 |
| ENSRNOG00000005708 | -1.27392468960908 | 0.021014 | down | 5:+:31568419-31810484 | 5:+:31568419-31810484 | Mmp16 |
| ENSRNOG00000021984 | -1.04737949919704 | 0.020116 | down | 13:-:93095200-93307199 | 13:-:93095200-93307199 | Rgs7 |
| ENSRNOG00000005390 | -1.33047490818888 | 0.020961 | down | 4:-:122644135-122741110 | 4:-:122644135-122741110 | Nup210 |
| ENSRNOG00000055295 | 1.129864 | 0.020887 | up | 9:-:79601901-79630536 | 9:-:79601901-79630536 | Pecr |
| ENSRNOG00000020650 | -2.20501137373687 | 0.019393 | down | 1:+:101161252-101173174 | 1:+:101161252-101173174 | Slc17a7 |
| ENSRNOG00000012290 | 4.082104 | 0.016272 | up | 3:+:110975923-110979957 | 3:+:110975923-110979957 | Gchfr |
| ENSRNOG00000007159 | 3.390795 | 0.012759 | up | 10:+:69412017-69413870 | 10:+:69412017-69413870 | Ccl2 |
| ENSRNOG00000012148 | -1.91453266177707 | 0.0202 | down | 2:-:80473202-80667481 | 2:-:80473202-80667481 | Trio |
| ENSRNOG00000012236 | 1.506388 | 0.020934 | up | 1:+:142136452-142138737 | 1:+:142136452-142138737 | Hddc3 |
| ENSRNOG00000017401 | 1.795837 | 0.018846 | up | 5:+:157423213-157501737 | 5:+:157423213-157501737 | Tmco4 |
| ENSRNOG00000003285 | -3.55239703960518 | 0.015354 | down | 10:+:3321476-3357470 | 10:+:3321476-3357470 | Mpv17l |
| ENSRNOG00000002983 | -3.47761176041717 | 0.021279 | down | 19:-:25821780-25914696 | 19:-:25821780-25914696 | Nfix |
| ENSRNOG00000020481 | 1.789206 | 0.020901 | up | 1:-:82163595-82166363 | 1:-:82163595-82166363 | Pafah1b3 |
| ENSRNOG00000029195 | 1.930053 | 0.018516 | up | 8:+:116754178-116764126 | 8:+:116754178-116764126 | Uba7 |
| ENSRNOG00000016358 | -1.01321709521323 | 0.02029 | down | 1:+:125367280-125548890 | 1:+:125367280-125548890 | Apba2 |
| ENSRNOG00000005185 | -2.6127387484663 | 0.021463 | down | 10:-:83328410-83332851 | 10:-:83328410-83332851 | Nxph3 |
| ENSRNOG00000007350 | 1.904439 | 0.019157 | up | 7:-:119783849-119797098 | 7:-:119783849-119797098 | Rac2 |
| ENSRNOG00000046250 | -3.48973984684065 | 0.013736 | down | 1:-:54719971-54748763 | 1:-:54719971-54748763 | LOC100911027 |
| ENSRNOG00000012073 | 1.189591 | 0.021386 | up | 8:+:115150514-115154550 | 8:+:115150514-115154550 | Abhd14b |
| ENSRNOG00000015941 | 1.38227 | 0.021454 | up | 10:+:88326080-88338207 | 10:+:88326080-88338207 | Fkbp10 |
| ENSRNOG00000022635 | -1.97711376118181 | 0.020718 | down | 1:+:156552328-157269747 | 1:+:156552328-157269747 | Dlg2 |
| ENSRNOG00000028879 | -4.07802158986432 | 0.019058 | down | 7:+:142397371-142464158 | 7:+:142397371-142464158 | Slc4a8 |
| ENSRNOG00000003510 | 2.732508 | 0.014367 | up | 13:-:80755058-80775264 | 13:-:80755058-80775264 | Fmo2 |
| ENSRNOG00000003622 | 1.673536 | 0.018779 | up | X:+:14578264-14612547 | X:+:14578264-14612547 | Cybb |
| ENSRNOG00000051948 | -1.90792107438658 | 0.02185 | down | X:+:156812064-156837227 | X:+:156812064-156837227 | Hcfc1 |
| ENSRNOG00000048824 | -2.43328124347664 | 0.020392 | down | 18:-:73831326-73873280 | 18:-:73831326-73873280 | Rnf165 |
| ENSRNOG00000009225 | 2.696045 | 0.01633 | up | 10:+:84718824-84731880 | 10:+:84718824-84731880 | Copz2 |
| ENSRNOG00000029740 | 2.484021 | 0.012863 | up | 1:+:257076221-257082655 | 1:+:257076221-257082655 | Slc35g1 |
| ENSRNOG00000017749 | 3.678564 | 0.01426 | up | 1:-:98492828-98493978 | 1:-:98492828-98493978 | Nkg7 |
| ENSRNOG00000018730 | 1.425674 | 0.02218 | up | 1:-:80631450-80666585 | 1:-:80631450-80666585 | Nectin2 |
| ENSRNOG00000004624 | 1.89344 | 0.021813 | up | 3:-:36642587-36660758 | 3:-:36642587-36660758 | Rnd3 |
| ENSRNOG00000049403 | -2.34788841480023 | 0.016882 | down | 13:-:43494350-43566680 | 13:-:43494350-43566680 | LOC108348294 |
| ENSRNOG00000017803 | 1.496427 | 0.022121 | up | 17:-:90099025-90149894 | 17:-:90099025-90149894 | Apbb1ip |
| ENSRNOG00000013042 | -3.92890536719354 | 0.01815 | down | 8:-:89129453-89130991 | 8:-:89129453-89130991 | Htr1b |
| ENSRNOG00000008301 | 1.131409 | 0.021868 | up | 13:-:90807199-90814119 | 13:-:90807199-90814119 | Tagln2 |
| ENSRNOG00000007032 | 2.004172 | 0.022232 | up | 7:-:138704126-138707221 | 7:-:138704126-138707221 | Amigo2 |
| ENSRNOG00000004411 | 1.626107 | 0.020361 | up | 7:+:58814805-58847563 | 7:+:58814805-58847563 | Tspan8 |
| ENSRNOG00000014258 | 1.743575 | 0.019233 | up | 1:-:4637491-4653210 | 1:-:4637491-4653210 | Rab32 |
| ENSRNOG00000047880 | -3.65248147547493 | 0.016828 | down | 12:+:18074033-18082197 | 12:+:18074033-18082197 | LOC103689975 |
| ENSRNOG00000032844 | 2.634337 | 0.020863 | up | 20:-:4127644-4132616 | 20:-:4127644-4132616 | RT1-Da |
| ENSRNOG00000008098 | -1.19643711314075 | 0.021483 | down | 4:-:56110658-56114254 | 4:-:56110658-56114254 | Lrrc4 |
| ENSRNOG00000005278 | -1.66991947673652 | 0.021972 | down | 7:-:57253023-57679795 | 7:-:57253023-57679795 | Trhde |
| ENSRNOG00000023085 | 1.990558 | 0.021319 | up | 7:+:3133506-3143911 | 7:+:3133506-3143911 | Pmel |
| ENSRNOG00000051078 | -2.67274913145701 | 0.013498 | down | 5:-:77941148-77945016 | 5:-:77941148-77945016 | Zfp37 |
| ENSRNOG00000001049 | 1.047893 | 0.020802 | up | 15:+:57891680-57894504 | 15:+:57891680-57894504 | Tpt1 |
| ENSRNOG00000011931 | -1.33846050447014 | 0.021382 | down | 1:+:244615821-244782706 | 1:+:244615821-244782706 | Smarca2 |
| ENSRNOG00000060105 | -1.80834672888099 | 0.022284 | down | 5:-:81179605-82168427 | 5:-:81179605-82168427 | Astn2 |
| ENSRNOG00000000219 | 1.12894 | 0.021753 | up | 8:-:130813214-130930990 | 8:-:130813214-130930990 | Ano10 |
| ENSRNOG00000015415 | 1.324684 | 0.022098 | up | 6:+:10533151-10568581 | 6:+:10533151-10568581 | Rhoq |
| ENSRNOG00000001964 | -1.11138101163608 | 0.021722 | down | 11:-:53514595-53575175 | 11:-:53514595-53575175 | Cd47 |
| ENSRNOG00000002886 | -1.15681585295527 | 0.02108 | down | 10:+:55275411-55406732 | 10:+:55275411-55406732 | Myh10 |
| ENSRNOG00000007030 | -1.65650617276366 | 0.022544 | down | 5:+:43603043-43757549 | 5:+:43603043-43757549 | Epha7 |
| ENSRNOG00000020845 | 2.380928 | 0.021615 | up | 1:+:88875375-88879303 | 1:+:88875375-88879303 | Tyrobp |
| ENSRNOG00000052450 | 3.136656 | 0.016003 | up | 7:+:144725291-144725621 | 7:+:144725291-144725621 | AABR07058936.1 |
| ENSRNOG00000017516 | -1.01714998096586 | 0.022335 | down | 2:-:199334664-199354793 | 2:-:199334664-199354793 | Bcl9 |
| ENSRNOG00000038436 | -1.34654452334555 | 0.02154 | down | 2:+:123555673-123766676 | 2:+:123555673-123766676 | RGD1307100 |
| ENSRNOG00000014385 | 2.681992 | 0.015299 | up | 2:-:207440886-207455258 | 2:-:207440886-207455258 | Wnt2b |
| ENSRNOG00000025936 | 1.08495 | 0.021579 | up | 12:-:40877613-40881124 | 12:-:40877613-40881124 | Rpl6 |
| ENSRNOG00000013428 | -1.94079559725308 | 0.019695 | down | 4:-:65736585-65818521 | 4:-:65736585-65818521 | Atp6v0a4 |
| ENSRNOG00000007686 | -1.40442110435163 | 0.021982 | down | 7:+:123510804-123526542 | 7:+:123510804-123526542 | Septin3 |
| ENSRNOG00000012989 | -2.1492350233613 | 0.021232 | down | 5:-:148470061-148492232 | 5:-:148470061-148492232 | Serinc2 |
| ENSRNOG00000001960 | 2.620996 | 0.021154 | up | 14:+:22091777-22126021 | 14:+:22091777-22126021 | Sult1d1 |
| ENSRNOG00000004527 | -2.46616452483227 | 0.021296 | down | 7:+:49385705-49656856 | 7:+:49385705-49656856 | Lin7a |
| ENSRNOG00000042980 | -1.15683464080029 | 0.023303 | down | 10:+:31146107-31240582 | 10:+:31146107-31240582 | Adam19 |
| ENSRNOG00000007763 | 1.004864 | 0.023074 | up | 5:-:164720587-164747083 | 5:-:164720587-164747083 | Plod1 |
| ENSRNOG00000049385 | 2.054898 | 0.018935 | up | 2:-:197803584-197814808 | 2:-:197803584-197814808 | Adamtsl4 |
| ENSRNOG00000008595 | 2.233519 | 0.018107 | up | 8:-:53770681-53816447 | 8:-:53770681-53816447 | Ttc12 |
| ENSRNOG00000011647 | 3.090129 | 0.016756 | up | 2:+:190007216-190008511 | 2:+:190007216-190008511 | S100a6 |
| ENSRNOG00000030019 | -1.26375818378596 | 0.021524 | down | 2:-:204003742-204032023 | 2:-:204003742-204032023 | Atp1a1 |
| ENSRNOG00000037198 | 3.313274 | 0.019556 | up | 4:+:153805993-153834430 | 4:+:153805993-153834430 | Usp18 |
| ENSRNOG00000020577 | -1.19807104083079 | 0.021945 | down | 7:-:11267218-11294249 | 7:-:11267218-11294249 | Pip5k1c |
| ENSRNOG00000027596 | -1.20126534257642 | 0.023257 | down | 3:+:33440191-33504238 | 3:+:33440191-33504238 | Mbd5 |
| ENSRNOG00000023116 | 1.59045 | 0.023001 | up | 6:+:56846789-57193961 | 6:+:56846789-57193961 | Agmo |
| ENSRNOG00000003809 | 1.216738 | 0.022851 | up | X:+:43625169-43629765 | X:+:43625169-43629765 | Sat1 |
| ENSRNOG00000020863 | 2.98421 | 0.01673 | up | 2:-:195676048-195678848 | 2:-:195676048-195678848 | Oaz3 |
| ENSRNOG00000062168 | 3.838637 | 0.017824 | up | 2:+:187902007-187902546 | 2:+:187902007-187902546 | AC119762.8 |
| ENSRNOG00000017044 | -4.17316981850598 | 0.016658 | down | 3:-:2832847-2841331 | 3:-:2832847-2841331 | Ccdc183 |
| ENSRNOG00000017408 | -1.13369272374312 | 0.023317 | down | 1:-:170212817-170238890 | 1:-:170212817-170238890 | Fam160a2 |
| ENSRNOG00000051615 | 1.232794 | 0.022806 | up | 5:-:152195361-152198813 | 5:-:152195361-152198813 | Hmgn2 |
| ENSRNOG00000026124 | 1.957713 | 0.023538 | up | 5:+:154489590-154491223 | 5:+:154489590-154491223 | Id3 |
| ENSRNOG00000021206 | 1.395377 | 0.02345 | up | 1:+:222844144-222877193 | 1:+:222844144-222877193 | Plaat3 |
| ENSRNOG00000014233 | 3.662181 | 0.016292 | up | 10:-:88055841-88060561 | 10:-:88055841-88060561 | Krt19 |
| ENSRNOG00000015268 | -1.21150437277968 | 0.023353 | down | 1:+:247084228-247087077 | 1:+:247084228-247087077 | Plpp6 |
| ENSRNOG00000016753 | 1.404096 | 0.023521 | up | 18:-:74461055-74485139 | 18:-:74461055-74485139 | Slc14a1 |
| ENSRNOG00000002800 | 2.81769 | 0.02178 | up | X:+:70563570-70572295 | X:+:70563570-70572295 | Gdpd2 |
| ENSRNOG00000009779 | 3.479263 | 0.018365 | up | 7:-:143596511-143603803 | 7:-:143596511-143603803 | Krt8 |
| ENSRNOG00000004688 | -1.50546486222545 | 0.024011 | down | 7:-:118156946-118396728 | 7:-:118156946-118396728 | Rbfox2 |
| ENSRNOG00000031041 | 2.026154 | 0.024391 | up | 4:+:182745448-182746381 | 4:+:182745448-182746381 | Rps4y2 |
| ENSRNOG00000007266 | 3.622629 | 0.016737 | up | 8:+:57983556-58011962 | 8:+:57983556-58011962 | RGD1311251 |
| ENSRNOG00000001422 | -3.07093781462149 | 0.01637 | down | 12:+:22835019-22980603 | 12:+:22835019-22980603 | Col26a1 |
| ENSRNOG00000048139 | 3.442836 | 0.017773 | up | 5:-:153729250-153737161 | 5:-:153729250-153737161 | Ncmap |
| ENSRNOG00000024221 | 2.791603 | 0.016279 | up | 20:-:48586325-48706793 | 20:-:48586325-48706793 | Mettl24 |
| ENSRNOG00000002013 | -2.14503830012444 | 0.023956 | down | 11:+:61321459-61417842 | 11:+:61321459-61417842 | Sidt1 |
| ENSRNOG00000014082 | 2.919174 | 0.022653 | up | 7:-:143958858-143967484 | 7:-:143958858-143967484 | Sp7 |
| ENSRNOG00000062277 | -2.53374700344559 | 0.01585 | down | 12:-:30495542-30496547 | 12:-:30495542-30496547 | AABR07036010.4 |
| ENSRNOG00000012531 | -2.17475706787106 | 0.025224 | down | 5:-:155022493-155204456 | 5:-:155022493-155204456 | Ephb2 |
| ENSRNOG00000002149 | 4.162002 | 0.018014 | up | 14:+:9555264-9562189 | 14:+:9555264-9562189 | Nkx6-1 |
| ENSRNOG00000020424 | 1.195847 | 0.025271 | up | 1:+:202432366-202560628 | 1:+:202432366-202560628 | Plpp4 |
| ENSRNOG00000008855 | 1.460496 | 0.025012 | up | 15:-:37377316-37383277 | 15:-:37377316-37383277 | Gjb2 |
| ENSRNOG00000015447 | -2.21956010873768 | 0.025216 | down | 7:-:145435584-145450301 | 7:-:145435584-145450301 | Calcoco1 |
| ENSRNOG00000047080 | 3.578452 | 0.024072 | up | 17:-:90266794-90315492 | 17:-:90266794-90315492 | Gng4 |
| ENSRNOG00000011310 | -4.28751215913203 | 0.024409 | down | 1:-:52360296-52544450 | 1:-:52360296-52544450 | Pde10a |
| ENSRNOG00000032929 | -1.37028618602219 | 0.025593 | down | 1:-:225925773-225952516 | 1:-:225925773-225952516 | Incenp |
| ENSRNOG00000009949 | 1.55866 | 0.025623 | up | 2:-:138819960-138833933 | 2:-:138819960-138833933 | Pcdh18 |
| ENSRNOG00000013072 | -4.24878138488492 | 0.024389 | down | 4:-:59445767-59809321 | 4:-:59445767-59809321 | Plxna4 |
| ENSRNOG00000015441 | 1.204825 | 0.02565 | up | 1:+:196942364-196967220 | 1:+:196942364-196967220 | Il4r |
| ENSRNOG00000017503 | -3.87583978347779 | 0.022111 | down | 18:-:56626712-56728968 | 18:-:56626712-56728968 | Ppargc1b |
| ENSRNOG00000048315 | 1.183883 | 0.025209 | up | 6:-:1428834-1466201 | 6:-:1428834-1466201 | Eif2ak2 |
| ENSRNOG00000019306 | -1.39793990113373 | 0.024529 | down | 1:-:219688451-219719272 | 1:-:219688451-219719272 | Syt12 |
| ENSRNOG00000024705 | 1.805175 | 0.025806 | up | 4:-:78205812-78208767 | 4:-:78205812-78208767 | Rarres2 |
| ENSRNOG00000026793 | -2.63890044624243 | 0.025878 | down | 7:+:27309966-27363394 | 7:+:27309966-27363394 | Nt5dc3 |
| ENSRNOG00000033973 | -1.52332023890394 | 0.025887 | down | 6:-:104406897-104409005 | 6:-:104406897-104409005 | Ccdc177 |
| ENSRNOG00000019533 | -3.54576556506081 | 0.021584 | down | 17:+:6935860-7029240 | 17:+:6935860-7029240 | Klhl3 |
| ENSRNOG00000001141 | -3.97352379690822 | 0.022854 | down | 12:+:45727112-45882548 | 12:+:45727112-45882548 | Srrm4 |
| ENSRNOG00000016618 | -2.9688014859348 | 0.017173 | down | 5:+:172887217-172889383 | 5:+:172887217-172889383 | Tmem52 |
| ENSRNOG00000034025 | -1.39171773555845 | 0.024312 | down | 3:-:79233525-79390956 | 3:-:79233525-79390956 | Ptprj |
| ENSRNOG00000030719 | -2.17208280554622 | 0.025752 | down | 16:+:78539489-78850222 | 16:+:78539489-78850222 | Csmd1 |
| ENSRNOG00000042321 | -2.44646941109983 | 0.022672 | down | 3:-:64554633-64554953 | 3:-:64554633-64554953 | AABR07052588.1 |
| ENSRNOG00000042264 | -3.57187320031472 | 0.018254 | down | 18:+:30826260-30828641 | 18:+:30826260-30828641 | Pcdhgb1 |
| ENSRNOG00000037799 | -2.80163406061296 | 0.017506 | down | X:-:82875339-83151511 | X:-:82875339-83151511 | Hdx |
| ENSRNOG00000058006 | 2.738762 | 0.026337 | up | 16:-:10722106-10726707 | 16:-:10722106-10726707 | Sncg |
| ENSRNOG00000009207 | -1.28929841464531 | 0.025084 | down | 3:-:164230852-164239250 | 3:-:164230852-164239250 | Spata2 |
| ENSRNOG00000000569 | 1.45439 | 0.025625 | up | 20:+:29897594-29919998 | 20:+:29897594-29919998 | Vsir |
| ENSRNOG00000018836 | 1.039356 | 0.024426 | up | 19:-:57396606-57422093 | 19:-:57396606-57422093 | RGD1559896 |
| ENSRNOG00000038752 | -2.66076596101549 | 0.017301 | down | 2:-:102301406-102301790 | 2:-:102301406-102301790 | AABR07009538.1 |
| ENSRNOG00000011060 | 1.612686 | 0.026168 | up | 10:+:65606898-65612324 | 10:+:65606898-65612324 | Unc119 |
| ENSRNOG00000023633 | 5.439387 | 0.022552 | up | 8:+:59344083-59352132 | 8:+:59344083-59352132 | Crabp1 |
| ENSRNOG00000012325 | 1.044489 | 0.024765 | up | 15:-:3033495-3435888 | 15:-:3033495-3435888 | Adk |
| ENSRNOG00000059113 | -3.77951048364374 | 0.023944 | down | 15:-:87404841-87450319 | 15:-:87404841-87450319 | AABR07019085.1 |
| ENSRNOG00000005332 | 1.672227 | 0.024377 | up | 7:+:123168811-123183335 | 7:+:123168811-123183335 | Csdc2 |
| ENSRNOG00000008078 | 2.423185 | 0.025038 | up | 10:+:94207314-94228236 | 10:+:94207314-94228236 | Kcnh6 |
| ENSRNOG00000019203 | 3.589589 | 0.025059 | up | 3:+:162346490-162470065 | 3:+:162346490-162470065 | Eya2 |
| ENSRNOG00000025209 | -1.151220038066 | 0.025493 | down | 4:-:147854309-147893992 | 4:-:147854309-147893992 | Plxnd1 |
| ENSRNOG00000010208 | 2.473661 | 0.023701 | up | X:-:1364786-1369384 | X:-:1364786-1369384 | Timp1 |
| ENSRNOG00000042939 | 1.230682 | 0.026831 | up | 4:+:161720501-161729192 | 4:+:161720501-161729192 | Nrip2 |
| ENSRNOG00000023446 | -1.28963360097685 | 0.025743 | down | 14:-:3284089-3300200 | 14:-:3284089-3300200 | Btbd8 |
| ENSRNOG00000013232 | -2.68901706205094 | 0.017829 | down | 3:-:23033775-23066658 | 3:-:23033775-23066658 | Nr6a1 |
| ENSRNOG00000006508 | 1.754023 | 0.026113 | up | 4:-:98976550-98995590 | 4:-:98976550-98995590 | Thnsl2 |
| ENSRNOG00000001048 | -2.64493338625174 | 0.017811 | down | 12:+:12756452-12760838 | 12:+:12756452-12760838 | Ankrd61 |
| ENSRNOG00000037951 | -2.19611625287835 | 0.026404 | down | X:+:71960851-71972810 | X:+:71960851-71972810 | AABR07039210.1 |
| ENSRNOG00000007066 | -3.72055521885676 | 0.023094 | down | 4:+:199916-209599 | 4:+:199916-209599 | Htr5a |
| ENSRNOG00000009180 | -4.62365597238532 | 0.02264 | down | 3:+:148510779-148533404 | 3:+:148510779-148533404 | Xkr7 |
| ENSRNOG00000033863 | -2.52272964371553 | 0.025455 | down | 3:-:2800650-2803574 | 3:-:2800650-2803574 | Ajm1 |
| ENSRNOG00000012490 | -1.24657520925714 | 0.02682 | down | 17:-:48304322-48562905 | 17:-:48304322-48562905 | Amph |
| ENSRNOG00000012988 | 1.361382 | 0.02525 | up | 1:+:59156251-59214452 | 1:+:59156251-59214452 | Lix1 |
| ENSRNOG00000021062 | 1.681773 | 0.02718 | up | 1:-:89464860-89474252 | 1:-:89464860-89474252 | Fxyd5 |
| ENSRNOG00000023720 | -1.04951034800615 | 0.025117 | down | 8:-:30039408-30222036 | 8:-:30039408-30222036 | Ntm |
| ENSRNOG00000015781 | -4.31439697428863 | 0.023889 | down | 2:-:227738650-227890077 | 2:-:227738650-227890077 | Ndst3 |
| ENSRNOG00000018378 | -1.87425916841652 | 0.02714 | down | 17:+:81798756-82017682 | 17:+:81798756-82017682 | Cacnb2 |
| ENSRNOG00000058243 | 2.883217 | 0.019201 | up | 9:-:46414564-46475040 | 9:-:46414564-46475040 | Rfx8 |
| ENSRNOG00000018795 | 1.445704 | 0.024943 | up | 16:+:20293229-20295294 | 16:+:20293229-20295294 | Rpl18a |
| ENSRNOG00000015078 | 2.041768 | 0.027432 | up | 1:-:213810696-213811901 | 1:-:213810696-213811901 | Ifitm3 |
| ENSRNOG00000011858 | -3.63877854204333 | 0.023975 | down | 16:+:67350539-67595225 | 16:+:67350539-67595225 | Unc5d |
| ENSRNOG00000015003 | 1.046656 | 0.027485 | up | 1:-:141474180-141481315 | 1:-:141474180-141481315 | Pex11a |
| ENSRNOG00000016208 | -3.68439051652814 | 0.024787 | down | 18:-:75095632-75207306 | 18:-:75095632-75207306 | Setbp1 |
| ENSRNOG00000021157 | 1.312758 | 0.025534 | up | 2:+:197655786-197679458 | 2:+:197655786-197679458 | Ctss |
| ENSRNOG00000008862 | -2.31094723796528 | 0.02796 | down | 8:-:48606403-48619592 | 8:-:48606403-48619592 | Abcg4 |
| ENSRNOG00000053424 | -3.12320151181732 | 0.019303 | down | 1:+:260832285-260832421 | 1:+:260832285-260832421 | AABR07006860.1 |
| ENSRNOG00000046666 | -2.85515603892461 | 0.019119 | down | 18:-:74187162-74198369 | 18:-:74187162-74198369 | Haus1 |
| ENSRNOG00000046991 | -2.40189343880444 | 0.021549 | down | 11:-:39766584-39767802 | 11:-:39766584-39767802 | RGD1565472 |
| ENSRNOG00000001010 | -1.34334452490721 | 0.026131 | down | 12:+:12374790-12402552 | 12:+:12374790-12402552 | Tecpr1 |
| ENSRNOG00000017628 | 1.773759 | 0.02715 | up | 8:-:50222896-50231357 | 8:-:50222896-50231357 | Tagln |
| ENSRNOG00000005960 | 1.715967 | 0.028011 | up | 8:-:43315592-43336304 | 8:-:43315592-43336304 | RGD1311744 |
| ENSRNOG00000060463 | -4.16338670369345 | 0.027071 | down | 6:+:9483594-9491667 | 6:+:9483594-9491667 | AABR07062799.2 |
| ENSRNOG00000061538 | -3.92584819032131 | 0.020488 | down | 5:+:143189409-143194764 | 5:+:143189409-143194764 | AABR07049892.1 |
| ENSRNOG00000026647 | 1.336498 | 0.02815 | up | 10:-:57060007-57064600 | 10:-:57060007-57064600 | Cxcl16 |
| ENSRNOG00000001148 | 1.09845 | 0.025389 | up | 12:-:46791528-46794797 | 12:-:46791528-46794797 | Rplp0 |
| ENSRNOG00000032410 | -1.77546439216892 | 0.027751 | down | 12:-:8111667-8418966 | 12:-:8111667-8418966 | Mtus2 |
| ENSRNOG00000009288 | -3.60238777798332 | 0.025163 | down | 15:-:110385217-110612681 | 15:-:110385217-110612681 | Fgf14 |
| ENSRNOG00000008992 | -4.24564257970785 | 0.027576 | down | 5:+:143500441-143715546 | 5:+:143500441-143715546 | Grik3 |
| ENSRNOG00000029141 | -3.4171158033214 | 0.022768 | down | 5:+:133221139-133420644 | 5:+:133221139-133420644 | Trabd2b |
| ENSRNOG00000023830 | 1.58623 | 0.02879 | up | 19:+:26022849-26025532 | 19:+:26022849-26025532 | Dnase2 |
| ENSRNOG00000000816 | 1.157385 | 0.028732 | up | 20:-:3372413-3397039 | 20:-:3372413-3397039 | Ppp1r18 |
| ENSRNOG00000007706 | -3.689665622617 | 0.025887 | down | 5:-:124574079-124642569 | 5:-:124574079-124642569 | Prkaa2 |
| ENSRNOG00000018452 | -2.52094032347738 | 0.026956 | down | 16:-:20056765-20097287 | 16:-:20056765-20097287 | Unc13a |
| ENSRNOG00000027433 | -3.62402022569205 | 0.021001 | down | 4:+:62019970-62030874 | 4:+:62019970-62030874 | LOC100910708 |
| ENSRNOG00000016451 | 3.030581 | 0.022196 | up | 2:-:186330298-186333805 | 2:-:186330298-186333805 | Cd1d1 |
| ENSRNOG00000008533 | -1.70966736721423 | 0.029068 | down | 7:-:114339434-114380613 | 7:-:114339434-114380613 | Ago2 |
| ENSRNOG00000018812 | 3.165001 | 0.029088 | up | 8:+:62283336-62284716 | 8:+:62283336-62284716 | Rpp25 |
| ENSRNOG00000027233 | -3.7548051607306 | 0.027022 | down | X:-:115627653-115908693 | X:-:115627653-115908693 | Trpc5 |
| ENSRNOG00000050735 | 1.570401 | 0.02772 | up | 20:-:56192390-56197025 | 20:-:56192390-56197025 | Cd99 |
| ENSRNOG00000050910 | 1.604902 | 0.029373 | up | 17:+:36690249-36694325 | 17:+:36690249-36694325 | LOC688583 |
| ENSRNOG00000057161 | -1.672794655824 | 0.027361 | down | 17:+:28825009-28829441 | 17:+:28825009-28829441 | AABR07027407.1 |
| ENSRNOG00000019850 | -1.11368060502214 | 0.027085 | down | 9:+:82571269-82628709 | 9:+:82571269-82628709 | Speg |
| ENSRNOG00000010626 | 2.985245 | 0.026421 | up | 10:+:105498728-105504393 | 10:+:105498728-105504393 | Sphk1 |
| ENSRNOG00000042560 | -1.73349404870624 | 0.029424 | down | 16:+:71058022-71075320 | 16:+:71058022-71075320 | Bag4 |
| ENSRNOG00000046094 | 2.608815 | 0.024826 | up | 1:+:214446659-214450666 | 1:+:214446659-214450666 | Cd151 |
| ENSRNOG00000053044 | 3.546924 | 0.022619 | up | 2:-:208225406-208225888 | 2:-:208225406-208225888 | AABR07012775.1 |
| ENSRNOG00000030021 | 2.945567 | 0.021885 | up | 10:-:70797124-70802782 | 10:-:70797124-70802782 | Ccl6 |
| ENSRNOG00000014490 | 2.649506 | 0.024695 | up | 2:+:240461505-240482145 | 2:+:240461505-240482145 | Bdh2 |
| ENSRNOG00000009378 | 1.229152 | 0.026407 | up | 8:+:69121682-69126805 | 8:+:69121682-69126805 | Rpl4 |
| ENSRNOG00000042289 | -2.86052311576021 | 0.028938 | down | 11:+:57207656-57260568 | 11:+:57207656-57260568 | Plcxd2 |
| ENSRNOG00000047261 | -2.5162477791729 | 0.026371 | down | 18:+:14756684-14964991 | 18:+:14756684-14964991 | AABR07031445.1 |
| ENSRNOG00000023529 | 1.326515 | 0.026984 | up | 14:-:2860965-2867397 | 14:-:2860965-2867397 | Rpl5 |
| ENSRNOG00000027276 | -3.74538919915347 | 0.027675 | down | 5:-:15747614-16140896 | 5:-:15747614-16140896 | Xkr4 |
| ENSRNOG00000049751 | -3.19836001351719 | 0.024882 | down | 17:-:53833023-53915076 | 17:-:53833023-53915076 | LOC100912163 |
| ENSRNOG00000009845 | 1.14974 | 0.028659 | up | 2:-:260124418-260148589 | 2:-:260124418-260148589 | Acadm |
| ENSRNOG00000014874 | -1.3015251100735 | 0.029104 | down | 14:+:81725513-81811142 | 14:+:81725513-81811142 | Zfyve28 |
| ENSRNOG00000022691 | -1.68036016060203 | 0.028692 | down | 18:+:73564247-73625137 | 18:+:73564247-73625137 | St8sia5 |
| ENSRNOG00000010691 | 1.793696 | 0.027019 | up | 19:-:830877-838099 | 19:-:830877-838099 | Cmtm3 |
| ENSRNOG00000026271 | -2.03957045488543 | 0.030481 | down | 5:-:73918747-73986373 | 5:-:73918747-73986373 | Tmem245 |
| ENSRNOG00000012333 | -1.9237772051567 | 0.028481 | down | 16:-:79696891-79700992 | 16:-:79696891-79700992 | Kbtbd11 |
| ENSRNOG00000039544 | 1.327447 | 0.030597 | up | X:-:15506724-15520712 | X:-:15506724-15520712 | Kcnd1 |
| ENSRNOG00000027592 | 2.830716 | 0.030201 | up | 4:-:171069191-171176581 | 4:-:171069191-171176581 | Rerg |
| ENSRNOG00000016352 | -1.53502219667291 | 0.029754 | down | 3:+:149935731-150038257 | 3:+:149935731-150038257 | Cbfa2t2 |
| ENSRNOG00000020583 | 1.191992 | 0.029166 | up | 1:-:101086877-101095594 | 1:-:101086877-101095594 | Fcgrt |
| ENSRNOG00000001117 | -1.21230939127035 | 0.030513 | down | 12:+:13734429-13747215 | 12:+:13734429-13747215 | Fbxl18 |
| ENSRNOG00000002922 | 1.474325 | 0.030207 | up | 10:+:48569555-48586365 | 10:+:48569555-48586365 | Adora2b |
| ENSRNOG00000023760 | -1.43458873437946 | 0.030726 | down | 9:-:71498293-71651512 | 9:-:71498293-71651512 | Plekhm3 |
| ENSRNOG00000017703 | 1.248608 | 0.03079 | up | 1:+:219144205-219183963 | 1:+:219144205-219183963 | Unc93b1 |
| ENSRNOG00000056838 | -2.92314819633571 | 0.02402 | down | 8:-:121652886-121662993 | 8:-:121652886-121662993 | AABR07071551.1 |
| ENSRNOG00000016472 | -1.4582938695742 | 0.030845 | down | 9:-:79782845-79898912 | 9:-:79782845-79898912 | Marchf4 |
| ENSRNOG00000053469 | 1.471578 | 0.030555 | up | 10:-:106767161-106781950 | 10:-:106767161-106781950 | AABR07030861.1 |
| ENSRNOG00000020028 | -1.60097754684091 | 0.030506 | down | 18:+:30592794-30595181 | 18:+:30592794-30595181 | Pcdhb22 |
| ENSRNOG00000042602 | -1.58392160086959 | 0.030372 | down | 2:-:89498123-89498395 | 2:-:89498123-89498395 | AABR07009224.1 |
| ENSRNOG00000003749 | -1.18263187760989 | 0.030917 | down | X:+:14498119-14534473 | X:+:14498119-14534473 | Xk |
| ENSRNOG00000009341 | -1.48079132180679 | 0.029417 | down | 5:+:139007642-139077377 | 5:+:139007642-139077377 | Hivep3 |
| ENSRNOG00000014948 | 2.720551 | 0.023592 | up | 19:+:52077109-52085496 | 19:+:52077109-52085496 | Osgin1 |
| ENSRNOG00000014276 | 1.854344 | 0.031169 | up | 1:+:257157264-257466064 | 1:+:257157264-257466064 | Plce1 |
| ENSRNOG00000001516 | -1.79568469750158 | 0.02841 | down | 3:+:58632476-58924038 | 3:+:58632476-58924038 | Rapgef4 |
| ENSRNOG00000011677 | -1.48507832449741 | 0.028254 | down | 9:+:60021534-60070552 | 9:+:60021534-60070552 | Slc39a10 |
| ENSRNOG00000011460 | -4.43356606770273 | 0.031255 | down | 1:-:13838707-13915594 | 1:-:13838707-13915594 | Arfgef3 |
| ENSRNOG00000054579 | -2.10796186014443 | 0.023558 | down | 2:+:250450371-250465561 | 2:+:250450371-250465561 | AABR07013689.1 |
| ENSRNOG00000021174 | 1.329575 | 0.03034 | up | 1:+:222310920-222451484 | 1:+:222310920-222451484 | Macrod1 |
| ENSRNOG00000051671 | -1.30775194542319 | 0.028201 | down | 8:+:72029489-72198358 | 8:+:72029489-72198358 | Herc1 |
| ENSRNOG00000056659 | -1.27815128569436 | 0.031239 | down | X:+:156655960-156705233 | X:+:156655960-156705233 | Mecp2 |
| ENSRNOG00000036813 | -1.82318958511916 | 0.028949 | down | 3:-:153005741-153042395 | 3:-:153005741-153042395 | Ndrg3 |
| ENSRNOG00000053064 | -2.73248266503606 | 0.023069 | down | 1:-:126386929-126403406 | 1:-:126386929-126403406 | AABR07004130.1 |
| ENSRNOG00000054990 | -2.54294228974263 | 0.022359 | down | 4:-:58408730-58485447 | 4:-:58408730-58485447 | AABR07060133.1 |
| ENSRNOG00000061515 | -2.28986480361077 | 0.031284 | down | 11:+:71796282-71808498 | 11:+:71796282-71808498 | Fbxo45 |
| ENSRNOG00000012747 | -1.696986805568 | 0.028804 | down | 17:+:7675531-7797863 | 17:+:7675531-7797863 | Spock1 |
| ENSRNOG00000014840 | -3.60750984361108 | 0.026374 | down | 1:+:235166718-235347937 | 1:+:235166718-235347937 | Gna14 |
| ENSRNOG00000021338 | -1.67134214499717 | 0.027976 | down | 1:-:226912612-226924244 | 1:-:226912612-226924244 | Tmem132a |
| ENSRNOG00000005159 | -2.18344632381586 | 0.031468 | down | 7:-:112673465-112833083 | 7:-:112673465-112833083 | Fam135b |
| ENSRNOG00000056290 | 5.191615 | 0.025243 | up | 4:-:103257811-103258134 | 4:-:103257811-103258134 | AABR07061052.1 |
| ENSRNOG00000023546 | 2.318887 | 0.031653 | up | 12:-:23839399-23841049 | 12:-:23839399-23841049 | Hspb1 |
| ENSRNOG00000047931 | 1.093537 | 0.027499 | up | X:+:28593405-28595395 | X:+:28593405-28595395 | Tmsb4x |
| ENSRNOG00000002265 | -3.16490826815698 | 0.025011 | down | 11:+:67188630-67258771 | 11:+:67188630-67258771 | Casr |
| ENSRNOG00000003479 | -1.44523330048896 | 0.031393 | down | 19:+:24044103-24265444 | 19:+:24044103-24265444 | Rnf150 |
| ENSRNOG00000014137 | 1.530527 | 0.030535 | up | 7:+:126096793-126176468 | 7:+:126096793-126176468 | Fbln1 |
| ENSRNOG00000006885 | 2.038687 | 0.031237 | up | 7:-:136967200-136997050 | 7:-:136967200-136997050 | Dbx2 |
| ENSRNOG00000009266 | 1.001383 | 0.029542 | up | 5:+:62071346-62094633 | 5:+:62071346-62094633 | Anp32b |
| ENSRNOG00000059373 | -2.38009291133354 | 0.026696 | down | 4:+:155088317-155092827 | 4:+:155088317-155092827 | LOC100909548 |
| ENSRNOG00000009137 | -1.3281578051142 | 0.030501 | down | 5:+:19284548-19311088 | 5:+:19284548-19311088 | Ubxn2b |
| ENSRNOG00000060961 | -3.3734694339499 | 0.028329 | down | 8:-:61430419-61436104 | 8:-:61430419-61436104 | AABR07070246.1 |
| ENSRNOG00000024382 | 1.963452 | 0.02779 | up | 13:+:89385859-89396051 | 13:+:89385859-89396051 | Fcgr3a |
| ENSRNOG00000048516 | -2.7452689437077 | 0.023732 | down | X:-:20806602-20807216 | X:-:20806602-20807216 | Fam156b |
| ENSRNOG00000047836 | 4.971169 | 0.025319 | up | 3:-:19320304-19320915 | 3:-:19320304-19320915 | RGD1565617 |
| ENSRNOG00000022218 | 2.304538 | 0.031554 | up | 2:-:256897772-256915569 | 2:-:256897772-256915569 | Ifi44 |
| ENSRNOG00000060951 | -2.99096499148824 | 0.023574 | down | 3:-:66403184-66436668 | 3:-:66403184-66436668 | AABR07052613.1 |
| ENSRNOG00000021104 | 2.166297 | 0.030101 | up | 1:-:101900733-101903910 | 1:-:101900733-101903910 | Emp3 |
| ENSRNOG00000043300 | 1.366946 | 0.029263 | up | 5:-:58018039-58019836 | 5:-:58018039-58019836 | Enho |
| ENSRNOG00000056565 | 2.786485 | 0.025054 | up | 4:+:732721-736212 | 4:+:732721-736212 | AABR07059002.1 |
| ENSRNOG00000023304 | -3.15400899845608 | 0.025748 | down | 13:+:98924962-98967735 | 13:+:98924962-98967735 | Lin9 |
| ENSRNOG00000058912 | -2.93410577489458 | 0.024889 | down | 20:+:7330250-7361559 | 20:+:7330250-7361559 | LOC499407 |
| ENSRNOG00000054751 | -2.09552109842958 | 0.031434 | down | 2:+:58534476-58590758 | 2:+:58534476-58590758 | Lmbrd2 |
| ENSRNOG00000027552 | 1.304273 | 0.032408 | up | 4:-:157735748-157743199 | 4:-:157735748-157743199 | Tapbpl |
| ENSRNOG00000004483 | -2.21595861804861 | 0.031598 | down | 7:+:59039720-59325947 | 7:+:59039720-59325947 | Ptprr |
| ENSRNOG00000008798 | 2.621215 | 0.024468 | up | 10:+:64952119-64964862 | 10:+:64952119-64964862 | Pipox |
| ENSRNOG00000017409 | 2.314009 | 0.032452 | up | 9:+:82033543-82047166 | 9:+:82033543-82047166 | Wnt6 |
| ENSRNOG00000052795 | 1.646938 | 0.032478 | up | 20:+:5646097-5711702 | 20:+:5646097-5711702 | Itpr3 |
| ENSRNOG00000017283 | -1.43599889885638 | 0.030127 | down | 3:+:3310954-3365340 | 3:+:3310954-3365340 | Kcnt1 |
| ENSRNOG00000028097 | -3.94414139860542 | 0.031585 | down | 2:+:115678344-115788687 | 2:+:115678344-115788687 | Slc7a14 |
| ENSRNOG00000051406 | -3.01391120049163 | 0.024938 | down | 18:+:4314467-4314748 | 18:+:4314467-4314748 | 7SK |
| ENSRNOG00000038746 | 2.743815 | 0.02481 | up | 8:-:54965583-54998864 | 8:-:54965583-54998864 | Bco2 |
| ENSRNOG00000007060 | 1.281157 | 0.032643 | up | 5:-:104984414-105010857 | 5:-:104984414-105010857 | Plin2 |
| ENSRNOG00000025895 | 1.119566 | 0.032222 | up | 9:-:55243255-55256340 | 9:-:55243255-55256340 | Cavin2 |
| ENSRNOG00000008168 | 1.770945 | 0.031791 | up | 4:-:151500957-151516894 | 4:-:151500957-151516894 | Wnt5b |
| ENSRNOG00000003038 | 1.155041 | 0.032798 | up | 13:-:83438493-83457888 | 13:-:83438493-83457888 | Sft2d2 |
| ENSRNOG00000048425 | 5.274887 | 0.026681 | up | 6:-:140879774-140880070 | 6:-:140879774-140880070 | AABR07065776.3 |
| ENSRNOG00000020298 | 1.647182 | 0.032033 | up | 1:+:199941161-199965191 | 1:+:199941161-199965191 | Bag3 |
| ENSRNOG00000052506 | 1.044919 | 0.030706 | up | 1:-:247144491-247169693 | 1:-:247144491-247169693 | Ak3 |
| ENSRNOG00000018645 | 1.321741 | 0.028996 | up | 8:+:128806129-128809986 | 8:+:128806129-128809986 | Rpsa |
| ENSRNOG00000020942 | 2.353369 | 0.030957 | up | 1:+:101517714-101540802 | 1:+:101517714-101540802 | Plekha4 |
| ENSRNOG00000047387 | -2.75964921428938 | 0.025381 | down | 1:-:59297294-59347472 | 1:-:59297294-59347472 | Lnpep |
| ENSRNOG00000022166 | -3.03353588737922 | 0.025599 | down | X:-:114131898-114232939 | X:-:114131898-114232939 | Ammecr1 |
| ENSRNOG00000010645 | 2.637701 | 0.026543 | up | 15:+:24141651-24165537 | 15:+:24141651-24165537 | Lgals3 |
| ENSRNOG00000005965 | 1.452388 | 0.032867 | up | 7:+:135803698-135832652 | 7:+:135803698-135832652 | Irak4 |
| ENSRNOG00000058166 | -3.0321083580521 | 0.026999 | down | 7:+:74350479-74406295 | 7:+:74350479-74406295 | AABR07057495.1 |
| ENSRNOG00000032018 | -3.81638656437919 | 0.028959 | down | 5:+:150080072-150080926 | 5:+:150080072-150080926 | Tmem200b |
| ENSRNOG00000051073 | -3.04349267818998 | 0.027062 | down | X:-:115599716-115625360 | X:-:115599716-115625360 | AABR07040953.1 |
| ENSRNOG00000004708 | 1.844034 | 0.031251 | up | 7:+:91588458-91593297 | 7:+:91588458-91593297 | Aard |
| ENSRNOG00000050675 | -2.91963287795882 | 0.0253 | down | 10:+:92628356-92638100 | 10:+:92628356-92638100 | Myl4 |
| ENSRNOG00000003872 | -2.08268902390932 | 0.026726 | down | 9:-:52894365-52912293 | 9:-:52894365-52912293 | NEWGENE_620180 |
| ENSRNOG00000001295 | 1.188299 | 0.028893 | up | 20:-:13130636-13142856 | 20:-:13130636-13142856 | S100b |
| ENSRNOG00000000459 | 3.069609 | 0.029658 | up | 20:-:3973420-3978845 | 20:-:3973420-3978845 | Psmb9 |
| ENSRNOG00000017689 | 1.484562 | 0.029908 | up | 16:-:6992062-7007287 | 16:-:6992062-7007287 | Itih3 |
| ENSRNOG00000012789 | -4.04360444540195 | 0.029361 | down | 19:-:55176258-55183557 | 19:-:55176258-55183557 | Mlnr |
| ENSRNOG00000011748 | 5.376316 | 0.027952 | up | 2:+:190003223-190005871 | 2:+:190003223-190005871 | S100a5 |
| ENSRNOG00000054782 | -1.26563801625212 | 0.029935 | down | 15:+:4209703-4236895 | 15:+:4209703-4236895 | Ppp3cb |
| ENSRNOG00000037563 | 2.270313 | 0.025985 | up | 10:-:56268720-56270640 | 10:-:56268720-56270640 | Cd68 |
| ENSRNOG00000054935 | -2.26743974945801 | 0.02817 | down | 7:+:142941435-142944169 | 7:+:142941435-142944169 | AC119007.4 |
| ENSRNOG00000004500 | 1.907523 | 0.031924 | up | 7:+:102586313-102591240 | 7:+:102586313-102591240 | Myc |
| ENSRNOG00000031244 | 1.039233 | 0.032759 | up | 18:-:4371051-4371899 | 18:-:4371051-4371899 | LOC108349606 |
| ENSRNOG00000057499 | -3.24253278742648 | 0.027766 | down | 9:-:110055894-110056257 | 9:-:110055894-110056257 | AABR07068587.1 |
| ENSRNOG00000010461 | 1.27805 | 0.0341 | up | 2:-:44903337-44907030 | 2:-:44903337-44907030 | Gpx8 |
| ENSRNOG00000016731 | 1.680482 | 0.033586 | up | 5:-:59016622-59025631 | 5:-:59016622-59025631 | Tpm2 |
| ENSRNOG00000005369 | -2.1388439186448 | 0.032186 | down | 3:+:41019898-41181070 | 3:+:41019898-41181070 | Kcnj3 |
| ENSRNOG00000022929 | -3.78284597984345 | 0.033291 | down | 2:+:62236577-62303919 | 2:+:62236577-62303919 | Mtmr12 |
| ENSRNOG00000005996 | -4.20664422768557 | 0.027838 | down | 3:-:15411939-15433252 | 3:-:15411939-15433252 | Lhx6 |
| ENSRNOG00000034177 | -3.46057157857923 | 0.029162 | down | 9:-:110057155-110225486 | 9:-:110057155-110225486 | Efna5 |
| ENSRNOG00000042888 | -1.39781084248244 | 0.033545 | down | 19:-:49093355-49198073 | 19:-:49093355-49198073 | Cdyl2 |
| ENSRNOG00000007654 | 1.366708 | 0.03323 | up | 7:+:69213147-69263142 | 7:+:69213147-69263142 | Lrig3 |
| ENSRNOG00000020614 | -3.02725168253839 | 0.02656 | down | 1:-:216702319-216703534 | 1:-:216702319-216703534 | Phlda2 |
| ENSRNOG00000010763 | 1.311136 | 0.033107 | up | 1:+:157920786-157922274 | 1:+:157920786-157922274 | Fam181b |
| ENSRNOG00000022505 | 4.350883 | 0.027085 | up | 17:+:43458553-43470378 | 17:+:43458553-43470378 | Slc17a4 |
| ENSRNOG00000057092 | 3.145315 | 0.028393 | up | 10:+:70411738-70435156 | 10:+:70411738-70435156 | Slfn4 |
| ENSRNOG00000021095 | 2.549401 | 0.026618 | up | 1:-:89502562-89509343 | 1:-:89502562-89509343 | Fxyd3 |
| ENSRNOG00000019482 | -1.54957074317002 | 0.030438 | down | 19:-:11513201-11669578 | 19:-:11513201-11669578 | Gnao1 |
| ENSRNOG00000017695 | 1.367234 | 0.0339 | up | 2:+:208738132-208739047 | 2:+:208738132-208739047 | AABR07012795.1 |
| ENSRNOG00000013766 | 1.325641 | 0.033597 | up | 18:+:70733872-70762395 | 18:+:70733872-70762395 | Acaa2 |
| ENSRNOG00000013548 | -2.86020407781057 | 0.034615 | down | 1:-:77533148-77535681 | 1:-:77533148-77535681 | Selenow |
| ENSRNOG00000015113 | 2.977614 | 0.027022 | up | 18:+:16590197-16636084 | 18:+:16590197-16636084 | Mocos |
| ENSRNOG00000029876 | -3.6721577632404 | 0.033647 | down | 8:+:408001-899974 | 8:+:408001-899974 | Gucy1a2 |
| ENSRNOG00000052572 | -1.12746901986781 | 0.033248 | down | X:-:153539668-154051181 | X:-:153539668-154051181 | Aff2 |
| ENSRNOG00000011623 | -1.44025605904504 | 0.033053 | down | 2:-:41570597-41785792 | 2:-:41570597-41785792 | Rab3c |
| ENSRNOG00000053577 | -1.73035680898377 | 0.03239 | down | 8:-:44165367-44327551 | 8:-:44165367-44327551 | Gramd1b |
| ENSRNOG00000047641 | 4.267951 | 0.027519 | up | 6:-:138764502-138764901 | 6:-:138764502-138764901 | AABR07065651.3 |
| ENSRNOG00000028801 | 3.171842 | 0.031439 | up | 4:-:10423139-10517848 | 4:-:10423139-10517848 | Gsap |
| ENSRNOG00000003742 | -2.59853661747786 | 0.03453 | down | X:+:35599258-35771711 | X:+:35599258-35771711 | Cdkl5 |
| ENSRNOG00000024272 | -3.28652812633926 | 0.031868 | down | 9:-:69830424-69878706 | 9:-:69830424-69878706 | Ino80d |
| ENSRNOG00000048979 | 3.119698 | 0.028154 | up | 7:+:14821691-14828517 | 7:+:14821691-14828517 | LOC691320 |
| ENSRNOG00000058329 | 2.328034 | 0.027169 | up | 3:+:9681871-9718051 | 3:+:9681871-9718051 | Prrx2 |
| ENSRNOG00000020073 | -3.48696219172581 | 0.032392 | down | 18:+:30435119-30437458 | 18:+:30435119-30437458 | Pcdhb8 |
| ENSRNOG00000011321 | 1.440385 | 0.034916 | up | 9:+:12740885-12942358 | 9:+:12740885-12942358 | Rftn1 |
| ENSRNOG00000036604 | 1.619535 | 0.035188 | up | 1:+:252894663-252900726 | 1:+:252894663-252900726 | Ifit2 |
| ENSRNOG00000039025 | 1.297588 | 0.034111 | up | 3:-:29861224-29861621 | 3:-:29861224-29861621 | AABR07051947.1 |
| ENSRNOG00000058352 | 4.36867 | 0.028058 | up | 6:+:139486775-139487576 | 6:+:139486775-139487576 | AABR07065699.4 |
| ENSRNOG00000008360 | -2.79008168836251 | 0.029953 | down | 4:+:113910685-113913687 | 4:+:113910685-113913687 | Pcgf1 |
| ENSRNOG00000001011 | 2.76714 | 0.030719 | up | 12:-:37038733-37047628 | 12:-:37038733-37047628 | Rflna |
| ENSRNOG00000007675 | -2.63624427277111 | 0.034511 | down | 5:+:39329423-39370900 | 5:+:39329423-39370900 | Gpr63 |
| ENSRNOG00000030930 | 2.983745 | 0.028194 | up | 11:-:14253848-14304725 | 11:-:14253848-14304725 | Samsn1 |
| ENSRNOG00000017037 | -1.43750114790234 | 0.034038 | down | 5:-:157346286-157368472 | 5:-:157346286-157368472 | Otud3 |
| ENSRNOG00000020678 | 2.212977 | 0.035357 | up | 10:+:89358376-89366626 | 10:+:89358376-89366626 | Ifi35 |
| ENSRNOG00000061431 | 5.30992 | 0.029732 | up | 4:+:106323089-106323738 | 4:+:106323089-106323738 | AABR07061134.1 |
| ENSRNOG00000022595 | -1.74695807898536 | 0.032759 | down | 17:-:61332176-61332391 | 17:-:61332176-61332391 | LOC100362965 |
| ENSRNOG00000008930 | -3.38367725928406 | 0.027896 | down | 5:-:79553742-79570073 | 5:-:79553742-79570073 | Tnfsf15 |
| ENSRNOG00000054284 | -2.75729996111797 | 0.027524 | down | 19:-:37020852-37021958 | 19:-:37020852-37021958 | AABR07072639.2 |
| ENSRNOG00000015206 | 1.527367 | 0.033399 | up | 5:-:78368867-78379346 | 5:-:78368867-78379346 | Alad |
| ENSRNOG00000038835 | 2.072578 | 0.029817 | up | 11:+:67082193-67118795 | 11:+:67082193-67118795 | Cd86 |
| ENSRNOG00000046500 | -1.7057041101166 | 0.033495 | down | 1:+:81395841-81399672 | 1:+:81395841-81399672 | Irgq |
| ENSRNOG00000042353 | 2.627416 | 0.028208 | up | 19:-:43506976-43528851 | 19:-:43506976-43528851 | Mlkl |
| ENSRNOG00000028523 | 1.115291 | 0.034179 | up | 12:+:39790965-39824064 | 12:+:39790965-39824064 | Tctn1 |
| ENSRNOG00000002323 | -1.57879607645889 | 0.035333 | down | X:-:78870964-78911601 | X:-:78870964-78911601 | RGD1566265 |
| ENSRNOG00000030332 | 4.374483 | 0.028571 | up | 6:-:139140679-139142218 | 6:-:139140679-139142218 | Ighg1 |
| ENSRNOG00000029500 | 1.000183 | 0.034664 | up | 20:-:5468078-5476193 | 20:-:5468078-5476193 | Tapbp |
| ENSRNOG00000023809 | -3.25572038298613 | 0.034295 | down | 8:+:29453643-29962825 | 8:+:29453643-29962825 | Opcml |
| ENSRNOG00000016896 | 1.051177 | 0.031046 | up | 7:-:121297355-121311565 | 7:-:121297355-121311565 | Rpl3 |
| ENSRNOG00000055375 | 4.611717 | 0.028961 | up | 6:-:143194515-143195445 | 6:-:143194515-143195445 | AABR07065837.1 |
| ENSRNOG00000017672 | 1.892842 | 0.034667 | up | 17:-:69806065-69827112 | 17:-:69806065-69827112 | Akr1c14 |
| ENSRNOG00000006740 | 1.688298 | 0.034285 | up | 14:+:84447885-84452367 | 14:+:84447885-84452367 | Castor1 |
| ENSRNOG00000056810 | -2.87597470905277 | 0.029529 | down | X:-:23243470-23246719 | X:-:23243470-23246719 | AABR07037536.1 |
| ENSRNOG00000007539 | 1.675216 | 0.035829 | up | 6:-:45655954-45669148 | 6:-:45655954-45669148 | Rsad2 |
| ENSRNOG00000031785 | 4.401367 | 0.028831 | up | 7:-:143488684-143497108 | 7:-:143488684-143497108 | Krt76 |
| ENSRNOG00000010535 | -1.08584134407145 | 0.033203 | down | 2:+:74360622-74693341 | 2:+:74360622-74693341 | Cdh18 |
| ENSRNOG00000001336 | 1.540269 | 0.035114 | up | 12:-:38981927-38995570 | 12:-:38981927-38995570 | Orai1 |
| ENSRNOG00000046848 | 2.752374 | 0.031027 | up | 8:+:103459161-103484535 | 8:+:103459161-103484535 | PCOLCE2 |
| ENSRNOG00000012934 | -1.11633399431113 | 0.032183 | down | 16:-:83006718-83132785 | 16:-:83006718-83132785 | Arhgef7 |
| ENSRNOG00000007907 | -1.63671384520267 | 0.032613 | down | 6:+:3657325-3716078 | 6:+:3657325-3716078 | Tmem178a |
| ENSRNOG00000016625 | 3.48245 | 0.030225 | up | 1:-:48317995-48360261 | 1:-:48317995-48360261 | Slc22a2 |
| ENSRNOG00000061814 | -1.87300561695179 | 0.033608 | down | 11:+:87242522-87292955 | 11:+:87242522-87292955 | Dgcr2 |
| ENSRNOG00000060352 | -2.54948049592815 | 0.029698 | down | 12:-:30947613-30948195 | 12:-:30947613-30948195 | AABR07036024.2 |
| ENSRNOG00000002322 | 1.140794 | 0.035071 | up | 13:-:102780877-102790639 | 13:-:102780877-102790639 | RGD1310587 |
| ENSRNOG00000061304 | -1.72959907694065 | 0.032434 | down | X:-:157239306-157312028 | X:-:157239306-157312028 | Atp2b3 |
| ENSRNOG00000059886 | -2.67967643777282 | 0.02837 | down | 3:+:80965783-80968447 | 3:+:80965783-80968447 | AC135645.2 |
| ENSRNOG00000055246 | -1.21022829938034 | 0.032219 | down | 10:-:48629121-48772890 | 10:-:48629121-48772890 | Ncor1 |
| ENSRNOG00000033490 | -4.1475862541728 | 0.029933 | down | 8:+:40009691-40014734 | 8:+:40009691-40014734 | Vsig2 |
| ENSRNOG00000010529 | 1.277526 | 0.036314 | up | 1:-:56653938-56683731 | 1:-:56653938-56683731 | Thbs2 |
| ENSRNOG00000025269 | -2.0656591646781 | 0.036025 | down | 2:-:187774062-187786731 | 2:-:187774062-187786731 | Slc25a44 |
| ENSRNOG00000003049 | 2.968032 | 0.028877 | up | X:+:21696772-21699241 | X:+:21696772-21699241 | Hsd17b10 |
| ENSRNOG00000008169 | -3.66576666174602 | 0.032687 | down | 5:-:105336262-105582375 | 5:-:105336262-105582375 | Slc24a2 |
| ENSRNOG00000020936 | 2.573204 | 0.02948 | up | 8:-:118890399-118893702 | 8:-:118890399-118893702 | Nradd |
| ENSRNOG00000000299 | -1.98740882505456 | 0.034343 | down | 20:+:46428124-46519144 | 20:+:46428124-46519144 | Foxo3 |
| ENSRNOG00000022101 | 2.299498 | 0.034271 | up | 2:+:187322416-187326789 | 2:+:187322416-187326789 | Crabp2 |
| ENSRNOG00000053804 | -1.57568912457006 | 0.033386 | down | 4:-:160253073-160334910 | 4:-:160253073-160334910 | Prmt8 |
| ENSRNOG00000021839 | -1.66840914522027 | 0.036363 | down | 8:-:6255850-6305033 | 8:-:6255850-6305033 | Cep126 |
| ENSRNOG00000014509 | -1.27717771867576 | 0.032698 | down | 15:+:41448064-41530398 | 15:+:41448064-41530398 | Sacs |
| ENSRNOG00000017539 | -4.28029818153642 | 0.031709 | down | 3:+:161413298-161421520 | 3:+:161413298-161421520 | Mmp9 |
| ENSRNOG00000018086 | 1.150354 | 0.034923 | up | 1:+:224800252-224818482 | 1:+:224800252-224818482 | Slc22a8 |
| ENSRNOG00000015773 | 2.294708 | 0.028984 | up | 1:+:196996581-197024166 | 1:+:196996581-197024166 | Il21r |
| ENSRNOG00000060020 | 2.822857 | 0.031391 | up | 7:-:140767737-140770647 | 7:-:140767737-140770647 | C1ql4 |
| ENSRNOG00000019206 | 3.102709 | 0.030343 | up | 1:-:194767474-194769524 | 1:-:194767474-194769524 | Nupr1 |
| ENSRNOG00000004062 | 3.387695 | 0.029714 | up | 13:-:47342079-47377703 | 13:-:47342079-47377703 | C4bpa |
| ENSRNOG00000054418 | -4.50439387800261 | 0.033655 | down | 1:+:198800801-198801816 | 1:+:198800801-198801816 | AABR07005779.3 |
| ENSRNOG00000007663 | 1.908327 | 0.036646 | up | 5:-:105209846-105212173 | 5:-:105209846-105212173 | LOC100911372 |
| ENSRNOG00000010957 | -2.14817197066297 | 0.036864 | down | 4:-:67396192-67520356 | 4:-:67396192-67520356 | Braf |
| ENSRNOG00000055959 | 3.561649 | 0.030265 | up | KL568149.1:+:194682-194805 | KL568149.1:+:194682-194805 | AC242253.1 |
| ENSRNOG00000028512 | 1.002721 | 0.035381 | up | 7:-:14044586-14054639 | 7:-:14044586-14054639 | Ilvbl |
| ENSRNOG00000042118 | -1.97268116338045 | 0.036618 | down | 2:+:57276919-57348481 | 2:+:57276919-57348481 | Cplane1 |
| ENSRNOG00000049517 | -3.20935360693661 | 0.034477 | down | 1:-:14402913-14412807 | 1:-:14402913-14412807 | Tnfaip3 |
| ENSRNOG00000015257 | -2.68937523726637 | 0.034359 | down | 8:-:67376294-67492037 | 8:-:67376294-67492037 | Coro2b |
| ENSRNOG00000026392 | -1.36288489759871 | 0.036276 | down | 2:+:72006099-72540263 | 2:+:72006099-72540263 | Cdh12 |
| ENSRNOG00000019176 | 1.442914 | 0.037042 | up | X:-:113583844-113584459 | X:-:113583844-113584459 | Kcne5 |
| ENSRNOG00000015518 | 1.458622 | 0.036017 | up | 1:-:256806472-256813711 | 1:-:256806472-256813711 | Rbp4 |
| ENSRNOG00000050995 | -2.53793718444154 | 0.029612 | down | 1:+:84652632-84653384 | 1:+:84652632-84653384 | AABR07002774.4 |
| ENSRNOG00000053272 | 1.534169 | 0.034825 | up | 13:+:51022681-51030802 | 13:+:51022681-51030802 | Chi3l1 |
| ENSRNOG00000024870 | -2.26491356860182 | 0.034209 | down | 7:+:30699476-31681182 | 7:+:30699476-31681182 | Anks1b |
| ENSRNOG00000033235 | 1.539991 | 0.037044 | up | 1:-:80517411-80544825 | 1:-:80517411-80544825 | Relb |
| ENSRNOG00000058335 | -2.66028079818913 | 0.030496 | down | 3:-:25335682-25351082 | 3:-:25335682-25351082 | AABR07051879.1 |
| ENSRNOG00000058105 | -1.69270598222078 | 0.033257 | down | 1:-:168971274-168972725 | 1:-:168971274-168972725 | Hbb |
| ENSRNOG00000006263 | -1.5330638360924 | 0.038074 | down | X:+:128897181-128925407 | X:+:128897181-128925407 | Sh2d1a |
| ENSRNOG00000011145 | 4.083047 | 0.033311 | up | 4:-:85286372-85329362 | 4:-:85286372-85329362 | Crhr2 |
| ENSRNOG00000050000 | 4.222993 | 0.033232 | up | 11:+:86092468-86092779 | 11:+:86092468-86092779 | AABR07034739.1 |
| ENSRNOG00000043233 | 1.533691 | 0.038227 | up | 7:-:15198940-15225410 | 7:-:15198940-15225410 | Cyp4f6 |
| ENSRNOG00000003614 | -2.06780403368616 | 0.036928 | down | 13:+:43850751-44157924 | 13:+:43850751-44157924 | Mgat5 |
| ENSRNOG00000036802 | -1.34702096974302 | 0.035203 | down | 3:+:154863072-154869460 | 3:+:154863072-154869460 | Snhg11 |
| ENSRNOG00000003504 | 1.092994 | 0.034859 | up | 19:-:26074987-26084679 | 19:-:26074987-26084679 | Rnaseh2a |
| ENSRNOG00000023496 | -1.96392099941294 | 0.038378 | down | 1:-:125553439-125967756 | 1:-:125553439-125967756 | Fam189a1 |
| ENSRNOG00000003354 | -2.15204318187252 | 0.030217 | down | X:+:92131209-92132357 | X:+:92131209-92132357 | Pabpc5 |
| ENSRNOG00000056130 | -2.83686668456186 | 0.031642 | down | 10:+:1214731-1221289 | 10:+:1214731-1221289 | AABR07028970.2 |
| ENSRNOG00000003515 | 1.063734 | 0.037814 | up | 13:-:99271366-99300579 | 13:-:99271366-99300579 | Ephx1 |
| ENSRNOG00000004699 | 1.858829 | 0.038684 | up | 3:-:101545208-101547478 | 3:-:101545208-101547478 | Fibin |
| ENSRNOG00000039336 | 2.409326 | 0.033322 | up | 5:+:59228199-59228519 | 5:+:59228199-59228519 | Hrct1 |
| ENSRNOG00000001177 | 1.524332 | 0.037444 | up | 12:+:47254484-47263747 | 12:+:47254484-47263747 | Acads |
| ENSRNOG00000005214 | 1.560403 | 0.037723 | up | 14:-:100151210-100217913 | 14:-:100151210-100217913 | Plek |
| ENSRNOG00000016267 | -1.49824199494957 | 0.038014 | down | 1:-:204245645-204322544 | 1:-:204245645-204322544 | Chst15 |
| ENSRNOG00000003666 | 5.447911 | 0.036564 | up | 14:+:21177237-21183542 | 14:+:21177237-21183542 | Jchain |
| ENSRNOG00000018163 | -4.15506736735978 | 0.038623 | down | 1:-:43567842-43638161 | 1:-:43567842-43638161 | Ipcef1 |
| ENSRNOG00000047258 | -2.30379878414987 | 0.03093 | down | 15:-:47433566-47442664 | 15:-:47433566-47442664 | Prss55 |
| ENSRNOG00000001113 | 1.182568 | 0.034194 | up | 12:+:14021727-14078134 | 12:+:14021727-14078134 | Mmd2 |
| ENSRNOG00000004048 | -1.43262079943628 | 0.036223 | down | 7:+:132857628-133018584 | 7:+:132857628-133018584 | Lrrk2 |
| ENSRNOG00000019118 | 1.415988 | 0.035815 | up | 3:-:162084315-162147393 | 3:-:162084315-162147393 | Slc13a3 |
| ENSRNOG00000011189 | 1.125714 | 0.036513 | up | 8:-:115134765-115140080 | 8:-:115134765-115140080 | Acy1 |
| ENSRNOG00000058439 | 4.737458 | 0.035631 | up | X:-:156392630-156399760 | X:-:156392630-156399760 | LOC100910130 |
| ENSRNOG00000037380 | 2.239956 | 0.031605 | up | 1:+:91746486-91747174 | 1:+:91746486-91747174 | AC136661.1 |
| ENSRNOG00000059121 | 3.447773 | 0.033717 | up | 6:-:139783839-139911839 | 6:-:139783839-139911839 | AABR07065714.1 |
| ENSRNOG00000027731 | -2.54874070275005 | 0.031542 | down | 5:-:157159936-157165767 | 5:-:157159936-157165767 | Ubxn10 |
| ENSRNOG00000004327 | 1.077334 | 0.039261 | up | 14:-:91905919-91996774 | 14:-:91905919-91996774 | Ddc |
| ENSRNOG00000047076 | 3.080128 | 0.032028 | up | 12:+:41155497-41167505 | 12:+:41155497-41167505 | Oas1g |
| ENSRNOG00000056849 | -2.83206392934085 | 0.03383 | down | X:-:22369521-22399901 | X:-:22369521-22399901 | Kantr |
| ENSRNOG00000003543 | 2.665502 | 0.03207 | up | X:+:23668363-23693162 | X:+:23668363-23693162 | Gpr143 |
| ENSRNOG00000003121 | -1.85281390426532 | 0.036145 | down | 10:+:62035866-62109078 | 10:+:62035866-62109078 | Rtn4rl1 |
| ENSRNOG00000060634 | -2.33681570923008 | 0.031457 | down | 1:-:84953311-84959283 | 1:-:84953311-84959283 | AABR07002784.2 |
| ENSRNOG00000016361 | 1.359249 | 0.038791 | up | 9:+:81816872-81844364 | 9:+:81816872-81844364 | Plcd4 |
| ENSRNOG00000010575 | 1.734312 | 0.037122 | up | 2:-:243175346-243224883 | 2:-:243175346-243224883 | Dapp1 |
| ENSRNOG00000017969 | -1.7763599551825 | 0.035606 | down | 2:+:217963456-218005509 | 2:+:217963456-218005509 | Olfm3 |
| ENSRNOG00000049484 | -1.14086289779368 | 0.034402 | down | 3:-:165412803-165477771 | 3:-:165412803-165477771 | Atp9a |
| ENSRNOG00000005447 | -1.66121190857858 | 0.037701 | down | 10:-:74243812-74298599 | 10:-:74243812-74298599 | Ypel2 |
| ENSRNOG00000016581 | 1.582922 | 0.039204 | up | 17:+:32904119-32911495 | 17:+:32904119-32911495 | Serpinb1a |
| ENSRNOG00000053812 | 3.350148 | 0.035281 | up | 2:-:196105569-196113149 | 2:-:196105569-196113149 | LOC103689947 |
| ENSRNOG00000032708 | 3.57855 | 0.037036 | up | 20:+:4039413-4049711 | 20:+:4039413-4049711 | RT1-Bb |
| ENSRNOG00000026055 | -2.49406021914824 | 0.038359 | down | 4:-:85911861-85915099 | 4:-:85911861-85915099 | Neurod6 |
| ENSRNOG00000059120 | -2.06375916199824 | 0.034348 | down | 2:-:156792500-156807305 | 2:-:156792500-156807305 | AABR07011031.1 |
| ENSRNOG00000015603 | -2.86973704249066 | 0.036996 | down | 6:+:9790422-9973396 | 6:+:9790422-9973396 | Prkce |
| ENSRNOG00000020821 | -1.96694091325595 | 0.038401 | down | 1:+:88750462-88765220 | 1:+:88750462-88765220 | LOC108348122 |
| ENSRNOG00000018792 | 2.83962 | 0.034398 | up | 10:+:14828597-14833827 | 10:+:14828597-14833827 | Tekt4 |
| ENSRNOG00000045680 | -1.68008055644616 | 0.035944 | down | 1:-:214408100-214408549 | 1:-:214408100-214408549 | LOC100911402 |
| ENSRNOG00000047014 | -3.10927972770845 | 0.039572 | down | 2:+:22909569-23009678 | 2:+:22909569-23009678 | Homer1 |
| ENSRNOG00000012634 | -1.71260457520023 | 0.038338 | down | 5:-:60591023-60658564 | 5:-:60591023-60658564 | Fbxo10 |
| ENSRNOG00000060635 | -1.8869329090747 | 0.038736 | down | 3:-:83052724-83134363 | 3:-:83052724-83134363 | AABR07052897.3 |
| ENSRNOG00000010911 | -1.50146909953562 | 0.040075 | down | 8:-:111970288-111984572 | 8:-:111970288-111984572 | Tmem108 |
| ENSRNOG00000008409 | 1.565404 | 0.040154 | up | 7:+:18440742-18491448 | 7:+:18440742-18491448 | Myo1f |
| ENSRNOG00000059863 | -2.06387573624318 | 0.036022 | down | 13:-:110673851-110678389 | 13:-:110673851-110678389 | AABR07022168.1 |
| ENSRNOG00000000441 | 2.048449 | 0.039474 | up | 20:+:4355175-4357107 | 20:+:4355175-4357107 | Gpsm3 |
| ENSRNOG00000056248 | -3.34472293548161 | 0.039539 | down | 10:+:908806-953474 | 10:+:908806-953474 | Marf1 |
| ENSRNOG00000037165 | -2.39156319348016 | 0.039091 | down | 5:-:153807955-153840178 | 5:-:153807955-153840178 | Nipal3 |
| ENSRNOG00000016117 | 1.212574 | 0.040457 | up | 1:-:256585410-256734730 | 1:-:256585410-256734730 | Myof |
| ENSRNOG00000051772 | -2.97593637360484 | 0.035567 | down | 17:-:22252020-22263289 | 17:-:22252020-22263289 | AABR07027272.1 |
| ENSRNOG00000059475 | -2.10321014226382 | 0.034794 | down | 14:+:43166370-43171890 | 14:+:43166370-43171890 | AABR07014987.1 |
| ENSRNOG00000000781 | -2.04265058613467 | 0.040913 | down | 20:-:2098375-2103864 | 20:-:2098375-2103864 | Rnf39 |
| ENSRNOG00000012557 | -3.17768888399201 | 0.034529 | down | 10:-:66225995-66229311 | 10:-:66225995-66229311 | Lgals5 |
| ENSRNOG00000038119 | 3.764451 | 0.034419 | up | 11:+:84827062-84833286 | 11:+:84827062-84833286 | AABR07034706.1 |
| ENSRNOG00000014519 | -2.42882475409365 | 0.032889 | down | 10:-:56366609-56368360 | 10:-:56366609-56368360 | Slc35g3 |
| ENSRNOG00000011155 | 2.423837 | 0.040319 | up | 10:+:105564535-105565162 | 10:+:105564535-105565162 | AABR07072207.1 |
| ENSRNOG00000006226 | -1.33162367912704 | 0.036978 | down | 7:-:119228102-119352605 | 7:-:119228102-119352605 | Cacng2 |
| ENSRNOG00000053968 | -1.72938978563642 | 0.03998 | down | 15:-:87453035-87540474 | 15:-:87453035-87540474 | AABR07019086.1 |
| ENSRNOG00000061100 | 1.133447 | 0.041177 | up | 4:+:100218661-100222130 | 4:+:100218661-100222130 | Tmem150a |
| ENSRNOG00000050869 | 1.657182 | 0.040224 | up | 11:+:89008008-89009146 | 11:+:89008008-89009146 | Cebpd |
| ENSRNOG00000042867 | 2.561036 | 0.033492 | up | 16:+:81463129-81487566 | 16:+:81463129-81487566 | LOC688801 |
| ENSRNOG00000062053 | -2.15577142612045 | 0.033789 | down | 10:-:92342011-92344829 | 10:-:92342011-92344829 | AABR07030527.2 |
| ENSRNOG00000046791 | -4.29130142044057 | 0.0384 | down | 20:+:28572242-28717243 | 20:+:28572242-28717243 | Sh3rf3 |
| ENSRNOG00000006992 | 1.078666 | 0.035279 | up | 5:+:2632712-2640021 | 5:+:2632712-2640021 | Rpl7 |
| ENSRNOG00000060775 | -1.73885530540905 | 0.038978 | down | 15:+:86243148-86457739 | 15:+:86243148-86457739 | Lmo7 |
| ENSRNOG00000002610 | 1.109072 | 0.037805 | up | 10:+:7041510-7055101 | 10:+:7041510-7055101 | Carhsp1 |
| ENSRNOG00000001120 | -1.50721116067448 | 0.036932 | down | 12:-:43421308-43576804 | 12:-:43421308-43576804 | Med13l |
| ENSRNOG00000001337 | -1.83297828467572 | 0.039341 | down | 12:-:38845621-38869346 | 12:-:38845621-38869346 | Setd1b |
| ENSRNOG00000031778 | -2.01254761536763 | 0.037325 | down | 2:+:187512164-187538026 | 2:+:187512164-187538026 | Mef2d |
| ENSRNOG00000056518 | -2.4405676990203 | 0.033525 | down | 3:+:154866941-154867073 | 3:+:154866941-154867073 | SNORA71 |
| ENSRNOG00000023152 | -1.07320533360234 | 0.038612 | down | 5:-:166672450-166695134 | 5:-:166672450-166695134 | Tmem201 |
| ENSRNOG00000011697 | -2.47495429581876 | 0.041426 | down | 19:-:32401661-32528965 | 19:-:32401661-32528965 | Zfp827 |
| ENSRNOG00000011146 | 1.135519 | 0.038729 | up | 2:-:104916738-104958034 | 2:-:104916738-104958034 | Gyg1 |
| ENSRNOG00000015418 | 4.24125 | 0.038649 | up | 18:-:12528111-12640716 | 18:-:12528111-12640716 | Klhl14 |
| ENSRNOG00000001255 | -1.66442491201853 | 0.041134 | down | 12:-:38567247-38638536 | 12:-:38567247-38638536 | Mlxip |
| ENSRNOG00000048578 | -2.03133858168006 | 0.037365 | down | 15:+:32386816-32387346 | 15:+:32386816-32387346 | AABR07017901.1 |
| ENSRNOG00000014647 | 1.025103 | 0.039538 | up | 19:+:37127508-37171069 | 19:+:37127508-37171069 | Cbfb |
| ENSRNOG00000028895 | 2.303914 | 0.040471 | up | 11:-:80638945-80650802 | 11:-:80638945-80650802 | Rtp4 |
| ENSRNOG00000052298 | -3.05692384610225 | 0.038611 | down | 14:+:42007312-42009998 | 14:+:42007312-42009998 | AABR07014974.1 |
| ENSRNOG00000024595 | -3.53868510283013 | 0.041142 | down | 3:+:55094637-55338445 | 3:+:55094637-55338445 | Cers6 |
| ENSRNOG00000014007 | -2.71549056067909 | 0.040337 | down | 17:-:23823632-23923792 | 17:-:23823632-23923792 | Gfod1 |
| ENSRNOG00000011044 | -1.87803345021171 | 0.040142 | down | 6:-:128473570-128567198 | 6:-:128473570-128567198 | Clmn |
| ENSRNOG00000010473 | 1.048792 | 0.04179 | up | 4:+:147686490-147714585 | 4:+:147686490-147714585 | Cand2 |
| ENSRNOG00000006548 | 1.343707 | 0.040903 | up | 10:+:93520132-93581296 | 10:+:93520132-93581296 | Mrc2 |
| ENSRNOG00000022395 | -1.72014969581732 | 0.038943 | down | 10:+:106712127-106762043 | 10:+:106712127-106762043 | Tnrc6c |
| ENSRNOG00000005284 | -4.16235477733315 | 0.041161 | down | 3:+:111545007-111553604 | 3:+:111545007-111553604 | Itpka |
| ENSRNOG00000016156 | -2.1544408895128 | 0.035734 | down | 7:-:121011680-121029754 | 7:-:121011680-121029754 | Nptxr |
| ENSRNOG00000026649 | -1.80339691619292 | 0.040143 | down | 6:+:28235695-28340577 | 6:+:28235695-28340577 | Dnmt3a |
| ENSRNOG00000019265 | -1.72760105612912 | 0.038753 | down | 18:-:31414250-31430973 | 18:-:31414250-31430973 | Pcdh12 |
| ENSRNOG00000026226 | -1.05439563275144 | 0.039137 | down | 5:+:114940053-115000691 | 5:+:114940053-115000691 | Hook1 |
| ENSRNOG00000006623 | 1.780135 | 0.040109 | up | 3:-:46327383-46361092 | 3:-:46327383-46361092 | Cd302 |
| ENSRNOG00000047653 | 1.90444 | 0.037458 | up | 12:-:50390940-50404550 | 12:-:50390940-50404550 | Crybb1 |
| ENSRNOG00000023410 | 2.031998 | 0.03544 | up | 7:-:118927008-118933812 | 7:-:118927008-118933812 | Apol9a |
| ENSRNOG00000007338 | 1.865788 | 0.042053 | up | 4:+:122835436-122895121 | 4:+:122835436-122895121 | Fbln2 |
| ENSRNOG00000012022 | -2.351181456613 | 0.037138 | down | 8:-:55694804-55696601 | 8:-:55694804-55696601 | RGD1562914 |
| ENSRNOG00000013742 | -1.21496903217799 | 0.0371 | down | 19:-:12481540-12942963 | 19:-:12481540-12942963 | Large1 |
| ENSRNOG00000030575 | 3.451786 | 0.037857 | up | 2:+:223029559-223045185 | 2:+:223029559-223045185 | AABR07013111.1 |
| ENSRNOG00000059961 | 1.132264 | 0.038844 | up | 7:-:139232251-139254551 | 7:-:139232251-139254551 | Rapgef3 |
| ENSRNOG00000042711 | -2.22026195834458 | 0.035814 | down | 11:-:86917667-86920094 | 11:-:86917667-86920094 | AABR07072264.1 |
| ENSRNOG00000019207 | -1.91239217468781 | 0.03667 | down | 1:+:100297152-100344377 | 1:+:100297152-100344377 | Shank1 |
| ENSRNOG00000011821 | 2.774384 | 0.037542 | up | 2:+:189997129-189999604 | 2:+:189997129-189999604 | S100a4 |
| ENSRNOG00000000701 | 1.279201 | 0.037636 | up | 12:-:48621454-48627297 | 12:-:48621454-48627297 | Iscu |
| ENSRNOG00000008157 | -1.0909997043141 | 0.036868 | down | 4:+:147037179-147195096 | 4:+:147037179-147195096 | Syn2 |
| ENSRNOG00000013794 | 2.437318 | 0.043446 | up | 8:+:106449321-106470842 | 8:+:106449321-106470842 | Rbp1 |
| ENSRNOG00000049802 | -1.48964355267233 | 0.03982 | down | 18:-:18003537-18079560 | 18:-:18003537-18079560 | AABR07031533.1 |
| ENSRNOG00000000528 | 1.601753 | 0.043092 | up | 20:+:6973398-6990023 | 20:+:6973398-6990023 | Fgd2 |
| ENSRNOG00000000635 | -3.53674159092477 | 0.042932 | down | 20:+:21564975-21880715 | 20:+:21564975-21880715 | Arid5b |
| ENSRNOG00000003267 | 1.091207 | 0.039884 | up | 13:+:99184564-99188431 | 13:+:99184564-99188431 | Pycr2 |
| ENSRNOG00000029071 | -1.37224265763212 | 0.040056 | down | 2:+:247248407-247397483 | 2:+:247248407-247397483 | Unc5c |
| ENSRNOG00000031312 | 1.026231 | 0.041031 | up | 4:+:157864969-157877633 | 4:+:157864969-157877633 | Tnfrsf1a |
| ENSRNOG00000003214 | 1.050796 | 0.043356 | up | 10:+:48987822-48989595 | 10:+:48987822-48989595 | AABR07029742.1 |
| ENSRNOG00000060194 | -2.48204172691719 | 0.036479 | down | 15:-:96790502-96817727 | 15:-:96790502-96817727 | AABR07019254.2 |
| ENSRNOG00000010319 | 1.486327 | 0.041584 | up | 15:+:57221292-57277811 | 15:+:57221292-57277811 | Lcp1 |
| ENSRNOG00000031671 | -1.88511517722095 | 0.039733 | down | 4:+:150133590-150142713 | 4:+:150133590-150142713 | Rasgef1a |
| ENSRNOG00000001313 | -1.35970540089481 | 0.039755 | down | 20:-:14593819-14620019 | 20:-:14593819-14620019 | Gnaz |
| ENSRNOG00000006278 | -1.7755398602195 | 0.043274 | down | 7:-:72326896-72328128 | 7:-:72326896-72328128 | Tspyl5 |
| ENSRNOG00000000918 | 1.021521 | 0.037852 | up | 12:-:30308469-30314519 | 12:-:30308469-30314519 | Zbed5 |
| ENSRNOG00000011893 | 1.345837 | 0.03719 | up | 2:-:185440291-185444897 | 2:-:185440291-185444897 | Rps3a |
| ENSRNOG00000007304 | -1.02743390777657 | 0.038062 | down | 4:+:89078711-89169146 | 4:+:89078711-89169146 | Herc3 |
| ENSRNOG00000001982 | -1.15645365953513 | 0.041519 | down | 11:-:51037393-51202820 | 11:-:51037393-51202820 | Cblb |
| ENSRNOG00000046744 | 1.125793 | 0.040634 | up | 18:-:3673915-3676188 | 18:-:3673915-3676188 | Ankrd29 |
| ENSRNOG00000011171 | -2.33860728401007 | 0.041978 | down | 5:+:139783951-139819016 | 5:+:139783951-139819016 | Rims3 |
| ENSRNOG00000001242 | 1.583455 | 0.039354 | up | 20:+:13817795-13825475 | 20:+:13817795-13825475 | Gstt3 |
| ENSRNOG00000002338 | -1.24374481497762 | 0.040648 | down | 10:-:76386471-76407989 | 10:-:76386471-76407989 | Dgke |
| ENSRNOG00000022163 | 4.571679 | 0.040868 | up | 18:+:12637441-12641158 | 18:+:12637441-12641158 | LOC102555023 |
| ENSRNOG00000023931 | -2.42649985923952 | 0.037184 | down | 1:+:88113445-88116548 | 1:+:88113445-88116548 | Ggn |
| ENSRNOG00000013321 | 1.153418 | 0.042676 | up | X:+:122507374-122690465 | X:+:122507374-122690465 | Dock11 |
| ENSRNOG00000002041 | -1.4055537693996 | 0.041307 | down | 11:+:61083757-61158838 | 11:+:61083757-61158838 | Boc |
| ENSRNOG00000051688 | 2.463044 | 0.037466 | up | 16:+:10554136-10564927 | 16:+:10554136-10564927 | Syt15 |
| ENSRNOG00000048053 | 2.547953 | 0.037138 | up | 10:+:70398995-70400317 | 10:+:70398995-70400317 | AC128859.3 |
| ENSRNOG00000002202 | -2.16769287643797 | 0.044774 | down | 14:+:16491573-16493876 | 14:+:16491573-16493876 | Sowahb |
| ENSRNOG00000002115 | 1.190134 | 0.038663 | up | 11:+:30363280-30368862 | 11:+:30363280-30368862 | Sod1 |
| ENSRNOG00000041411 | -1.98913548956487 | 0.039655 | down | 3:-:113417087-113417187 | 3:-:113417087-113417187 | - |
| ENSRNOG00000013565 | -1.16726240027329 | 0.044149 | down | 1:-:92087215-92120009 | 1:-:92087215-92120009 | Zfp507 |
| ENSRNOG00000010484 | -2.35321345904367 | 0.044168 | down | 5:-:100924622-100977902 | 5:-:100924622-100977902 | Zdhhc21 |
| ENSRNOG00000017991 | -1.05314080806413 | 0.044334 | down | 17:+:1936163-1942865 | 17:+:1936163-1942865 | Cdk20 |
| ENSRNOG00000001618 | 2.506171 | 0.037951 | up | 11:-:38251955-38274217 | 11:-:38251955-38274217 | Ripk4 |
| ENSRNOG00000047635 | -1.18584577145883 | 0.039187 | down | 4:+:68011932-68059249 | 4:+:68011932-68059249 | Tmem178b |
| ENSRNOG00000050404 | 1.737346 | 0.039738 | up | 3:-:171294856-171342646 | 3:-:171294856-171342646 | Pmepa1 |
| ENSRNOG00000027096 | 3.235911 | 0.039131 | up | 1:-:220844558-220848153 | 1:-:220844558-220848153 | Ctsw |
| ENSRNOG00000013085 | -2.3881216015524 | 0.037407 | down | 5:-:34174411-34813116 | 5:-:34174411-34813116 | Nkain3 |
| ENSRNOG00000020778 | -1.73803433564213 | 0.04126 | down | 2:-:189088570-189096785 | 2:-:189088570-189096785 | Chrnb2 |
| ENSRNOG00000053168 | -3.30426782912715 | 0.040522 | down | 2:+:230476320-230479594 | 2:+:230476320-230479594 | LOC102553088 |
| ENSRNOG00000020369 | 1.14525 | 0.039073 | up | 1:-:215828102-215846911 | 1:-:215828102-215846911 | Igf2 |
| ENSRNOG00000054626 | 1.16538 | 0.038359 | up | 5:-:136020941-136023511 | 5:-:136020941-136023511 | Rps8 |
| ENSRNOG00000048088 | -1.26074642155419 | 0.042679 | down | 4:+:58053041-58063138 | 4:+:58053041-58063138 | Mest |
| ENSRNOG00000062204 | 2.096162 | 0.04113 | up | 2:-:185286358-185287960 | 2:-:185286358-185287960 | AABR07012097.1 |
| ENSRNOG00000057832 | 2.526078 | 0.037977 | up | 18:-:15192962-15225427 | 18:-:15192962-15225427 | Rnf125 |
| ENSRNOG00000011885 | 1.150302 | 0.043734 | up | 1:+:91596397-91657395 | 1:+:91596397-91657395 | Rhpn2 |
| ENSRNOG00000007613 | 1.23059 | 0.041784 | up | 8:+:48443767-48445637 | 8:+:48443767-48445637 | C1qtnf5 |
| ENSRNOG00000024832 | -4.59464234962048 | 0.044424 | down | 17:+:88215834-88651476 | 17:+:88215834-88651476 | Gpr158 |
| ENSRNOG00000012821 | 2.838698 | 0.03929 | up | 15:-:61923299-61947764 | 15:-:61923299-61947764 | Cnmd |
| ENSRNOG00000000239 | 3.06432 | 0.039964 | up | 10:+:69423086-69424979 | 10:+:69423086-69424979 | Ccl7 |
| ENSRNOG00000024635 | -1.83279904558997 | 0.045307 | down | 10:-:14326802-14373334 | 10:-:14326802-14373334 | Cramp1 |
| ENSRNOG00000007918 | 2.178798 | 0.041362 | up | 4:+:66670618-66846805 | 4:+:66670618-66846805 | Tbxas1 |
| ENSRNOG00000013436 | -1.66618723196378 | 0.045585 | down | 1:-:15893533-16203909 | 1:-:15893533-16203909 | Pde7b |
| ENSRNOG00000008519 | -1.5749999113481 | 0.039914 | down | 8:-:102088505-102159828 | 8:-:102088505-102159828 | Dipk2a |
| ENSRNOG00000029501 | 1.432341 | 0.044697 | up | 1:+:261291870-261318984 | 1:+:261291870-261318984 | Hoga1 |
| ENSRNOG00000056978 | -2.90080158653082 | 0.038481 | down | 12:-:40881397-40881530 | 12:-:40881397-40881530 | - |
| ENSRNOG00000010457 | -1.62950953145724 | 0.042132 | down | 6:+:110624856-110637382 | 6:+:110624856-110637382 | Vash1 |
| ENSRNOG00000021010 | 1.243266 | 0.039879 | up | 1:-:221504170-221516110 | 1:-:221504170-221516110 | Arl2 |
| ENSRNOG00000003866 | 1.89378 | 0.045663 | up | 13:-:45314933-45318878 | 13:-:45314933-45318878 | Cxcr4 |
| ENSRNOG00000011068 | 1.17946 | 0.045157 | up | 1:+:251145253-251230715 | 1:+:251145253-251230715 | Papss2 |
| ENSRNOG00000020261 | -1.5994037302313 | 0.042328 | down | 18:+:27558089-27570508 | 18:+:27558089-27570508 | Fam53c |
| ENSRNOG00000007922 | 2.6314 | 0.039297 | up | 5:+:138300107-138305683 | 5:+:138300107-138305683 | Cldn19 |
| ENSRNOG00000042929 | -1.73653803721451 | 0.042982 | down | 13:-:88636875-88943592 | 13:-:88636875-88943592 | LOC100361087 |
| ENSRNOG00000042404 | 2.121344 | 0.039244 | up | 15:+:59216705-59279967 | 15:+:59216705-59279967 | Ccdc122 |
| ENSRNOG00000020081 | 1.233606 | 0.045181 | up | 1:+:266482858-266514569 | 1:+:266482858-266514569 | As3mt |
| ENSRNOG00000014218 | -1.09675392456251 | 0.040896 | down | 8:-:49100759-49106177 | 8:-:49100759-49106177 | Tmem25 |
| ENSRNOG00000053391 | 2.969596 | 0.040681 | up | 18:+:78282423-78283480 | 18:+:78282423-78283480 | AABR07032724.1 |
| ENSRNOG00000010695 | 2.133734 | 0.04587 | up | 2:+:179952227-180128283 | 2:+:179952227-180128283 | Pdgfc |
| ENSRNOG00000004947 | -3.23318686373366 | 0.04503 | down | 7:-:15024693-15027933 | 7:-:15024693-15027933 | Zfp871 |
| ENSRNOG00000024591 | 3.452339 | 0.039642 | up | 10:+:50928309-51014562 | 10:+:50928309-51014562 | Hs3st3a1 |
| ENSRNOG00000028641 | -1.04673465911309 | 0.041196 | down | 20:+:6049286-6086099 | 20:+:6049286-6086099 | Brpf3 |
| ENSRNOG00000060608 | 2.55 | 0.038736 | up | 15:-:47938116-47938988 | 15:-:47938116-47938988 | AABR07018226.2 |
| ENSRNOG00000032490 | -1.64747455479762 | 0.042919 | down | 13:+:31081804-31231213 | 13:+:31081804-31231213 | Cdh7 |
| ENSRNOG00000060599 | -1.91794923518924 | 0.040771 | down | 1:+:112976770-113265364 | 1:+:112976770-113265364 | Gabrb3 |
| ENSRNOG00000006963 | 2.160889 | 0.039295 | up | 13:+:48426820-48449490 | 13:+:48426820-48449490 | Ctse |
| ENSRNOG00000061366 | -3.01360426173324 | 0.044002 | down | X:+:23217954-23219754 | X:+:23217954-23219754 | AABR07037528.1 |
| ENSRNOG00000022392 | 1.581657 | 0.044166 | up | 12:+:45905371-45920013 | 12:+:45905371-45920013 | Hspb8 |
| ENSRNOG00000046803 | 3.311962 | 0.041192 | up | 5:-:171624796-171648563 | 5:-:171624796-171648563 | Arhgef16 |
| ENSRNOG00000037113 | 1.793687 | 0.046495 | up | 10:+:70370300-70376331 | 10:+:70370300-70376331 | Slfn2 |
| ENSRNOG00000012503 | -1.33151528263578 | 0.042672 | down | 5:-:160325107-160352927 | 5:-:160325107-160352927 | Dnajc16 |
| ENSRNOG00000006831 | -3.87518771969993 | 0.045474 | down | 8:+:7128656-7187796 | 8:+:7128656-7187796 | Pgr |
| ENSRNOG00000009409 | 1.361626 | 0.040113 | up | 5:+:164972480-164977916 | 5:+:164972480-164977916 | Fbxo2 |
| ENSRNOG00000034150 | 2.803338 | 0.04094 | up | X:-:139475655-139476206 | X:-:139475655-139476206 | AABR07041778.1 |
| ENSRNOG00000036918 | 1.800658 | 0.044167 | up | 4:+:183656013-183665754 | 4:+:183656013-183665754 | Etfbkmt |
| ENSRNOG00000009427 | 6.322902 | 0.045033 | up | 10:-:85032799-85049331 | 10:-:85032799-85049331 | Tbx21 |
| ENSRNOG00000052887 | -1.67309162689971 | 0.039376 | down | 5:+:120340646-120492487 | 5:+:120340646-120492487 | Dnajc6 |
| ENSRNOG00000046168 | -1.0983130272921 | 0.040052 | down | 2:+:166140112-166218774 | 2:+:166140112-166218774 | Ppm1l |
| ENSRNOG00000019675 | 2.259899 | 0.041087 | up | 1:-:199437832-199439210 | 1:-:199437832-199439210 | Pycard |
| ENSRNOG00000030055 | 1.073741 | 0.041207 | up | 5:-:168126157-168136666 | 5:-:168126157-168136666 | Vamp3 |
| ENSRNOG00000011387 | -3.67076747581292 | 0.046861 | down | 4:-:115066309-115157263 | 4:-:115066309-115157263 | Tet3 |
| ENSRNOG00000027271 | -2.71119253632448 | 0.043415 | down | 10:+:95642640-95657977 | 10:+:95642640-95657977 | RGD1359290 |
| ENSRNOG00000031930 | 1.422479 | 0.046871 | up | 7:-:142273833-142300382 | 7:-:142273833-142300382 | Bin2 |
| ENSRNOG00000062220 | 2.827468 | 0.040834 | up | 17:+:15429708-15603199 | 17:+:15429708-15603199 | Cenpp |
| ENSRNOG00000027516 | -2.66583249088581 | 0.042009 | down | 20:-:5106890-5107899 | 20:-:5106890-5107899 | Ly6g5b |
| ENSRNOG00000059922 | 4.110548 | 0.041595 | up | 15:+:105941836-105943359 | 15:+:105941836-105943359 | AABR07019442.1 |
| ENSRNOG00000061272 | 1.036812 | 0.045275 | up | 2:+:180878153-180883459 | 2:+:180878153-180883459 | AABR07011996.1 |
| ENSRNOG00000021524 | 4.597647 | 0.04324 | up | 11:+:30904733-30915225 | 11:+:30904733-30915225 | Mrap |
| ENSRNOG00000013940 | -2.84925675336285 | 0.043417 | down | 2:+:127844575-127947952 | 2:+:127844575-127947952 | RGD1565989 |
| ENSRNOG00000020165 | -2.10016564753807 | 0.040042 | down | 1:+:199720038-199721010 | 1:+:199720038-199721010 | Ahsp |
| ENSRNOG00000015152 | -3.54135677933769 | 0.041334 | down | 1:+:213886775-213897783 | 1:+:213886775-213897783 | Pkp3 |
| ENSRNOG00000010897 | 1.219716 | 0.042475 | up | 3:+:22811927-22888598 | 3:+:22811927-22888598 | Nek6 |
| ENSRNOG00000022709 | -3.26439841986184 | 0.041243 | down | 5:-:846658-848163 | 5:-:846658-848163 | AABR07046657.1 |
| ENSRNOG00000004649 | 2.173102 | 0.03976 | up | 3:-:121876263-121882726 | 3:-:121876263-121882726 | Il1b |
| ENSRNOG00000004314 | -1.13433294219016 | 0.041207 | down | 7:-:66843865-67116980 | 7:-:66843865-67116980 | Ppm1h |
| ENSRNOG00000008225 | -1.23124497478558 | 0.047709 | down | 6:+:76661416-76661824 | 6:+:76661416-76661824 | AABR07064349.1 |
| ENSRNOG00000005655 | 4.951019 | 0.045097 | up | 5:+:8459660-8574655 | 5:+:8459660-8574655 | Cpa6 |
| ENSRNOG00000045558 | -2.0384762346134 | 0.0473 | down | 13:+:113691932-113711647 | 13:+:113691932-113711647 | Cd34 |
| ENSRNOG00000019007 | 1.458684 | 0.040184 | up | 8:+:129240528-129243402 | 8:+:129240528-129243402 | Rpl14 |
| ENSRNOG00000025155 | -1.07969508763829 | 0.040068 | down | 12:-:12409876-12546111 | 12:-:12409876-12546111 | Lmtk2 |
| ENSRNOG00000050091 | 2.228626 | 0.04262 | up | 11:-:90221056-90234286 | 11:-:90221056-90234286 | Efcab1 |
| ENSRNOG00000038891 | 3.13524 | 0.043175 | up | 14:-:41663688-41786084 | 14:-:41663688-41786084 | Grxcr1 |
| ENSRNOG00000000137 | 1.860517 | 0.045764 | up | 17:-:28104589-28191436 | 17:-:28104589-28191436 | Ly86 |
| ENSRNOG00000018830 | -3.93412143534802 | 0.04793 | down | 9:-:44839960-45292047 | 9:-:44839960-45292047 | Aff3 |
| ENSRNOG00000047098 | -2.27187227770815 | 0.048074 | down | 1:+:168945449-168965566 | 1:+:168945449-168965566 | Hbb-bs |
| ENSRNOG00000012708 | -2.82610391209429 | 0.042122 | down | 9:-:21141077-21338582 | 9:-:21141077-21338582 | Ptchd4 |
| ENSRNOG00000023034 | -2.26859058406281 | 0.040553 | down | 18:+:51679768-51683102 | 18:+:51679768-51683102 | Tex43 |
| ENSRNOG00000016490 | -3.04873375687045 | 0.041865 | down | 1:-:44550994-44615760 | 1:-:44550994-44615760 | Nox3 |
| ENSRNOG00000025757 | 1.16181 | 0.048203 | up | 15:-:33605654-33629699 | 15:-:33605654-33629699 | Myh6 |
| ENSRNOG00000011009 | -3.64876870817866 | 0.048349 | down | 19:+:784618-824420 | 19:+:784618-824420 | Cmtm4 |
| ENSRNOG00000042679 | -1.45015016512717 | 0.047627 | down | 1:+:260732485-260830854 | 1:+:260732485-260830854 | Lcor |
| ENSRNOG00000009523 | -3.62328157099098 | 0.048448 | down | 1:-:281065168-281101438 | 1:-:281065168-281101438 | Rab11fip2 |
| ENSRNOG00000016245 | -1.32871544985788 | 0.043934 | down | 19:+:22450030-22486531 | 19:+:22450030-22486531 | Neto2 |
| ENSRNOG00000002991 | -1.75113726214048 | 0.048564 | down | 10:-:61756138-61772293 | 10:-:61756138-61772293 | Srr |
| ENSRNOG00000001926 | 2.050766 | 0.041861 | up | 11:+:77815181-77830416 | 11:+:77815181-77830416 | Cldn1 |
| ENSRNOG00000053081 | 2.168915 | 0.041035 | up | 2:+:198390166-198390576 | 2:+:198390166-198390576 | LOC102548682 |
| ENSRNOG00000032240 | 1.665157 | 0.047634 | up | 2:+:248178389-248197160 | 2:+:248178389-248197160 | Gbp5 |
| ENSRNOG00000022555 | 2.313126 | 0.043984 | up | 10:-:36709018-36716601 | 10:-:36709018-36716601 | LOC497899 |
| ENSRNOG00000015226 | -1.58322532413004 | 0.044416 | down | 2:-:202891537-203043847 | 2:-:202891537-203043847 | Man1a2 |
| ENSRNOG00000022242 | 2.732744 | 0.042054 | up | 14:+:17228856-17234712 | 14:+:17228856-17234712 | Cxcl9 |
| ENSRNOG00000022116 | 1.120463 | 0.042277 | up | 15:-:37400889-37410848 | 15:-:37400889-37410848 | Gjb6 |
| ENSRNOG00000049281 | -1.13198160053053 | 0.043624 | down | 2:+:188516582-188522601 | 2:+:188516582-188522601 | Gba |
| ENSRNOG00000001352 | -2.50256551009685 | 0.041488 | down | 12:-:40713841-40822159 | 12:-:40713841-40822159 | Hectd4 |
| ENSRNOG00000016804 | 3.059713 | 0.042998 | up | 15:+:33596301-33599685 | 15:+:33596301-33599685 | Il25 |
| ENSRNOG00000001572 | 3.067087 | 0.04282 | up | 3:+:61685619-61687758 | 3:+:61685619-61687758 | Hoxd1 |
| ENSRNOG00000014373 | -3.14110631252943 | 0.048185 | down | 1:-:174066911-174119815 | 1:-:174066911-174119815 | Trim66 |
| ENSRNOG00000040266 | -2.93386603674291 | 0.046257 | down | 6:-:3234090-3254779 | 6:-:3234090-3254779 | Cdkl4 |
| ENSRNOG00000031515 | 2.211952 | 0.044312 | up | 4:-:165424763-165460075 | 4:-:165424763-165460075 | Klra2 |
| ENSRNOG00000017120 | -1.15676869539337 | 0.043962 | down | 1:+:140998240-141087405 | 1:+:140998240-141087405 | Abhd2 |
| ENSRNOG00000011339 | -2.719916600091 | 0.04527 | down | 1:+:267359830-267412810 | 1:+:267359830-267412810 | Slk |
| ENSRNOG00000056071 | -2.30258245810654 | 0.042674 | down | X:+:115909168-115910880 | X:+:115909168-115910880 | AABR07040959.1 |
| ENSRNOG00000043350 | -1.48102186072725 | 0.042399 | down | 17:-:18442127-18592750 | 17:-:18442127-18592750 | Cap2 |
| ENSRNOG00000045999 | 1.936708 | 0.042175 | up | 9:-:10896680-10897240 | 9:-:10896680-10897240 | Tnfaip8l1 |
| ENSRNOG00000031669 | -2.82672434537217 | 0.044409 | down | 11:-:79205730-79703736 | 11:-:79205730-79703736 | Lpp |
| ENSRNOG00000003674 | 1.469576 | 0.049522 | up | X:-:31852323-31968152 | X:-:31852323-31968152 | Pir |
| ENSRNOG00000059262 | -2.99695657856935 | 0.046142 | down | 16:-:16454743-16473307 | 16:-:16454743-16473307 | AABR07024786.1 |
| ENSRNOG00000003603 | -1.9861547857913 | 0.04283 | down | 10:-:51502894-51669297 | 10:-:51502894-51669297 | Arhgap44 |
| ENSRNOG00000033498 | 1.338058 | 0.047636 | up | 1:-:142014958-142020525 | 1:-:142014958-142020525 | Cib1 |
| ENSRNOG00000032664 | -2.65793417938939 | 0.043225 | down | 20:+:3162039-3162939 | 20:+:3162039-3162939 | AABR07044362.2 |
| ENSRNOG00000017318 | -1.76559055989683 | 0.049361 | down | 17:+:44025267-44039284 | 17:+:44025267-44039284 | Zfp322a |
| ENSRNOG00000019780 | -2.52676304697547 | 0.044267 | down | 2:-:211003046-211017778 | 2:-:211003046-211017778 | Sypl2 |
| ENSRNOG00000017092 | -2.79394453852871 | 0.046664 | down | 17:+:16455026-16470842 | 17:+:16455026-16470842 | Zfp169 |
| ENSRNOG00000051789 | -2.4609785735571 | 0.044443 | down | 2:+:20349845-20350945 | 2:+:20349845-20350945 | AABR07007584.1 |
| ENSRNOG00000039463 | -2.82699916528004 | 0.046607 | down | 18:+:30808404-30810824 | 18:+:30808404-30810824 | Pcdhga1 |
| ENSRNOG00000060937 | -2.57392299731728 | 0.044506 | down | 4:-:12366293-12472658 | 4:-:12366293-12472658 | AABR07059258.1 |
| ENSRNOG00000029321 | -3.01620858055904 | 0.048691 | down | 4:+:91373942-91972325 | 4:+:91373942-91972325 | Ccser1 |
| ENSRNOG00000013663 | 1.051102 | 0.049568 | up | 1:+:103172987-103177417 | 1:+:103172987-103177417 | Tmem86a |
| ENSRNOG00000032180 | -2.39601538169238 | 0.044244 | down | 8:-:39762035-39830306 | 8:-:39762035-39830306 | Ccdc15 |
| ENSRNOG00000050123 | 2.93986 | 0.044356 | up | 3:-:151483249-151486693 | 3:-:151483249-151486693 | Gdf5 |
| ENSRNOG00000059295 | -1.56823746765238 | 0.045895 | down | 6:-:27473748-27512287 | 6:-:27473748-27512287 | Selenoi |
| ENSRNOG00000056617 | -2.97170571092342 | 0.049296 | down | 15:-:4006470-4022223 | 15:-:4006470-4022223 | Zswim8 |
| ENSRNOG00000001499 | 3.41822 | 0.046125 | up | 1:-:83991578-83993270 | 1:-:83991578-83993270 | Mia |
| ENSRNOG00000001259 | -1.04071637819109 | 0.044698 | down | 12:+:40018937-40219291 | 12:+:40018937-40219291 | Cux2 |
| ENSRNOG00000025059 | 1.498175 | 0.04998 | up | 7:-:70833663-70842405 | 7:-:70833663-70842405 | Nxph4 |
| ENSRNOG00000013364 | 1.153987 | 0.049269 | up | 1:+:29191192-29201531 | 1:+:29191192-29201531 | Hey2 |
| ENSRNOG00000062224 | -4.24932737965847 | 0.046825 | down | 2:+:184409306-184449404 | 2:+:184409306-184449404 | AABR07012058.1 |
| ENSRNOG00000056447 | 3.86819 | 0.046814 | up | 3:+:18787606-18787893 | 3:+:18787606-18787893 | AABR07051658.1 |
| ENSRNOG00000002364 | -1.49272482944751 | 0.043503 | down | 10:-:47719014-47725172 | 10:-:47719014-47725172 | Rnf112 |
| ENSRNOG00000009434 | 2.163258 | 0.046681 | up | 8:-:111727984-111777677 | 8:-:111727984-111777677 | RGD1310507 |
| ENSRNOG00000001056 | 1.126951 | 0.044253 | up | 12:-:2537275-2540668 | 12:-:2537275-2540668 | Snapc2 |
| ENSRNOG00000050100 | 3.765625 | 0.046535 | up | 14:-:34194217-34218961 | 14:-:34194217-34218961 | Exoc1l |
| ENSRNOG00000023657 | -2.7236938819998 | 0.047419 | down | 4:-:89693280-89695928 | 4:-:89693280-89695928 | Gprin3 |
| ENSRNOG00000030729 | 2.344894 | 0.04576 | up | 20:-:4302344-4316715 | 20:-:4302344-4316715 | C4b |
| ENSRNOG00000014863 | 1.458307 | 0.047933 | up | 5:+:59348639-59415135 | 5:+:59348639-59415135 | Reck |
| ENSRNOG00000059764 | -2.92546247593858 | 0.047174 | down | 1:-:195074330-195096460 | 1:-:195074330-195096460 | Snrpn |
| ENSRNOG00000021068 | -1.07933718079191 | 0.045384 | down | 2:-:196227670-196270826 | 2:-:196227670-196270826 | Pip5k1a |
| ENSRNOG00000005111 | -2.65440302331776 | 0.04715 | down | 3:-:103734435-103745236 | 3:-:103734435-103745236 | Nutm1 |
| ENSRNOG00000060123 | -1.13082895996214 | 0.043331 | down | 7:+:117723263-117730702 | 7:+:117723263-117730702 | Kifc2 |
| ENSRNOG00000027145 | -3.10128763315046 | 0.049461 | down | 8:+:75516904-75607212 | 8:+:75516904-75607212 | Rora |
| ENSRNOG00000010357 | 2.146079 | 0.045068 | up | 13:-:55861015-55878094 | 13:-:55861015-55878094 | Lhx9 |
| ENSRNOG00000059654 | -1.971685742586 | 0.046044 | down | 8:-:48801848-48802204 | 8:-:48801848-48802204 | AC105645.5 |
| ENSRNOG00000054632 | -2.47821354925002 | 0.046198 | down | 3:-:25429558-25430792 | 3:-:25429558-25430792 | AABR07051882.1 |
| ENSRNOG00000000857 | 2.883121 | 0.046473 | up | 20:-:5020667-5037088 | 20:-:5020667-5037088 | Msh5 |
| ENSRNOG00000060178 | -1.92270063156637 | 0.049349 | down | 2:+:184597104-184599418 | 2:+:184597104-184599418 | AABR07012061.1 |
| ENSRNOG00000016769 | 3.032853 | 0.046702 | up | 1:+:152072665-152153449 | 1:+:152072665-152153449 | Rab38 |
| ENSRNOG00000028016 | 2.182791 | 0.045744 | up | 13:+:70226647-70257576 | 13:+:70226647-70257576 | AABR07021402.1 |
| ENSRNOG00000037495 | 3.318163 | 0.04884 | up | 6:-:143590014-143590448 | 6:-:143590014-143590448 | AABR07065883.1 |
| ENSRNOG00000047384 | -2.29545499146146 | 0.045988 | down | 11:-:72083217-72086743 | 11:-:72083217-72086743 | Pigz |
| ENSRNOG00000059928 | 2.832603 | 0.049428 | up | 10:+:46182869-46183215 | 10:+:46182869-46183215 | AC097038.1 |
| ENSRNOG00000058301 | -1.64869833658038 | 0.048578 | down | 1:+:246926595-246926915 | 1:+:246926595-246926915 | 7SK |
| ENSRNOG00000013874 | 1.451533 | 0.042999 | up | 8:-:66862143-66863476 | 8:-:66862143-66863476 | Rplp1 |
| ENSRNOG00000018003 | -2.65737669815793 | 0.048902 | down | 2:-:25222328-25235275 | 2:-:25222328-25235275 | F2rl1 |
| ENSRNOG00000046527 | -1.00589122259843 | 0.047421 | down | 7:+:108613739-108756747 | 7:+:108613739-108756747 | Phf20l1 |
| ENSRNOG00000012424 | -2.33318145481029 | 0.046099 | down | 9:+:70133969-70290117 | 9:+:70133969-70290117 | Adam23 |
| ENSRNOG00000015403 | -1.9606148924336 | 0.048541 | down | 5:-:152322916-152324469 | 5:-:152322916-152324469 | Cd52 |
| ENSRNOG00000007778 | 3.203094 | 0.049373 | up | 10:-:55774006-55783489 | 10:-:55774006-55783489 | Alox15b |
| ENSRNOG00000014215 | -2.02041337968479 | 0.045754 | down | 1:+:240908483-240933198 | 1:+:240908483-240933198 | Klf9 |
| ENSRNOG00000030840 | -1.8515774086696 | 0.047627 | down | 11:-:4174881-4397361 | 11:-:4174881-4397361 | Cadm2 |
| ENSRNOG00000057978 | -2.52351554044755 | 0.048409 | down | 10:-:1741216-1744647 | 10:-:1741216-1744647 | AABR07028997.1 |
| ENSRNOG00000017477 | 2.414164 | 0.049565 | up | 5:-:173078590-173081839 | 5:-:173078590-173081839 | Mmp23 |
| ENSRNOG00000017132 | -1.30761107832128 | 0.048847 | down | 5:+:77052484-77157915 | 5:+:77052484-77157915 | Snx30 |
| ENSRNOG00000001349 | 1.174145 | 0.049187 | up | 12:-:19306607-19314016 | 12:-:19306607-19314016 | Mcm7 |
| ENSRNOG00000021891 | -1.1321612054552 | 0.046336 | down | 11:+:86903122-86915715 | 11:+:86903122-86915715 | Zdhhc8 |
| ENSRNOG00000019651 | 1.143419 | 0.048705 | up | 19:-:37917014-37938857 | 19:-:37917014-37938857 | Slc12a4 |
| ENSRNOG00000003384 | 2.262999 | 0.04995 | up | 10:-:50366952-50402616 | 10:-:50366952-50402616 | Hs3st3b1 |
| ENSRNOG00000008736 | 2.361827 | 0.049894 | up | 13:-:90967739-90977734 | 13:-:90967739-90977734 | Slamf8 |
| ENSRNOG00000012630 | 1.12006 | 0.049138 | up | 2:+:207271853-207279314 | 2:+:207271853-207279314 | Rhoc |
| ENSRNOG00000038955 | 2.236171 | 0.049429 | up | 18:+:55685613-55704904 | 18:+:55685613-55704904 | MGC105567 |
| ENSRNOG00000020308 | 1.069652 | 0.047347 | up | 1:+:87009730-87016005 | 1:+:87009730-87016005 | Ech1 |
| ENSRNOG00000002079 | -1.08102941138576 | 0.045857 | down | 14:+:8080275-8368254 | 14:+:8080275-8368254 | Mapk10 |
| ENSRNOG00000003251 | -1.25643534208259 | 0.049189 | down | 13:+:60435946-60444957 | 13:+:60435946-60444957 | B3galt2 |
| ENSRNOG00000005932 | -1.83594507593014 | 0.048397 | down | 5:-:86586996-86696388 | 5:-:86586996-86696388 | Megf9 |
| ENSRNOG00000060350 | -1.06676377284863 | 0.045877 | down | 4:+:123307624-123453478 | 4:+:123307624-123453478 | Iqsec1 |
| ENSRNOG00000053288 | -1.23186733251824 | 0.04589 | down | 20:+:20105047-20480623 | 20:+:20105047-20480623 | Ank3 |
| ENSRNOG00000008145 | -1.37310804470371 | 0.048462 | down | 6:+:135610743-135718564 | 6:+:135610743-135718564 | Traf3 |
| ENSRNOG00000014323 | -1.03152813644905 | 0.048436 | down | 2:+:218951141-218971886 | 2:+:218951141-218971886 | Extl2 |
| ENSRNOG00000056150 | -1.29253472914999 | 0.047259 | down | 14:-:86736878-86739335 | 14:-:86736878-86739335 | Purb |
| ENSRNOG00000058866 | -2.10961851821547 | 0.047244 | down | 8:+:82037977-82156617 | 8:+:82037977-82156617 | Myo5a |
| ENSRNOG00000027962 | -1.0301273322655 | 0.049086 | down | 13:-:70258915-70321752 | 13:-:70258915-70321752 | Smg7 |
| ENSRNOG00000005345 | -1.24457714552646 | 0.047034 | down | 6:-:37001356-37122023 | 6:-:37001356-37122023 | Vsnl1 |
| ENSRNOG00000030515 | -1.12427966171491 | 0.048263 | down | 13:-:49335408-49522415 | 13:-:49335408-49522415 | Nfasc |
| ENSRNOG00000004214 | 1.063846 | 0.048475 | up | 10:+:55492404-55496012 | 10:+:55492404-55496012 | Rpl26 |
| ENSRNOG00000004741 | 1.154621 | 0.048939 | up | 10:+:85978691-85981979 | 10:+:85978691-85981979 | Rpl19 |
| ENSRNOG00000020595 | 1.010268 | 0.048934 | up | 1:-:101116635-101118825 | 1:-:101116635-101118825 | Rps11 |
| ENSRNOG00000025589 | -1.42931459178148 | 0.049939 | down | 15:-:33768595-33775109 | 15:-:33768595-33775109 | Jph4 |
| ENSRNOG00000010488 | -1.37286603266165 | 0.049877 | down | 16:+:1749191-1954614 | 16:+:1749191-1954614 | Zmiz1 |

Abbreviations: DEGs: differentially expressed genes; VD: vascular dementia; CK: normal saline control; FC: fold change
