# Supplementary material for: Integrated transcriptome and metabolome analysis to investigate the mechanism of intranasal insulin treatment in a rat model of vascular dementia
Source: Front Pharmacol. 2023 May 15;14:1182803. doi: 10.3389/fphar.2023.1182803 (PMC10225696; doi:10.3389/fphar.2023.1182803)
Supplement: Supplementary file 6 [file Table5.docx]

Table S5 Top30 GO enrichment of DEGs between VD and CK groups

| Class | GO term | GO name | Deg number of this term |
| --- | --- | --- | --- |
| biological_process | GO:0009987 | cellular process | 615 |
| cellular_component | GO:0005623 | cell | 603 |
| cellular_component | GO:0044464 | cell part | 601 |
| biological_process | GO:0044699 | single-organism process | 593 |
| molecular_function | GO:0005488 | binding | 528 |
| cellular_component | GO:0043226 | organelle | 482 |
| biological_process | GO:0065007 | biological regulation | 476 |
| biological_process | GO:0050789 | regulation of biological process | 449 |
| cellular_component | GO:0016020 | membrane | 447 |
| biological_process | GO:0008152 | metabolic process | 431 |
| biological_process | GO:0050896 | response to stimulus | 392 |
| biological_process | GO:0032501 | multicellular organismal process | 345 |
| cellular_component | GO:0044425 | membrane part | 338 |
| biological_process | GO:0032502 | developmental process | 328 |
| biological_process | GO:0023052 | signaling | 297 |
| biological_process | GO:0051179 | localization | 293 |
| biological_process | GO:0071840 | cellular component organization or biogenesis | 273 |
| biological_process | GO:0048518 | positive regulation of biological process | 269 |
| cellular_component | GO:0005576 | extracellular region | 247 |
| cellular_component | GO:0044422 | organelle part | 241 |
| molecular_function | GO:0003824 | catalytic activity | 234 |
| cellular_component | GO:0044421 | extracellular region part | 231 |
| biological_process | GO:0048519 | negative regulation of biological process | 223 |
| cellular_component | GO:0032991 | macromolecular complex | 185 |
| biological_process | GO:0002376 | immune system process | 131 |
| biological_process | GO:0051704 | multi-organism process | 103 |
| biological_process | GO:0040011 | locomotion | 99 |
| cellular_component | GO:0045202 | synapse | 90 |
| cellular_component | GO:0030054 | cell junction | 87 |
| biological_process | GO:0022610 | biological adhesion | 85 |

Abbreviations: DEGs: differentially expressed genes; VD: vascular dementia; CK: normal saline control; GO: Gene Ontology
