# Supplementary material for: Integrated transcriptome and metabolome analysis to investigate the mechanism of intranasal insulin treatment in a rat model of vascular dementia
Source: Front Pharmacol. 2023 May 15;14:1182803. doi: 10.3389/fphar.2023.1182803 (PMC10225696; doi:10.3389/fphar.2023.1182803)
Supplement: Supplementary file 7 [file Table3.docx]

Table S3 DEGs between INS+VD and VD groups.

| ID | logFC | Pvalue | regulation | pos_info | ref_pos_info | symbol |
| --- | --- | --- | --- | --- | --- | --- |
| ENSRNOG00000057031 | 6.592853 | 3.56E-05 | up | 6:+:8886591-8889925 | 6:+:8886591-8889925 | Six3 |
| ENSRNOG00000025415 | 5.38052 | 0.000379 | up | 13:-:81988926-82006005 | 13:-:81988926-82006005 | Mettl11b |
| ENSRNOG00000007003 | 4.397863 | 0.000631 | up | 7:+:123308041-123361391 | 7:+:123308041-123361391 | Mei1 |
| ENSRNOG00000010412 | 3.883678 | 0.000655 | up | 5:+:61474000-61542653 | 5:+:61474000-61542653 | Ccdc180 |
| ENSRNOG00000058510 | 4.506364 | 0.000763 | up | 2:+:8860250-8884105 | 2:+:8860250-8884105 | AC127140.1 |
| ENSRNOG00000007830 | -1.79796604954472 | 0.000774 | down | 4:+:168752133-168755023 | 4:+:168752133-168755023 | Apold1 |
| ENSRNOG00000008431 | 2.111074 | 0.000953 | up | 5:-:62276100-62621737 | 5:-:62276100-62621737 | Gabbr2 |
| ENSRNOG00000026309 | -4.88935011102819 | 0.000979 | down | 4:-:157294287-157304653 | 4:-:157294287-157304653 | LOC100911585 |
| ENSRNOG00000003745 | -3.00510116446397 | 0.001037 | down | 13:-:109817728-109849632 | 13:-:109817728-109849632 | Atf3 |
| ENSRNOG00000027341 | 2.165614 | 0.001084 | up | 10:-:20844899-21265026 | 10:-:20844899-21265026 | Tenm2 |
| ENSRNOG00000056643 | 1.832486 | 0.001111 | up | 19:+:6046665-6426216 | 19:+:6046665-6426216 | Cdh8 |
| ENSRNOG00000050910 | -2.33643135981435 | 0.00113 | down | 17:+:36690249-36694325 | 17:+:36690249-36694325 | LOC688583 |
| ENSRNOG00000008644 | 6.182498 | 0.001164 | up | 6:-:77418096-77421286 | 6:-:77418096-77421286 | Nkx2-1 |
| ENSRNOG00000015741 | 1.865551 | 0.00122 | up | 7:-:132430853-132757558 | 7:-:132430853-132757558 | Slc2a13 |
| ENSRNOG00000032660 | 1.743068 | 0.001331 | up | 2:-:258792839-258997145 | 2:-:258792839-258997145 | Adgrl2 |
| ENSRNOG00000050877 | 3.427845 | 0.001429 | up | 2:+:9683570-9696050 | 2:+:9683570-9696050 | LOC100912538 |
| ENSRNOG00000004535 | 4.176859 | 0.001456 | up | 6:-:6794808-6842758 | 6:-:6794808-6842758 | Kcng3 |
| ENSRNOG00000000768 | -4.012199057872 | 0.001526 | down | 20:-:1876173-1897814 | 20:-:1876173-1897814 | Ubd |
| ENSRNOG00000012703 | -2.33050337397809 | 0.001621 | down | 2:-:104459999-104461863 | 2:-:104459999-104461863 | Crh |
| ENSRNOG00000012481 | -5.50628233100338 | 0.001727 | down | 2:+:207262934-207268609 | 2:+:207262934-207268609 | Ppm1j |
| ENSRNOG00000059746 | 3.957262 | 0.001741 | up | X:-:129223138-129336004 | X:-:129223138-129336004 | AABR07041411.1 |
| ENSRNOG00000002217 | -3.27787327635484 | 0.001843 | down | 14:+:10692764-10714524 | 14:+:10692764-10714524 | Plac8 |
| ENSRNOG00000024435 | 2.547465 | 0.001894 | up | 12:+:28381982-29268073 | 12:+:28381982-29268073 | Galnt17 |
| ENSRNOG00000004560 | 1.856953 | 0.001951 | up | 3:-:1740024-1924827 | 3:-:1740024-1924827 | Cacna1b |
| ENSRNOG00000007636 | 1.573557 | 0.002166 | up | 4:-:50326442-50860756 | 4:-:50326442-50860756 | Cadps2 |
| ENSRNOG00000014940 | -5.87752245519879 | 0.002334 | down | 1:-:261442108-261446570 | 1:-:261442108-261446570 | Sfrp5 |
| ENSRNOG00000057344 | -5.36465963811914 | 0.002369 | down | 3:-:20479688-20479999 | 3:-:20479688-20479999 | AABR07051733.2 |
| ENSRNOG00000047057 | 3.124492 | 0.00238 | up | 1:+:203971152-203988709 | 1:+:203971152-203988709 | Gpr26 |
| ENSRNOG00000032206 | 4.782372 | 0.002426 | up | 13:+:21678512-22590586 | 13:+:21678512-22590586 | Cntnap5b |
| ENSRNOG00000019134 | 4.273111 | 0.00243 | up | 18:+:57654290-57819698 | 18:+:57654290-57819698 | Htr4 |
| ENSRNOG00000012720 | -4.79622048020061 | 0.002456 | down | 1:-:32634796-32643771 | 1:-:32634796-32643771 | Irx4 |
| ENSRNOG00000018305 | 1.966955 | 0.002492 | up | 18:+:59748444-59755226 | 18:+:59748444-59755226 | St8sia3 |
| ENSRNOG00000018892 | 4.103512 | 0.002517 | up | 10:-:88669214-88670430 | 10:-:88669214-88670430 | Hcrt |
| ENSRNOG00000021823 | 3.372859 | 0.002649 | up | 15:-:51174989-51176858 | 15:-:51174989-51176858 | AABR07018318.1 |
| ENSRNOG00000061132 | 3.53498 | 0.002686 | up | 18:-:44029927-44141865 | 18:-:44029927-44141865 | LOC103694210 |
| ENSRNOG00000000814 | -2.51365795366935 | 0.002804 | down | 20:+:40769586-40773349 | 20:+:40769586-40773349 | Fabp7 |
| ENSRNOG00000015863 | 5.025698 | 0.002829 | up | 8:+:25246292-25482647 | 8:+:25246292-25482647 | Npsr1 |
| ENSRNOG00000016225 | -1.9116072395397 | 0.002902 | down | 17:+:15749978-15800401 | 17:+:15749978-15800401 | Fgd3 |
| ENSRNOG00000004606 | -1.99109931362912 | 0.003026 | down | 14:-:103181281-103321270 | 14:-:103181281-103321270 | Meis1 |
| ENSRNOG00000017178 | 2.411757 | 0.003141 | up | 19:-:41036510-41379588 | 19:-:41036510-41379588 | Hydin |
| ENSRNOG00000015773 | -3.34973680581509 | 0.003188 | down | 1:+:196996581-197024166 | 1:+:196996581-197024166 | Il21r |
| ENSRNOG00000015550 | -2.06125218009193 | 0.00326 | down | 3:-:2686123-2689084 | 3:-:2686123-2689084 | Ptgds |
| ENSRNOG00000019869 | 1.425639 | 0.003291 | up | 1:-:85291462-85300825 | 1:-:85291462-85300825 | Lrfn1 |
| ENSRNOG00000000825 | -3.42721050843209 | 0.003325 | down | 20:-:27651312-27657983 | 20:-:27651312-27657983 | Calhm5 |
| ENSRNOG00000022162 | -2.48776834433889 | 0.003407 | down | 3:-:13182024-13525983 | 3:-:13182024-13525983 | Pbx3 |
| ENSRNOG00000023708 | -1.41218669534533 | 0.003512 | down | 4:+:78458625-78462423 | 4:+:78458625-78462423 | Tmem176a |
| ENSRNOG00000010031 | -1.50448601285483 | 0.003527 | down | 10:+:65767053-65771038 | 10:+:65767053-65771038 | Vtn |
| ENSRNOG00000012697 | 3.597047 | 0.003563 | up | 3:-:7269853-7278758 | 3:-:7269853-7278758 | Spaca9 |
| ENSRNOG00000027489 | 3.078735 | 0.003614 | up | 12:-:51214192-51250230 | 12:-:51214192-51250230 | Mn1 |
| ENSRNOG00000047085 | 1.857482 | 0.003708 | up | 18:-:28012877-28017925 | 18:-:28012877-28017925 | Lrrtm2 |
| ENSRNOG00000046057 | -5.40847706476239 | 0.003738 | down | 10:+:86340940-86342858 | 10:+:86340940-86342858 | Pnmt |
| ENSRNOG00000002549 | -2.46312998605877 | 0.003765 | down | 13:-:37479758-37492680 | 13:-:37479758-37492680 | Htr5b |
| ENSRNOG00000053168 | 4.094326 | 0.00391 | up | 2:+:230476320-230479594 | 2:+:230476320-230479594 | LOC102553088 |
| ENSRNOG00000032569 | 3.033925 | 0.004083 | up | 7:+:11737293-11746703 | 7:+:11737293-11746703 | Lingo3 |
| ENSRNOG00000062158 | 2.298929 | 0.004113 | up | 1:-:221158098-221158792 | 1:-:221158098-221158792 | AC134224.3 |
| ENSRNOG00000024277 | -2.72515728080373 | 0.00427 | down | 12:+:15700825-15844512 | 12:+:15700825-15844512 | AABR07035470.1 |
| ENSRNOG00000013232 | 3.371261 | 0.004449 | up | 3:-:23033775-23066658 | 3:-:23033775-23066658 | Nr6a1 |
| ENSRNOG00000059401 | 3.54402 | 0.004456 | up | 10:+:108340240-108372862 | 10:+:108340240-108372862 | Ccdc40 |
| ENSRNOG00000047384 | 2.932046 | 0.004477 | up | 11:-:72083217-72086743 | 11:-:72083217-72086743 | Pigz |
| ENSRNOG00000018141 | 2.302403 | 0.00448 | up | 17:-:42165817-42226377 | 17:-:42165817-42226377 | RGD1307443 |
| ENSRNOG00000003209 | -2.42213356282283 | 0.004486 | down | 13:-:89542378-89565813 | 13:-:89542378-89565813 | Pcp4l1 |
| ENSRNOG00000027731 | 3.450909 | 0.00449 | up | 5:-:157159936-157165767 | 5:-:157159936-157165767 | Ubxn10 |
| ENSRNOG00000051905 | 3.966968 | 0.004522 | up | 4:-:45292437-45332420 | 4:-:45292437-45332420 | Wnt2 |
| ENSRNOG00000004772 | -2.86268992745976 | 0.004749 | down | 3:-:44150992-44177689 | 3:-:44150992-44177689 | Cytip |
| ENSRNOG00000011250 | -4.75356250473157 | 0.004788 | down | 4:-:85381889-85386231 | 4:-:85381889-85386231 | Inmt |
| ENSRNOG00000028801 | -4.68969248461916 | 0.004976 | down | 4:-:10423139-10517848 | 4:-:10423139-10517848 | Gsap |
| ENSRNOG00000009047 | 2.921902 | 0.005 | up | 8:+:58431407-58435654 | 8:+:58431407-58435654 | Sln |
| ENSRNOG00000029980 | 2.799943 | 0.005056 | up | 8:-:52985313-53146953 | 8:-:52985313-53146953 | Zbtb16 |
| ENSRNOG00000054484 | 3.585049 | 0.005067 | up | 14:-:78393083-78398305 | 14:-:78393083-78398305 | AABR07015812.2 |
| ENSRNOG00000009881 | 3.039754 | 0.005078 | up | 14:+:107785029-107798115 | 14:+:107785029-107798115 | Fam161a |
| ENSRNOG00000047635 | 1.521744 | 0.005129 | up | 4:+:68011932-68059249 | 4:+:68011932-68059249 | Tmem178b |
| ENSRNOG00000007159 | -3.66277937458546 | 0.005137 | down | 10:+:69412017-69413870 | 10:+:69412017-69413870 | Ccl2 |
| ENSRNOG00000024526 | 2.97572 | 0.00517 | up | 5:+:172259520-172269213 | 5:+:172259520-172269213 | Ttc34 |
| ENSRNOG00000032401 | -1.5280785955066 | 0.005191 | down | 7:+:94777702-94778696 | 7:+:94777702-94778696 | H3f3c |
| ENSRNOG00000047387 | 3.465845 | 0.005398 | up | 1:-:59297294-59347472 | 1:-:59297294-59347472 | Lnpep |
| ENSRNOG00000006532 | -1.29978560407786 | 0.005422 | down | 10:-:104573664-104575890 | 10:-:104573664-104575890 | H3f3a |
| ENSRNOG00000024114 | 2.838468 | 0.005455 | up | 9:+:70059683-70089778 | 9:+:70059683-70089778 | Zdbf2 |
| ENSRNOG00000057840 | 3.598224 | 0.005471 | up | 16:+:77995246-78016003 | 16:+:77995246-78016003 | AABR07026473.1 |
| ENSRNOG00000038980 | 1.666899 | 0.00552 | up | 3:+:35679750-35707410 | 3:+:35679750-35707410 | Lypd6 |
| ENSRNOG00000011313 | 1.765969 | 0.005577 | up | 1:-:269973351-270472866 | 1:-:269973351-270472866 | Sorcs1 |
| ENSRNOG00000002256 | -2.45258398485119 | 0.005627 | down | 14:-:17143040-17225389 | 14:-:17143040-17225389 | Art3 |
| ENSRNOG00000020982 | -3.05953623429694 | 0.005656 | down | 1:+:221420271-221421842 | 1:+:221420271-221421842 | LOC687780 |
| ENSRNOG00000008855 | -1.45951563690679 | 0.005747 | down | 15:-:37377316-37383277 | 15:-:37377316-37383277 | Gjb2 |
| ENSRNOG00000014893 | 4.602494 | 0.005848 | up | 2:-:251912368-251970768 | 2:-:251912368-251970768 | Wdr63 |
| ENSRNOG00000005233 | 3.061486 | 0.005914 | up | 7:-:107130402-107223047 | 7:-:107130402-107223047 | Lrrc6 |
| ENSRNOG00000050000 | -5.95234248011717 | 0.005919 | down | 11:+:86092468-86092779 | 11:+:86092468-86092779 | AABR07034739.1 |
| ENSRNOG00000032328 | 2.297536 | 0.005993 | up | 17:+:12762752-12763952 | 17:+:12762752-12763952 | Diras2 |
| ENSRNOG00000021231 | 2.03909 | 0.006086 | up | 3:-:123175860-123179644 | 3:-:123175860-123179644 | Lzts3 |
| ENSRNOG00000013515 | 1.322769 | 0.006111 | up | 5:-:149922352-149996334 | 5:-:149922352-149996334 | Ptpru |
| ENSRNOG00000001142 | -1.48387974996539 | 0.006148 | down | 12:+:46316236-46326790 | 12:+:46316236-46326790 | Prkab1 |
| ENSRNOG00000054917 | 2.573226 | 0.006155 | up | 1:-:216970254-216971183 | 1:-:216970254-216971183 | Mrgpre |
| ENSRNOG00000018143 | -1.99222666477704 | 0.006181 | down | 5:-:135675832-135677432 | 5:-:135675832-135677432 | Hpdl |
| ENSRNOG00000050706 | 4.442556 | 0.006332 | up | 2:+:235596123-235715057 | 2:+:235596123-235715057 | Col25a1 |
| ENSRNOG00000016239 | -1.94530977347932 | 0.006436 | down | 18:+:80939875-80949226 | 18:+:80939875-80949226 | Zadh2 |
| ENSRNOG00000005018 | 1.633785 | 0.006544 | up | 3:+:51687809-51822250 | 3:+:51687809-51822250 | Scn2a |
| ENSRNOG00000059840 | 2.145724 | 0.006645 | up | 17:-:10153994-10208360 | 17:-:10153994-10208360 | Unc5a |
| ENSRNOG00000054984 | 3.968379 | 0.006865 | up | 16:+:78236947-78404678 | 16:+:78236947-78404678 | AABR07026483.1 |
| ENSRNOG00000004737 | -1.22069504706395 | 0.007093 | down | 13:+:90116843-90140371 | 13:+:90116843-90140371 | Cd48 |
| ENSRNOG00000016997 | 3.796942 | 0.007151 | up | 20:-:6150814-6160963 | 20:-:6150814-6160963 | AABR07044442.1 |
| ENSRNOG00000030930 | -4.16740785194277 | 0.007174 | down | 11:-:14253848-14304725 | 11:-:14253848-14304725 | Samsn1 |
| ENSRNOG00000002873 | 3.778749 | 0.007206 | up | 10:-:44736545-44746549 | 10:-:44736545-44746549 | Fam183a |
| ENSRNOG00000053045 | 2.508406 | 0.007235 | up | 12:-:48817320-48857724 | 12:-:48817320-48857724 | Wscd2 |
| ENSRNOG00000020588 | -3.31283265311521 | 0.007252 | down | 2:-:188655912-188660179 | 2:-:188655912-188660179 | Efna4 |
| ENSRNOG00000005956 | 3.046724 | 0.007266 | up | 7:+:73222730-73242899 | 7:+:73222730-73242899 | Erich5 |
| ENSRNOG00000033099 | 1.794638 | 0.007275 | up | 18:-:66523120-67224566 | 18:-:66523120-67224566 | Dcc |
| ENSRNOG00000052555 | -3.10266747329167 | 0.00731 | down | 2:+:198388809-198389201 | 2:+:198388809-198389201 | Hist2h2aa2 |
| ENSRNOG00000001313 | 1.535747 | 0.007314 | up | 20:-:14593819-14620019 | 20:-:14593819-14620019 | Gnaz |
| ENSRNOG00000043451 | -4.62146543340676 | 0.007391 | down | 14:-:6673686-6679901 | 14:-:6673686-6679901 | Spp1 |
| ENSRNOG00000011132 | 2.897354 | 0.00747 | up | 3:-:94064234-94182714 | 3:-:94064234-94182714 | LOC100362814 |
| ENSRNOG00000001953 | -1.5624288659734 | 0.007576 | down | 11:+:37798370-37880821 | 11:+:37798370-37880821 | Bace2 |
| ENSRNOG00000009708 | 3.036827 | 0.007753 | up | 5:-:169200287-169212170 | 5:-:169200287-169212170 | Tas1r1 |
| ENSRNOG00000003741 | 2.105092 | 0.007757 | up | 10:-:108682638-108691367 | 10:-:108682638-108691367 | Nptx1 |
| ENSRNOG00000045771 | 2.605812 | 0.007812 | up | 4:+:70252366-70330803 | 4:+:70252366-70330803 | Chl1 |
| ENSRNOG00000011858 | 4.531988 | 0.007823 | up | 16:+:67350539-67595225 | 16:+:67350539-67595225 | Unc5d |
| ENSRNOG00000013017 | 1.065001 | 0.007825 | up | 1:-:146399217-146556171 | 1:-:146399217-146556171 | Arnt2 |
| ENSRNOG00000018488 | -3.07918699084477 | 0.007878 | down | 3:+:161519743-161534704 | 3:+:161519743-161534704 | Cd40 |
| ENSRNOG00000017003 | 1.385251 | 0.00792 | up | 4:-:121217629-121257885 | 4:-:121217629-121257885 | Plxna1 |
| ENSRNOG00000018680 | -3.05823064411738 | 0.007945 | down | 18:+:70970596-70975494 | 18:+:70970596-70975494 | Rpl17 |
| ENSRNOG00000005505 | 2.332132 | 0.008117 | up | 4:-:170154227-170186942 | 4:-:170154227-170186942 | Wbp11 |
| ENSRNOG00000000129 | 1.887907 | 0.008195 | up | 5:+:58359498-58383070 | 5:+:58359498-58383070 | Phf24 |
| ENSRNOG00000051915 | 1.609643 | 0.008216 | up | 1:-:88103719-88112683 | 1:-:88103719-88112683 | Spred3 |
| ENSRNOG00000008145 | 1.823904 | 0.008272 | up | 6:+:135610743-135718564 | 6:+:135610743-135718564 | Traf3 |
| ENSRNOG00000033697 | -2.17087977310551 | 0.008355 | down | 8:+:2635851-2651652 | 8:+:2635851-2651652 | Casp4 |
| ENSRNOG00000011054 | -1.1861202512722 | 0.00836 | down | 5:+:149047681-149069719 | 5:+:149047681-149069719 | Laptm5 |
| ENSRNOG00000002051 | 1.08553 | 0.008441 | up | 11:-:31104787-31180642 | 11:-:31104787-31180642 | Synj1 |
| ENSRNOG00000023951 | 1.561168 | 0.00853 | up | 7:-:63361696-63407241 | 7:-:63361696-63407241 | Tbc1d30 |
| ENSRNOG00000005726 | 2.643962 | 0.008573 | up | 4:-:16454904-17058921 | 4:-:16454904-17058921 | Pclo |
| ENSRNOG00000032618 | 3.347027 | 0.008689 | up | 8:+:116686601-116700132 | 8:+:116686601-116700132 | Mst1r |
| ENSRNOG00000053450 | 1.280702 | 0.00875 | up | 1:-:64438472-64446818 | 1:-:64438472-64446818 | Myadm |
| ENSRNOG00000000842 | -1.55173346065437 | 0.008772 | down | 20:+:5049496-5052585 | 20:+:5049496-5052585 | Ddah2 |
| ENSRNOG00000013610 | 2.201004 | 0.008774 | up | 8:+:59561721-59590172 | 8:+:59561721-59590172 | Chrna5 |
| ENSRNOG00000029682 | -1.38378762314364 | 0.008825 | down | 20:+:5040337-5049166 | 20:+:5040337-5049166 | Clic1 |
| ENSRNOG00000009884 | -2.17022782179086 | 0.008882 | down | 7:+:120153184-120156289 | 7:+:120153184-120156289 | Lgals1 |
| ENSRNOG00000006365 | 5.695223 | 0.009075 | up | 4:+:51614676-51644492 | 4:+:51614676-51644492 | Asb15 |
| ENSRNOG00000021225 | 3.747428 | 0.009183 | up | 3:+:123106694-123107534 | 3:+:123106694-123107534 | Oxt |
| ENSRNOG00000046666 | 3.173489 | 0.009195 | up | 18:-:74187162-74198369 | 18:-:74187162-74198369 | Haus1 |
| ENSRNOG00000010362 | -1.59903623315684 | 0.009203 | down | 8:+:75687100-75723594 | 8:+:75687100-75723594 | Anxa2 |
| ENSRNOG00000000569 | -1.12101178266704 | 0.009269 | down | 20:+:29897594-29919998 | 20:+:29897594-29919998 | Vsir |
| ENSRNOG00000046889 | -1.72631465022343 | 0.0093 | down | 13:-:36117119-36174908 | 13:-:36117119-36174908 | Dbi |
| ENSRNOG00000059469 | 4.625519 | 0.00933 | up | 14:-:106861676-106864892 | 14:-:106861676-106864892 | Otx1 |
| ENSRNOG00000013920 | 2.595799 | 0.009376 | up | 18:-:31812109-32207801 | 18:-:31812109-32207801 | Arhgap26 |
| ENSRNOG00000018019 | 1.596919 | 0.009397 | up | 1:-:279946811-280015358 | 1:-:279946811-280015358 | Hspa12a |
| ENSRNOG00000018330 | -3.00736021928348 | 0.009509 | down | 7:+:121889157-121918772 | 7:+:121889157-121918772 | Fam83f |
| ENSRNOG00000000777 | -1.29309003219597 | 0.00951 | down | 20:+:3176107-3179818 | 20:+:3176107-3179818 | RT1-S3 |
| ENSRNOG00000015999 | -1.20201561326305 | 0.009593 | down | 7:-:12401207-12405022 | 7:-:12401207-12405022 | Cirbp |
| ENSRNOG00000057404 | -3.40850678526451 | 0.009636 | down | 10:-:47632192-47666921 | 10:-:47632192-47666921 | Slc47a1 |
| ENSRNOG00000002369 | 1.455907 | 0.009693 | up | 13:+:71086745-71141075 | 13:+:71086745-71141075 | Rgs8 |
| ENSRNOG00000042353 | -3.08937688182234 | 0.009749 | down | 19:-:43506976-43528851 | 19:-:43506976-43528851 | Mlkl |
| ENSRNOG00000005359 | 1.747518 | 0.009767 | up | 3:+:51883559-52120290 | 3:+:51883559-52120290 | Csrnp3 |
| ENSRNOG00000014064 | -1.45177083966447 | 0.009767 | down | 8:+:97439161-97458287 | 8:+:97439161-97458287 | Ctsh |
| ENSRNOG00000013907 | -1.66784303550961 | 0.009778 | down | 19:+:23389375-23405039 | 19:+:23389375-23405039 | Sall1 |
| ENSRNOG00000005309 | 2.585183 | 0.00979 | up | 7:+:142575672-142684114 | 7:+:142575672-142684114 | Scn8a |
| ENSRNOG00000061304 | 1.247011 | 0.009817 | up | X:-:157239306-157312028 | X:-:157239306-157312028 | Atp2b3 |
| ENSRNOG00000045747 | 2.645684 | 0.009823 | up | 1:-:91058529-91063928 | 1:-:91058529-91063928 | Capns1 |
| ENSRNOG00000005332 | -1.92263070661875 | 0.009918 | down | 7:+:123168811-123183335 | 7:+:123168811-123183335 | Csdc2 |
| ENSRNOG00000053391 | -3.72911632262388 | 0.009933 | down | 18:+:78282423-78283480 | 18:+:78282423-78283480 | AABR07032724.1 |
| ENSRNOG00000033688 | 1.185657 | 0.009939 | up | 6:+:137288810-137311932 | 6:+:137288810-137311932 | Cep170b |
| ENSRNOG00000020151 | -3.66685934167327 | 0.009941 | down | 19:+:38768467-38838395 | 19:+:38768467-38838395 | Cdh1 |
| ENSRNOG00000005450 | 1.871774 | 0.009954 | up | 10:-:31028697-31041626 | 10:-:31028697-31041626 | Lsm11 |
| ENSRNOG00000036475 | -2.35916687479086 | 0.009974 | down | 5:+:115225263-115225366 | 5:+:115225263-115225366 | AABR07049302.1 |
| ENSRNOG00000033490 | 3.872669 | 0.010031 | up | 8:+:40009691-40014734 | 8:+:40009691-40014734 | Vsig2 |
| ENSRNOG00000004956 | 1.818743 | 0.010086 | up | 10:-:37270183-37311625 | 10:-:37270183-37311625 | Jade2 |
| ENSRNOG00000016945 | -3.34889108616473 | 0.010247 | down | 5:+:157282669-157285328 | 5:+:157282669-157285328 | Pla2g2a |
| ENSRNOG00000010992 | -4.04231076634201 | 0.010286 | down | 2:-:45517789-45518502 | 2:-:45517789-45518502 | Hspb3 |
| ENSRNOG00000005985 | 1.629995 | 0.010329 | up | 15:+:344360-1047956 | 15:+:344360-1047956 | Kcnma1 |
| ENSRNOG00000005003 | 1.278259 | 0.010375 | up | 6:+:144384773-145133042 | 6:+:144384773-145133042 | Ptprn2 |
| ENSRNOG00000002468 | 2.577805 | 0.010495 | up | 13:+:77485113-77678437 | 13:+:77485113-77678437 | Tnr |
| ENSRNOG00000011624 | 2.782987 | 0.010566 | up | 3:-:176589906-176644951 | 3:-:176589906-176644951 | Kcnq2 |
| ENSRNOG00000023375 | -1.29258397603406 | 0.010689 | down | 10:+:65448950-65452177 | 10:+:65448950-65452177 | Rab34 |
| ENSRNOG00000003622 | -1.77556432577022 | 0.010769 | down | X:+:14578264-14612547 | X:+:14578264-14612547 | Cybb |
| ENSRNOG00000019592 | 3.223655 | 0.01081 | up | 9:-:97183214-97290639 | 9:-:97183214-97290639 | Iqca |
| ENSRNOG00000022946 | 2.995012 | 0.010865 | up | 1:+:48433079-48521256 | 1:+:48433079-48521256 | Slc22a3 |
| ENSRNOG00000014048 | 1.350524 | 0.010899 | up | 19:-:19265164-19315357 | 19:-:19265164-19315357 | Cyld |
| ENSRNOG00000013042 | 4.181873 | 0.010907 | up | 8:-:89129453-89130991 | 8:-:89129453-89130991 | Htr1b |
| ENSRNOG00000002626 | 1.624772 | 0.011003 | up | 10:+:80790168-81296363 | 10:+:80790168-81296363 | Car10 |
| ENSRNOG00000013991 | 2.505293 | 0.01106 | up | 9:-:46360061-46401911 | 9:-:46360061-46401911 | Creg2 |
| ENSRNOG00000060687 | 2.108301 | 0.011099 | up | 3:+:139695028-139833967 | 3:+:139695028-139833967 | Slc24a3 |
| ENSRNOG00000058011 | -3.9224146720684 | 0.011103 | down | 15:+:48976146-48977111 | 15:+:48976146-48977111 | AABR07018244.2 |
| ENSRNOG00000010720 | 3.422789 | 0.011244 | up | 2:-:32444565-32518643 | 2:-:32444565-32518643 | Mast4 |
| ENSRNOG00000004554 | -1.3402478323025 | 0.011272 | down | 7:+:38742051-38782323 | 7:+:38742051-38782323 | Dcn |
| ENSRNOG00000001959 | -2.52262415922286 | 0.011288 | down | 11:-:37891156-37914983 | 11:-:37891156-37914983 | Mx1 |
| ENSRNOG00000012876 | -1.68599185609698 | 0.011512 | down | 4:+:153874852-153912155 | 4:+:153874852-153912155 | Slc6a13 |
| ENSRNOG00000028404 | 1.681001 | 0.011578 | up | 10:+:86303727-86312762 | 10:+:86303727-86312762 | Ppp1r1b |
| ENSRNOG00000008930 | 3.771657 | 0.011664 | up | 5:-:79553742-79570073 | 5:-:79553742-79570073 | Tnfsf15 |
| ENSRNOG00000011000 | 2.793668 | 0.011772 | up | 9:-:28442229-28973246 | 9:-:28442229-28973246 | Rims1 |
| ENSRNOG00000046639 | -1.9296318142796 | 0.011786 | down | 10:+:59539405-59711874 | 10:+:59539405-59711874 | Itgae |
| ENSRNOG00000023643 | 2.575537 | 0.011869 | up | 12:-:30702571-30728655 | 12:-:30702571-30728655 | Mmp17 |
| ENSRNOG00000005206 | 2.059037 | 0.011931 | up | 7:-:106717229-107009330 | 7:-:106717229-107009330 | Kcnq3 |
| ENSRNOG00000013436 | 2.190483 | 0.011966 | up | 1:-:15893533-16203909 | 1:-:15893533-16203909 | Pde7b |
| ENSRNOG00000043085 | 1.969277 | 0.011968 | up | 8:-:105323837-105462168 | 8:-:105323837-105462168 | Clstn2 |
| ENSRNOG00000009264 | 1.080909 | 0.011996 | up | 4:-:152087379-152380184 | 4:-:152087379-152380184 | Erc1 |
| ENSRNOG00000023151 | 3.108182 | 0.012 | up | 1:+:225899313-225902676 | 1:+:225899313-225902676 | Scgb2a1 |
| ENSRNOG00000058217 | 2.834509 | 0.01207 | up | X:-:129480906-129561668 | X:-:129480906-129561668 | AABR07041418.1 |
| ENSRNOG00000001484 | 1.652839 | 0.012092 | up | 12:-:25601404-25638797 | 12:-:25601404-25638797 | Castor2 |
| ENSRNOG00000051204 | 1.150467 | 0.01217 | up | 11:+:33925498-34027435 | 11:+:33925498-34027435 | Dop1b |
| ENSRNOG00000047211 | 1.417282 | 0.012205 | up | 15:-:48601266-48670257 | 15:-:48601266-48670257 | Fzd3 |
| ENSRNOG00000057557 | 3.704408 | 0.012285 | up | 2:+:60131776-60325692 | 2:+:60131776-60325692 | Prlr |
| ENSRNOG00000030069 | -3.13770275518122 | 0.012303 | down | 8:+:106816152-106827580 | 8:+:106816152-106827580 | Faim |
| ENSRNOG00000058047 | -4.21465357910679 | 0.012346 | down | 1:-:24854907-24858316 | 1:-:24854907-24858316 | AABR07000733.1 |
| ENSRNOG00000055498 | 2.873736 | 0.012355 | up | AABR07024104.1:-:57351-59242 | AABR07024104.1:-:57351-59242 | LOC103694875 |
| ENSRNOG00000000886 | 2.736181 | 0.012383 | up | 12:-:29308341-29743705 | 12:-:29308341-29743705 | Caln1 |
| ENSRNOG00000058678 | 2.497418 | 0.012488 | up | 20:-:4964125-4966710 | 20:-:4964125-4966710 | AABR07072810.1 |
| ENSRNOG00000006831 | 4.086924 | 0.012663 | up | 8:+:7128656-7187796 | 8:+:7128656-7187796 | Pgr |
| ENSRNOG00000016957 | -1.63484436964223 | 0.012686 | down | 9:+:80118029-80144789 | 9:+:80118029-80144789 | Igfbp2 |
| ENSRNOG00000029865 | -5.99837770206829 | 0.012798 | down | 9:+:94279155-94283919 | 9:+:94279155-94283919 | Prss56 |
| ENSRNOG00000026793 | 2.829871 | 0.012853 | up | 7:+:27309966-27363394 | 7:+:27309966-27363394 | Nt5dc3 |
| ENSRNOG00000055226 | 1.190256 | 0.012974 | up | 3:+:14889510-15060286 | 3:+:14889510-15060286 | Dab2ip |
| ENSRNOG00000025787 | 3.40488 | 0.01306 | up | 11:+:88424414-88508601 | 11:+:88424414-88508601 | Spag6l |
| ENSRNOG00000043304 | -1.37674970258528 | 0.013265 | down | 18:+:58270410-58301002 | 18:+:58270410-58301002 | Apcdd1 |
| ENSRNOG00000024536 | 3.021887 | 0.013301 | up | 18:-:61759289-61788863 | 18:-:61759289-61788863 | AABR07032338.1 |
| ENSRNOG00000031930 | -1.71934982408862 | 0.013329 | down | 7:-:142273833-142300382 | 7:-:142273833-142300382 | Bin2 |
| ENSRNOG00000014722 | 1.848967 | 0.013332 | up | 9:-:67293309-67341341 | 9:-:67293309-67341341 | Raph1 |
| ENSRNOG00000002793 | 2.808649 | 0.013406 | up | 10:+:102136283-102143434 | 10:+:102136283-102143434 | Sstr2 |
| ENSRNOG00000061650 | 3.541815 | 0.013425 | up | 8:+:116771982-116773868 | 8:+:116771982-116773868 | AC128059.5 |
| ENSRNOG00000019496 | -1.71158852314523 | 0.013507 | down | 10:+:88764732-88789057 | 10:+:88764732-88789057 | Stat5a |
| ENSRNOG00000004411 | -1.63074581533819 | 0.013603 | down | 7:+:58814805-58847563 | 7:+:58814805-58847563 | Tspan8 |
| ENSRNOG00000012759 | 3.301458 | 0.013667 | up | 9:-:30515089-30844199 | 9:-:30515089-30844199 | Col19a1 |
| ENSRNOG00000007957 | 1.607197 | 0.013786 | up | 10:+:75055020-75076434 | 10:+:75055020-75076434 | Tspoap1 |
| ENSRNOG00000005996 | 4.766546 | 0.013793 | up | 3:-:15411939-15433252 | 3:-:15411939-15433252 | Lhx6 |
| ENSRNOG00000037198 | -2.32809756932891 | 0.013876 | down | 4:+:153805993-153834430 | 4:+:153805993-153834430 | Usp18 |
| ENSRNOG00000008765 | 1.632165 | 0.013884 | up | 2:-:98423569-98610368 | 2:-:98423569-98610368 | Zfhx4 |
| ENSRNOG00000022893 | 2.366432 | 0.0139 | up | 12:+:31530699-31620760 | 12:+:31530699-31620760 | Rimbp2 |
| ENSRNOG00000051456 | 3.4309 | 0.013924 | up | 2:+:254022079-254039326 | 2:+:254022079-254039326 | AABR07013729.1 |
| ENSRNOG00000001658 | 2.425105 | 0.013975 | up | 11:-:35024196-35099383 | 11:-:35024196-35099383 | Kcnj6 |
| ENSRNOG00000017079 | 1.212477 | 0.014001 | up | 1:+:165237847-165286980 | 1:+:165237847-165286980 | Pgm2l1 |
| ENSRNOG00000032240 | -2.29687411944938 | 0.014187 | down | 2:+:248178389-248197160 | 2:+:248178389-248197160 | Gbp5 |
| ENSRNOG00000011677 | 1.456871 | 0.014258 | up | 9:+:60021534-60070552 | 9:+:60021534-60070552 | Slc39a10 |
| ENSRNOG00000013484 | -1.54121109770465 | 0.014357 | down | 9:-:27368272-27452902 | 9:-:27368272-27452902 | Gsta1 |
| ENSRNOG00000002736 | 1.248654 | 0.014408 | up | 13:-:78011047-78609045 | 13:-:78011047-78609045 | Rabgap1l |
| ENSRNOG00000033517 | -1.70515190006274 | 0.014576 | down | 1:+:20115995-20116513 | 1:+:20115995-20116513 | LOC100360791 |
| ENSRNOG00000016897 | -1.4864542835889 | 0.014672 | down | 1:-:141097695-141111400 | 1:-:141097695-141111400 | Rlbp1 |
| ENSRNOG00000014987 | -3.40781124137774 | 0.014683 | down | 9:+:15166118-15184468 | 9:+:15166118-15184468 | Mdfi |
| ENSRNOG00000052319 | 1.925936 | 0.014708 | up | 1:+:165284410-165284635 | 1:+:165284410-165284635 | AABR07004881.1 |
| ENSRNOG00000028580 | 1.578881 | 0.014724 | up | 19:-:58583632-58735173 | 19:-:58583632-58735173 | Pcnx2 |
| ENSRNOG00000008465 | -1.16622288771334 | 0.014733 | down | 4:-:78450724-78458179 | 4:-:78450724-78458179 | Tmem176b |
| ENSRNOG00000043103 | 2.422307 | 0.014769 | up | 5:-:74000227-74029238 | 5:-:74000227-74029238 | Frrs1l |
| ENSRNOG00000010479 | 1.03489 | 0.014856 | up | 15:-:87506959-93868301 | 15:-:87506959-93868301 | Mycbp2 |
| ENSRNOG00000024899 | -2.4604656609255 | 0.014913 | down | 14:-:15253125-15258207 | 14:-:15253125-15258207 | Cxcl13 |
| ENSRNOG00000048145 | 2.571848 | 0.014962 | up | 6:+:79254339-79258611 | 6:+:79254339-79258611 | Sstr1 |
| ENSRNOG00000061230 | 1.102697 | 0.015032 | up | X:+:156909913-156928057 | X:+:156909913-156928057 | L1cam |
| ENSRNOG00000003866 | -2.32503324647334 | 0.015048 | down | 13:-:45314933-45318878 | 13:-:45314933-45318878 | Cxcr4 |
| ENSRNOG00000014082 | -3.05179399320517 | 0.015236 | down | 7:-:143958858-143967484 | 7:-:143958858-143967484 | Sp7 |
| ENSRNOG00000012881 | -1.63060637093911 | 0.015253 | down | 4:-:10323607-10329241 | 4:-:10323607-10329241 | Fgl2 |
| ENSRNOG00000049410 | 1.340803 | 0.015548 | up | 17:-:63990599-63994169 | 17:-:63990599-63994169 | Chrm3 |
| ENSRNOG00000026310 | 3.39167 | 0.015595 | up | 16:-:25001666-25192675 | 16:-:25001666-25192675 | Marchf1 |
| ENSRNOG00000038999 | -1.94659113029052 | 0.015599 | down | 20:+:5351605-5421098 | 20:+:5351605-5421098 | RT1-A1 |
| ENSRNOG00000003049 | -3.48017012525585 | 0.015606 | down | X:+:21696772-21699241 | X:+:21696772-21699241 | Hsd17b10 |
| ENSRNOG00000012053 | -1.39506694649328 | 0.015705 | down | 2:+:189922996-189928848 | 2:+:189922996-189928848 | S100a16 |
| ENSRNOG00000034174 | -3.42945327551398 | 0.01576 | down | 3:+:149424392-149449350 | 3:+:149424392-149449350 | Bpifb4 |
| ENSRNOG00000020277 | 1.293948 | 0.01581 | up | 10:+:89089646-89103614 | 10:+:89089646-89103614 | Cntnap1 |
| ENSRNOG00000039025 | -1.31696439485944 | 0.015872 | down | 3:-:29861224-29861621 | 3:-:29861224-29861621 | AABR07051947.1 |
| ENSRNOG00000016221 | 2.367252 | 0.016051 | up | 8:+:49418965-49427689 | 8:+:49418965-49427689 | Scn2b |
| ENSRNOG00000016980 | -2.2574213461101 | 0.016167 | down | 1:-:198544262-198559568 | 1:-:198544262-198559568 | Qprt |
| ENSRNOG00000003512 | 1.280886 | 0.016331 | up | 10:-:27310725-27366665 | 10:-:27310725-27366665 | Gabra1 |
| ENSRNOG00000018416 | 1.209882 | 0.01634 | up | 9:+:16862248-16902199 | 9:+:16862248-16902199 | Ttbk1 |
| ENSRNOG00000013729 | 2.678199 | 0.016342 | up | 4:-:65834035-65962539 | 4:-:65834035-65962539 | RGD1306271 |
| ENSRNOG00000031312 | -1.19432537516851 | 0.016357 | down | 4:+:157864969-157877633 | 4:+:157864969-157877633 | Tnfrsf1a |
| ENSRNOG00000055124 | 2.518408 | 0.016373 | up | 1:-:62507149-62538222 | 1:-:62507149-62538222 | AABR07001942.1 |
| ENSRNOG00000014850 | -3.02618260502767 | 0.016397 | down | 7:+:144052061-144060685 | 7:+:144052061-144060685 | Amhr2 |
| ENSRNOG00000059947 | -2.69712245699407 | 0.016427 | down | 6:+:33885495-33908016 | 6:+:33885495-33908016 | Sdc1 |
| ENSRNOG00000052236 | 2.788591 | 0.016529 | up | 1:+:173275336-173284941 | 1:+:173275336-173284941 | AABR07004992.1 |
| ENSRNOG00000014961 | -1.14410776513802 | 0.016553 | down | 5:-:161947137-161981441 | 5:-:161947137-161981441 | Pdpn |
| ENSRNOG00000051261 | 2.893351 | 0.016649 | up | 13:+:77264239-77485250 | 13:+:77264239-77485250 | AABR07021536.1 |
| ENSRNOG00000017198 | 2.80154 | 0.01667 | up | 1:-:78976318-78997869 | 1:-:78976318-78997869 | Hif3a |
| ENSRNOG00000000902 | 1.251788 | 0.016684 | up | 12:-:6322668-6341902 | 12:-:6322668-6341902 | Hsph1 |
| ENSRNOG00000051615 | -1.27448790326901 | 0.016715 | down | 5:-:152195361-152198813 | 5:-:152195361-152198813 | Hmgn2 |
| ENSRNOG00000017927 | -4.03117483732119 | 0.016776 | down | 1:+:214278296-214281483 | 1:+:214278296-214281483 | Drd4 |
| ENSRNOG00000019440 | -3.22536230901952 | 0.016785 | down | 1:+:81230612-81245996 | 1:+:81230612-81245996 | Kcnn4 |
| ENSRNOG00000025278 | 1.701742 | 0.0168 | up | 3:-:23362707-23474170 | 3:-:23362707-23474170 | Scai |
| ENSRNOG00000001368 | 2.462078 | 0.016802 | up | 12:+:41073824-41148490 | 12:+:41073824-41148490 | Rph3a |
| ENSRNOG00000033202 | 2.146611 | 0.016838 | up | 4:-:117256770-117268178 | 4:-:117256770-117268178 | Fbxo41 |
| ENSRNOG00000053541 | 2.315844 | 0.016849 | up | 7:-:140132469-140147984 | 7:-:140132469-140147984 | Kansl2 |
| ENSRNOG00000016793 | 2.588039 | 0.016855 | up | 2:-:205207799-205212681 | 2:-:205207799-205212681 | Tshb |
| ENSRNOG00000007650 | -1.2900020771865 | 0.016913 | down | 7:+:3320103-3335582 | 7:+:3320103-3335582 | Cd63 |
| ENSRNOG00000019692 | -1.30650354670532 | 0.016956 | down | 10:-:15164439-15166457 | 10:-:15164439-15166457 | Metrn |
| ENSRNOG00000002045 | -1.12197123558464 | 0.017121 | down | 14:-:14364008-14426437 | 14:-:14364008-14426437 | Anxa3 |
| ENSRNOG00000016046 | 2.207115 | 0.017156 | up | 17:+:53229785-53773657 | 17:+:53229785-53773657 | Hecw1 |
| ENSRNOG00000058180 | 2.324942 | 0.017197 | up | 7:+:380741-381732 | 7:+:380741-381732 | LOC102554748 |
| ENSRNOG00000028879 | 3.962182 | 0.017219 | up | 7:+:142397371-142464158 | 7:+:142397371-142464158 | Slc4a8 |
| ENSRNOG00000007457 | -1.94159566644518 | 0.017289 | down | 3:-:72161189-72171078 | 3:-:72161189-72171078 | Serping1 |
| ENSRNOG00000018087 | -1.0428602970898 | 0.01729 | down | 17:+:80882666-80891212 | 17:+:80882666-80891212 | Vim |
| ENSRNOG00000030719 | 2.183218 | 0.017359 | up | 16:+:78539489-78850222 | 16:+:78539489-78850222 | Csmd1 |
| ENSRNOG00000000456 | -2.80532333504889 | 0.017371 | down | 20:+:3990613-3993769 | 20:+:3990613-3993769 | Psmb8 |
| ENSRNOG00000012962 | -1.11922497579289 | 0.017418 | down | 8:-:113672758-113675128 | 8:-:113672758-113675128 | Nudt16 |
| ENSRNOG00000010832 | -2.60464459209219 | 0.017444 | down | 16:-:54386288-54450426 | 16:-:54386288-54450426 | Pdgfrl |
| ENSRNOG00000011931 | 1.315174 | 0.017487 | up | 1:+:244615821-244782706 | 1:+:244615821-244782706 | Smarca2 |
| ENSRNOG00000015505 | -2.73843308532843 | 0.017504 | down | 4:+:155313671-155336228 | 4:+:155313671-155336228 | Mfap5 |
| ENSRNOG00000011917 | -3.6937439168302 | 0.017643 | down | 10:-:94497448-94500591 | 10:-:94497448-94500591 | Cd79b |
| ENSRNOG00000059456 | 1.491558 | 0.017672 | up | 12:+:49626871-49746272 | 12:+:49626871-49746272 | Grk3 |
| ENSRNOG00000025612 | 1.884598 | 0.017676 | up | 12:+:50090464-50241995 | 12:+:50090464-50241995 | Sez6l |
| ENSRNOG00000012807 | -1.2901853734616 | 0.017715 | down | 5:-:155261250-155264143 | 5:-:155261250-155264143 | C1qa |
| ENSRNOG00000003929 | 2.015272 | 0.017974 | up | X:-:104391607-104493757 | X:-:104391607-104493757 | Pcdh19 |
| ENSRNOG00000033615 | -1.61684639175406 | 0.018049 | down | MT:+:9451-9798 | MT:+:9451-9798 | Mt-nd3 |
| ENSRNOG00000050404 | -1.09145398887615 | 0.0181 | down | 3:-:171294856-171342646 | 3:-:171294856-171342646 | Pmepa1 |
| ENSRNOG00000003680 | 2.473198 | 0.018166 | up | 10:+:27973681-28187565 | 10:+:27973681-28187565 | Gabrb2 |
| ENSRNOG00000020021 | 2.103239 | 0.018209 | up | 1:+:214927172-214947624 | 1:+:214927172-214947624 | Brsk2 |
| ENSRNOG00000026136 | -1.28786349659836 | 0.018228 | down | 18:+:44716226-44779914 | 18:+:44716226-44779914 | Tnfaip8 |
| ENSRNOG00000003066 | 3.172827 | 0.018251 | up | 10:+:45659143-45681393 | 10:+:45659143-45681393 | Wnt9a |
| ENSRNOG00000023760 | 1.492588 | 0.018457 | up | 9:-:71498293-71651512 | 9:-:71498293-71651512 | Plekhm3 |
| ENSRNOG00000046424 | 1.806187 | 0.018471 | up | 5:-:164684509-164714145 | 5:-:164684509-164714145 | Mfn2 |
| ENSRNOG00000013072 | 4.31425 | 0.018515 | up | 4:-:59445767-59809321 | 4:-:59445767-59809321 | Plxna4 |
| ENSRNOG00000037627 | -1.20674025161021 | 0.018516 | down | 10:+:55924938-55926783 | 10:+:55924938-55926783 | Trappc1 |
| ENSRNOG00000006557 | 1.335717 | 0.018581 | up | 10:-:31278746-31419235 | 10:-:31278746-31419235 | Cyfip2 |
| ENSRNOG00000019615 | -2.75027589906637 | 0.018589 | down | 16:-:7627603-7681576 | 16:-:7627603-7681576 | Colq |
| ENSRNOG00000003809 | -1.13887625449271 | 0.0186 | down | X:+:43625169-43629765 | X:+:43625169-43629765 | Sat1 |
| ENSRNOG00000046834 | -1.68260719526872 | 0.018634 | down | 9:+:9721105-9747167 | 9:+:9721105-9747167 | C3 |
| ENSRNOG00000033942 | 4.521755 | 0.018674 | up | 10:+:5930298-6119990 | 10:+:5930298-6119990 | Grin2a |
| ENSRNOG00000012749 | -1.29847950599266 | 0.018674 | down | 5:-:155246447-155252003 | 5:-:155246447-155252003 | C1qb |
| ENSRNOG00000020307 | 2.503736 | 0.01869 | up | 2:+:211320420-211332327 | 2:+:211320420-211332327 | RGD1309139 |
| ENSRNOG00000030330 | 3.607823 | 0.018715 | up | X:+:92596378-92691573 | X:+:92596378-92691573 | AABR07040095.1 |
| ENSRNOG00000017803 | -1.26707647627235 | 0.018774 | down | 17:-:90099025-90149894 | 17:-:90099025-90149894 | Apbb1ip |
| ENSRNOG00000020843 | -1.11256285299405 | 0.018972 | down | 1:-:101448346-101449829 | 1:-:101448346-101449829 | LOC100360087 |
| ENSRNOG00000011475 | 1.989673 | 0.018982 | up | 10:-:85460059-85517683 | 10:-:85460059-85517683 | Srcin1 |
| ENSRNOG00000017108 | 1.704388 | 0.018998 | up | 7:+:121311024-121334437 | 7:+:121311024-121334437 | Syngr1 |
| ENSRNOG00000017123 | -1.01277527034021 | 0.018999 | down | 3:+:114087287-114093309 | 3:+:114087287-114093309 | B2m |
| ENSRNOG00000051521 | -1.9455466743378 | 0.019058 | down | 8:+:13870770-13870891 | 8:+:13870770-13870891 | AC105648.2 |
| ENSRNOG00000007271 | 1.133708 | 0.019068 | up | 6:-:105459706-105518748 | 6:-:105459706-105518748 | Map3k9 |
| ENSRNOG00000021524 | -5.21080282289673 | 0.019142 | down | 11:+:30904733-30915225 | 11:+:30904733-30915225 | Mrap |
| ENSRNOG00000024990 | -3.63342398023949 | 0.019215 | down | 3:+:66673071-66780075 | 3:+:66673071-66780075 | Ppp1r1c |
| ENSRNOG00000005550 | 1.121091 | 0.019224 | up | 6:+:83083740-83438601 | 6:+:83083740-83438601 | Lrfn5 |
| ENSRNOG00000050898 | -3.93816249912881 | 0.019238 | down | 11:+:85532526-85533034 | 11:+:85532526-85533034 | AABR07034730.2 |
| ENSRNOG00000046990 | 1.781761 | 0.019285 | up | 3:-:152150152-152159749 | 3:-:152150152-152159749 | Rbm12 |
| ENSRNOG00000052695 | -2.65110637336072 | 0.01929 | down | X:-:28686163-28698844 | X:-:28686163-28698844 | LOC102549869 |
| ENSRNOG00000010423 | -2.9480569949051 | 0.019323 | down | 7:-:12697743-12707922 | 7:-:12697743-12707922 | Misp |
| ENSRNOG00000001427 | 1.04502 | 0.019338 | up | 12:+:23544287-23564569 | 12:+:23544287-23564569 | Orai2 |
| ENSRNOG00000013085 | 2.954546 | 0.019352 | up | 5:-:34174411-34813116 | 5:-:34174411-34813116 | Nkain3 |
| ENSRNOG00000007104 | 1.834249 | 0.019363 | up | 4:+:140247313-140580748 | 4:+:140247313-140580748 | Itpr1 |
| ENSRNOG00000001825 | 3.023821 | 0.019432 | up | 11:-:88912599-88972176 | 11:-:88912599-88972176 | AABR07034767.1 |
| ENSRNOG00000001628 | -1.81997393932278 | 0.01947 | down | 11:+:36851038-36912229 | 11:+:36851038-36912229 | Pcp4 |
| ENSRNOG00000060054 | 2.120403 | 0.019492 | up | 10:-:63928064-63952726 | 10:-:63928064-63952726 | Doc2b |
| ENSRNOG00000054344 | -1.7518343877009 | 0.019516 | down | 19:-:25774143-25775659 | 19:-:25774143-25775659 | Ier2 |
| ENSRNOG00000002322 | -1.25769793498103 | 0.019676 | down | 13:-:102780877-102790639 | 13:-:102780877-102790639 | RGD1310587 |
| ENSRNOG00000015160 | -1.69471850828298 | 0.019709 | down | 5:+:25349928-25353661 | 5:+:25349928-25353661 | Gem |
| ENSRNOG00000013213 | 1.257043 | 0.019731 | up | 9:-:83111222-83253458 | 9:-:83111222-83253458 | Epha4 |
| ENSRNOG00000009872 | -1.07865297071819 | 0.01977 | down | 4:+:7355574-7387253 | 4:+:7355574-7387253 | Kcnh2 |
| ENSRNOG00000025558 | 3.732635 | 0.019821 | up | 5:+:74649765-74788497 | 5:+:74649765-74788497 | Palm2 |
| ENSRNOG00000002976 | 1.865321 | 0.019845 | up | 11:+:64975594-65018108 | 11:+:64975594-65018108 | Maats1 |
| ENSRNOG00000052572 | 1.418018 | 0.019846 | up | X:-:153539668-154051181 | X:-:153539668-154051181 | Aff2 |
| ENSRNOG00000002266 | -3.7640231353904 | 0.019956 | down | 14:-:35650985-35652709 | 14:-:35650985-35652709 | Gsx2 |
| ENSRNOG00000042499 | -1.63917924965597 | 0.020037 | down | 4:-:100882216-100883275 | 4:-:100882216-100883275 | LOC100364435 |
| ENSRNOG00000024494 | 2.933014 | 0.020054 | up | 7:-:33702770-33793565 | 7:-:33702770-33793565 | AABR07056633.1 |
| ENSRNOG00000026787 | 1.364922 | 0.020064 | up | 3:+:110574417-110589921 | 3:+:110574417-110589921 | Disp2 |
| ENSRNOG00000013000 | -1.11151518195242 | 0.020139 | down | 4:-:176701983-176720012 | 4:-:176701983-176720012 | Ldhb |
| ENSRNOG00000009325 | -1.22177246238397 | 0.020166 | down | 5:+:154269118-154286544 | 5:+:154269118-154286544 | Fuca1 |
| ENSRNOG00000028630 | 2.291675 | 0.020287 | up | 12:-:44536293-44911147 | 12:-:44536293-44911147 | Ksr2 |
| ENSRNOG00000002979 | 2.758085 | 0.020425 | up | 13:-:83403264-83425641 | 13:-:83403264-83425641 | Tbx19 |
| ENSRNOG00000004201 | 2.283765 | 0.020598 | up | 7:+:78092037-78594138 | 7:+:78092037-78594138 | Rims2 |
| ENSRNOG00000007539 | -1.74679848843746 | 0.020776 | down | 6:-:45655954-45669148 | 6:-:45655954-45669148 | Rsad2 |
| ENSRNOG00000012061 | 1.206441 | 0.021013 | up | 1:+:192233910-192574831 | 1:+:192233910-192574831 | Prkcb |
| ENSRNOG00000032664 | 2.920159 | 0.021029 | up | 20:+:3162039-3162939 | 20:+:3162039-3162939 | AABR07044362.2 |
| ENSRNOG00000046791 | 4.182559 | 0.021097 | up | 20:+:28572242-28717243 | 20:+:28572242-28717243 | Sh3rf3 |
| ENSRNOG00000028077 | 4.511982 | 0.021304 | up | 11:-:61162628-61234944 | 11:-:61162628-61234944 | Cfap44 |
| ENSRNOG00000019854 | -2.43009569062127 | 0.021326 | down | 1:+:100577056-100589836 | 1:+:100577056-100589836 | Napsa |
| ENSRNOG00000027374 | 2.971441 | 0.021365 | up | 1:-:211983729-212022212 | 1:-:211983729-212022212 | AABR07005985.1 |
| ENSRNOG00000047003 | 2.408658 | 0.021463 | up | 9:+:12420368-12421059 | 9:+:12420368-12421059 | AABR07066693.1 |
| ENSRNOG00000046254 | -1.15342674005113 | 0.021477 | down | 9:-:9431860-9585865 | 9:-:9431860-9585865 | Adgre1 |
| ENSRNOG00000005410 | -1.99556665201993 | 0.021494 | down | 6:+:145740035-145785606 | 6:+:145740035-145785606 | Cdca7l |
| ENSRNOG00000012804 | -1.20891248015464 | 0.021528 | down | 5:-:155255005-155258392 | 5:-:155255005-155258392 | C1qc |
| ENSRNOG00000003732 | 2.142551 | 0.021553 | up | 6:+:119519714-119609296 | 6:+:119519714-119609296 | Flrt2 |
| ENSRNOG00000018346 | -2.26676437309857 | 0.021648 | down | 17:-:35907108-35958077 | 17:-:35907108-35958077 | Agtr1a |
| ENSRNOG00000061508 | 3.865259 | 0.021682 | up | X:+:33884499-34057399 | X:+:33884499-34057399 | Reps2 |
| ENSRNOG00000052022 | 1.382847 | 0.021692 | up | X:+:153064028-153067500 | X:+:153064028-153067500 | Pnma3 |
| ENSRNOG00000001449 | 1.068764 | 0.021697 | up | 12:+:24324590-24341938 | 12:+:24324590-24341938 | Pom121 |
| ENSRNOG00000020182 | -1.0674101495007 | 0.021766 | down | 1:-:198420806-198450047 | 1:-:198420806-198450047 | Mvp |
| ENSRNOG00000013663 | -1.24448080358447 | 0.021833 | down | 1:+:103172987-103177417 | 1:+:103172987-103177417 | Tmem86a |
| ENSRNOG00000021962 | -1.06257144734177 | 0.021891 | down | 10:+:90550147-90552057 | 10:+:90550147-90552057 | Fzd2 |
| ENSRNOG00000055843 | 2.453491 | 0.021932 | up | 1:-:63286802-63293635 | 1:-:63286802-63293635 | Zik1 |
| ENSRNOG00000013014 | -1.77154567174968 | 0.021944 | down | 19:-:55249616-55257876 | 19:-:55249616-55257876 | Cyba |
| ENSRNOG00000048130 | 3.382491 | 0.021956 | up | 8:+:118392773-118414603 | 8:+:118392773-118414603 | LOC100911725 |
| ENSRNOG00000042519 | 1.773984 | 0.021987 | up | 8:-:60867819-61079526 | 8:-:60867819-61079526 | Peak1 |
| ENSRNOG00000018317 | 1.044559 | 0.022021 | up | 4:+:118655728-118795774 | 4:+:118655728-118795774 | Aak1 |
| ENSRNOG00000054955 | 1.999967 | 0.022048 | up | 1:+:212181374-212211578 | 1:+:212181374-212211578 | Adgra1 |
| ENSRNOG00000004269 | 1.980061 | 0.02206 | up | 6:+:48452369-48857936 | 6:+:48452369-48857936 | Myt1l |
| ENSRNOG00000003749 | 1.185148 | 0.022083 | up | X:+:14498119-14534473 | X:+:14498119-14534473 | Xk |
| ENSRNOG00000034177 | 3.355862 | 0.02222 | up | 9:-:110057155-110225486 | 9:-:110057155-110225486 | Efna5 |
| ENSRNOG00000012325 | -1.07856868608648 | 0.022295 | down | 15:-:3033495-3435888 | 15:-:3033495-3435888 | Adk |
| ENSRNOG00000012858 | 3.206481 | 0.022305 | up | 1:+:213597478-213600685 | 1:+:213597478-213600685 | Odf3 |
| ENSRNOG00000049484 | 1.246821 | 0.022491 | up | 3:-:165412803-165477771 | 3:-:165412803-165477771 | Atp9a |
| ENSRNOG00000049281 | 1.250379 | 0.022507 | up | 2:+:188516582-188522601 | 2:+:188516582-188522601 | Gba |
| ENSRNOG00000012568 | 1.101642 | 0.022521 | up | 3:-:79960301-80003032 | 3:-:79960301-80003032 | Madd |
| ENSRNOG00000001647 | 1.514122 | 0.022525 | up | 11:+:36075709-36092495 | 11:+:36075709-36092495 | Ets2 |
| ENSRNOG00000002496 | 2.059469 | 0.02258 | up | 11:+:66316606-66566331 | 11:+:66316606-66566331 | Stxbp5l |
| ENSRNOG00000051792 | -2.05923364845033 | 0.022634 | down | 8:-:55164736-55171718 | 8:-:55164736-55171718 | LOC689959 |
| ENSRNOG00000002475 | -2.08272176554041 | 0.022635 | down | 7:-:138895079-138895597 | 7:-:138895079-138895597 | AABR07058831.1 |
| ENSRNOG00000055076 | 2.596708 | 0.022656 | up | 16:-:26080258-26098379 | 16:-:26080258-26098379 | AABR07025023.1 |
| ENSRNOG00000048169 | -2.00334630797508 | 0.022671 | down | 4:+:153774486-153791328 | 4:+:153774486-153791328 | Tuba8 |
| ENSRNOG00000011977 | 1.235586 | 0.022732 | up | 2:+:85377318-85808970 | 2:+:85377318-85808970 | Sema5a |
| ENSRNOG00000025634 | 1.675235 | 0.022746 | up | 15:-:33720701-33752665 | 15:-:33720701-33752665 | Zfhx2 |
| ENSRNOG00000017429 | -2.781949502738 | 0.022782 | down | 1:-:197765644-197770669 | 1:-:197765644-197770669 | Lat |
| ENSRNOG00000024705 | -1.68762581478313 | 0.022786 | down | 4:-:78205812-78208767 | 4:-:78205812-78208767 | Rarres2 |
| ENSRNOG00000009253 | 3.877401 | 0.022805 | up | 8:+:28352772-28387383 | 8:+:28352772-28387383 | Igsf9b |
| ENSRNOG00000012460 | -1.58653110464269 | 0.022837 | down | 1:-:229599009-229601032 | 1:-:229599009-229601032 | Cntf |
| ENSRNOG00000007350 | -1.64570210694431 | 0.022839 | down | 7:-:119783849-119797098 | 7:-:119783849-119797098 | Rac2 |
| ENSRNOG00000028082 | 2.789513 | 0.022899 | up | 5:+:70592871-70599428 | 5:+:70592871-70599428 | Tal2 |
| ENSRNOG00000001499 | -4.05699699868622 | 0.022959 | down | 1:-:83991578-83993270 | 1:-:83991578-83993270 | Mia |
| ENSRNOG00000005299 | 1.239526 | 0.023052 | up | 7:-:70513343-70556827 | 7:-:70513343-70556827 | Kif5a |
| ENSRNOG00000013190 | -1.32824066812847 | 0.023094 | down | 1:+:53174879-53192048 | 1:+:53174879-53192048 | Rnaset2 |
| ENSRNOG00000006096 | -2.43566865600128 | 0.023171 | down | 5:-:27986663-28177340 | 5:-:27986663-28177340 | Slc26a7 |
| ENSRNOG00000009180 | 3.117799 | 0.023222 | up | 3:+:148510779-148533404 | 3:+:148510779-148533404 | Xkr7 |
| ENSRNOG00000054339 | -2.45137466454594 | 0.023276 | down | X:-:156438764-156438899 | X:-:156438764-156438899 | SNORA70 |
| ENSRNOG00000009170 | 1.245267 | 0.023296 | up | 8:-:58932580-59077690 | 8:-:58932580-59077690 | Dmxl2 |
| ENSRNOG00000025198 | -2.63024649871428 | 0.023436 | down | 7:-:29959597-29986163 | 7:-:29959597-29986163 | Gas2l3 |
| ENSRNOG00000007645 | 1.333785 | 0.023526 | up | 13:-:90703036-90710287 | 13:-:90703036-90710287 | Kcnj9 |
| ENSRNOG00000027233 | 3.671362 | 0.023539 | up | X:-:115627653-115908693 | X:-:115627653-115908693 | Trpc5 |
| ENSRNOG00000000443 | -1.31418089240926 | 0.023599 | down | 20:-:4302347-4508214 | 20:-:4302347-4508214 | LOC103689965 |
| ENSRNOG00000038047 | -3.16094859985042 | 0.023654 | down | 17:+:78793336-78793724 | 17:+:78793336-78793724 | Mt1 |
| ENSRNOG00000059479 | 5.172045 | 0.023712 | up | 14:+:87312203-87421659 | 14:+:87312203-87421659 | Adcy1 |
| ENSRNOG00000016180 | 1.234801 | 0.023768 | up | 5:-:25577451-25584288 | 5:-:25577451-25584288 | Pdp1 |
| ENSRNOG00000058337 | -2.29715638856129 | 0.023783 | down | 3:-:110498114-110517163 | 3:-:110498114-110517163 | Plcb2 |
| ENSRNOG00000000991 | -1.1289282551858 | 0.023817 | down | 12:-:11252296-11265865 | 12:-:11252296-11265865 | Arpc1b |
| ENSRNOG00000023688 | 3.820232 | 0.023826 | up | 17:+:11101306-11103541 | 17:+:11101306-11103541 | Drd1 |
| ENSRNOG00000000816 | -1.16295584259356 | 0.023843 | down | 20:-:3372413-3397039 | 20:-:3372413-3397039 | Ppp1r18 |
| ENSRNOG00000000843 | -4.12993592679858 | 0.023846 | down | 20:+:5057701-5059933 | 20:+:5057701-5059933 | Ly6g6c |
| ENSRNOG00000013364 | -1.33751206766115 | 0.023868 | down | 1:+:29191192-29201531 | 1:+:29191192-29201531 | Hey2 |
| ENSRNOG00000034089 | 3.178103 | 0.023932 | up | 8:+:128622428-128657955 | 8:+:128622428-128657955 | Ttc21a |
| ENSRNOG00000004496 | 1.113236 | 0.023936 | up | 6:+:42180894-42289908 | 6:+:42180894-42289908 | Rock2 |
| ENSRNOG00000002844 | 1.300164 | 0.023938 | up | X:+:15988604-15990484 | X:+:15988604-15990484 | Usp27x |
| ENSRNOG00000031743 | -1.46595136587597 | 0.024021 | down | 2:+:248276709-248293784 | 2:+:248276709-248293784 | Gbp2 |
| ENSRNOG00000049498 | -2.32189740419178 | 0.024169 | down | 16:-:23961067-23991570 | 16:-:23961067-23991570 | Nat2 |
| ENSRNOG00000018666 | -1.97024156079164 | 0.024267 | down | 3:+:3767394-3794359 | 3:+:3767394-3794359 | Gpsm1 |
| ENSRNOG00000024657 | 4.295769 | 0.02428 | up | 13:-:48807720-48848997 | 13:-:48807720-48848997 | Mfsd4a |
| ENSRNOG00000059113 | 3.657968 | 0.02429 | up | 15:-:87404841-87450319 | 15:-:87404841-87450319 | AABR07019085.1 |
| ENSRNOG00000012290 | -3.14351032861928 | 0.02433 | down | 3:+:110975923-110979957 | 3:+:110975923-110979957 | Gchfr |
| ENSRNOG00000019387 | -1.31818189571938 | 0.024369 | down | 16:+:20426566-20430752 | 16:+:20426566-20430752 | Ifi30 |
| ENSRNOG00000001431 | -1.75852498246301 | 0.024386 | down | 12:-:23596981-23624316 | 12:-:23596981-23624316 | Rasa4 |
| ENSRNOG00000031306 | 2.690884 | 0.024528 | up | 4:+:24222500-24232017 | 4:+:24222500-24232017 | Zfp804b |
| ENSRNOG00000014055 | -1.99486738551647 | 0.024535 | down | 16:-:23970743-23991573 | 16:-:23970743-23991573 | Nat1 |
| ENSRNOG00000011044 | 1.959393 | 0.024578 | up | 6:-:128473570-128567198 | 6:-:128473570-128567198 | Clmn |
| ENSRNOG00000012960 | -1.31889599649713 | 0.024594 | down | 3:-:2579258-2584523 | 3:-:2579258-2584523 | Uap1l1 |
| ENSRNOG00000011508 | 1.840963 | 0.024623 | up | X:+:65040104-65074712 | X:+:65040104-65074712 | Zc3h12b |
| ENSRNOG00000021079 | -1.42941555019497 | 0.02463 | down | 1:-:89484199-89488223 | 1:-:89484199-89488223 | Fxyd1 |
| ENSRNOG00000004214 | -1.36285277292205 | 0.024778 | down | 10:+:55492404-55496012 | 10:+:55492404-55496012 | Rpl26 |
| ENSRNOG00000019244 | -1.24415432285815 | 0.024886 | down | 5:+:173288447-173292929 | 5:+:173288447-173292929 | Mxra8 |
| ENSRNOG00000002863 | 2.775299 | 0.024917 | up | 13:-:71906702-72367980 | 13:-:71906702-72367980 | Cacna1e |
| ENSRNOG00000038427 | 3.110725 | 0.024939 | up | 14:-:78359682-78377825 | 14:-:78359682-78377825 | AABR07015812.1 |
| ENSRNOG00000021839 | 1.237821 | 0.024946 | up | 8:-:6255850-6305033 | 8:-:6255850-6305033 | Cep126 |
| ENSRNOG00000011913 | -1.26118096527891 | 0.024998 | down | 2:+:104744461-104799853 | 2:+:104744461-104799853 | Cp |
| ENSRNOG00000031126 | -1.91800683109234 | 0.02501 | down | 8:-:46603728-46675544 | 8:-:46603728-46675544 | Tecta |
| ENSRNOG00000011815 | 1.610615 | 0.025031 | up | 1:-:24185435-24302298 | 1:-:24185435-24302298 | Sgk1 |
| ENSRNOG00000052296 | 1.710133 | 0.025151 | up | 7:+:130474279-130534679 | 7:+:130474279-130534679 | Shank3 |
| ENSRNOG00000047714 | -2.45126702267391 | 0.025159 | down | 13:-:36094520-36101411 | 13:-:36094520-36101411 | Tmem37 |
| ENSRNOG00000011824 | -3.79143022233571 | 0.025178 | down | 4:-:124110716-124113242 | 4:-:124110716-124113242 | Trh |
| ENSRNOG00000030021 | -2.49135676170443 | 0.025184 | down | 10:-:70797124-70802782 | 10:-:70797124-70802782 | Ccl6 |
| ENSRNOG00000027264 | 1.236131 | 0.025257 | up | 1:-:226297016-226353611 | 1:-:226297016-226353611 | Dagla |
| ENSRNOG00000033984 | 1.887968 | 0.025259 | up | 5:+:154037202-154058880 | 5:+:154037202-154058880 | Ifnlr1 |
| ENSRNOG00000014046 | 1.53072 | 0.025268 | up | 2:-:144303703-144323254 | 2:-:144303703-144323254 | Sertm1 |
| ENSRNOG00000038406 | 2.263991 | 0.025302 | up | 12:-:35970821-36398246 | 12:-:35970821-36398246 | Tmem132b |
| ENSRNOG00000058140 | -1.88485315215421 | 0.025325 | down | 16:+:3113681-3113781 | 16:+:3113681-3113781 | U6 |
| ENSRNOG00000018693 | -2.56454734940499 | 0.025332 | down | 10:+:56662242-56666086 | 10:+:56662242-56666086 | Asgr1 |
| ENSRNOG00000017539 | 3.477356 | 0.025363 | up | 3:+:161413298-161421520 | 3:+:161413298-161421520 | Mmp9 |
| ENSRNOG00000008901 | 3.470072 | 0.025378 | up | 5:-:62156839-62187930 | 5:-:62156839-62187930 | Coro2a |
| ENSRNOG00000021943 | 3.493225 | 0.025381 | up | 15:-:60277100-60289763 | 15:-:60277100-60289763 | Fam216b |
| ENSRNOG00000004252 | -1.32944500235551 | 0.02553 | down | 6:+:10151729-10236695 | 6:+:10151729-10236695 | LOC108348062 |
| ENSRNOG00000020322 | 1.193183 | 0.025581 | up | 9:+:98668231-98684900 | 9:+:98668231-98684900 | Asb1 |
| ENSRNOG00000003296 | 1.807781 | 0.025704 | up | 14:-:20935369-20953095 | 14:-:20935369-20953095 | Dck |
| ENSRNOG00000014509 | 1.334645 | 0.025719 | up | 15:+:41448064-41530398 | 15:+:41448064-41530398 | Sacs |
| ENSRNOG00000019374 | -1.04411590939181 | 0.025775 | down | 8:-:62405715-62424303 | 8:-:62405715-62424303 | Csk |
| ENSRNOG00000009922 | 2.719445 | 0.025792 | up | 1:-:281754724-281756159 | 1:-:281754724-281756159 | Prlhr |
| ENSRNOG00000028097 | 4.02463 | 0.025845 | up | 2:+:115678344-115788687 | 2:+:115678344-115788687 | Slc7a14 |
| ENSRNOG00000019885 | 1.145733 | 0.025858 | up | 2:-:206499463-206699105 | 2:-:206499463-206699105 | Magi3 |
| ENSRNOG00000001492 | 2.251582 | 0.025895 | up | 1:+:78025995-78060111 | 1:+:78025995-78060111 | Slc8a2 |
| ENSRNOG00000023226 | -1.59478579246962 | 0.025915 | down | 2:+:193892589-193901236 | 2:+:193892589-193901236 | S100a10 |
| ENSRNOG00000007057 | 1.505808 | 0.025916 | up | 5:+:146383942-146739418 | 5:+:146383942-146739418 | Csmd2 |
| ENSRNOG00000012062 | -1.14515852048075 | 0.026151 | down | 6:-:108467410-108488330 | 6:-:108467410-108488330 | Npc2 |
| ENSRNOG00000003949 | 2.620526 | 0.026164 | up | X:-:36857650-36884748 | X:-:36857650-36884748 | AABR07037995.1 |
| ENSRNOG00000017063 | -2.96608886677909 | 0.026176 | down | 3:-:2849984-2853272 | 3:-:2849984-2853272 | Fcna |
| ENSRNOG00000057044 | 1.201021 | 0.026178 | up | 3:-:12034413-12155098 | 3:-:12034413-12155098 | Garnl3 |
| ENSRNOG00000037331 | -1.88197330942826 | 0.02619 | down | 1:+:98398660-98402968 | 1:+:98398660-98402968 | Cd33 |
| ENSRNOG00000003553 | -2.06076474399955 | 0.026212 | down | 14:+:113202419-113295014 | 14:+:113202419-113295014 | Efemp1 |
| ENSRNOG00000010746 | -1.42668611855787 | 0.026221 | down | 4:-:147715473-147719072 | 4:-:147715473-147719072 | Rpl32 |
| ENSRNOG00000004917 | 1.112751 | 0.026241 | up | 13:-:74771522-75059326 | 13:-:74771522-75059326 | Rasal2 |
| ENSRNOG00000011268 | 1.702307 | 0.026261 | up | 5:+:169519212-169567107 | 5:+:169519212-169567107 | Chd5 |
| ENSRNOG00000011394 | 1.027327 | 0.026288 | up | 6:+:27768943-27815611 | 6:+:27768943-27815611 | Kif3c |
| ENSRNOG00000016398 | 1.074354 | 0.026346 | up | 5:+:166533181-166601684 | 5:+:166533181-166601684 | Clstn1 |
| ENSRNOG00000005809 | -1.18982749625373 | 0.026359 | down | 4:-:170913923-170932618 | 4:-:170913923-170932618 | Arhgdib |
| ENSRNOG00000000635 | 3.765017 | 0.026359 | up | 20:+:21564975-21880715 | 20:+:21564975-21880715 | Arid5b |
| ENSRNOG00000054212 | 2.147889 | 0.026444 | up | 3:-:66803247-66885094 | 3:-:66803247-66885094 | Pde1a |
| ENSRNOG00000018927 | 2.235803 | 0.026533 | up | 1:-:212622094-212622537 | 1:-:212622094-212622537 | Sprn |
| ENSRNOG00000037165 | 2.574723 | 0.026632 | up | 5:-:153807955-153840178 | 5:-:153807955-153840178 | Nipal3 |
| ENSRNOG00000013389 | 1.943753 | 0.026697 | up | 2:-:35077504-35104963 | 2:-:35077504-35104963 | Rgs7bp |
| ENSRNOG00000001516 | 1.878337 | 0.026729 | up | 3:+:58632476-58924038 | 3:+:58632476-58924038 | Rapgef4 |
| ENSRNOG00000005639 | 3.355073 | 0.026773 | up | X:+:67656253-67829026 | X:+:67656253-67829026 | Ar |
| ENSRNOG00000013312 | 2.207418 | 0.026834 | up | 13:+:57131395-57521836 | 13:+:57131395-57521836 | Kcnt2 |
| ENSRNOG00000011971 | -1.4957276533227 | 0.026911 | down | 4:-:157143592-157155609 | 4:-:157143592-157155609 | C1s |
| ENSRNOG00000006740 | -1.64597779056164 | 0.026926 | down | 14:+:84447885-84452367 | 14:+:84447885-84452367 | Castor1 |
| ENSRNOG00000008053 | 1.64563 | 0.026926 | up | 15:-:40000322-40428800 | 15:-:40000322-40428800 | Atp8a2 |
| ENSRNOG00000059538 | -1.21217287682805 | 0.026983 | down | 4:+:162934195-162943981 | 4:+:162934195-162943981 | Clec2g |
| ENSRNOG00000017188 | -1.82414969291212 | 0.026988 | down | 9:+:81968332-81998169 | 9:+:81968332-81998169 | Cyp27a1 |
| ENSRNOG00000049768 | 1.892167 | 0.027033 | up | 10:+:11392625-11512600 | 10:+:11392625-11512600 | Adcy9 |
| ENSRNOG00000005920 | -2.14361506918226 | 0.027045 | down | 3:+:145831012-145831765 | 3:+:145831012-145831765 | AABR07054266.1 |
| ENSRNOG00000005214 | -1.55757875905038 | 0.027056 | down | 14:-:100151210-100217913 | 14:-:100151210-100217913 | Plek |
| ENSRNOG00000010747 | -1.21340829990888 | 0.02707 | down | 2:+:84275884-84328998 | 2:+:84275884-84328998 | Dap |
| ENSRNOG00000000441 | -2.16888436548484 | 0.027112 | down | 20:+:4355175-4357107 | 20:+:4355175-4357107 | Gpsm3 |
| ENSRNOG00000042889 | -3.37327711689439 | 0.027123 | down | 9:+:94178221-94181010 | 9:+:94178221-94181010 | Alpg |
| ENSRNOG00000007663 | -1.80758839528282 | 0.027135 | down | 5:-:105209846-105212173 | 5:-:105209846-105212173 | LOC100911372 |
| ENSRNOG00000005811 | 1.069675 | 0.027145 | up | 6:-:34553367-34555306 | 6:-:34553367-34555306 | LOC688655 |
| ENSRNOG00000015447 | 2.233112 | 0.027147 | up | 7:-:145435584-145450301 | 7:-:145435584-145450301 | Calcoco1 |
| ENSRNOG00000062272 | -1.67277080835718 | 0.027155 | down | 17:+:15656555-15656954 | 17:+:15656555-15656954 | LOC100361933 |
| ENSRNOG00000038607 | -1.96088325204154 | 0.027166 | down | 1:+:72784966-72787367 | 1:+:72784966-72787367 | Tmem86b |
| ENSRNOG00000024159 | -1.31667157127899 | 0.027167 | down | 13:-:89601896-89606326 | 13:-:89601896-89606326 | Fcer1g |
| ENSRNOG00000000394 | -1.19708538227635 | 0.027264 | down | 20:-:32120320-32139789 | 20:-:32120320-32139789 | Srgn |
| ENSRNOG00000027271 | 2.929619 | 0.027273 | up | 10:+:95642640-95657977 | 10:+:95642640-95657977 | RGD1359290 |
| ENSRNOG00000020674 | -1.50043415762352 | 0.027302 | down | 10:+:89352835-89356547 | 10:+:89352835-89356547 | Rpl27 |
| ENSRNOG00000014373 | 3.513349 | 0.027347 | up | 1:-:174066911-174119815 | 1:-:174066911-174119815 | Trim66 |
| ENSRNOG00000018378 | 1.78357 | 0.027354 | up | 17:+:81798756-82017682 | 17:+:81798756-82017682 | Cacnb2 |
| ENSRNOG00000031700 | 1.396885 | 0.027458 | up | 1:+:15412603-15613746 | 1:+:15412603-15613746 | Map3k5 |
| ENSRNOG00000026616 | -1.92104239483939 | 0.027504 | down | 4:+:67378188-67385266 | 4:+:67378188-67385266 | Ndufb2 |
| ENSRNOG00000011171 | 2.359944 | 0.027536 | up | 5:+:139783951-139819016 | 5:+:139783951-139819016 | Rims3 |
| ENSRNOG00000060105 | 1.209191 | 0.027647 | up | 5:-:81179605-82168427 | 5:-:81179605-82168427 | Astn2 |
| ENSRNOG00000028733 | 1.058184 | 0.027662 | up | 12:+:17614632-17712373 | 12:+:17614632-17712373 | Prkar1b |
| ENSRNOG00000047836 | -5.01840312833497 | 0.02767 | down | 3:-:19320304-19320915 | 3:-:19320304-19320915 | RGD1565617 |
| ENSRNOG00000023603 | 1.717383 | 0.027702 | up | 9:+:41096835-41099189 | 9:+:41096835-41099189 | Amer3 |
| ENSRNOG00000042235 | 2.464375 | 0.027713 | up | 5:+:172881813-172884599 | 5:+:172881813-172884599 | AC130035.1 |
| ENSRNOG00000048553 | -2.46931578388121 | 0.027823 | down | 5:-:147819618-147823232 | 5:-:147819618-147823232 | Iqcc |
| ENSRNOG00000029071 | 1.206098 | 0.02784 | up | 2:+:247248407-247397483 | 2:+:247248407-247397483 | Unc5c |
| ENSRNOG00000056290 | -5.22564577962159 | 0.027862 | down | 4:-:103257811-103258134 | 4:-:103257811-103258134 | AABR07061052.1 |
| ENSRNOG00000014264 | 1.563044 | 0.027892 | up | 17:+:23661429-23761079 | 17:+:23661429-23761079 | AABR07027306.1 |
| ENSRNOG00000004077 | 1.221126 | 0.0279 | up | 7:+:54980120-55159351 | 7:+:54980120-55159351 | Kcnc2 |
| ENSRNOG00000057545 | 2.140462 | 0.028019 | up | 3:-:8432047-8432593 | 3:-:8432047-8432593 | AC114363.1 |
| ENSRNOG00000028121 | -5.36859666878174 | 0.028026 | down | 10:+:103874383-103883953 | 10:+:103874383-103883953 | Otop2 |
| ENSRNOG00000060045 | 1.089456 | 0.028059 | up | 11:+:87858453-87973422 | 11:+:87858453-87973422 | Pi4ka |
| ENSRNOG00000008709 | 1.467642 | 0.028079 | up | 8:+:33239139-33392305 | 8:+:33239139-33392305 | Arhgap32 |
| ENSRNOG00000011334 | 1.157829 | 0.028105 | up | 6:+:111049559-111120799 | 6:+:111049559-111120799 | Tmem63c |
| ENSRNOG00000014684 | -1.26328729503405 | 0.028177 | down | 2:-:189840403-189856090 | 2:-:189840403-189856090 | Npr1 |
| ENSRNOG00000027960 | 3.094999 | 0.028194 | up | 6:-:107572233-107578467 | 6:-:107572233-107578467 | AC094055.1 |
| ENSRNOG00000004487 | 2.576246 | 0.028214 | up | 13:+:110511668-110522769 | 13:+:110511668-110522769 | Nek2 |
| ENSRNOG00000061400 | -2.95946604951483 | 0.028214 | down | 10:-:56507122-56507244 | 10:-:56507122-56507244 | U2 |
| ENSRNOG00000050534 | -2.32228774913921 | 0.028216 | down | 1:+:236580034-236585035 | 1:+:236580034-236585035 | Gcnt1 |
| ENSRNOG00000018690 | 1.663012 | 0.028218 | up | 1:-:42492761-42587721 | 1:-:42492761-42587721 | Rgs17 |
| ENSRNOG00000024762 | -6.59448458056413 | 0.028278 | down | X:-:138098185-138148967 | X:-:138098185-138148967 | Frmd7 |
| ENSRNOG00000024266 | -1.25419572326829 | 0.028439 | down | 1:-:89109750-89124132 | 1:-:89109750-89124132 | Haus5 |
| ENSRNOG00000058460 | -4.56511051329422 | 0.028482 | down | 3:+:16846412-16847128 | 3:+:16846412-16847128 | AABR07051551.2 |
| ENSRNOG00000018735 | -1.94044087533993 | 0.028694 | down | 18:+:56071478-56080849 | 18:+:56071478-56080849 | Cd74 |
| ENSRNOG00000005498 | -3.02742214926377 | 0.028717 | down | 3:-:48442635-48451650 | 3:-:48442635-48451650 | Gcg |
| ENSRNOG00000003201 | -1.16979854112961 | 0.028838 | down | X:-:72074108-72078551 | X:-:72074108-72078551 | Rps4x |
| ENSRNOG00000046536 | 2.328969 | 0.028975 | up | 14:+:94590765-94602088 | 14:+:94590765-94602088 | AABR07016141.1 |
| ENSRNOG00000053044 | -3.40755814015212 | 0.029023 | down | 2:-:208225406-208225888 | 2:-:208225406-208225888 | AABR07012775.1 |
| ENSRNOG00000020602 | -1.41084151809434 | 0.029036 | down | 16:+:21275311-21282246 | 16:+:21275311-21282246 | Ndufa13 |
| ENSRNOG00000004680 | 1.472906 | 0.029147 | up | 3:+:35014538-35257407 | 3:+:35014538-35257407 | Kif5c |
| ENSRNOG00000047641 | -4.33381689489068 | 0.029204 | down | 6:-:138764502-138764901 | 6:-:138764502-138764901 | AABR07065651.3 |
| ENSRNOG00000016998 | 2.155744 | 0.029246 | up | 17:+:19249952-19533814 | 17:+:19249952-19533814 | Atxn1 |
| ENSRNOG00000062277 | 1.969192 | 0.029295 | up | 12:-:30495542-30496547 | 12:-:30495542-30496547 | AABR07036010.4 |
| ENSRNOG00000056151 | 2.403538 | 0.029308 | up | 2:-:21559368-21698937 | 2:-:21559368-21698937 | AABR07007642.1 |
| ENSRNOG00000048425 | -5.30741918729945 | 0.029314 | down | 6:-:140879774-140880070 | 6:-:140879774-140880070 | AABR07065776.3 |
| ENSRNOG00000007939 | -1.09643048773851 | 0.029408 | down | 7:-:116922944-116926555 | 7:-:116922944-116926555 | Naprt |
| ENSRNOG00000009433 | -3.20145957740192 | 0.029532 | down | 2:-:230199343-230273709 | 2:-:230199343-230273709 | Mcub |
| ENSRNOG00000048782 | -1.0723192912553 | 0.029548 | down | 4:-:122994425-123040609 | 4:-:122994425-123040609 | Wnt7a |
| ENSRNOG00000016580 | -1.5111058145528 | 0.029667 | down | 2:+:19823234-19824804 | 2:+:19823234-19824804 | Rps23 |
| ENSRNOG00000008236 | -1.25263627079648 | 0.029746 | down | 5:-:29573898-29601748 | 5:-:29573898-29601748 | Decr1 |
| ENSRNOG00000011011 | -4.72122477731216 | 0.029774 | down | 1:-:142721002-142724511 | 1:-:142721002-142724511 | Nmb |
| ENSRNOG00000058352 | -4.43301453329536 | 0.029868 | down | 6:+:139486775-139487576 | 6:+:139486775-139487576 | AABR07065699.4 |
| ENSRNOG00000008463 | 1.182663 | 0.029932 | up | 4:+:22445414-22586689 | 4:+:22445414-22586689 | Rundc3b |
| ENSRNOG00000034246 | -1.90855838184737 | 0.029954 | down | 5:-:138628482-138629026 | 5:-:138628482-138629026 | Rps27a |
| ENSRNOG00000004109 | 1.238555 | 0.02997 | up | 7:+:79638046-79964405 | 7:+:79638046-79964405 | Zfpm2 |
| ENSRNOG00000018045 | -3.06330007316084 | 0.029982 | down | 2:+:26085846-26086265 | 2:+:26085846-26086265 | AABR07007730.1 |
| ENSRNOG00000026647 | -1.15343097958203 | 0.030014 | down | 10:-:57060007-57064600 | 10:-:57060007-57064600 | Cxcl16 |
| ENSRNOG00000007066 | 3.356339 | 0.030031 | up | 4:+:199916-209599 | 4:+:199916-209599 | Htr5a |
| ENSRNOG00000001872 | 3.193039 | 0.03006 | up | 11:-:87418205-87434482 | 11:-:87418205-87434482 | Lrrc74b |
| ENSRNOG00000005438 | 1.098996 | 0.030109 | up | 3:+:137618898-137925605 | 3:+:137618898-137925605 | Pcsk2 |
| ENSRNOG00000047618 | -2.39342202238486 | 0.030247 | down | 12:+:7454884-7548614 | 12:+:7454884-7548614 | LOC103690050 |
| ENSRNOG00000010266 | -1.54061396900637 | 0.030365 | down | 2:+:32820322-32833125 | 2:+:32820322-32833125 | Cd180 |
| ENSRNOG00000030332 | -4.4343102550766 | 0.030385 | down | 6:-:139140679-139142218 | 6:-:139140679-139142218 | Ighg1 |
| ENSRNOG00000020942 | -2.24412053620886 | 0.030522 | down | 1:+:101517714-101540802 | 1:+:101517714-101540802 | Plekha4 |
| ENSRNOG00000001148 | -1.08262252718113 | 0.030574 | down | 12:-:46791528-46794797 | 12:-:46791528-46794797 | Rplp0 |
| ENSRNOG00000047401 | -4.50162315555624 | 0.030594 | down | 1:-:101173382-101175570 | 1:-:101173382-101175570 | Gfy |
| ENSRNOG00000052664 | 4.245443 | 0.030634 | up | 1:+:255479261-255532143 | 1:+:255479261-255532143 | Tnks2 |
| ENSRNOG00000011748 | -5.40743728346881 | 0.030684 | down | 2:+:190003223-190005871 | 2:+:190003223-190005871 | S100a5 |
| ENSRNOG00000016480 | -3.74899087237273 | 0.030708 | down | 17:-:78904873-78910671 | 17:-:78904873-78910671 | Acbd7 |
| ENSRNOG00000004180 | 1.19796 | 0.030709 | up | 7:-:66017086-66172360 | 7:-:66017086-66172360 | Tafa2 |
| ENSRNOG00000005708 | 1.091111 | 0.030776 | up | 5:+:31568419-31810484 | 5:+:31568419-31810484 | Mmp16 |
| ENSRNOG00000005159 | 2.072634 | 0.030777 | up | 7:-:112673465-112833083 | 7:-:112673465-112833083 | Fam135b |
| ENSRNOG00000009821 | 1.677868 | 0.030821 | up | 2:-:170452702-170460754 | 2:-:170452702-170460754 | Slitrk3 |
| ENSRNOG00000006509 | 2.043066 | 0.030881 | up | 4:-:144638335-144869919 | 4:-:144638335-144869919 | Srgap3 |
| ENSRNOG00000011937 | 1.688939 | 0.030919 | up | 2:+:34186091-34219802 | 2:+:34186091-34219802 | Sgtb |
| ENSRNOG00000055375 | -4.66867046432728 | 0.03093 | down | 6:-:143194515-143195445 | 6:-:143194515-143195445 | AABR07065837.1 |
| ENSRNOG00000027393 | 2.152132 | 0.030938 | up | 3:-:3699826-3700200 | 3:-:3699826-3700200 | AC129824.1 |
| ENSRNOG00000016411 | -1.54657212671986 | 0.030958 | down | 1:+:22758212-22760370 | 1:+:22758212-22760370 | Rps12 |
| ENSRNOG00000016625 | -3.35252966469505 | 0.031085 | down | 1:-:48317995-48360261 | 1:-:48317995-48360261 | Slc22a2 |
| ENSRNOG00000010065 | 3.774939 | 0.031095 | up | 15:-:60803098-60959769 | 15:-:60803098-60959769 | Dgkh |
| ENSRNOG00000014227 | -1.99559400619601 | 0.031141 | down | 1:-:103298174-103323476 | 1:-:103298174-103323476 | Mrgprx3 |
| ENSRNOG00000047247 | 1.264358 | 0.031224 | up | 9:+:10603813-10645939 | 9:+:10603813-10645939 | Ptprs |
| ENSRNOG00000019958 | 1.189552 | 0.031347 | up | 9:+:17841410-17845921 | 9:+:17841410-17845921 | Tmem151b |
| ENSRNOG00000006735 | -2.23237331943366 | 0.031407 | down | 5:-:107845951-107858104 | 5:-:107845951-107858104 | Cdkn2b |
| ENSRNOG00000029134 | 1.402913 | 0.031408 | up | 19:+:25029037-25069142 | 19:+:25029037-25069142 | Adgrl1 |
| ENSRNOG00000033266 | -3.20532157347953 | 0.031428 | down | 10:-:13348307-13352371 | 10:-:13348307-13352371 | Prss30 |
| ENSRNOG00000004281 | 1.942648 | 0.031431 | up | 14:-:92342957-92577936 | 14:-:92342957-92577936 | Cobl |
| ENSRNOG00000014723 | 2.073873 | 0.031442 | up | 19:-:55441572-55510460 | 19:-:55441572-55510460 | Cbfa2t3 |
| ENSRNOG00000033894 | 3.489086 | 0.031486 | up | 16:-:15764632-15798974 | 16:-:15764632-15798974 | AABR07024769.1 |
| ENSRNOG00000007894 | 1.026768 | 0.031486 | up | 5:-:99413187-99566318 | 5:-:99413187-99566318 | Mpdz |
| ENSRNOG00000053753 | -1.55724875471483 | 0.031504 | down | 8:-:64811941-64833697 | 8:-:64811941-64833697 | AABR07070307.1 |
| ENSRNOG00000000105 | 2.010215 | 0.031519 | up | 17:-:10756285-10818835 | 17:-:10756285-10818835 | Cplx2 |
| ENSRNOG00000008615 | 1.576838 | 0.031666 | up | 7:+:94130852-94163645 | 7:+:94130852-94163645 | Mal2 |
| ENSRNOG00000008862 | 2.133592 | 0.031669 | up | 8:-:48606403-48619592 | 8:-:48606403-48619592 | Abcg4 |
| ENSRNOG00000028505 | -1.11963354607844 | 0.031711 | down | 20:+:5441876-5445553 | 20:+:5441876-5445553 | Rps18 |
| ENSRNOG00000001963 | -1.28489309933261 | 0.031723 | down | 11:+:38035450-38059950 | 11:+:38035450-38059950 | Mx2 |
| ENSRNOG00000054334 | -2.51939902269638 | 0.031779 | down | 11:+:60054408-60066739 | 11:+:60054408-60066739 | Abhd10 |
| ENSRNOG00000012789 | 3.989941 | 0.031871 | up | 19:-:55176258-55183557 | 19:-:55176258-55183557 | Mlnr |
| ENSRNOG00000055499 | 1.640231 | 0.032025 | up | 16:+:11599753-11932324 | 16:+:11599753-11932324 | Grid1 |
| ENSRNOG00000055185 | 3.631155 | 0.032029 | up | X:-:157168144-157172068 | X:-:157168144-157172068 | Dusp9 |
| ENSRNOG00000004688 | 1.421909 | 0.032049 | up | 7:-:118156946-118396728 | 7:-:118156946-118396728 | Rbfox2 |
| ENSRNOG00000019879 | -2.90442492866998 | 0.032176 | down | 1:-:85218803-85220237 | 1:-:85218803-85220237 | Sycn |
| ENSRNOG00000008423 | 1.400632 | 0.032226 | up | 6:-:51011426-51019407 | 6:-:51011426-51019407 | Gpr22 |
| ENSRNOG00000027849 | 1.91067 | 0.032231 | up | 10:+:86795787-86812325 | 10:+:86795787-86812325 | Wipf2 |
| ENSRNOG00000003620 | -2.02074331866048 | 0.032249 | down | 13:-:80837420-80862963 | 13:-:80837420-80862963 | Fmo3 |
| ENSRNOG00000011119 | 1.517379 | 0.032307 | up | 16:-:884861-930527 | 16:-:884861-930527 | Spin1 |
| ENSRNOG00000061431 | -5.3448690773598 | 0.032421 | down | 4:+:106323089-106323738 | 4:+:106323089-106323738 | AABR07061134.1 |
| ENSRNOG00000025406 | 1.94485 | 0.032437 | up | 2:-:26167199-26438790 | 2:-:26167199-26438790 | Iqgap2 |
| ENSRNOG00000007706 | 3.423113 | 0.032457 | up | 5:-:124574079-124642569 | 5:-:124574079-124642569 | Prkaa2 |
| ENSRNOG00000018795 | -1.38977820971597 | 0.032599 | down | 16:+:20293229-20295294 | 16:+:20293229-20295294 | Rpl18a |
| ENSRNOG00000000861 | -1.81111146255885 | 0.032634 | down | X:+:140878216-140888795 | X:+:140878216-140888795 | Zic3 |
| ENSRNOG00000055751 | 2.42374 | 0.032763 | up | 11:+:78029038-78169648 | 11:+:78029038-78169648 | P3h2 |
| ENSRNOG00000006766 | -1.31404012312816 | 0.032799 | down | 7:+:72924799-72968101 | 7:+:72924799-72968101 | Laptm4b |
| ENSRNOG00000042478 | 2.448398 | 0.033233 | up | 4:+:22859622-22996645 | 4:+:22859622-22996645 | Adam22 |
| ENSRNOG00000008857 | -1.00521975348178 | 0.033499 | down | 7:+:18409147-18439012 | 7:+:18409147-18439012 | Adamts10 |
| ENSRNOG00000010189 | -1.66567409455319 | 0.033563 | down | 16:+:757403-762075 | 16:+:757403-762075 | Rps24 |
| ENSRNOG00000020557 | -1.51839849397689 | 0.033573 | down | 1:-:87959712-88066101 | 1:-:87959712-88066101 | Ryr1 |
| ENSRNOG00000059876 | 1.467654 | 0.033577 | up | 5:+:70441123-70511659 | 5:+:70441123-70511659 | Fsd1l |
| ENSRNOG00000020060 | -1.00806294081355 | 0.033583 | down | 1:-:100808241-100810522 | 1:-:100808241-100810522 | Atf5 |
| ENSRNOG00000015277 | -1.59590589108117 | 0.033623 | down | 3:+:113415774-113417478 | 3:+:113415774-113417478 | Serf2 |
| ENSRNOG00000058166 | 2.704076 | 0.033646 | up | 7:+:74350479-74406295 | 7:+:74350479-74406295 | AABR07057495.1 |
| ENSRNOG00000026989 | -2.1114678005708 | 0.033682 | down | 2:-:41869556-41871858 | 2:-:41869556-41871858 | Gapt |
| ENSRNOG00000006356 | 1.919967 | 0.033758 | up | 14:-:114834771-114868328 | 14:-:114834771-114868328 | RGD1562229 |
| ENSRNOG00000027040 | -1.14377371262316 | 0.033827 | down | 15:-:109307904-109316953 | 15:-:109307904-109316953 | Ggact |
| ENSRNOG00000003614 | 2.004482 | 0.033913 | up | 13:+:43850751-44157924 | 13:+:43850751-44157924 | Mgat5 |
| ENSRNOG00000000708 | 1.053109 | 0.033956 | up | 12:+:49328977-49395125 | 12:+:49328977-49395125 | Sgsm1 |
| ENSRNOG00000003875 | 1.767647 | 0.033976 | up | X:+:134742356-134792618 | X:+:134742356-134792618 | Ocrl |
| ENSRNOG00000009372 | 1.740593 | 0.034078 | up | 2:+:240021152-240118971 | 2:+:240021152-240118971 | Tacr3 |
| ENSRNOG00000014702 | -1.33998758663852 | 0.034154 | down | 17:+:21382455-21422407 | 17:+:21382455-21422407 | Elovl2 |
| ENSRNOG00000011893 | -1.3644113807001 | 0.034293 | down | 2:-:185440291-185444897 | 2:-:185440291-185444897 | Rps3a |
| ENSRNOG00000054191 | -1.98257723167783 | 0.034318 | down | 1:+:228362665-228362786 | 1:+:228362665-228362786 | AABR07072030.1 |
| ENSRNOG00000031397 | 2.667863 | 0.034333 | up | 14:+:77829400-78097919 | 14:+:77829400-78097919 | Stk32b |
| ENSRNOG00000008949 | 2.534053 | 0.034364 | up | 15:+:4064706-4072635 | 15:+:4064706-4072635 | Synpo2l |
| ENSRNOG00000011431 | 1.620393 | 0.034507 | up | 16:-:60128201-60208462 | 16:-:60128201-60208462 | Mfhas1 |
| ENSRNOG00000010626 | -2.6025020728287 | 0.034537 | down | 10:+:105498728-105504393 | 10:+:105498728-105504393 | Sphk1 |
| ENSRNOG00000007686 | 1.262106 | 0.034617 | up | 7:+:123510804-123526542 | 7:+:123510804-123526542 | Septin3 |
| ENSRNOG00000045621 | -3.22257296363143 | 0.034626 | down | 12:+:47031595-47036956 | 12:+:47031595-47036956 | Gatc |
| ENSRNOG00000002092 | 3.014862 | 0.034671 | up | 11:+:30550141-30663465 | 11:+:30550141-30663465 | Hunk |
| ENSRNOG00000059121 | -3.54270648498803 | 0.03468 | down | 6:-:139783839-139911839 | 6:-:139783839-139911839 | AABR07065714.1 |
| ENSRNOG00000017409 | -2.73531738912399 | 0.034719 | down | 9:+:82033543-82047166 | 9:+:82033543-82047166 | Wnt6 |
| ENSRNOG00000008475 | 1.437935 | 0.034793 | up | 5:-:40032855-40237591 | 5:-:40032855-40237591 | Fut9 |
| ENSRNOG00000022248 | 1.92674 | 0.03482 | up | 10:+:15183803-15186978 | 10:+:15183803-15186978 | Fbxl16 |
| ENSRNOG00000010083 | -1.00011364903045 | 0.034963 | down | 10:-:105430520-105452228 | 10:-:105430520-105452228 | Prpsap1 |
| ENSRNOG00000017401 | -1.45659609255376 | 0.034971 | down | 5:+:157423213-157501737 | 5:+:157423213-157501737 | Tmco4 |
| ENSRNOG00000018158 | 1.358243 | 0.034974 | up | 1:+:47162394-47202549 | 1:+:47162394-47202549 | Tmem181 |
| ENSRNOG00000027596 | 1.139152 | 0.034988 | up | 3:+:33440191-33504238 | 3:+:33440191-33504238 | Mbd5 |
| ENSRNOG00000017703 | -1.13760802636361 | 0.035089 | down | 1:+:219144205-219183963 | 1:+:219144205-219183963 | Unc93b1 |
| ENSRNOG00000016220 | -1.06192403223223 | 0.035097 | down | 3:+:12009578-12011666 | 3:+:12009578-12011666 | Rpl12 |
| ENSRNOG00000001141 | 3.58004 | 0.035119 | up | 12:+:45727112-45882548 | 12:+:45727112-45882548 | Srrm4 |
| ENSRNOG00000009845 | -1.13595556480476 | 0.03513 | down | 2:-:260124418-260148589 | 2:-:260124418-260148589 | Acadm |
| ENSRNOG00000058335 | 2.546042 | 0.035143 | up | 3:-:25335682-25351082 | 3:-:25335682-25351082 | AABR07051879.1 |
| ENSRNOG00000037951 | 2.067954 | 0.035195 | up | X:+:71960851-71972810 | X:+:71960851-71972810 | AABR07039210.1 |
| ENSRNOG00000007528 | 2.055453 | 0.035212 | up | 3:-:48671079-48831467 | 3:-:48671079-48831467 | Kcnh7 |
| ENSRNOG00000025551 | 3.130388 | 0.035264 | up | 7:-:74783019-74901997 | 7:-:74783019-74901997 | Rgs22 |
| ENSRNOG00000019357 | -1.04417395889307 | 0.035312 | down | 10:+:59743544-59748062 | 10:+:59743544-59748062 | Tax1bp3 |
| ENSRNOG00000020254 | 1.380809 | 0.035335 | up | 9:-:98555169-98597359 | 9:-:98555169-98597359 | Per2 |
| ENSRNOG00000008409 | -1.38099530529811 | 0.035368 | down | 7:+:18440742-18491448 | 7:+:18440742-18491448 | Myo1f |
| ENSRNOG00000050647 | 2.480147 | 0.035452 | up | 20:-:4877324-4879779 | 20:-:4877324-4879779 | Hspa1b |
| ENSRNOG00000016581 | -1.57889089822008 | 0.03548 | down | 17:+:32904119-32911495 | 17:+:32904119-32911495 | Serpinb1a |
| ENSRNOG00000012181 | 1.309156 | 0.035571 | up | 16:-:22537056-22561496 | 16:-:22537056-22561496 | Lpl |
| ENSRNOG00000047206 | -2.01491265956594 | 0.035599 | down | 1:+:75233703-75264293 | 1:+:75233703-75264293 | LOC100911727 |
| ENSRNOG00000012562 | -1.18279052112742 | 0.035613 | down | 7:+:12764993-12771310 | 7:+:12764993-12771310 | Grin3b |
| ENSRNOG00000030714 | 1.95856 | 0.035729 | up | 8:-:116873721-116965396 | 8:-:116873721-116965396 | Bsn |
| ENSRNOG00000013572 | -1.26879280649951 | 0.035758 | down | 2:-:164628566-164634434 | 2:-:164628566-164634434 | Lxn |
| ENSRNOG00000005883 | 3.300679 | 0.035766 | up | 15:-:11632759-11812485 | 15:-:11632759-11812485 | Nek10 |
| ENSRNOG00000000490 | -1.33189652841332 | 0.035821 | down | 20:-:7215774-7219548 | 20:-:7215774-7219548 | Rps10 |
| ENSRNOG00000054121 | 1.496731 | 0.035846 | up | 4:-:108661494-108717309 | 4:-:108661494-108717309 | AABR07061178.1 |
| ENSRNOG00000019974 | -1.39758716663104 | 0.035915 | down | 16:+:20668971-20671127 | 16:+:20668971-20671127 | Uba52 |
| ENSRNOG00000008533 | 1.625258 | 0.035988 | up | 7:-:114339434-114380613 | 7:-:114339434-114380613 | Ago2 |
| ENSRNOG00000004430 | -1.06738714737558 | 0.035993 | down | 10:-:109243039-109267500 | 10:-:109243039-109267500 | Cep131 |
| ENSRNOG00000004641 | 3.323635 | 0.035999 | up | 3:+:142739781-142741644 | 3:+:142739781-142741644 | Sstr4 |
| ENSRNOG00000019578 | -1.42110683148904 | 0.036 | down | 1:-:85405512-85408444 | 1:-:85405512-85408444 | Rps16 |
| ENSRNOG00000008471 | 1.43747 | 0.036036 | up | 13:+:52976507-53023103 | 13:+:52976507-53023103 | Kif21b |
| ENSRNOG00000049580 | 2.728787 | 0.03612 | up | 20:-:45813169-45815940 | 20:-:45813169-45815940 | Gpr6 |
| ENSRNOG00000016058 | -1.83927261557317 | 0.036165 | down | 1:+:264827739-264831151 | 1:+:264827739-264831151 | Kazald1 |
| ENSRNOG00000021035 | -1.24742296257324 | 0.036183 | down | 1:+:101701975-101703550 | 1:+:101701975-101703550 | Rpl18 |
| ENSRNOG00000018162 | -2.55067812017548 | 0.036187 | down | 9:-:82230232-82327534 | 9:-:82230232-82327534 | Nhej1 |
| ENSRNOG00000028384 | -1.17053696900289 | 0.036444 | down | 3:+:172195844-172207685 | 3:+:172195844-172207685 | Npepl1 |
| ENSRNOG00000055858 | 2.691599 | 0.036534 | up | 1:-:16659442-16687817 | 1:-:16659442-16687817 | Myb |
| ENSRNOG00000020583 | -1.0572919982852 | 0.036572 | down | 1:-:101086877-101095594 | 1:-:101086877-101095594 | Fcgrt |
| ENSRNOG00000014862 | 2.906311 | 0.036634 | up | 1:-:112158519-112811936 | 1:-:112158519-112811936 | Gabrg3 |
| ENSRNOG00000023529 | -1.21382139865926 | 0.036736 | down | 14:-:2860965-2867397 | 14:-:2860965-2867397 | Rpl5 |
| ENSRNOG00000021250 | 3.082978 | 0.036767 | up | 3:-:123848825-123849788 | 3:-:123848825-123849788 | Rnf24 |
| ENSRNOG00000009863 | 1.026994 | 0.036833 | up | 4:-:145450861-145454834 | 4:-:145450861-145454834 | Prrt3 |
| ENSRNOG00000018774 | -1.56642165108922 | 0.036903 | down | 18:+:56044369-56047316 | 18:+:56044369-56047316 | LOC100911847 |
| ENSRNOG00000013668 | -1.52111837473228 | 0.036905 | down | 4:+:100407658-100419446 | 4:+:100407658-100419446 | Capg |
| ENSRNOG00000038211 | 2.57422 | 0.036984 | up | 18:-:85989490-85990860 | 18:-:85989490-85990860 | AABR07032856.1 |
| ENSRNOG00000000185 | -1.080442087819 | 0.036998 | down | 7:+:119626637-119631425 | 7:+:119626637-119631425 | Mpst |
| ENSRNOG00000009260 | -1.83185929534067 | 0.037005 | down | 5:+:154260062-154268126 | 5:+:154260062-154268126 | Cnr2 |
| ENSRNOG00000017277 | -1.98931358048543 | 0.037008 | down | 1:-:190997029-191007503 | 1:-:190997029-191007503 | Igsf6 |
| ENSRNOG00000026271 | 1.827439 | 0.03722 | up | 5:-:73918747-73986373 | 5:-:73918747-73986373 | Tmem245 |
| ENSRNOG00000017072 | 1.834557 | 0.037282 | up | 9:-:92503597-92530938 | 9:-:92503597-92530938 | Slc16a14 |
| ENSRNOG00000000815 | -1.04468231280667 | 0.037356 | down | 20:+:40778927-40800868 | 20:+:40778927-40800868 | Smpdl3a |
| ENSRNOG00000003603 | 2.032444 | 0.037534 | up | 10:-:51502894-51669297 | 10:-:51502894-51669297 | Arhgap44 |
| ENSRNOG00000019106 | -1.4104655738445 | 0.037575 | down | 1:-:143167362-143169657 | 1:-:143167362-143169657 | Rps17 |
| ENSRNOG00000011107 | 2.097007 | 0.037656 | up | 2:-:91450162-91497091 | 2:-:91450162-91497091 | Pcsk1 |
| ENSRNOG00000009815 | 1.498007 | 0.037685 | up | 15:+:43582020-43589680 | 15:+:43582020-43589680 | Pnma2 |
| ENSRNOG00000047466 | 1.346993 | 0.037707 | up | 3:+:100768637-100819210 | 3:+:100768637-100819210 | Bdnf |
| ENSRNOG00000056314 | -2.63416812079907 | 0.037834 | down | 7:-:22495523-22497727 | 7:-:22495523-22497727 | AABR07056330.1 |
| ENSRNOG00000014948 | -2.44144622424538 | 0.037852 | down | 19:+:52077109-52085496 | 19:+:52077109-52085496 | Osgin1 |
| ENSRNOG00000036615 | 2.618186 | 0.037918 | up | 1:-:241338704-241460868 | 1:-:241338704-241460868 | RGD1560242 |
| ENSRNOG00000018371 | -1.08353800479326 | 0.037926 | down | 18:+:63130542-63140181 | 18:+:63130542-63140181 | Tubb6 |
| ENSRNOG00000006144 | 1.4054 | 0.037927 | up | 4:-:134784668-135069970 | 4:-:134784668-135069970 | Cntn3 |
| ENSRNOG00000011460 | 4.085036 | 0.038031 | up | 1:-:13838707-13915594 | 1:-:13838707-13915594 | Arfgef3 |
| ENSRNOG00000000390 | 2.932982 | 0.038042 | up | 20:-:27220707-27247309 | 20:-:27220707-27247309 | Rufy2 |
| ENSRNOG00000010575 | -1.68219413901426 | 0.038059 | down | 2:-:243175346-243224883 | 2:-:243175346-243224883 | Dapp1 |
| ENSRNOG00000047446 | -1.62306238337141 | 0.038168 | down | 19:+:53044379-53047081 | 19:+:53044379-53047081 | Foxc2 |
| ENSRNOG00000012067 | 3.628416 | 0.038201 | up | 1:+:229003961-229019527 | 1:+:229003961-229019527 | Fam111a |
| ENSRNOG00000002079 | 1.114817 | 0.038262 | up | 14:+:8080275-8368254 | 14:+:8080275-8368254 | Mapk10 |
| ENSRNOG00000019675 | -2.02437673750241 | 0.038355 | down | 1:-:199437832-199439210 | 1:-:199437832-199439210 | Pycard |
| ENSRNOG00000018086 | -1.3830037823189 | 0.038377 | down | 1:+:224800252-224818482 | 1:+:224800252-224818482 | Slc22a8 |
| ENSRNOG00000059262 | 2.803916 | 0.03841 | up | 16:-:16454743-16473307 | 16:-:16454743-16473307 | AABR07024786.1 |
| ENSRNOG00000025860 | 3.350349 | 0.038444 | up | 19:-:10363108-10380809 | 19:-:10363108-10380809 | Drc7 |
| ENSRNOG00000000857 | -3.25326769850643 | 0.038466 | down | 20:-:5020667-5037088 | 20:-:5020667-5037088 | Msh5 |
| ENSRNOG00000009822 | -1.35931648286839 | 0.038539 | down | 2:-:182840727-182846061 | 2:-:182840727-182846061 | Tlr2 |
| ENSRNOG00000030318 | 1.938193 | 0.038569 | up | 8:+:13365583-13376070 | 8:+:13365583-13376070 | Gpr83 |
| ENSRNOG00000008674 | 3.285961 | 0.038633 | up | 3:-:81867438-81911197 | 3:-:81867438-81911197 | Prdm11 |
| ENSRNOG00000004284 | -1.52849865283427 | 0.038689 | down | 7:+:37812831-37815088 | 7:+:37812831-37815088 | Btg1 |
| ENSRNOG00000012942 | 1.61667 | 0.03869 | up | 9:+:2190915-2274629 | 9:+:2190915-2274629 | Satb1 |
| ENSRNOG00000008428 | 1.903634 | 0.038718 | up | 8:+:53678777-53743642 | 8:+:53678777-53743642 | Drd2 |
| ENSRNOG00000030575 | -3.54132498964662 | 0.038758 | down | 2:+:223029559-223045185 | 2:+:223029559-223045185 | AABR07013111.1 |
| ENSRNOG00000003959 | 2.561157 | 0.038791 | up | 13:-:61565483-61591139 | 13:-:61565483-61591139 | Rgs18 |
| ENSRNOG00000003479 | 1.437346 | 0.038855 | up | 19:+:24044103-24265444 | 19:+:24044103-24265444 | Rnf150 |
| ENSRNOG00000004676 | 2.467159 | 0.038904 | up | X:+:114929029-115036669 | X:+:114929029-115036669 | Pak3 |
| ENSRNOG00000004841 | 1.901712 | 0.038963 | up | 6:+:73553210-73990534 | 6:+:73553210-73990534 | Akap6 |
| ENSRNOG00000055426 | 2.47723 | 0.038995 | up | 5:-:150483134-150484207 | 5:-:150483134-150484207 | Rab42 |
| ENSRNOG00000056658 | 2.255765 | 0.039095 | up | 1:+:187149453-187261632 | 1:+:187149453-187261632 | Xylt1 |
| ENSRNOG00000051073 | 2.666291 | 0.039127 | up | X:-:115599716-115625360 | X:-:115599716-115625360 | AABR07040953.1 |
| ENSRNOG00000012156 | -1.16743418085838 | 0.039165 | down | 1:+:234749568-234796739 | 1:+:234749568-234796739 | Ostf1 |
| ENSRNOG00000007326 | 1.358327 | 0.03921 | up | 6:-:8318979-8346197 | 6:-:8318979-8346197 | Prepl |
| ENSRNOG00000019328 | -1.03905391395962 | 0.039222 | down | 2:-:200484246-200513564 | 2:-:200484246-200513564 | Phgdh |
| ENSRNOG00000047080 | -2.24808375056527 | 0.03945 | down | 17:-:90266794-90315492 | 17:-:90266794-90315492 | Gng4 |
| ENSRNOG00000048986 | 2.326429 | 0.039475 | up | 6:+:129320389-129320640 | 6:+:129320389-129320640 | D430019H16Rik |
| ENSRNOG00000029410 | -1.27106166945629 | 0.03951 | down | 13:-:30799969-30800451 | 13:-:30799969-30800451 | Rpl21 |
| ENSRNOG00000048053 | -2.65988146595831 | 0.039548 | down | 10:+:70398995-70400317 | 10:+:70398995-70400317 | AC128859.3 |
| ENSRNOG00000011310 | 3.975479 | 0.039549 | up | 1:-:52360296-52544450 | 1:-:52360296-52544450 | Pde10a |
| ENSRNOG00000020486 | -1.8851024167864 | 0.039575 | down | 1:-:100984550-100993269 | 1:-:100984550-100993269 | Bcl2l12 |
| ENSRNOG00000059008 | 1.725909 | 0.039644 | up | 10:+:85301875-85330397 | 10:+:85301875-85330397 | Socs7 |
| ENSRNOG00000013413 | 1.393557 | 0.039716 | up | 1:+:234252757-234435839 | 1:+:234252757-234435839 | Rorb |
| ENSRNOG00000052840 | 2.752674 | 0.039772 | up | 10:+:93811350-94127697 | 10:+:93811350-94127697 | Tanc2 |
| ENSRNOG00000023794 | 2.614232 | 0.039779 | up | 8:+:89369417-89556942 | 8:+:89369417-89556942 | Mei4 |
| ENSRNOG00000012531 | 1.338723 | 0.039809 | up | 5:-:155022493-155204456 | 5:-:155022493-155204456 | Ephb2 |
| ENSRNOG00000012503 | 1.29865 | 0.039838 | up | 5:-:160325107-160352927 | 5:-:160325107-160352927 | Dnajc16 |
| ENSRNOG00000000156 | -2.45870951105322 | 0.039964 | down | 5:+:171472273-171620947 | 5:+:171472273-171620947 | LOC100911486 |
| ENSRNOG00000020778 | 1.761622 | 0.040024 | up | 2:-:189088570-189096785 | 2:-:189088570-189096785 | Chrnb2 |
| ENSRNOG00000032844 | -1.91910145434882 | 0.040042 | down | 20:-:4127644-4132616 | 20:-:4127644-4132616 | RT1-Da |
| ENSRNOG00000018778 | 1.331291 | 0.040125 | up | 8:+:52127399-52189722 | 8:+:52127399-52189722 | Cadm1 |
| ENSRNOG00000015903 | 1.43902 | 0.040304 | up | 4:+:117743710-117882464 | 4:+:117743710-117882464 | Add2 |
| ENSRNOG00000009184 | 1.306439 | 0.040364 | up | 4:-:131366744-131694755 | 4:-:131366744-131694755 | Foxp1 |
| ENSRNOG00000008011 | 2.705147 | 0.0404 | up | 5:+:131380297-131455211 | 5:+:131380297-131455211 | Agbl4 |
| ENSRNOG00000008001 | -1.54825202592872 | 0.040408 | down | 5:+:128501847-128568188 | 5:+:128501847-128568188 | Rab3b |
| ENSRNOG00000012634 | 1.747026 | 0.040424 | up | 5:-:60591023-60658564 | 5:-:60591023-60658564 | Fbxo10 |
| ENSRNOG00000031090 | -1.64673175938324 | 0.040451 | down | 20:-:4694472-4896970 | 20:-:4694472-4896970 | RT1-CE7 |
| ENSRNOG00000047931 | -1.03101501556806 | 0.040459 | down | X:+:28593405-28595395 | X:+:28593405-28595395 | Tmsb4x |
| ENSRNOG00000002327 | 1.288361 | 0.040493 | up | 14:-:38643385-39112600 | 14:-:38643385-39112600 | Gabrb1 |
| ENSRNOG00000002129 | 2.127102 | 0.040511 | up | 14:-:5418741-5428757 | 14:-:5418741-5428757 | Lrrc8b |
| ENSRNOG00000051837 | 1.771846 | 0.040544 | up | 14:-:46136786-46153212 | 14:-:46136786-46153212 | Nwd2 |
| ENSRNOG00000004423 | 2.373513 | 0.040558 | up | 13:+:95589668-95591236 | 13:+:95589668-95591236 | Zbtb18 |
| ENSRNOG00000043233 | -1.34606274601273 | 0.040577 | down | 7:-:15198940-15225410 | 7:-:15198940-15225410 | Cyp4f6 |
| ENSRNOG00000039086 | 2.92375 | 0.040593 | up | 8:+:48569328-48577855 | 8:+:48569328-48577855 | Ccdc153 |
| ENSRNOG00000016768 | 1.247378 | 0.040636 | up | 3:+:177225737-177231663 | 3:+:177225737-177231663 | Oprl1 |
| ENSRNOG00000050052 | -1.03092957772605 | 0.040647 | down | 12:+:17395624-17405103 | 12:+:17395624-17405103 | Cox19 |
| ENSRNOG00000011821 | -2.40554181025342 | 0.040648 | down | 2:+:189997129-189999604 | 2:+:189997129-189999604 | S100a4 |
| ENSRNOG00000008869 | 2.395532 | 0.04067 | up | 4:+:29978739-30241911 | 4:+:29978739-30241911 | Ppp1r9a |
| ENSRNOG00000000894 | 1.48461 | 0.040869 | up | 12:-:5573729-5822874 | 12:-:5573729-5822874 | Fry |
| ENSRNOG00000032656 | 1.851397 | 0.040913 | up | 3:-:157544583-158328881 | 3:-:157544583-158328881 | Ptprt |
| ENSRNOG00000027157 | -4.8206972032479 | 0.040932 | down | 20:+:5067330-5068624 | 20:+:5067330-5068624 | Ly6g6e |
| ENSRNOG00000061688 | -1.99942101527278 | 0.041057 | down | 8:+:13868558-13868688 | 8:+:13868558-13868688 | AC105648.11 |
| ENSRNOG00000006472 | -1.03855883644308 | 0.041131 | down | 6:+:99433550-99436289 | 6:+:99433550-99436289 | Hspa2 |
| ENSRNOG00000059149 | 2.486863 | 0.041141 | up | 10:-:90700111-90709932 | 10:-:90700111-90709932 | AABR07030498.1 |
| ENSRNOG00000049403 | 1.759928 | 0.04139 | up | 13:-:43494350-43566680 | 13:-:43494350-43566680 | LOC108348294 |
| ENSRNOG00000055023 | -2.18608111272005 | 0.041551 | down | 1:+:226258417-226258557 | 1:+:226258417-226258557 | AABR07006258.2 |
| ENSRNOG00000054017 | -1.51475074884309 | 0.041564 | down | 17:+:44522140-44526583 | 17:+:44522140-44526583 | LOC100912564 |
| ENSRNOG00000000916 | 2.167526 | 0.041658 | up | 12:+:7208850-7271872 | 12:+:7208850-7271872 | LOC100910196 |
| ENSRNOG00000021062 | -1.37046671199831 | 0.041766 | down | 1:-:89464860-89474252 | 1:-:89464860-89474252 | Fxyd5 |
| ENSRNOG00000012302 | 1.291075 | 0.041778 | up | 2:-:181045703-181102918 | 2:-:181045703-181102918 | Gucy1a1 |
| ENSRNOG00000001435 | -2.11328975914927 | 0.041796 | down | 12:+:23752844-23762877 | 12:+:23752844-23762877 | Ssc4d |
| ENSRNOG00000011138 | -1.11890343993052 | 0.041922 | down | 8:+:115131367-115133401 | 8:+:115131367-115133401 | Rpl29 |
| ENSRNOG00000017093 | -1.01665671521507 | 0.041945 | down | 17:+:30965942-30994410 | 17:+:30965942-30994410 | Pxdc1 |
| ENSRNOG00000008169 | 3.465099 | 0.041966 | up | 5:-:105336262-105582375 | 5:-:105336262-105582375 | Slc24a2 |
| ENSRNOG00000036911 | 1.396352 | 0.041973 | up | 4:+:184019087-184165540 | 4:+:184019087-184165540 | Bicd1 |
| ENSRNOG00000014258 | -1.39002605151732 | 0.042033 | down | 1:-:4637491-4653210 | 1:-:4637491-4653210 | Rab32 |
| ENSRNOG00000020652 | -1.21957142368522 | 0.042056 | down | 1:+:82480195-82497199 | 1:+:82480195-82497199 | Tgfb1 |
| ENSRNOG00000053424 | 2.681547 | 0.042128 | up | 1:+:260832285-260832421 | 1:+:260832285-260832421 | AABR07006860.1 |
| ENSRNOG00000020845 | -1.81697830547203 | 0.042129 | down | 1:+:88875375-88879303 | 1:+:88875375-88879303 | Tyrobp |
| ENSRNOG00000021589 | 2.403058 | 0.04213 | up | X:-:74945082-74968405 | X:-:74945082-74968405 | Nexmif |
| ENSRNOG00000053064 | 2.175721 | 0.042137 | up | 1:-:126386929-126403406 | 1:-:126386929-126403406 | AABR07004130.1 |
| ENSRNOG00000018775 | -1.34632397847142 | 0.042152 | down | 18:+:29191731-29232978 | 18:+:29191731-29232978 | Cystm1 |
| ENSRNOG00000019288 | 2.16152 | 0.042157 | up | 19:-:11425105-11451278 | 19:-:11425105-11451278 | Ogfod1 |
| ENSRNOG00000008992 | 3.747423 | 0.042178 | up | 5:+:143500441-143715546 | 5:+:143500441-143715546 | Grik3 |
| ENSRNOG00000050430 | -1.55315278883123 | 0.042253 | down | 9:-:9617786-9675110 | 9:-:9617786-9675110 | Vav1 |
| ENSRNOG00000020350 | 1.398628 | 0.042293 | up | 1:-:220516316-220644636 | 1:-:220516316-220644636 | Pacs1 |
| ENSRNOG00000038074 | -1.66385412935744 | 0.042297 | down | 15:+:75067297-75067723 | 15:+:75067297-75067723 | LOC498555 |
| ENSRNOG00000026605 | -2.85225420380698 | 0.042326 | down | 6:-:127336305-127337791 | 6:-:127336305-127337791 | Ifi27l2b |
| ENSRNOG00000027276 | 3.355697 | 0.042337 | up | 5:-:15747614-16140896 | 5:-:15747614-16140896 | Xkr4 |
| ENSRNOG00000021904 | 1.282362 | 0.042341 | up | 6:+:135313008-135402814 | 6:+:135313008-135402814 | Tecpr2 |
| ENSRNOG00000012486 | -1.08988310522674 | 0.042361 | down | 9:+:38536920-38745099 | 9:+:38536920-38745099 | Prim2 |
| ENSRNOG00000000957 | -1.57151630012025 | 0.042397 | down | 12:-:9996951-9998779 | 12:-:9996951-9998779 | Rpl21 |
| ENSRNOG00000030840 | 1.787893 | 0.042447 | up | 11:-:4174881-4397361 | 11:-:4174881-4397361 | Cadm2 |
| ENSRNOG00000052015 | 2.780758 | 0.04246 | up | 19:-:14951616-14953755 | 19:-:14951616-14953755 | AABR07042937.1 |
| ENSRNOG00000058080 | -2.51066189983817 | 0.042515 | down | 7:-:2221800-2221953 | 7:-:2221800-2221953 | 5_8S_rRNA |
| ENSRNOG00000052450 | -2.72050063367093 | 0.042725 | down | 7:+:144725291-144725621 | 7:+:144725291-144725621 | AABR07058936.1 |
| ENSRNOG00000019718 | 1.79013 | 0.042755 | up | 16:+:8128689-8177841 | 16:+:8128689-8177841 | Galnt15 |
| ENSRNOG00000054626 | -1.11908665854371 | 0.042787 | down | 5:-:136020941-136023511 | 5:-:136020941-136023511 | Rps8 |
| ENSRNOG00000008843 | -1.03501234638081 | 0.042797 | down | 10:+:13797562-13810913 | 10:+:13797562-13810913 | Eci1 |
| ENSRNOG00000036794 | -4.07430376067775 | 0.043052 | down | 10:+:90217924-90226537 | 10:+:90217924-90226537 | Asb16 |
| ENSRNOG00000000844 | -4.17400404357783 | 0.043101 | down | 20:-:5061658-5064469 | 20:-:5061658-5064469 | Ly6g6d |
| ENSRNOG00000006623 | -1.42456942995526 | 0.043129 | down | 3:-:46327383-46361092 | 3:-:46327383-46361092 | Cd302 |
| ENSRNOG00000006178 | 1.917923 | 0.043195 | up | 6:+:134958854-135085769 | 6:+:134958854-135085769 | Dync1h1 |
| ENSRNOG00000036837 | -3.20596796740828 | 0.043199 | down | 7:-:144872747-144880092 | 7:-:144872747-144880092 | Nfe2 |
| ENSRNOG00000023809 | 2.986174 | 0.043216 | up | 8:+:29453643-29962825 | 8:+:29453643-29962825 | Opcml |
| ENSRNOG00000005655 | -5.01990212630394 | 0.043306 | down | 5:+:8459660-8574655 | 5:+:8459660-8574655 | Cpa6 |
| ENSRNOG00000050206 | 2.295212 | 0.043308 | up | 1:+:217151166-217592763 | 1:+:217151166-217592763 | Shank2 |
| ENSRNOG00000054620 | 2.578178 | 0.043424 | up | 1:-:48880111-48891130 | 1:-:48880111-48891130 | AC135026.1 |
| ENSRNOG00000020514 | 2.947325 | 0.043438 | up | 1:+:185673177-186182829 | 1:+:185673177-186182829 | Sox6 |
| ENSRNOG00000001611 | -1.26495323425988 | 0.043525 | down | 11:-:47116886-47122095 | 11:-:47116886-47122095 | Rpl24 |
| ENSRNOG00000060635 | 1.582213 | 0.043532 | up | 3:-:83052724-83134363 | 3:-:83052724-83134363 | AABR07052897.3 |
| ENSRNOG00000017060 | 1.798748 | 0.043558 | up | 17:-:65535403-65955606 | 17:-:65535403-65955606 | Ryr2 |
| ENSRNOG00000008237 | 1.246613 | 0.043633 | up | 5:+:58505500-58715576 | 5:+:58505500-58715576 | Unc13b |
| ENSRNOG00000022490 | -1.55558284363708 | 0.043634 | down | 3:-:9096044-9096481 | 3:-:9096044-9096481 | LOC100361008 |
| ENSRNOG00000015226 | 1.580977 | 0.043676 | up | 2:-:202891537-203043847 | 2:-:202891537-203043847 | Man1a2 |
| ENSRNOG00000056221 | 2.643234 | 0.043746 | up | X:+:111735820-111741023 | X:+:111735820-111741023 | AABR07040839.1 |
| ENSRNOG00000001593 | 1.196096 | 0.043764 | up | 3:-:61433863-61494778 | 3:-:61433863-61494778 | Lnpk |
| ENSRNOG00000055959 | -3.27896611882058 | 0.043778 | down | KL568149.1:+:194682-194805 | KL568149.1:+:194682-194805 | AC242253.1 |
| ENSRNOG00000014936 | -1.18409514016755 | 0.04384 | down | 1:-:213750192-213751405 | 1:-:213750192-213751405 | Ifitm2 |
| ENSRNOG00000053498 | -2.53939849528196 | 0.043901 | down | 7:+:140781799-140786906 | 7:+:140781799-140786906 | Dnajc22 |
| ENSRNOG00000007256 | 1.274642 | 0.043903 | up | 5:-:28504558-28737719 | 5:-:28504558-28737719 | Necab1 |
| ENSRNOG00000049213 | 2.948682 | 0.043912 | up | 2:-:181530832-181531978 | 2:-:181530832-181531978 | Npy2r |
| ENSRNOG00000050090 | 1.079516 | 0.043985 | up | 2:-:210249663-210299770 | 2:-:210249663-210299770 | Slc6a17 |
| ENSRNOG00000018237 | -1.23729866102821 | 0.044007 | down | 1:-:219291679-219294147 | 1:-:219291679-219294147 | Gstp1 |
| ENSRNOG00000037380 | -2.02089796717087 | 0.044026 | down | 1:+:91746486-91747174 | 1:+:91746486-91747174 | AC136661.1 |
| ENSRNOG00000008861 | 1.682322 | 0.044181 | up | 5:+:15087349-15090583 | 5:+:15087349-15090583 | AABR07047011.1 |
| ENSRNOG00000006397 | 3.721398 | 0.04428 | up | 3:-:103966451-104018861 | 3:-:103966451-104018861 | Chrm5 |
| ENSRNOG00000012714 | 2.687593 | 0.044305 | up | 20:-:12927320-12938891 | 20:-:12927320-12938891 | RGD1564149 |
| ENSRNOG00000057235 | 2.572354 | 0.044325 | up | 9:+:41208046-41208893 | 9:+:41208046-41208893 | AABR07067355.1 |
| ENSRNOG00000050955 | 2.25526 | 0.04434 | up | 10:+:1920529-1952605 | 10:+:1920529-1952605 | AABR07029002.3 |
| ENSRNOG00000005758 | 2.191647 | 0.044346 | up | 7:-:24043126-24313885 | 7:-:24043126-24313885 | Btbd11 |
| ENSRNOG00000009288 | 3.028144 | 0.044402 | up | 15:-:110385217-110612681 | 15:-:110385217-110612681 | Fgf14 |
| ENSRNOG00000014975 | 3.720136 | 0.04447 | up | 5:+:76092287-76102590 | 5:+:76092287-76102590 | Zfp483 |
| ENSRNOG00000000638 | 1.451691 | 0.044544 | up | 20:+:22060224-22086108 | 20:+:22060224-22086108 | Zfp365 |
| ENSRNOG00000004062 | -2.89862091437534 | 0.044561 | down | 13:-:47342079-47377703 | 13:-:47342079-47377703 | C4bpa |
| ENSRNOG00000062298 | -1.24548502938894 | 0.044598 | down | 1:-:101120325-101123402 | 1:-:101120325-101123402 | Rpl13a |
| ENSRNOG00000017002 | 1.753698 | 0.045162 | up | 1:+:277537585-277538985 | 1:+:277537585-277538985 | Adrb1 |
| ENSRNOG00000019207 | 1.908249 | 0.045272 | up | 1:+:100297152-100344377 | 1:+:100297152-100344377 | Shank1 |
| ENSRNOG00000001597 | 1.967554 | 0.045379 | up | 3:-:60721135-60795951 | 3:-:60721135-60795951 | Atf2 |
| ENSRNOG00000048402 | -10.0013985359237 | 0.045435 | down | 6:-:138092131-138093643 | 6:-:138092131-138093643 | Igh-6 |
| ENSRNOG00000027026 | 3.285707 | 0.045472 | up | 4:-:82836260-83137527 | 4:-:82836260-83137527 | Jazf1 |
| ENSRNOG00000014214 | -1.28396536142795 | 0.045472 | down | 1:+:174132798-174135816 | 1:+:174132798-174135816 | Rpl27a |
| ENSRNOG00000014783 | -1.17982464946839 | 0.045516 | down | 1:+:213737788-213742637 | 1:+:213737788-213742637 | Pgghg |
| ENSRNOG00000010872 | -1.3558053665988 | 0.04565 | down | 6:-:136142956-136145837 | 6:-:136142956-136145837 | Ckb |
| ENSRNOG00000019482 | 1.435487 | 0.045713 | up | 19:-:11513201-11669578 | 19:-:11513201-11669578 | Gnao1 |
| ENSRNOG00000033256 | -1.90866420200433 | 0.045816 | down | 8:+:22559098-22576154 | 8:+:22559098-22576154 | LOC691141 |
| ENSRNOG00000012410 | -1.47897395965331 | 0.045823 | down | 2:-:189900667-189903219 | 2:-:189900667-189903219 | S100a1 |
| ENSRNOG00000013023 | -1.27312843526249 | 0.0459 | down | 1:-:77836196-77844189 | 1:-:77836196-77844189 | Nop53 |
| ENSRNOG00000001225 | -1.5201815779133 | 0.045936 | down | 20:+:11863082-11896774 | 20:+:11863082-11896774 | Fam207a |
| ENSRNOG00000042576 | 1.572583 | 0.045955 | up | 3:-:94624071-94657377 | 3:-:94624071-94657377 | Tcp11l1 |
| ENSRNOG00000021265 | 1.528199 | 0.046042 | up | 3:+:124896618-124935226 | 3:+:124896618-124935226 | Cds2 |
| ENSRNOG00000054165 | 2.798925 | 0.046092 | up | 6:-:26426869-26445787 | 6:-:26426869-26445787 | LOC103692570 |
| ENSRNOG00000017418 | -1.16655662812977 | 0.046175 | down | 1:-:164435878-164441167 | 1:-:164435878-164441167 | Rps3 |
| ENSRNOG00000051948 | 1.583148 | 0.046206 | up | X:+:156812064-156837227 | X:+:156812064-156837227 | Hcfc1 |
| ENSRNOG00000007743 | -2.05583256591565 | 0.046423 | down | 4:+:172119331-172134607 | 4:+:172119331-172134607 | Mgst1 |
| ENSRNOG00000039955 | -3.67016479127766 | 0.046521 | down | 1:-:20125966-20155960 | 1:-:20125966-20155960 | Samd3 |
| ENSRNOG00000061519 | 2.523758 | 0.046558 | up | 6:+:43234526-43343551 | 6:+:43234526-43343551 | Asap2 |
| ENSRNOG00000021151 | -1.38912103807092 | 0.04666 | down | 1:+:222229835-222231944 | 1:+:222229835-222231944 | Ppp1r14b |
| ENSRNOG00000016971 | 1.570392 | 0.046825 | up | 19:-:41559487-41567013 | 19:-:41559487-41567013 | Zfp612 |
| ENSRNOG00000008683 | 1.476518 | 0.046927 | up | 6:+:22696397-23203775 | 6:+:22696397-23203775 | Alk |
| ENSRNOG00000010119 | 1.730679 | 0.046939 | up | 2:-:118721334-118745766 | 2:-:118721334-118745766 | Zmat3 |
| ENSRNOG00000017914 | -1.32655254778119 | 0.046995 | down | 1:-:170317113-170318935 | 1:-:170317113-170318935 | Cavin3 |
| ENSRNOG00000003585 | 2.728771 | 0.047001 | up | X:+:14019961-14109568 | X:+:14019961-14109568 | Sytl5 |
| ENSRNOG00000012393 | -1.89872328409088 | 0.047105 | down | 2:+:189906022-189912516 | 2:+:189906022-189912516 | S100a13 |
| ENSRNOG00000002963 | -1.07847711591747 | 0.047179 | down | 10:-:91040005-91047177 | 10:-:91040005-91047177 | C1ql1 |
| ENSRNOG00000014871 | -1.7740112436047 | 0.047193 | down | 8:+:98745310-98761840 | 8:+:98745310-98761840 | Zic4 |
| ENSRNOG00000007178 | -3.23160615113635 | 0.047199 | down | 4:+:99239115-99243351 | 4:+:99239115-99243351 | Cd8a |
| ENSRNOG00000024603 | -1.46562172471523 | 0.047316 | down | 7:-:12282109-12283608 | 7:-:12282109-12283608 | Rps15 |
| ENSRNOG00000016278 | -2.33948483279586 | 0.047327 | down | 19:-:10619220-10620671 | 19:-:10619220-10620671 | Ccl17 |
| ENSRNOG00000015148 | 3.525962 | 0.047363 | up | 16:+:3293599-3679222 | 16:+:3293599-3679222 | Erc2 |
| ENSRNOG00000058308 | 1.077029 | 0.047368 | up | 10:-:68248655-68517564 | 10:-:68248655-68517564 | Asic2 |
| ENSRNOG00000037509 | -2.02491233188307 | 0.047408 | down | 12:-:51845796-51877624 | 12:-:51845796-51877624 | Chek2 |
| ENSRNOG00000061814 | 1.542932 | 0.047414 | up | 11:+:87242522-87292955 | 11:+:87242522-87292955 | Dgcr2 |
| ENSRNOG00000011009 | 3.611436 | 0.047438 | up | 19:+:784618-824420 | 19:+:784618-824420 | Cmtm4 |
| ENSRNOG00000020105 | -2.69543436835396 | 0.04744 | down | 9:+:98490608-98500867 | 9:+:98490608-98500867 | Klhl30 |
| ENSRNOG00000001245 | -1.91559272263277 | 0.047455 | down | 20:+:12429315-12629981 | 20:+:12429315-12629981 | Pcbp3 |
| ENSRNOG00000012747 | 1.467803 | 0.047472 | up | 17:+:7675531-7797863 | 17:+:7675531-7797863 | Spock1 |
| ENSRNOG00000015233 | -1.00973258043376 | 0.047509 | down | 8:-:60028789-60086403 | 8:-:60028789-60086403 | Etfa |
| ENSRNOG00000050188 | 2.912084 | 0.047557 | up | 10:-:95434374-95434688 | 10:-:95434374-95434688 | AABR07030603.1 |
| ENSRNOG00000046566 | 3.848722 | 0.047561 | up | 1:+:173607101-173625550 | 1:+:173607101-173625550 | Tub |
| ENSRNOG00000010986 | -1.07559786705758 | 0.047615 | down | 8:-:122812459-122841477 | 8:-:122812459-122841477 | Cmtm7 |
| ENSRNOG00000046500 | 1.536325 | 0.047636 | up | 1:+:81395841-81399672 | 1:+:81395841-81399672 | Irgq |
| ENSRNOG00000016560 | -1.51626937962434 | 0.047677 | down | 17:+:15845931-15858771 | 17:+:15845931-15858771 | Card19 |
| ENSRNOG00000056447 | -3.93020593755631 | 0.047819 | down | 3:+:18787606-18787893 | 3:+:18787606-18787893 | AABR07051658.1 |
| ENSRNOG00000017939 | 1.360602 | 0.047846 | up | 3:-:60513077-60611924 | 3:-:60513077-60611924 | Chn1 |
| ENSRNOG00000004048 | 1.429333 | 0.047848 | up | 7:+:132857628-133018584 | 7:+:132857628-133018584 | Lrrk2 |
| ENSRNOG00000011885 | -1.16424390889538 | 0.04789 | down | 1:+:91596397-91657395 | 1:+:91596397-91657395 | Rhpn2 |
| ENSRNOG00000029366 | 1.318276 | 0.047905 | up | 1:-:198451324-198454914 | 1:-:198451324-198454914 | Prrt2 |
| ENSRNOG00000017672 | -1.58414616819795 | 0.04794 | down | 17:-:69806065-69827112 | 17:-:69806065-69827112 | Akr1c14 |
| ENSRNOG00000054019 | -2.22027561650922 | 0.047946 | down | 7:-:73213826-73213957 | 7:-:73213826-73213957 | SNORA72 |
| ENSRNOG00000055382 | 1.474674 | 0.048077 | up | 2:+:50099576-50499799 | 2:+:50099576-50499799 | Hcn1 |
| ENSRNOG00000060961 | 2.896991 | 0.048078 | up | 8:-:61430419-61436104 | 8:-:61430419-61436104 | AABR07070246.1 |
| ENSRNOG00000003508 | 2.073814 | 0.048079 | up | 10:-:16821880-16910641 | 10:-:16821880-16910641 | Ergic1 |
| ENSRNOG00000026260 | -1.25099913152637 | 0.048174 | down | 5:-:154388140-154394328 | 5:-:154388140-154394328 | Rpl11 |
| ENSRNOG00000052173 | -1.48926950631094 | 0.048204 | down | 2:+:58448917-58458517 | 2:+:58448917-58458517 | AABR07008439.1 |
| ENSRNOG00000019305 | -1.88110339986358 | 0.04822 | down | 18:+:57286322-57347577 | 18:+:57286322-57347577 | Sh3tc2 |
| ENSRNOG00000057499 | 2.64866 | 0.048245 | up | 9:-:110055894-110056257 | 9:-:110055894-110056257 | AABR07068587.1 |
| ENSRNOG00000011406 | -2.89692673514089 | 0.048315 | down | 10:+:70884531-70886355 | 10:+:70884531-70886355 | Ccl4 |
| ENSRNOG00000048824 | 2.382216 | 0.048333 | up | 18:-:73831326-73873280 | 18:-:73831326-73873280 | Rnf165 |
| ENSRNOG00000003313 | 3.479904 | 0.048336 | up | X:-:30612903-30831483 | X:-:30612903-30831483 | Glra2 |
| ENSRNOG00000014532 | -2.48740117943254 | 0.048337 | down | 3:+:154786215-154813464 | 3:+:154786215-154813464 | Lbp |
| ENSRNOG00000019453 | -1.12955674119173 | 0.048389 | down | 1:-:65660470-65664767 | 1:-:65660470-65664767 | Rps5 |
| ENSRNOG00000000905 | 2.002635 | 0.048513 | up | 12:-:6671940-6703979 | 12:-:6671940-6703979 | Tex26 |
| ENSRNOG00000020821 | 1.711521 | 0.048566 | up | 1:+:88750462-88765220 | 1:+:88750462-88765220 | LOC108348122 |
| ENSRNOG00000037476 | 1.320431 | 0.048598 | up | 12:-:52106506-52187164 | 12:-:52106506-52187164 | Galnt9 |
| ENSRNOG00000021170 | -1.48354578138792 | 0.048636 | down | 2:-:198112076-198120120 | 2:-:198112076-198120120 | Plekho1 |
| ENSRNOG00000012550 | -1.46455916963481 | 0.048639 | down | 5:-:134919044-134927235 | 5:-:134919044-134927235 | Uqcrh |
| ENSRNOG00000013167 | -1.23675736882656 | 0.048711 | down | 16:-:36077617-36080191 | 16:-:36077617-36080191 | Hmgb2 |
| ENSRNOG00000054751 | 1.813385 | 0.048713 | up | 2:+:58534476-58590758 | 2:+:58534476-58590758 | Lmbrd2 |
| ENSRNOG00000004107 | -1.17286548820106 | 0.04876 | down | 10:-:85721394-85725429 | 10:-:85721394-85725429 | Rpl23 |
| ENSRNOG00000019573 | -1.19081387886371 | 0.048888 | down | 19:-:37913336-37916813 | 19:-:37913336-37916813 | Lcat |
| ENSRNOG00000024595 | 3.313915 | 0.048924 | up | 3:+:55094637-55338445 | 3:+:55094637-55338445 | Cers6 |
| ENSRNOG00000050655 | 1.047511 | 0.048945 | up | 20:+:28920616-28971966 | 20:+:28920616-28971966 | P4ha1 |
| ENSRNOG00000023304 | 2.657972 | 0.049094 | up | 13:+:98924962-98967735 | 13:+:98924962-98967735 | Lin9 |
| ENSRNOG00000030726 | -2.07631410387494 | 0.049143 | down | 10:+:56710464-56723611 | 10:+:56710464-56723611 | Asgr2 |
| ENSRNOG00000032902 | -1.65026666192643 | 0.049172 | down | 10:-:40380388-40381886 | 10:-:40380388-40381886 | Ybx1-ps3 |
| ENSRNOG00000027837 | 2.874637 | 0.049227 | up | X:-:123052024-123092217 | X:-:123052024-123092217 | AABR07041232.1 |
| ENSRNOG00000042289 | 2.365368 | 0.04925 | up | 11:+:57207656-57260568 | 11:+:57207656-57260568 | Plcxd2 |
| ENSRNOG00000000436 | -1.80115998785523 | 0.049251 | down | 20:-:4378206-4380600 | 20:-:4378206-4380600 | Egfl8 |
| ENSRNOG00000006958 | 1.578113 | 0.0493 | up | 12:-:13372302-13374500 | 12:-:13372302-13374500 | AC126572.1 |
| ENSRNOG00000029235 | 2.321732 | 0.049302 | up | 17:-:71723620-71897972 | 17:-:71723620-71897972 | Sfmbt2 |
| ENSRNOG00000006553 | -2.58838130291314 | 0.049306 | down | 5:-:102415847-102786331 | 5:-:102415847-102786331 | Bnc2 |
| ENSRNOG00000028021 | -1.37461967961189 | 0.049505 | down | 3:-:4341249-4341771 | 3:-:4341249-4341771 | Rps13 |
| ENSRNOG00000042560 | 1.469488 | 0.049506 | up | 16:+:71058022-71075320 | 16:+:71058022-71075320 | Bag4 |
| ENSRNOG00000037495 | -3.39423872965 | 0.049547 | down | 6:-:143590014-143590448 | 6:-:143590014-143590448 | AABR07065883.1 |
| ENSRNOG00000010208 | -1.83450143689953 | 0.049692 | down | X:-:1364786-1369384 | X:-:1364786-1369384 | Timp1 |
| ENSRNOG00000011151 | 2.105528 | 0.049709 | up | 1:+:161401527-161880923 | 1:+:161401527-161880923 | Tenm4 |
| ENSRNOG00000017503 | 2.958948 | 0.049832 | up | 18:-:56626712-56728968 | 18:-:56626712-56728968 | Ppargc1b |
| ENSRNOG00000038190 | 1.758473 | 0.04988 | up | 18:-:86420361-86878142 | 18:-:86420361-86878142 | Dok6 |
| ENSRNOG00000010853 | 3.564081 | 0.0499 | up | 1:-:123899657-124039196 | 1:-:123899657-124039196 | Chrna7 |
| ENSRNOG00000016117 | -1.03908156770369 | 0.049908 | down | 1:-:256585410-256734730 | 1:-:256585410-256734730 | Myof |
| ENSRNOG00000011202 | 1.621837 | 0.049916 | up | 3:-:176527516-176548208 | 3:-:176527516-176548208 | Chrna4 |
| ENSRNOG00000021497 | 1.302948 | 0.04993 | up | 13:-:95081390-95348913 | 13:-:95081390-95348913 | Akt3 |

Abbreviations: DEGs: differentially expressed genes; VD: vascular dementia; INS: insulin; FC: fold change
