# Supplementary material for: Integrated transcriptome and metabolome analysis to investigate the mechanism of intranasal insulin treatment in a rat model of vascular dementia
Source: Front Pharmacol. 2023 May 15;14:1182803. doi: 10.3389/fphar.2023.1182803 (PMC10225696; doi:10.3389/fphar.2023.1182803)
Supplement: Supplementary file 8 [file Table4.docx]

Table S4 Top 30 GO enrichment of DEGs between INS+VD and VD groups

| Class | GO term | GO name | Deg number of this term |
| --- | --- | --- | --- |
| cellular_component | GO:0005623 | cell | 450 |
| cellular_component | GO:0044464 | cell part | 448 |
| biological_process | GO:0009987 | cellular process | 448 |
| biological_process | GO:0044699 | single-organism process | 430 |
| molecular_function | GO:0005488 | binding | 387 |
| biological_process | GO:0065007 | biological regulation | 369 |
| biological_process | GO:0050789 | regulation of biological process | 351 |
| cellular_component | GO:0043226 | organelle | 334 |
| cellular_component | GO:0016020 | membrane | 333 |
| biological_process | GO:0008152 | metabolic process | 303 |
| biological_process | GO:0050896 | response to stimulus | 302 |
| biological_process | GO:0032501 | multicellular organismal process | 277 |
| biological_process | GO:0032502 | developmental process | 248 |
| cellular_component | GO:0044425 | membrane part | 239 |
| biological_process | GO:0023052 | signaling | 239 |
| biological_process | GO:0051179 | localization | 225 |
| biological_process | GO:0048518 | positive regulation of biological process | 218 |
| biological_process | GO:0071840 | cellular component organization or biogenesis | 206 |
| cellular_component | GO:0044422 | organelle part | 177 |
| biological_process | GO:0048519 | negative regulation of biological process | 176 |
| cellular_component | GO:0032991 | macromolecular complex | 163 |
| cellular_component | GO:0005576 | extracellular region | 155 |
| cellular_component | GO:0044421 | extracellular region part | 142 |
| molecular_function | GO:0003824 | catalytic activity | 128 |
| biological_process | GO:0002376 | immune system process | 95 |
| molecular_function | GO:0060089 | molecular transducer activity | 85 |
| biological_process | GO:0040011 | locomotion | 79 |
| cellular_component | GO:0030054 | cell junction | 77 |
| cellular_component | GO:0045202 | synapse | 75 |
| biological_process | GO:0051704 | multi-organism process | 75 |

Abbreviations: DEGs: differentially expressed genes; VD: vascular dementia; INS: insulin; GO: Gene Ontology
