# Supplementary material for: Integrated transcriptome and metabolome analysis to investigate the mechanism of intranasal insulin treatment in a rat model of vascular dementia
Source: Front Pharmacol. 2023 May 15;14:1182803. doi: 10.3389/fphar.2023.1182803 (PMC10225696; doi:10.3389/fphar.2023.1182803)
Supplement: Supplementary file 9 [file Table6.docx]

Table S6 Top30 KEGG pathways of DEGs between INS+VD and VD groups

| #Term | ID | Input number | P-Value | Corrected P-Value |
| --- | --- | --- | --- | --- |
| Ribosome | rno03010 | 34 | 5.4E-14 | 5.32E-11 |
| Neuroactive ligand-receptor interaction | rno04080 | 36 | 9.29E-09 | 8.33E-07 |
| Calcium signaling pathway | rno04020 | 26 | 9.97E-08 | 7.56E-06 |
| Morphine addiction | rno05032 | 18 | 2.65E-07 | 1.86E-05 |
| Retrograde endocannabinoid signaling | rno04723 | 17 | 4.03E-06 | 0.000181 |
| cAMP signaling pathway | rno04024 | 24 | 4.51E-06 | 0.000193 |
| Cholinergic synapse | rno04725 | 17 | 8.96E-06 | 0.000354 |
| Dopaminergic synapse | rno04728 | 18 | 1.22E-05 | 0.000436 |
| Nicotine addiction | rno05033 | 10 | 2.36E-05 | 0.000803 |
| Circadian entrainment | rno04713 | 14 | 6.69E-05 | 0.002127 |
| Insulin secretion | rno04911 | 13 | 0.000113 | 0.003421 |
| GABAergic synapse | rno04727 | 13 | 0.000173 | 0.004865 |
| Estrogen signaling pathway | rno04915 | 13 | 0.000257 | 0.00703 |
| Salivary secretion | rno04970 | 11 | 0.000366 | 0.009754 |
| Oxytocin signaling pathway | rno04921 | 17 | 0.000389 | 0.010095 |
| Glutamatergic synapse | rno04724 | 14 | 0.000477 | 0.012047 |
| Axon guidance | rno04360 | 18 | 0.000558 | 0.013766 |
| Serotonergic synapse | rno04726 | 14 | 0.000655 | 0.015763 |
| Chagas disease (American trypanosomiasis) | rno05142 | 13 | 0.000686 | 0.016111 |
| Pertussis | rno05133 | 10 | 0.000862 | 0.019767 |
| HTLV-I infection | rno05166 | 23 | 0.001215 | 0.026615 |
| Gap junction | rno04540 | 11 | 0.001424 | 0.03038 |
| Cocaine addiction | rno05030 | 8 | 0.001448 | 0.03038 |
| Antigen processing and presentation | rno04612 | 10 | 0.001559 | 0.031512 |
| Chemokine signaling pathway | rno04062 | 17 | 0.001566 | 0.031512 |
| Systemic lupus erythematosus | rno05322 | 12 | 0.001874 | 0.036961 |
| Melanogenesis | rno04916 | 11 | 0.002336 | 0.04266 |
| Thyroid hormone synthesis | rno04918 | 9 | 0.002446 | 0.043845 |
| Proteoglycans in cancer | rno05205 | 18 | 0.002568 | 0.045108 |
| Pathways in cancer | rno05200 | 28 | 0.003382 | 0.053782 |

Abbreviations: KEGG; Kyoto Encyclopedia of Genes and Genomes; DEGs: differentially expressed genes; VD: vascular dementia; INS: insulin;
